# Modular Synthesis of Highly Substituted 3-Azapyrroles by Rh(II)-Catalyzed N–H Bond Insertion and Cyclodehydration

**DOI:** 10.1021/acs.joc.2c00434

**Published:** 2022-05-03

**Authors:** Matthew B. Williams, Alistair Boyer

**Affiliations:** School of Chemistry, University of Glasgow, Glasgow G12 8QQ, United Kingdom

## Abstract

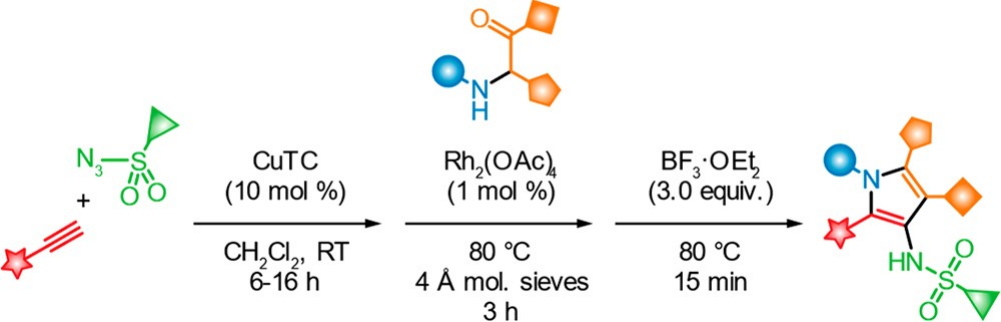

A modular synthesis of highly substituted 3-azapyrroles has been developed using a three-step sequence comprising copper-catalyzed alkyne–azide cycloaddition (CuAAC), N–H bond insertion, and cyclodehydration. 1-Sulfonyl-1,2,3-triazoles (1-STs) can be accessed from common alkyne and sulfonyl azide building blocks by CuAAC using CuTC. Rhodium(II)-acetate-promoted 1-ST denitrogenation results in highly electrophilic rhodium azavinyl carbenes that, here, underwent insertion into the N–H bond of secondary α-aminoketones to form 1,2-aminoalkenes. These products were cyclized and dehydrated using BF_3_·OEt_2_ into highly substituted 3-azapyrroles. The three steps (CuAAC, N–H bond insertion, and cyclodehydration) could be telescoped into a one-pot process. The method proved to be highly efficient and tolerated a wide range of substituents.

## Introduction

**Figure 1:**
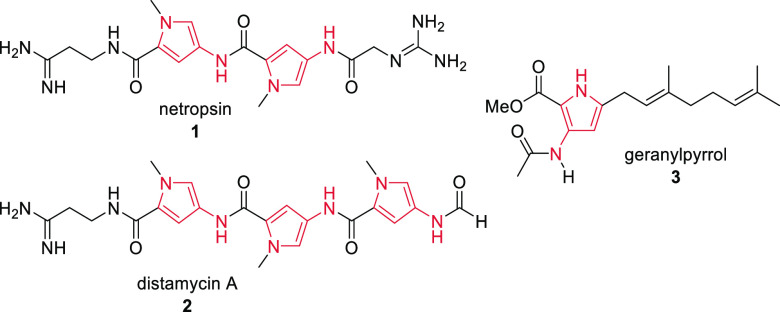
Selected biologically active 3-azapyrroles.

Pyrroles are ubiquitous five-membered nitrogen-containing heteroaromatic compounds that display valuable properties, making them key fragments in a wide variety of natural products,^1^ pharmaceuticals,^2^ and functional materials.^3^ Several valuable pyrroles have 3-aza substitution, including the DNA minor groove binders netropsin^4^**1** and distamycin A^5^**2** as well as other natural products such as geranylpyrrol **3** (Figure 1).^6^

The value of pyrroles has inspired many synthetic approaches spanning the whole history of organic chemistry.^7^ However, these established methods are not always easily adapted to the synthesis of 3-azapyrroles.^8^ Therefore, development of novel synthetic strategies toward this privileged heterocycle is necessary. Ideally, newly developed methods should begin from cheap and readily available starting materials and be straightforward to carry out practically, highly atom efficient, modular, and tolerant of a wide range of functional groups.

The reactivity of 1-sulfonyl-1,2,3-triazoles **4** (1-STs) has been developed such that they can be considered readily accessed building blocks^9^ for the facile synthesis of a wide range of value-added products.^10^ The sulfonyl group provides an ideal balance to the triazole heterocycle, bringing stability but also allowing on demand reactivity when an appropriate catalyst system is deployed. The majority of examples of this strategy involve triazoles and rhodium(II) carboxylate catalysts, although, recently, palladium(0) catalysts have shown analogous reactivity with 1-trifluoromethanesulfonylbenzotriazoles **5**.^11^

**Scheme 1:**
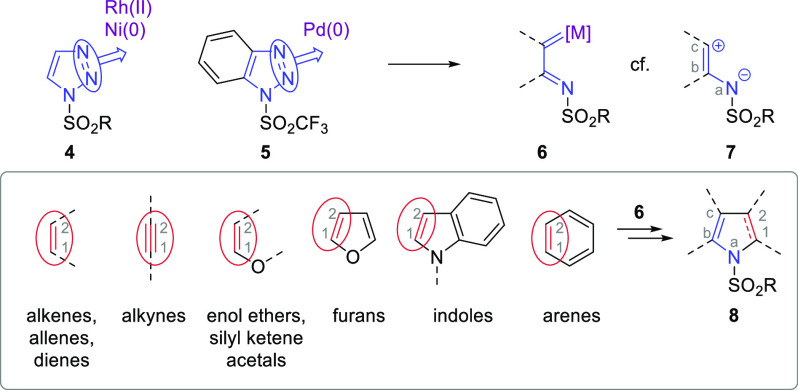
General Reactivity of 1-Sufonyl-1,2,3-triazoles and Selected Application to the Synthesis of Pyrroles and Indoles

**Scheme 2:**
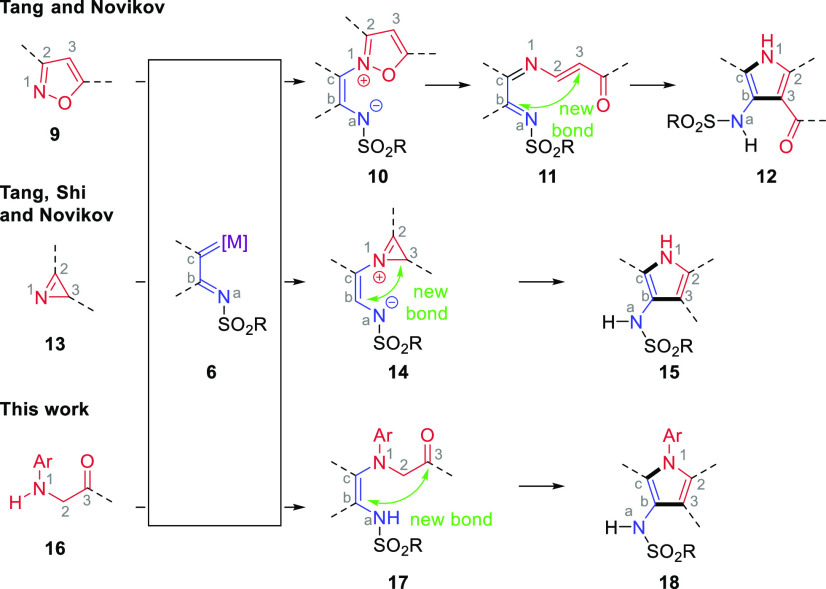
Approaches to 3-Azapyrroles from 1-Sufonyl-1,2,3-triazoles and This Approach

The key reactivity of 1-STs arises from the catalyst promoting Dimroth equilibration and denitrogenation of the nitrogen-rich heterocycle to give a highly reactive organometallic intermediate **6** (Scheme 1). In terms of reactivity, the organometallic species **6** can be considered as a three-atom ylidic synthon **7** with its positive component on the carbon and negative component on the nitrogen atom. Therefore, reaction with a suitable two-carbon π component results in the formation of a new five-membered nitrogen heterocycle **8**, i.e., a pyrrole, indole, or reduced version thereof. This reactivity has been developed across a wide spectrum of substrates: alkenes,^11b,11d,12^ alkynes,^13^ enol ethers,^14^ furans,^15^ indoles,^16^ and even arene moieties.^17^ There have also been some unique approaches to pyrroles and indoles from unsaturated carbonyl species,^18^ alcoholic unsaturated carbonyl species,^19^ unsaturated 1-STs,^20^ and vinyl anilines.^21^ The versatility of 1-ST reactivity means that many reactions are possible that result in heterocycle formation by the inclusion of a nitrogen-containing tether^22,23^ or by providing valuable access to substrates that can be converted into five-membered azaheterocycles in short order.^24,25^ In the majority of these examples, the mechanism dictates that the nitrogen atom remaining following denitrogenation of the triazole becomes the heteroatom component of the heterocycle that is generated. However, in reactions of 1-STs with certain nitrogen-containing substrates, the triazole nitrogen can become a 3-aza substituent of the pyrrole (Scheme 2).

Upon treatment with a Rh(II) carboxylate, 1-STs formed an electron-deficient metallocarbene **6** that reacted with the nitrogen lone pair of isoxazoles **10**. Ring opening of the isoxazole **11** was followed by cyclization to form a pyrrole **12** with a 3-azasulfonyl substituent.^26^ In a similar fashion but with azirenes, the azirene **13** lone pair reacted with the highly electron-deficient rhodium carbene **6** followed by collapse of the intermediate to form a pyrrole **15**, again with a 3-azasulfonyl substituent.^27^

The insertion of metallocarbenes into X–H bonds has been developed into a valuable reaction,^28^ and this formation of C–N bonds from N–H bonds is another class of transformation that has been demonstrated with 1-STs.^21,29^ Here, we recognized that α-aminoketones are the hydrated equivalent of azirenes. Therefore, bringing together an α-aminoketone **16** and a 1-ST through N–H bond insertion (**17**) and then performing cyclodehydration would result in a strategically complementary approach to valuable 3-azapyrroles **18**.

## Results

**Scheme 3:**
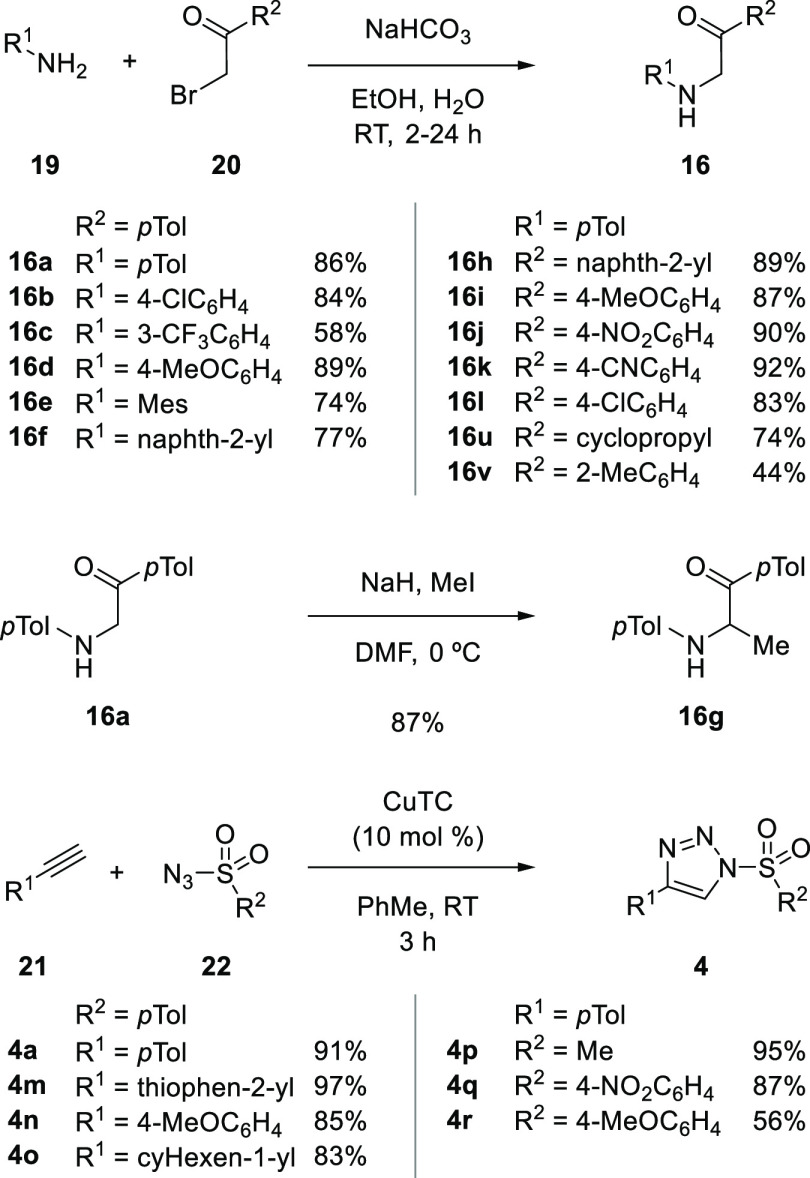
Synthesis of α-Aminoketones **16** and 1-STs **4** Used in This Work

The substrates for this study, namely, α-aminoketones and 1-STs are both readily accessed from readily available materials (Scheme 3). The α-aminoketones **16** were formed by simple S_N_2 displacement of commercial α-haloketones **20** by anilines **19**. Additional substitution could be introduced to the α-position of the ketone owing to its acidic nature (**16g**). The 1-STs **4** were formed in excellent yield by copper-catalyzed azide–alkyne cycloaddition (CuAAC), and the modular nature of 1-ST synthesis is a key strength to this methodology. It is noteworthy that the use of copper(I) thiophene-2-carboxylate (CuTC) has been specifically developed for 1-ST synthesis.^9^

Next, the focus was establishing the optimum conditions for insertion of the metallocarbene derived from the 1-ST into the N–H bond of an appropriate aniline to bring together all the requisite atoms for the planned pyrrole synthesis (Table 1). A 4-tolyl-1-tosyltriazole **4a** was treated with a slight excess of α-aminoketone **16a** and 5 mol % of Rh_2_(OAc)_4_ in toluene at 80 °C with 4 Å molecular sieves.^30^ These conditions resulted in the formation of the N–H insertion product **17a** in high yield (81%, entry 1).^31^ Commonly employed Rh_2_(octanoate)_4_ and Rh_2_(esp)_2_^32^ also promoted the N–H bond insertion but with lower efficiency, whereas no reaction occurred with bulky catalysts Rh_2_(triphenylacetate)_4_ and Rh_2_(*S*-*t*PTTL)_4_^33^ (entries 4 and 5). The choice of solvent had a minor effect on the reaction outcome, but the yield was improved to 93% when using toluene (entries 6–8). Increasing the temperature from 60 to 80 °C provided a small increase in yield (entry 9), but increasing the temperature further caused the yield to decrease (entries 10 and 11); no reaction occurred below 60 °C (entry 12). These findings were congruous with previous work that showed Rh_2_(OAc)_4_ and Rh_2_(octanoate)_4_ to be the most effective for related N–H bond insertions of α-amino esters, carbamates, and carbazoles.^21,29^ However, this process was more tolerant to solvent compared with other processes, specifically with toluene being generally incompatible in the other examples.

**Table 1:**
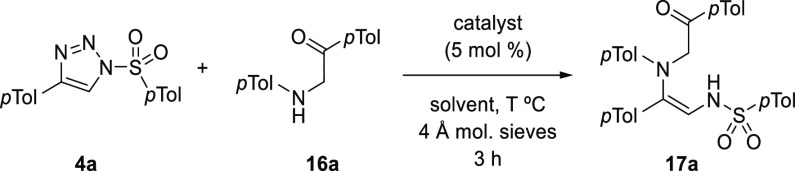
Optimization of N–H Insertion^a^

entry	catalyst	solvent	*T* (°C)	yield (%)
1	Rh_2_(OAc)_4_	CHCl_3_	60	81
2	Rh_2_(esp)_2_	CHCl_3_	60	70
3	Rh_2_(octanoate)_4_	CHCl_3_	60	38
4	Rh_2_(TPA)_4_	CHCl_3_	60	no reaction
5	Rh_2_(*S*-*t*PTTL)_4_	CHCl_3_	60	no reaction
6	Rh_2_(OAc)_4_	(CH_2_Cl)_2_	60	87
7	Rh_2_(OAc)_4_	CH_2_Cl_2_	60	91
8	Rh_2_(OAc)_4_	PhMe	60	93
**9**	**Rh**_**2**_**(OAc)**_**4**_	**PhMe**	**80**	**94**
10	Rh_2_(OAc)_4_	PhMe	100	85
11	Rh_2_(OAc)_4_	PhMe	120	58
12	Rh_2_(OAc)_4_	PhMe	40	no reaction

a 0.2 mmol **4a**, 1.1 equiv of **16a**, 0.03 M, sealed vial, yield by internal standard ^1^H NMR.

It was anticipated that the Lewis acidic nature of the Rh(II) carboxylate might also promote cyclodehydration of the 1,2-diaminoalkene to form the corresponding pyrrole, but this transformation was not detected. Therefore, the heterocycle forming process was evaluated in a separate^23d,26b,34^ operation (Table 2).

**Table 2:**
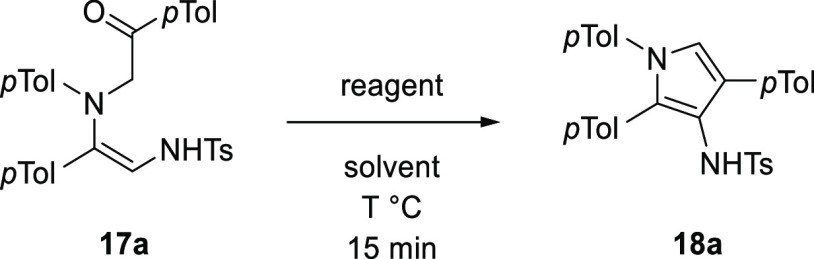
Optimization of Cyclodehydration^a^

entry	reagent (equiv)	solvent	*T* (°C)	yield (%)
1	BF_3_·OEt_2_ (0.5)	CH_2_Cl_2_	80	49
2	BF_3_·OEt_2_ (1.0)	CH_2_Cl_2_	80	66
**3**	**BF**_**3**_**·OEt**_**2**_ **(3.0)**	**CH**_**2**_**Cl**_**2**_	**80**	**72**
4	BF_3_·OEt_2_ (3.0)	(CH_2_Cl)_2_	80	65
5	BF_3_·OEt_2_ (3.0)	PhMe	80	19
6	BF_3_·OEt_2_ (3.0)	MeCN	80	38
7	BF_3_·OEt_2_ (3.0)	CH_2_Cl_2_	60	32
8	BF_3_·OEt_2_ (3.0)	CH_2_Cl_2_	40	32

a 0.2 mmol **17a**, 0.03 M, sealed vial, yield by internal standard ^1^H NMR.

Treatment of the 1,2-diaminoalkene **17a** with a selection of dehydrating/acidic reagents; including *p*-toluene sulfonic acid, trimethylsilyl triflate, acetic acid, and phosphorus oxychloride resulted in the decomposition of substrate with only a minimal amount of the desired pyrrole **18a**. Gratifyingly, using BF_3_·OEt_2_^35^ (0.5 equiv) in dichloromethane at 80 °C gave a modest 49% yield of pyrrole **18a** (Table 2, entry 1) with a cleaner reaction profile (^1^H NMR). Increasing the amount of Lewis acid from 0.5 to 3.0 equiv was accompanied by an increase in yield to 72% (entries 2 and 3). 1,2-Dichloroethane, toluene, and acetonitrile were considered as alternative solvents, but the yields were lower than those of dichloromethane, giving complex mixtures (entries 4–6). Reducing the temperature to less than 80 °C resulted in a more sluggish reaction (entries 7 and 8).

**Scheme 4:**
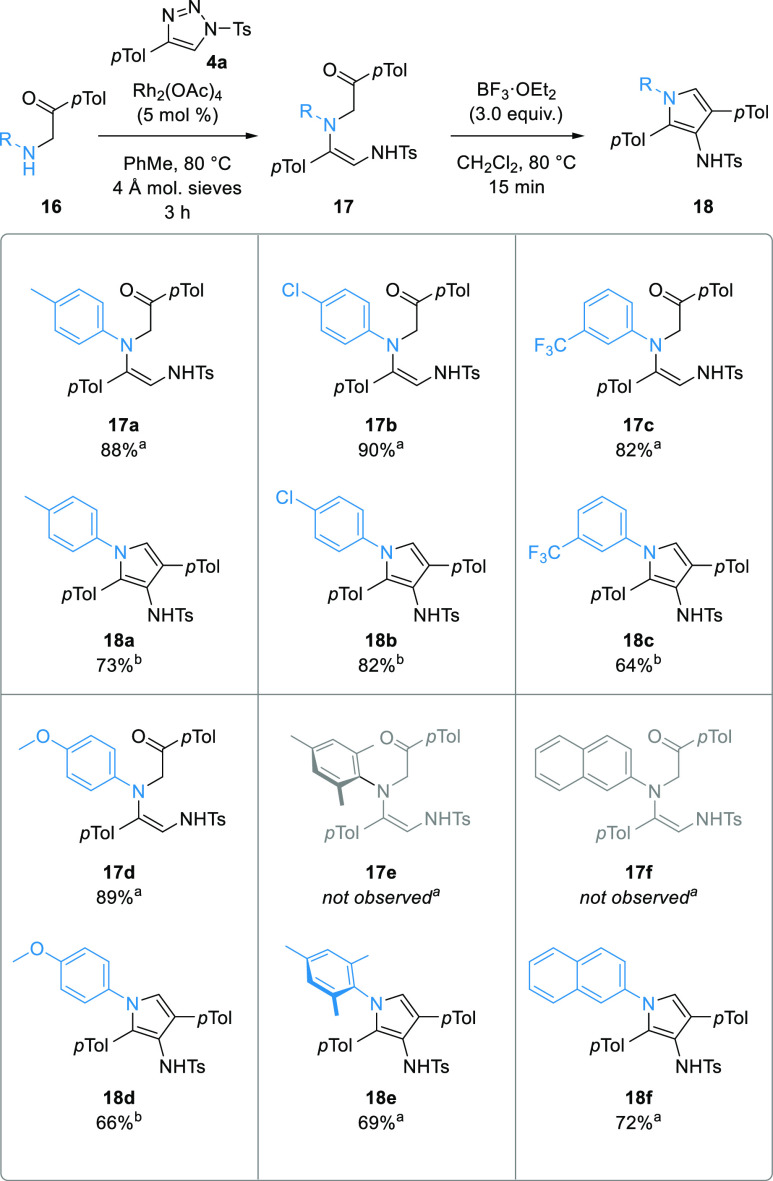
Scope of Arylamine in the Pyrrole Synthesis Isolated yield from Rh(II)-catalyzed N–H insertion. Isolated yield from BF_3_·OEt_2_-promoted cyclodehydration.

With the reaction conditions for a pyrrole synthesis sequence established, the generality of this process was explored (Scheme 4). Upon varying the *N*-aryl substituent, the Rh(II)-catalyzed N–H insertion and BF_3_·OEt_2_ promoted cyclodehydration were found to be highly tolerant with good to excellent yields obtained for both reactions (**17a**–**d** and **18a**–**d**). Surprisingly, when a mesityl group or naphthyl group was used, the Rh(II)-catalyzed reaction led to the pyrroles **18e**,**f** in good yield directly, and no 1,2-diamine product **17e**,**f** was observed. This difference in reactivity was attributed to promotion of the cyclization by raising the energy of unreactive conformations through steric interactions, placing the reacting centers in close proximity. Aliphatic amines were challenging substrates for the Rh(II)-catalyzed reaction and so were not compatible with this process, presumably owing to their increased basicity.

**Scheme 5:**
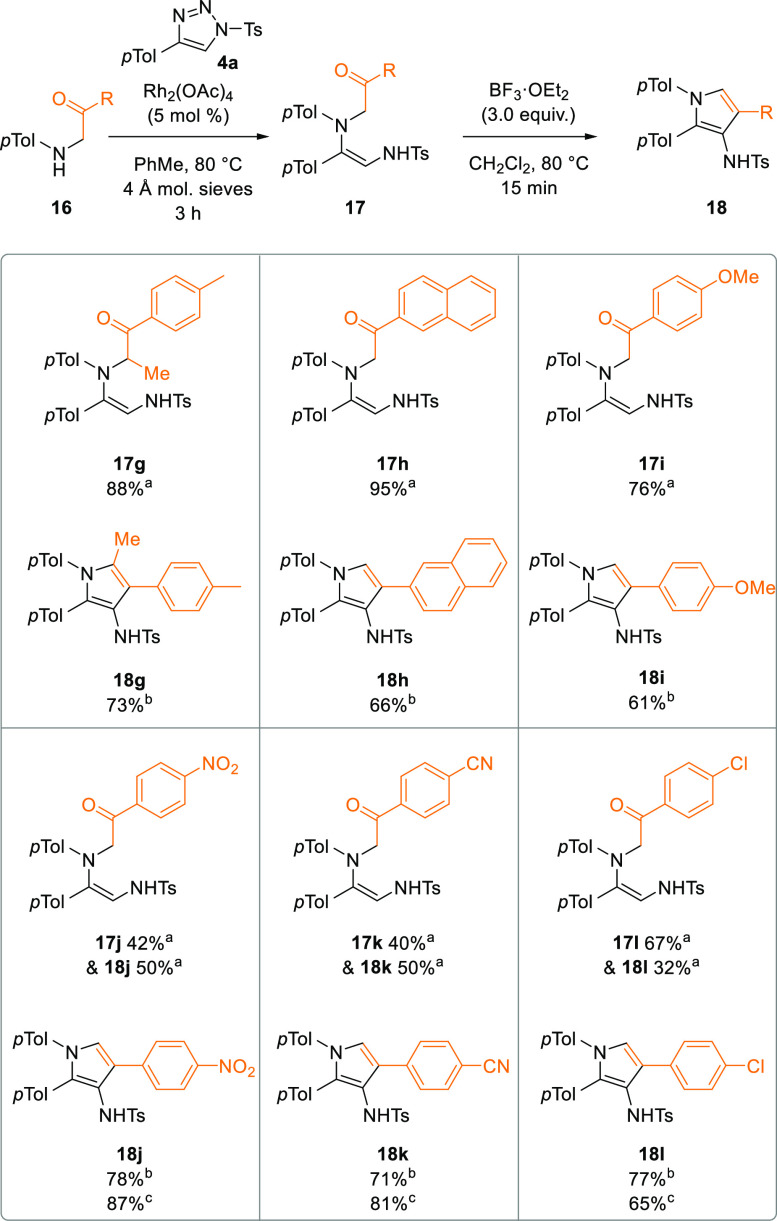
Scope of Ketone in the Pyrrole Synthesis Isolated yield from Rh(II)-catalyzed N–H insertion. Isolated yield from BF_3_·OEt_2_-promoted cyclodehydration. Isolated yield from Rh(II)-catalyzed N–H insertion with 16 h reaction time.

Variation of the ketone substituent was also studied, allowing control of the 4- and 5-positions of the resulting pyrrole (Scheme 5). Having an additional α-substituent in the substrate aminoketone was evaluated, and this proved to be compatible with the optimized conditions, giving a fully substituted pyrrole product **18g** in high yield over the two operations.

When more conjugated or electron-rich ketone substituents were studied, these also gave good yields for the two operations (**18h**,**i**). Interestingly, using more electron-withdrawing substituents NO_2_, CN, and Cl gave the expected N–H insertion products **17j**–**l** alongside the corresponding pyrroles **18j**–**l**, giving almost quantitative combined yields of the products. The two products were readily separated by column chromatography, and the 1,2-diaminoalkenes **17j**–**l** could be transformed into their corresponding pyrroles **18j**–**l** in good yield. Extending the reaction time in the presence of rhodium catalyst to 16 h led to formation of only the pyrrole products in high yield (87% of **18j**, 81% of **18k**, and 65% of **18l**). It is suggested that direct formation of pyrroles proceeded in these cases via the same products of N–H insertion, but that the more electron-withdrawing substituents activated the ketone toward nucleophilic attack, allowing cyclodehydration to occur with the more mildly Lewis acidic Rh(II) catalyst. This is consistent with the more electron-withdrawing NO_2_ and CN groups giving more pyrrole product than the Cl substituent.

**Scheme 6:**
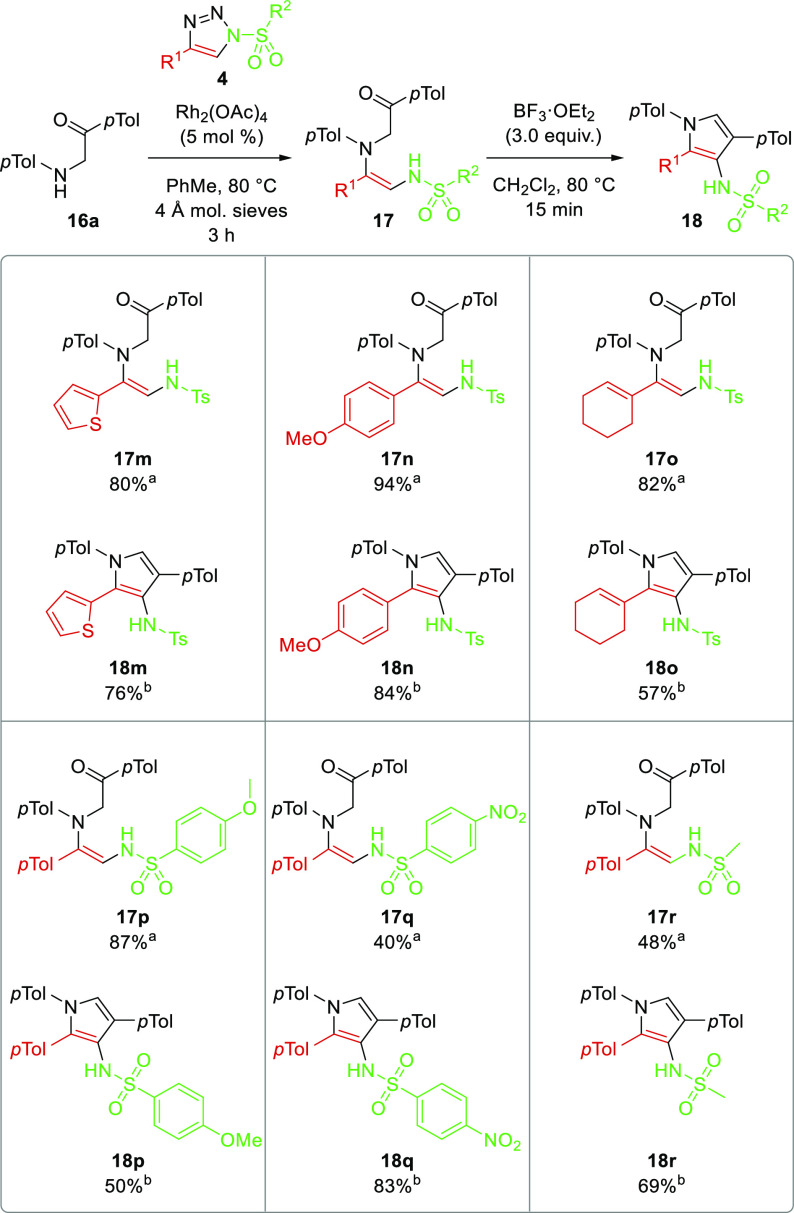
Scope of 1-ST in the Pyrrole Synthesis Isolated yield from Rh(II)-catalyzed N–H insertion. Isolated yield from BF_3_·OEt_2_-promoted cyclodehydration.

Different substituents at the 4-position of 1-ST **4** were also studied (Scheme 6). Heteroaromatic, electron-rich aromatic, and alkenyl 1-ST 4-substituents gave excellent yields for N–H insertion (**17m**–**o**). Interestingly, the alkene substrate completed N–H insertion (**17o**), despite the known intramolecular rearrangement of alkenyl 1-STs to a different class of pyrrole.^20^ Pyrroles **18m**,**n** were formed from these products using BF_3_·OEt_2_, again all with good to excellent yield. Variation of the sulfonyl group was also examined. Use of an electron-donating *p*-MeOC_6_H_4_SO_2_ group gave an excellent yield for N–H insertion (**17p**) but a moderate yield for cyclization to pyrrole **18p**. On the other hand, the electron-withdrawing *p*-NO_2_C_6_H_4_SO_2_ group and the small mesyl group gave a moderate yield for N–H insertion for **17q**,**r** but an excellent yield for cyclodehydration to the pyrrole **18q**,**r**.

**Scheme 7:**
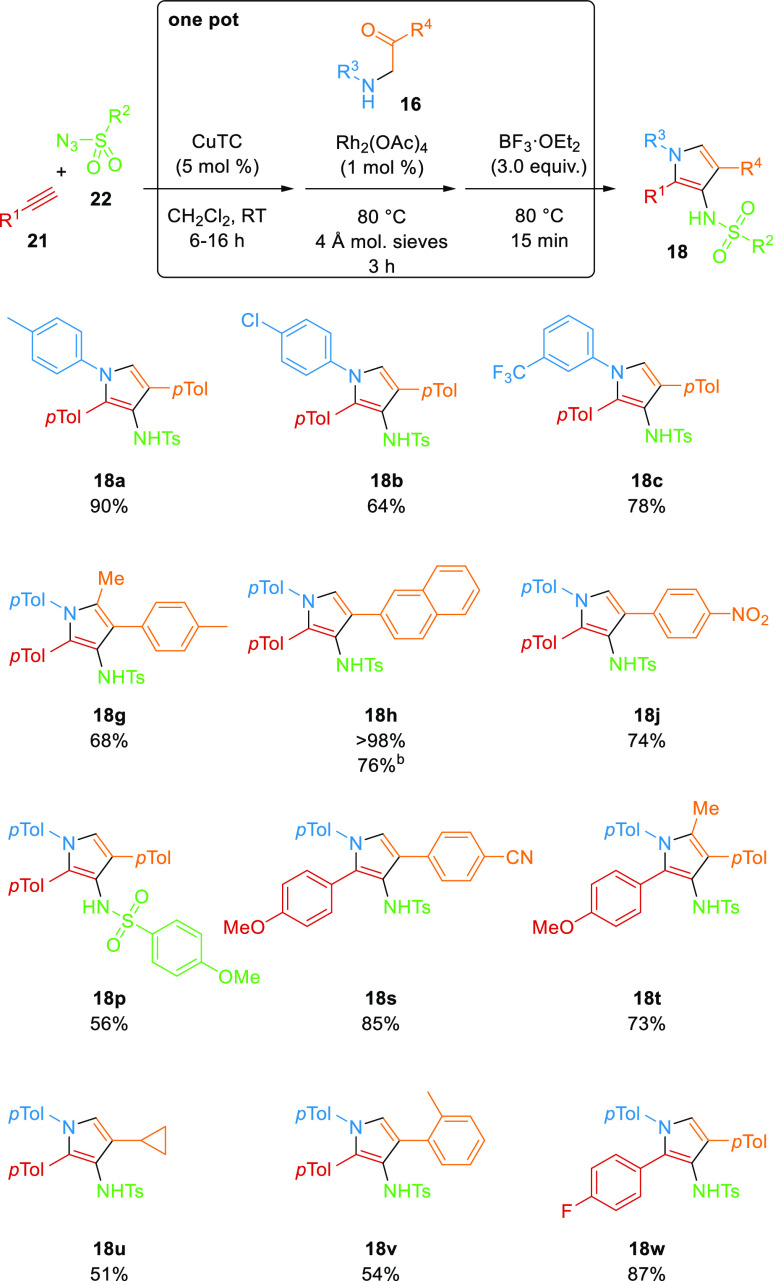
Three-Step, One-Pot Synthesis of Pyrroles **18** from Alkynes **21**, Sulfonyl Azides **22**, and α-Aminoketones **16** Isolated yield. On 4.6 mmol scale, using (CH_2_Cl)_2_ in place of CH_2_Cl_2_, isolated yield.

The key contributors to inefficiency in synthesis often come not from the reactions themselves but in the purification of intermediates, so combining or telescoping multiple operations in one pot or domino sequences is an excellent way to boost efficiency.^36^ CuAAC is highly versatile and has been incorporated in one-pot sequences with rhodium(II) reactions of 1-STs since the earliest work in the field,^18c,20,37^ so this was also investigated here (Scheme 7). Although, each of the sequential operations (CuAAC, N–H insertion, and cyclodehydration) had reagents and catalysts that should be tolerant to the previous conditions in the sequence, their solvent varied. Dichloromethane was selected for the one-pot process because it was significantly better than other solvents in the cyclodehydration step and worked well with the other steps, and the amount was selected to match the concentration of the most sensitive Rh(II)-catalyzed step. Treatment of *p-*tolylacetylene with tosyl azide in the presence of CuTC in CH_2_Cl_2_ gave complete conversion to the corresponding 1-ST **4a**. Without isolation, α-aminoketone **16a** and Rh_2_(OAc)_4_ were added to the mixture, and the vial was sealed and heated for 3 h at 80 °C. Then BF_3_·OEt_2_ (3.0 equiv) was added, and the reaction mixture was stirred for a further 15 min at 80 °C. Standard aqueous workup (NaHCO_3_) and filtration through silica gel afforded the pyrrole **18a** in 90% yield. This represented a marked improvement over the three separate steps (90 vs 58%) as well as making the overall process much more time- and material-efficient. Furthermore, the amount of copper and rhodium catalysts used was decreased to 5 and 1 mol %, respectively, without any deleterious effect. The one-pot process was applied to a range of terminal alkynes **21**, sulfonyl azides **22**, and amines **16**, all of which gave excellent yields of the corresponding pyrroles **18** with considerable improvement over the stepwise equivalent. The one-pot procedure was also completed on a larger scale (4.61 mmol), using (CH_2_Cl)_2_ in place of CH_2_Cl_2_ and under reflux instead of in a sealed vial, and the protocol delivered the product **18h** in excellent 76% yield.

Finally, the cleavage of the *N*-sulfonyl bond was demonstrated (eq 1). The *N*-tosyl pyrrole **18a** was treated with triflic acid at 90 °C, followed by workup with ethylene diamine,^38^ to reveal the pyrrole with a NH_2_ group **23a** in 77% yield.
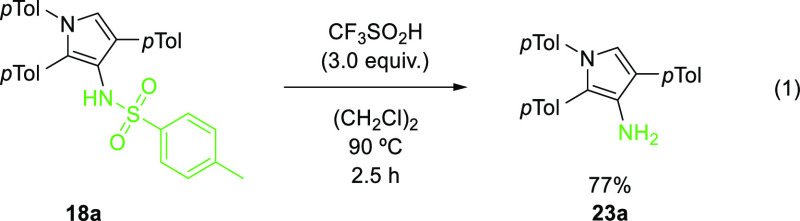
1

## Conclusion

A procedure has been developed that allows rapid assembly of 3-azapyrroles from readily available starting materials. The 1-ST and secondary amine starting materials were accessed in high yield from readily available building blocks. Rhodium(II) acetate was the optimum catalyst to promote 1-ST denitrogenation and insertion into the N–H bond of a range of α-aminoketones to form 1,2-aminoalkenes in high yields. For bulky arylamines and electron-deficient ketones, the 1,2-aminoalkenes cyclized into the corresponding 3-azapyrroles under rhodium catalysis, but for most examples, this was not the case and BF_3_·OEt_2_ proved to be a reliable Lewis acid for promoting the transformation into the heterocyclic products. These steps could be conducted individually or telescoped into a one-pot process beginning from sulfonyl azides, alkynes, and ketones, exploiting the orthogonal reactivity in the sequence. The availability of individual substrates means that this method represents a highly modular approach to pyrroles, allowing each of the five substituents to be customized through judicious choice of starting materials.

## Experimental Details

**CAUTION:** Nitrogen-rich compounds, such as azides and triazoles, can decompose violently with the loss of nitrogen gas. Although no problems were encountered in this study, appropriate precautions should be taken.

### General Information and Methods

NMR spectra were recorded on 400 and 500 MHz Bruker spectrometers. Chemical shifts are given in parts per million, and the spectra are calibrated to the residual ^1^H and ^13^C signals of the solvents. ^13^C NMR spectra were collected with complete proton decoupling, and assignments were made using COSY, HSQC, HMBC, and NOESY experiments. Samples were melted directly from the procedures described. High-resolution mass spectra were obtained on Agilent 6546 LC/Q-TOF and Bruker microTOFq instruments by Analytical Services at the University of Glasgow School of Chemistry. IR spectra were recorded using spectrometers fitted with an ATR device. CH_2_Cl_2_ and toluene were purified on a PureSolv PM500, and other reagents were used as received. Reactions were monitored by thin layer chromatography (TLC) using Merck TLC silica gel 60 F254 aluminum-foil baked plates. Compounds were visualized by UV light at nanometer resolution or by staining with potassium permanganate. Column chromatography was performed using a Teledyne ISCO Combiflash Rf+ system using Redisep Rf silica cartridges.

### General Procedure 1: CuAAC to Form 1STs

Copper(I) thiophene-2-carboxylate (10 mol %) and alkyne **21** (1.1 equiv) were dissolved in PhMe (0.1 M) and cooled to 0 °C (ice bath). After 10 min, sulfonyl azide **22** (1.0 equiv) was added in one portion and the reaction mixture was allowed to reach ambient temperature. When the reaction was complete (TLC, 1–3 h), the mixture was diluted with saturated aqueous NH_4_Cl and extracted with ethyl acetate (3 × 10 mL mmol^–1^). The combined organic layers were washed with brine, dried (MgSO_4_), filtered, and concentrated in vacuo. The residue was purified by flash column chromatography (SiO_2_, rapid gradient of 10–30% ethyl acetate in petroleum ether) to give the 1-ST **4**.

#### 4-(4-Tolyl)-1-tosyl-1,2,3-triazole (**4a**)

4-Ethynyltoluene (547 μL, 4.3 mmol, 1.1 equiv) and 4-toluenesulfonyl azide (809 mg, 4.1 mmol, 1.0 equiv) were treated with CuTC (69 mg, 0.36 mmol, 9 mol %) in PhMe (40 mL) according to General Procedure 1 to give the title compound **4a** (1.17 g, 91%) as a white solid: mp 127 °C dec (lit. 158–159 °C);^39^ ν_max_ (film) 3150, 2940, 1593, 1497, 1392, 1194, and 1179 cm^–1^; ^1^H NMR (400 MHz, 25.0 °C, CDCl_3_) δ 8.26 (1 H, s, triazole CH), 8.02 (2 H, d, *J* = 8.4 Hz, Ar), 7.71 (2 H, d, *J* = 8.4 Hz, Ar), 7.39 (2 H, d, *J* = 7.8 Hz, Ar), 7.23 (2 H, d, *J* = 7.8 Hz, Ar), 2.45 (3 H, s, CH_3_) and 2.38 (3 H, s, CH_3_); ^13^C{^1^H} NMR (101 MHz, 25.0 °C, CDCl_3_) δ 147.4 (Ar), 147.3 (triazole C4), 139.1 (Ar), 133.2 (Ar), 130.4 (2 × ArH), 129.7 (2 × ArH), 128.7 (2 × ArH), 126.0 (Ar), 126.0 (2 × ArH), 118.5 (triazole C5), 21.8 (CH_3_) and 21.3 (CH_3_); HRMS (ESI-TOF) *m*/*z* [M + Na]^+^ calcd for C_16_H_15_N_3_NaO_2_S^+^ 336.0777; found 336.0767. Recorded data are consistent with previous values.^39^

#### 4-(Thiophen-2-yl)-1-tosyl-1,2,3-triazole (**4m**)

2-Ethynylthiophene (438 μL, 4.6 mmol, 1.1 equiv) and 4-toluenesulfonyl azide (828 mg, 4.2 mmol, 1.0 equiv) were treated with CuTC (80 mg, 0.42 mmol, 10 mol %) in PhMe (42 mL) according to General Procedure 1 to give the title compound **4m** (1.24 g, 97%) as a white solid: mp 87 °C dec (lit. 140–141 °C);^18f^ ν_max_ (film) 3136, 2924, 1593, 1393, 1346, 1196, 1177, and 1092 cm^–1^; ^1^H NMR (400 MHz, 25.0 °C, CDCl_3_) δ 8.20 (1 H, s, triazole CH), 8.02 (2 H, d, *J* = 8.5 Hz, Ar), 7.44 (1 H, dd, *J* = 3.6, 1.2 Hz, thiophene CH), 7.39 (2 H, d, *J* = 8.5 Hz, Ar), 7.34 (1 H, dd, *J* = 5.1, 1.2 Hz, thiophene CH), 7.08 (1 H, dd, *J* = 5.1, 3.6 Hz, thiophene CH) and 2.45 (3 H, s, CH_3_); ^13^C{^1^H} NMR (101 MHz, 25.0 °C, CDCl_3_) δ 147.4 (triazole C4), 142.5 (thiophene C2), 133.0 (Ar), 130.8 (Ar), 130.5 (2 × ArH), 128.7 (2 × ArH), 127.8 (thiophene CH), 126.3 (thiophene CH), 125.6 (thiophene CH), 118.1 (triazole C5) and 21.8 (CH_3_); HRMS (ESI-TOF) *m*/*z* [M + Na]^+^ calcd for C_13_H_11_N_3_NaO_2_S_2_^+^ 328.0185; found 328.0184. Recorded data are consistent with previous values.^18f^

#### 4-(4-Methoxyphenyl)-1-tosyl-1,2,3-triazole (**4n**)

1-Ethynyl-4-methoxybenzene (535 μL, 4.1 mmol, 1.1 equiv) and 4-toluenesulfonyl azide (740 mg, 3.8 mmol, 1.0 equiv) were treated with CuTC (71 mg, 0.37 mmol, 10 mol %) in PhMe (40 mL) according to General Procedure 1 to give the title compound **4n** (1.05 g, 85%) as a white solid: mp 96 °C dec (lit. 100–101 °C);^40^ ν_max_ (film) 3144, 2932, 2839, 1616, 1497, 1393, 1331, 1250, 1177, and 1088 cm^–1^; ^1^H NMR (400 MHz, 25.0 °C, CDCl_3_) δ 8.22 (1 H, s, triazole CH), 8.01 (2 H, d, *J* = 8.5 Hz, Ar), 7.74 (2 H, d, *J* = 8.9 Hz, Ar), 7.37 (2 H, d, *J* = 8.5 Hz, Ar), 6.95 (2 H, d, *J* = 8.9 Hz, Ar), 3.83 (3 H, s, OCH_3_) and 2.43 (3 H, s, CH_3_); ^13^C{^1^H} NMR (101 MHz, 25.0 °C, CDCl_3_) δ 160.2 (Ar), 147.3 (Ar), 147.2 (triazole C4), 133.1 (Ar), 130.4 (2 × ArH), 128.6 (2 × ArH), 127.4 (2 × ArH), 121.4 (Ar), 117.9 (triazole C5), 114.4 (2 × ArH), 55.3 (OCH_3_) and 21.8 (CH_3_); HRMS (ESI-TOF) *m*/*z* [M + H]^+^ calcd for C_16_H_16_N_3_O_3_S^+^ 330.0907; found 330.0909. Recorded data are consistent with previous values.^40^

#### 4-(Cyclohexen-1-yl)-1-tosyl-1,2,3-triazole (**4o**)

1-Ethynylcyclohex-1-ene (485 μL, 4.1 mmol, 1.1 equiv) and 4-toluenesulfonyl azide (740 mg, 3.8 mmol, 1.0 equiv) were treated with CuTC (71 mg, 0.37 mmol, 10 mol %) in PhMe (40 mL) according to General Procedure 1 to give the title compound **4o** (950 mg, 83%) as a white solid: mp 100 °C dec (lit. 102–103 °C);^39^ ν_max_ (film) 3148, 2928, 2859, 1593, 1389, 1177, and 1092 cm^–1^; ^1^H NMR (400 MHz, 25.0 °C, CDCl_3_) δ 7.97 (2 H, d, *J* = 8.5 Hz, Ar), 7.88 (1 H, s, triazole CH), 7.36 (2 H, d, *J* = 8.5 Hz, Ar), 6.66 (1 H, tt, *J* = 3.9, 1.8 Hz, =CH), 2.43 (3 H, s, CH_3_), 2.34–2.27 (2 H, m, CH_2_), 2.22–2.15 (2 H, m, CH_2_), 1.78–1.71 (2 H, m, CH_2_) and 1.70–1.61 (2 H, m, CH_2_); ^13^C{^1^H} NMR (101 MHz, 25.0 °C, CDCl_3_) δ 148.9 (triazole C4), 147.0 (Ar), 133.3 (Ar), 130.3 (2 × ArH), 128.5 (2 × ArH), 127.7 (=CH), 125.8 (=C), 117.3 (triazole C5), 26.2 (CH_2_), 25.3 (CH_2_), 22.2 (CH_2_), 22.0 (CH_2_) and 21.8 (CH_3_); HRMS (ESI-TOF) *m*/*z* [M + H]^+^ calcd for C_15_H_18_N_3_O_2_S^+^ 304.1114; found 304.1113. Recorded data are consistent with previous values.^39^

#### 1-Methanesulfonyl-4-(4-tolyl)-1,2,3-triazole (**4p**)

4-Ethynyltoluene (558 μL, 4.4 mmol, 1.1 equiv) and methanesulfonyl azide (484 mg, 4.0 mmol, 1.0 equiv) were treated with CuTC (76 mg, 0.40 mmol, 10 mol %) in PhMe (40 mL) according to General Procedure 1 to give the title compound **4p** (898 mg, 95%) as a white solid: mp 95 °C dec (lit. 120–122 °C dec.);^41^ ν_max_ (film) 3140, 3028, 2924, 1381, 1231, 1184, 1161, and 1038 cm^–1^; ^1^H NMR (500 MHz, 25.0 °C, CDCl_3_) δ 8.26 (1 H, s, triazole CH), 7.76 (2 H, d, *J* = 8.0 Hz, Ar), 7.28 (2 H, d, *J* = 8.0 Hz, Ar), 3.56 (3 H, s, CH_3_) and 2.41 (3 H, s, CH_3_); ^13^C{^1^H} NMR (126 MHz, 25.0 °C, CDCl_3_) δ 147.6 (triazole C4), 139.4 (Ar), 129.8 (2 × ArH), 126.1 (2 × ArH), 125.8 (Ar), 118.4 (triazole C5), 42.7 (CH_3_) and 21.4 (CH_3_); HRMS (ESI-TOF) *m*/*z* [M + H]^+^ calcd for C_10_H_12_N_3_O_2_S^+^ 238.0645; found 238.0646. Recorded data are consistent with previous values.^41^

#### 1-(4-Nitrobenzenesulfonyl)-4-(4-tolyl)-1,2,3-triazole (**4q**)

4-Ethynyltoluene (533 μL, 4.2 mmol, 1.1 equiv) and 4-nitrobenzenesulfonyl azide (913 mg, 4.0 mmol, 1.0 equiv) were treated with CuTC (76 mg, 0.40 mmol, 10 mol %) in PhMe (40 mL) according to General Procedure 1 to give the title compound **4q** (1.20 g, 87%) as a white solid: mp 106 °C dec; ν_max_ (film) 3140, 3109, 1532, 1408, 1397, 1346, and 1188 cm^–1^; ^1^H NMR (500 MHz, 25.0 °C, CDCl_3_) δ 8.44 (2 H, d, *J* = 9.0 Hz, Ar), 8.36 (2 H, d, *J* = 9.0 Hz, Ar), 8.30 (1 H, s, triazole CH), 7.71 (2 H, d, *J* = 8.2 Hz, Ar), 7.25 (2 H, d, *J* = 8.2 Hz, Ar) and 2.39 (3 H, s, CH_3_); ^13^C{^1^H} NMR (126 MHz, 25.0 °C, CDCl_3_) δ 151.6 (Ar), 148.0 (triazole C4), 141.6 (Ar), 139.6 (Ar), 130.1 (2 × ArH), 129.8 (2 × ArH), 126.1 (2 × ArH), 125.4 (Ar), 125.0 (2 × ArH), 118.6 (triazole C5) and 21.4 (CH_3_); HRMS (ESI-TOF) *m*/*z* [M(hydrolyzed triazole) + H]^+^ calcd for C_9_H_10_N_3_^+^ 160.0869; found 160.0875.

#### 1-(4-Methoxylphenylsulfonyl)-4-(4-tolyl)-1,2,3-triazole (**4r**)

4-Ethynyltoluene (279 μL, 2.2 mmol, 1.1 equiv) and 4-methoxybenzenesulfonyl azide (426 mg, 2.0 mmol, 1.0 equiv) were treated with CuTC (38 mg, 0.20 mmol, 10 mol %) in PhMe (20 mL) according to General Procedure 1 to give the title compound **4r** (372 mg, 56%) as a white solid: mp 102 °C dec; ν_max_ (film) 2940, 2923, 1593, 1577, 1497, 1391, 1233, and 1168 cm^–1^; ^1^H NMR (500 MHz, 25.0 °C, CDCl_3_) δ 8.26 (1 H, s, triazole CH), 8.08 (2 H, d, *J* = 9.1 Hz, Ar), 7.71 (2 H, d, *J* = 8.1 Hz, Ar), 7.24 (2 H, d, *J* = 8.1 Hz, Ar), 7.03 (2 H, d, *J* = 9.1 Hz, Ar), 3.89 (3 H, s, OCH_3_) and 2.38 (3 H, s, CH_3_); ^13^C{^1^H} NMR (101 MHz, 25.0 °C, CDCl_3_) δ 12707.0 (Ar), 165.3 (Ar), 147.4 (triazole C4), 139.1 (Ar), 131.2 (2 × ArH), 129.7 (2 × ArH), 127.9 (Ar), 126.0 (2 × ArH), 118.4 (triazole C5), 115.1 (2 × ArH), 55.9 (OCH_3_) and 21.3 (CH_3_); HRMS (ESI-TOF) *m*/*z* [M + Na]^+^ calcd for C_16_H_15_N_3_NaO_3_S^+^ 352.0726; found 352.0719. Recorded data are consistent with previous values.^42^

### General Procedure 2: Formation of α-Aminoketones

Amine **19** (1.0 equiv) was dissolved in a 1:1 mixture of water and ethanol (0.2 M). NaHCO_3_ (1.0 equiv) was added followed by the α-bromoketone **20** (1.0 equiv), and the reaction mixture was stirred until completion (TLC, 2–24 h). The reaction mixture was diluted with ethyl acetate, and the aqueous phase was extracted with ethyl acetate (3 × 5 mL mmol^–1^). The combined organic layers were washed with brine, dried (MgSO_4_), and concentrated in vacuo. This product was either purified by recrystallization from CHCl_3_/pentane or by column chromatography (SiO_2_, gradient of 10–30% EtOAc in petroleum ether).

#### 1-(4-Tolyl)-2-(4-tolylamino)ethan-1-one (**16a**)

*p*-Toluidine (1.00 g, 9.4 mmol, 1.0 equiv) and 2-bromo-4′-methylacetophenone (2.00 g, 9.4 mmol, 1.0 equiv) were treated with sodium hydrogen carbonate (789 mg, 9.4 mmol, 1.0 equiv) in ethanol (30 mL) and water (30 mL) according to General Procedure 2 for 16 h, and then the crude product was purified by recrystallization (CHCl_3_/pentane) to give the title compound **16a** (1.93 g, 86%) as a yellow solid: mp 130–138 °C (lit. 130–133 °C);^43^ ν_max_ (film) 3405, 3029, 1682, 1612, 1527, and 1356 cm^–1^; ^1^H NMR (500 MHz, 22.2 °C, CDCl_3_) δ 7.92 (2 H, d, *J* = 8.5 Hz, Ar), 7.31 (2 H, d, *J* = 7.8 Hz, Ar), 7.04 (2 H, d, *J* = 7.8 Hz, Ar), 6.65 (2 H, d, *J* = 8.5 Hz, Ar), 4.85 (1 H, br s, NH), 4.58 (2 H, s, CH_2_), 2.44 (3 H, s, CH_3_) and 2.26 (3 H, s, CH_3_); ^13^C{^1^H} NMR (126 MHz, 22.6 °C, CDCl_3_) δ 194.9 (C=O), 144.9 (Ar), 144.8 (Ar), 132.5 (Ar), 129.8 (2 × ArH), 129.5 (2 × ArH), 127.8 (2 × ArH), 127.0 (Ar), 113.2 (2 × ArH), 50.6 (CH_2_), 21.8 (CH_3_) and 20.4 (CH_3_); HRMS (ESI-TOF) *m*/*z* [M + Na]^+^ calcd for C_16_H_17_NNaO^+^ 262.1202; found 262.1204.

#### 2-(4-Chlorophenylamino)-1-(4-tolyl)ethan-1-one (**16b**)

4-Chloroaniline (255 mg, 2.0 mmol, 1.0 equiv) and 2-bromo-4′-methylacetophenone (426 mg, 2.0 mmol, 1.0 equiv) were treated with sodium hydrogen carbonate (168 mg, 2.0 mmol, 1.0 equiv) in ethanol (5 mL) and water (5 mL) according to General Procedure 2 for 24 h, and then the crude product was purified by recrystallization (CHCl_3_/pentane) to give the title compound **16b** (438 mg, 84%) as a white solid: mp 125 °C dec (lit. 167–169 °C);^44^ ν_max_ (film) 3393, 3030, 1675, 1603, 1499, 1353, 1188, and 1092 cm^–1^; ^1^H NMR (400 MHz, 22.4 °C, CDCl_3_) δ 7.90 (2 H, d, *J* = 8.2 Hz, Ar), 7.31 (2 H, d, *J* = 8.2 Hz, Ar), 7.16 (2 H, d, *J* = 8.8 Hz, Ar), 6.64 (2 H, d, *J* = 8.8 Hz, Ar), 4.54 (2 H, s, CH_2_) and 2.44 (3 H, s, CH_3_); ^13^C{^1^H} NMR (101 MHz, 22.8 °C, CDCl_3_) δ 194.2 (C=O), 145.6 (Ar), 145.0 (Ar), 132.2 (Ar), 129.6 (2 × ArH), 129.2 (2 × ArH), 127.8 (2 × ArH), 122.4 (Ar), 114.1 (2 × ArH), 50.1 (CH_2_) and 21.7 (CH_3_); HRMS (ESI-TOF) *m*/*z* [M + Na]^+^ calcd for C_15_H_14_ClNNaO^+^ 282.0656; found 282.0655.

#### 1-(4-Tolyl)-2-(3-trifluoromethylphenylamino)ethan-1-one (**16c**)

3-Trifluoromethylaniline (323 mg, 2.0 mmol, 1.0 equiv) and 2-bromo-4′-methylacetophenone (426 mg, 2.0 mmol, 1.0 equiv) were treated with sodium hydrogen carbonate (168 mg, 2.0 mmol, 1.0 equiv) in ethanol (5 mL) and water (5 mL) according to General Procedure 2 for 16 h, and then the crude product was purified by recrystallization (CHCl_3_/pentane) to give the title compound **16c** (343 mg, 58%) as a white solid: mp 133–135 °C; ν_max_ (film) 3402, 2928, 1678, 1605, 1501, 1362, 1339, 1254, 1161, and 1115 cm^–1^; ^1^H NMR (400 MHz, 25.0 °C, CDCl_3_) δ 7.94 (2 H, d, *J* = 8.2 Hz, Ar), 7.33 (2 H, d, *J* = 8.2 Hz, Ar), 7.32–7.29 (1 H, m, Ar), 7.01–6.96 (1 H, m, Ar), 6.90–6.84 (2 H, m, Ar), 5.22 (1 H, br s, NH), 4.60 (2 H, s, CH_2_) and 2.45 (3 H, s, CH_3_); ^13^C{^1^H} NMR (101 MHz, 25.0 °C, CDCl_3_) δ 193.9 (C=O), 147.2 (Ar), 145.1 (Ar), 132.2 (Ar), 131.7 (q, *J* = 31.9 Hz, ArH), 129.7 (ArH), 129.6 (2 × ArH), 127.9 (2 × ArH), 124.3 (q, *J* = 272.3 Hz, CF_3_), 116.3 (ArH), 114.1 (q, *J* = 4.1 Hz, ArH), 108.7 (q, *J* = 4.0 Hz, ArH), 49.7 (CH_2_) and 21.8 (CH_3_); HRMS (ESI-TOF) *m*/*z* [M + H]^+^ calcd for C_16_H_15_F_3_NO^+^ 294.1100; found 294.1110.

#### 2-(4-Methoxyphenylamino)-1-(4-tolyl)ethan-1-one (**16d**)

*p*-Anisidine (246 mg, 2.0 mmol, 1.0 equiv) and 2-bromo-4′-methylacetophenone (426 mg, 2.0 mmol, 1.0 equiv) were treated with sodium hydrogen carbonate (168 mg, 2.0 mmol, 1.0 equiv) in ethanol (5 mL) and water (5 mL) according to General Procedure 2 for 24 h, and then the crude product was purified by recrystallization (CHCl_3_/pentane) to give the title compound **16d** (454 mg, 89%) as a yellow solid: mp 97–100 °C (lit. 102–103 °C);^43^ ν_max_ (film) 3395, 3007, 2930, 2832, 1675, 1607, 1515, 1236, 1182, and 1037 cm^–1^; ^1^H NMR (400 MHz, 22.4 °C, CDCl_3_) δ 7.91 (2 H, d, *J* = 8.2 Hz, Ar), 7.31 (2 H, d, *J* = 8.2 Hz, Ar), 6.83 (2 H, d, *J* = 8.9 Hz, Ar), 6.68 (2 H, d, *J* = 8.9 Hz, Ar), 4.55 (2 H, s, CH_2_), 3.76 (3 H, s, OCH_3_) and 2.44 (3 H, s, CH_3_); ^13^C{^1^H} NMR (101 MHz, 23.0 °C, CDCl_3_) δ 195.0 (C=O), 152.3 (Ar), 144.7 (Ar), 141.6 (Ar), 132.5 (Ar), 129.5 (2 × ArH), 127.8 (2 × ArH), 115.0 (2 × ArH), 114.2 (2 × ArH), 55.8 (OCH_3_), 51.1 (CH_2_) and 21.7 (CH_3_); HRMS (ESI-TOF) *m*/*z* [M + H]^+^ calcd for C_16_H_18_NO_2_^+^ 256.1332; found 256.1330.

#### 1-(4-Tolyl)-2-(2,4,6-trimethylphenylamino)ethan-1-one (**16e**)

2,4,6-Trimethylaniline (271 mg, 2.0 mmol, 1.0 equiv) and 2-bromo-4′-methylacetophenone (426 mg, 2.0 mmol, 1.0 equiv) were treated with sodium hydrogen carbonate (168 mg, 2.0 mmol, 1.0 equiv) in ethanol (5 mL) and water (5 mL) according to General Procedure 2 for 16 h, and then the crude product was purified by recrystallization (CHCl_3_/pentane) to give the title compound **16e** (397 mg, 74%) as an off white solid: mp 123 °C dec; ν_max_ (film) 3028, 2974, 2924, 2870, 1686, 1609, and 1069 cm^–1^; ^1^H NMR (400 MHz, 25.0 °C, CDCl_3_) δ 7.84 (2 H, d, *J* = 7.8 Hz, Ar), 7.27 (2 H, d, *J* = 7.8 Hz, Ar), 6.84 (2 H, s, Ar), 4.54 (1 H, br s, NH), 4.48 (2 H, s, CH_2_), 2.42 (3 H, s, CH_3_), 2.35 (6 H, s, 2 × CH_3_) and 2.24 (3 H, s, CH_3_); ^13^C{^1^H} NMR (101 MHz, 25.0 °C, CDCl_3_) δ 196.4 (C=O), 144.6 (Ar), 143.9 (Ar), 132.5 (Ar), 131.1 (Ar), 129.5 (2 × ArH), 129.4 (2 × ArH), 129.0 (2 × Ar), 127.7 (2 × ArH), 55.3 (CH_2_), 21.7 (CH_3_), 20.5 (CH_3_) and 18.7 (2 × CH_3_); HRMS (ESI-TOF) *m*/*z* [M + H]^+^ calcd for C_18_H_22_NO^+^ 268.1696; found 268.1696.

#### 2-(Naphth-2-ylamino)-1-(4-tolyl)ethan-1-one (**16f**)

2-Naphthylamine (285 mg, 2.0 mmol, 1.0 equiv) and 2-bromo-4′-methylacetophenone (426 mg, 2.0 mmol, 1.0 equiv) were treated with sodium hydrogen carbonate (202 mg, 2.4 mmol, 1.2 equiv) in ethanol (5 mL) and water (5 mL) according to General Procedure 2 for 16 h, and then the crude product was purified by recrystallization (CHCl_3_/pentane) to give the title compound **16f** (422 mg, 77%) as a gray solid: mp 120–125 °C; ν_max_ (film) 3418, 3051, 2920, 1686, 1582, 1523, 1408, and 1235 cm^–1^; ^1^H NMR (400 MHz, 25.0 °C, CDCl_3_) δ 8.09–8.04 (1 H, m, naphthyl CH), 7.99 (2 H, d, *J* = 8.1 Hz, Ar), 7.84–7.79 (1 H, m, naphthyl CH), 7.54–7.46 (2 H, m, naphthyl CH), 7.38 (1 H, dd, *J* = 8.2, 7.5 Hz, naphthyl CH), 7.34 (2 H, d, *J* = 8.1 Hz, Ar), 7.29–7.26 (1 H, m, naphthyl CH), 6.61 (1 H, dd, *J* = 7.5, 1.1 Hz, naphthyl CH), 5.83 (1 H, br s, NH), 4.73 (2 H, s, CH_2_) and 2.46 (3 H, s, CH_3_); ^13^C{^1^H} NMR (101 MHz, 25.0 °C, CDCl_3_) δ 194.6 (C=O), 145.0 (naphthyl C), 142.4 (Ar), 134.4 (naphthyl C), 132.5 (naphthyl C), 129.7 (2 × ArH), 128.6 (naphthyl CH), 128.0 (2 × ArH), 126.5 (naphthyl CH), 126.0 (naphthyl CH), 124.9 (naphthyl CH), 123.4 (Ar), 120.3 (naphthyl CH), 117.7 (naphthyl CH), 104.4 (naphthyl CH), 50.2 (CH_2_) and 21.8 (CH_3_); HRMS (ESI-TOF) *m*/*z* [M + H]^+^ calcd for C_19_H_18_NO^+^ 276.1383; found 276.1389.

#### 1-(4-Tolyl)-2-(4-tolylamino)propan-1-one (**16g**)

NaH (60% suspension in mineral oil, 50 mg, 1.25 mmol, 1.1 equiv) was suspended in DMF (10 mL) and cooled to 0 °C (ice bath), and 1-(4-tolyl)-2-(4-tolylamino)ethan-1-one **16a** (338 mg, 1.14 mmol, 1.0 equiv) was added in three portions. The reaction was stirred for 30 min and then MeI (92 μL, 1.48 mmol, 1.3 equiv) was added, and the reaction stirred for a further 3 h. Water was added, and the aqueous phase was extracted with Et_2_O (3 × 15 mL). The combined organic layers were washed with water, brine, dried over MgSO_4_, filtered, and concentrated in vacuo. Purification by flash column chromatography (SiO_2_, gradient of 5–20% EtOAc in petroleum ether) gave the title compound as an orange oil (250 mg, 87%). Recorded data are consistent with previous values:^45^ ν_max_ (film) 3387, 2982, 2920, 1682, 1609, and 1520 cm^–1^; ^1^H NMR (400 MHz, 25.0 °C, CDCl_3_) δ 7.92 (2 H, d, *J* = 8.3 Hz, Ar), 7.30 (2 H, d, *J* = 7.9 Hz, Ar), 6.98 (2 H, d, *J* = 7.9 Hz, Ar), 6.60 (2 H, d, *J* = 8.3 Hz, Ar), 5.08 (1 H, q, *J* = 6.9 Hz, CH), 2.43 (3 H, s, CH_3_), 2.23 (3 H, s, CH_3_) and 1.46 (3 H, d, *J* = 6.9 Hz, CH_3_); ^13^C{^1^H} NMR (101 MHz, 25.0 °C, CDCl_3_) δ 200.5 (C=O), 144.5 (Ar), 144.3 (Ar), 132.2 (Ar), 129.8 (2 × ArH), 129.5 (2 × ArH), 128.6 (2 × ArH), 127.1 (Ar), 113.7 (2 × ArH), 53.6 (CH), 21.7 (CH_3_), 20.4 (CH_3_) and 19.7 (CH_3_); HRMS (ESI-TOF) *m*/*z* [M + H]^+^ calcd for C_17_H_20_NO^+^ 254.1539; found 254.1542.

#### 1-(Naphth-2-yl)-2-(4-tolylamino)ethan-1-one (**16h**)

*p*-Toluidine (429 mg, 4.0 mmol, 1.0 equiv) and 2-bromo-1-(naphthalen-2-yl)ethan-1-one (996 mg, 4.0 mmol, 1.0 equiv) were treated with sodium hydrogen carbonate (336 mg, 4.0 mmol, 1.0 equiv) in ethanol (10 mL) and water (10 mL) according to General Procedure 2 for 2 h, and then the crude product was purified by recrystallization (CHCl_3_/pentane) to give the title compound **16h** (980 mg, 89%) as a yellow solid: mp 130 °C dec; ν_max_ (film) 3395, 2970, 2924, 2862, 1682, 1620, 1528, 1057, and 1015 cm^–1^; ^1^H NMR (400 MHz, 22.0 °C, CDCl_3_) δ 8.55 (1 H, s, naphthyl CH), 8.07 (1 H, dd, *J* = 8.6, 1.8 Hz, naphthyl CH), 8.01 (1 H, dd, *J* = 8.0, 1.3 Hz, naphthyl CH), 7.95 (1 H, d, *J* = 8.6 Hz, naphthyl CH), 7.91 (1 H, d, *J* = 8.4 Hz, naphthyl CH), 7.64 (1 H, ddd, *J* = 8.2, 6.9, 1.5 Hz, naphthyl CH), 7.59 (1 H, ddd, *J* = 8.2, 6.9, 1.5 Hz, naphthyl CH), 7.07 (2 H, d, *J* = 8.6 Hz, Ar), 6.72 (2 H, d, *J* = 8.4 Hz, Ar), 5.05 (1 H, br s, NH), 4.76 (2 H, s, CH_2_) and 2.27 (3 H, s, CH_3_); ^13^C{^1^H} NMR (101 MHz, 22.7 °C, CDCl_3_) δ 195.2 (C=O), 144.8 (naphthyl C), 135.9 (Ar), 132.5 (naphthyl C), 132.3 (naphthyl C), 129.9 (2 × ArH), 129.6 (naphthyl CH), 129.4 (naphthyl CH), 128.8 (naphthyl CH), 128.8 (naphthyl CH), 127.9 (naphthyl CH), 127.2 (Ar), 127.1 (naphthyl CH), 123.4 (naphthyl CH), 113.3 (2 × ArH), 50.9 (CH_2_) and 20.4 (CH_3_); HRMS (ESI-TOF) *m*/*z* [M + H]^+^ calcd for C_19_H_18_NO^+^ 276.1383; found 276.1391.

#### 1-(4-Methoxyphenyl)-2-(4-tolylamino)ethan-1-one (**16i**)

*p*-Toluidine (429 mg, 4.0 mmol, 1.0 equiv) and 2-bromo-4′-methoxyacetophenone (916 mg, 4.0 mmol, 1.0 equiv) were treated with sodium hydrogen carbonate (336 mg, 4.0 mmol, 1.0 equiv) in ethanol (10 mL) and water (10 mL) according to General Procedure 2 for 2 h to give the title compound **16i** (888 mg, 87%) as a brown solid: mp 111–114 °C; ν_max_ (film) 3402, 3001, 2920, 2843, 1674, 1601, 1512, 1258, 1177, and 1034 cm^–1^; ^1^H NMR (400 MHz, 22.3 °C, CDCl_3_) δ 8.00 (2 H, d, *J* = 8.9 Hz, Ar), 7.04 (2 H, d, *J* = 8.3 Hz, Ar), 6.98 (2 H, d, *J* = 8.9 Hz, Ar), 6.66 (2 H, d, *J* = 8.3 Hz, Ar), 5.00 (1 H, br s, NH), 4.56 (2 H, s, CH_2_), 3.90 (3 H, s, OCH_3_) and 2.26 (3 H, s, CH_3_); ^13^C{^1^H} NMR (101 MHz, 23.0 °C, CDCl_3_) δ 193.6 (C=O), 164.0 (Ar), 144.9 (Ar), 130.1 (2 × ArH), 129.9 (2 × ArH), 128.0 (Ar), 127.1 (Ar), 114.0 (2 × ArH), 113.3 (2 × ArH), 55.5 (OCH_3_), 50.4 (CH_2_) and 20.4 (CH_3_); HRMS (ESI-TOF) *m*/*z* [M + H]^+^ calcd for C_16_H_18_NO_2_^+^ 256.1332; found 256.1333.

#### 1-(4-Nitrophenyl)-2-(4-tolylamino)ethan-1-one (**16j**)

*p*-Toluidine (429 mg, 4.0 mmol, 1.0 equiv) and 2-bromo-4′-nitroacetophenone (976 mg, 4.0 mmol, 1.0 equiv) were treated with sodium hydrogen carbonate (336 mg, 4.0 mmol, 1.0 equiv) in ethanol (10 mL) and water (10 mL) according to General Procedure 2 for 2 h, and then the crude product was purified by recrystallization (CHCl_3_/pentane) to give the title compound **16j** (971 mg, 90%) as a red solid: mp 127 °C dec (lit. 147–150 °C);^46^ ν_max_ (film) 3032, 2924, 2866, 1678, 1601, 1524, and 1346 cm^–1^; ^1^H NMR (400 MHz, 22.2 °C, CDCl_3_) δ 8.37 (2 H, d, *J* = 8.8 Hz, Ar), 8.18 (2 H, d, *J* = 8.8 Hz, Ar), 7.05 (2 H, d, *J* = 8.4 Hz, Ar), 6.65 (2 H, d, *J* = 8.4 Hz, Ar), 4.74 (1 H, br s, NH), 4.66 (2 H, s, CH_2_) and 2.26 (3 H, s, CH_3_); ^13^C{^1^H} NMR (101 MHz, 23.0 °C, CDCl_3_) δ 194.1 (C=O), 150.7 (Ar), 144.4 (Ar), 139.4 (Ar), 130.0 (2 × ArH), 128.9 (2 × ArH), 127.6 (Ar), 124.1 (2 × ArH), 113.2 (2 × ArH), 51.4 (CH_2_) and 20.4 (CH_3_); HRMS (ESI-TOF) *m*/*z* [M + H]^+^ calcd for C_15_H_15_N_2_O_3_^+^ 271.1077; found 271.1084.

#### 1-(4-Cyanophenyl)-2-(4-tolylamino)ethan-1-one (**16k**)

*p*-Toluidine (429 mg, 4.0 mmol, 1.0 equiv) and 2-bromo-4′-cyanoacetophenone (896 mg, 4.0 mmol, 1.0 equiv) were treated with sodium hydrogen carbonate (336 mg, 4.0 mmol, 1.0 equiv) in ethanol (10 mL) and water (10 mL) according to General Procedure 2 for 2 h to give the title compound **16k** (921 mg, 92%) as an orange solid: mp 126 °C dec; ν_max_ (film) 3322, 2924, 2866, 2230, 1678, 1605, 1516, 1404, and 1277 cm^–1^; ^1^H NMR (400 MHz, 22.1 °C, CDCl_3_) δ 8.11 (2 H, d, *J* = 8.5 Hz, Ar), 7.83 (2 H, d, *J* = 8.5 Hz, Ar), 7.04 (2 H, d, *J* = 8.4 Hz, Ar), 6.64 (2 H, d, *J* = 8.4 Hz, Ar), 4.62 (2 H, s, CH_2_) and 2.26 (3 H, s, CH_3_); ^13^C{^1^H} NMR (101 MHz, 23.0 °C, CDCl_3_) δ 194.3 (C=O), 144.4 (Ar), 137.9 (Ar), 132.7 (2 × ArH), 129.9 (2 × ArH), 128.2 (2 × ArH), 127.5 (Ar), 117.7 (Ar), 117.1 (C≡N), 113.2 (2 × ArH), 51.2 (CH_2_) and 20.4 (CH_3_); HRMS (ESI-TOF) *m*/*z* [M + H]^+^ calcd for C_16_H_15_N_2_O^+^ 251.1179; found 251.1178.

#### 1-(4-Chlorophenyl)-2-(4-tolylamino)ethan-1-one (**16l**)

*p*-Toluidine (429 mg, 4.0 mmol, 1.0 equiv) and 2-bromo-4′-chloroacetophenone (934 mg, 4.0 mmol, 1.0 equiv) were treated with sodium hydrogen carbonate (336 mg, 4.0 mmol, 1.0 equiv) in ethanol (10 mL) and water (10 mL) according to General Procedure 2 for 2 h, and then the crude product was purified by recrystallization (CHCl_3_/pentane) to give the title compound **16l** (866 mg, 83%) as a yellow solid: mp 127–130 °C (lit. 150–152 °C);^44^ ν_max_ (film) 3391, 2916, 2859, 1682, 1589, and 1524 cm^–1^; ^1^H NMR (400 MHz, 22.4 °C, CDCl_3_) δ 7.96 (2 H, d, *J* = 8.6 Hz, Ar), 7.49 (2 H, d, *J* = 8.6 Hz, Ar), 7.04 (2 H, d, *J* = 8.4 Hz, Ar), 6.64 (2 H, d, *J* = 8.4 Hz, Ar), 4.76 (1 H, br s, NH), 4.58 (2 H, s, CH_2_) and 2.26 (3 H, s, CH_3_); ^13^C{^1^H} NMR (101 MHz, 22.8 °C, CDCl_3_) δ 194.2 (C=O), 144.7 (Ar), 140.3 (Ar), 133.3 (Ar), 129.9 (2 × ArH), 129.2 (2 × ArH), 129.2 (2 × ArH), 127.2 (Ar), 113.2 (2 × ArH), 50.7 (CH_2_) and 20.4 (CH_3_); HRMS (ESI-TOF) *m*/*z* [M + H]^+^ calcd for C_15_H_15_ClNO^+^ 260.0837; found 260.0834.

#### 1-Cyclopropyl-2-(4-tolylamino)ethan-1-one (**16u**)

*p*-Toluidine (429 mg, 4.0 mmol, 1.0 equiv) and 2-bromo-1-cyclopropylethanone (652 mg, 4.0 mmol, 1.0 equiv) were treated with sodium hydrogen carbonate (336 mg, 4.0 mmol, 1.0 equiv) in ethanol (10 mL) and water (10 mL) according to General Procedure 2 for 16 h to give the title compound **16u** (562 mg, 74%) as a brown solid: mp 75–78 °C; ν_max_ (film) 3395, 3013, 2920, 2859, 1694, 1616, 1524, 1393, and 1069 cm^–1^; ^1^H NMR (400 MHz, 25.0 °C, CDCl_3_) δ 7.01 (2 H, d, *J* = 8.5 Hz, Ar), 6.54 (2 H, d, *J* = 8.5 Hz, Ar), 4.52 (1 H, br s, NH), 4.15 (2 H, s, CH_2_), 2.24 (3 H, s, CH_3_), 2.01 (1 H, tt, *J* = 7.8, 4.6 Hz, cyclopropane CH), 1.16–1.12 (2 H, m, cyclopropane CH_2_) and 1.01–0.95 (2 H, m, cyclopropane CH_2_); ^13^C{^1^H} NMR (101 MHz, 25.0 °C, CDCl_3_) δ 206.5 (C=O), 144.8 (Ar), 129.8 (2 × ArH), 126.9 (Ar), 113.0 (2 × ArH), 54.5 (CH_2_), 20.4 (CH_3_), 18.8 (cyclopropane CH) and 11.3 (2 × cyclopropane CH_2_); HRMS (ESI-TOF) *m*/*z* [M + H]^+^ calcd for C_12_H_16_NO^+^ 190.1226; found 190.1230.

#### 1-(2-Tolyl)-2-(4-tolylamino)ethan-1-one (**16v**)

*p*-Toluidine (429 mg, 4.0 mmol, 1.0 equiv) and 2-bromo-2′-methylacetophenone (852 mg, 4.0 mmol, 1.0 equiv) were treated with sodium hydrogen carbonate (336 mg, 4.0 mmol, 1.0 equiv) in ethanol (10 mL) and water (10 mL) according to General Procedure 2 for 16 h, and then the crude product was purified by recrystallization (CHCl_3_/pentane) to give the title compound **16v** (421 mg, 44%) as an orange solid: mp 124–127 °C; ν_max_ (film) 3387, 2924, 2851, 1690, 1620, and 1524 cm^–1^; ^1^H NMR (400 MHz, 25.0 °C, CDCl_3_) δ 7.74–7.71 (1 H, m, Ar), 7.46–7.42 (1 H, m, Ar), 7.34–7.29 (2 H, m, Ar), 7.03 (2 H, d, *J* = 8.3 Hz, Ar), 6.62 (2 H, d, *J* = 8.3 Hz, Ar), 4.48 (2 H, s, CH_2_), 2.55 (3 H, s, CH_3_) and 2.25 (3 H, s, CH_3_); ^13^C{^1^H} NMR (101 MHz, 25.0 °C, CDCl_3_) δ 198.8 (C=O), 144.9 (Ar), 138.9 (Ar), 135.4 (Ar), 132.3 (ArH), 132.1 (ArH), 129.9 (2 × ArH), 128.1 (ArH), 127.0 (Ar), 125.9 (ArH), 113.2 (2 × ArH), 52.6 (CH_2_), 21.4 (CH_3_) and 20.4 (CH_3_); HRMS (ESI-TOF) *m*/*z* [M + H]^+^ calcd for C_16_H_18_NO^+^ 240.1383; found 240.1388.

### General Procedure 3: Rh(II)-Catalyzed N–H Bond Insertion

Under argon, Rh_2_(OAc)_4_ (5 mol %) was added to a solution of α-aminoketone **16** (1.1 equiv) and triazole **4** (1.0 equiv) in toluene (0.03 M) in a flame-dried vial with freshly dried 4 Å molecular sieves. The vial was sealed with a Teflon cap and stirred at 80 °C (heating block) until complete (TLC, 2–4 h). The reaction mixture was concentrated in vacuo and purified by flash column chromatography (SiO_2_, gradient from 10–30% EtOAc in petroleum ether) to give the 1,2-diamine product **17**.

#### 1-[(2-Oxo-2-(4-tolyl)ethyl)(4-tolyl)amino]-1-(4-tolyl)-2-(tosylamino)ethene (**17a**)

Triazole **4a** (94 mg, 0.30 mmol, 1.0 equiv) and α-aminoketone **16a** (79 mg, 0.33 mmol, 1.1 equiv) were treated with Rh_2_(OAc)_4_ (7 mg, 0.02 mmol, 5 mol %) in PhMe (10 mL) according to General Procedure 3 to give the title compound **17a** (139 mg, 88%) as a yellow solid: mp 61–69 °C; ν_max_ (film) 3250, 3032, 2924, 1690, 1609, 1512, and 1161 cm^–1^; ^1^H NMR (400 MHz, 22.4 °C, CDCl_3_) δ 9.36 (1 H, d, *J* = 10.6 Hz, NH), 7.87 (2 H, d, *J* = 8.3 Hz, Ar), 7.65 (2 H, d, *J* = 8.3 Hz, Ar), 7.30 (2 H, d, *J* = 8.0 Hz, Ar), 7.17 (2 H, d, *J* = 8.3 Hz, Ar), 7.12 (2 H, d, *J* = 8.3 Hz, Ar), 7.10 (2 H, d, *J* = 8.3 Hz, Ar), 6.92 (1 H, d, *J* = 10.6 Hz, =CH), 6.75 (2 H, d, *J* = 8.6 Hz, Ar), 6.30 (2 H, d, *J* = 8.6 Hz, Ar), 4.66 (2 H, br s, CH_2_), 2.45 (3 H, s, CH_3_), 2.34 (3 H, s, CH_3_), 2.33 (3 H, s, CH_3_) and 2.20 (3 H, s, CH_3_); ^13^C{^1^H} NMR (101 MHz, 23.2 °C, CDCl_3_) δ 197.2 (C=O), 145.3 (Ar), 143.3 (Ar), 143.0 (Ar), 137.9 (Ar), 137.4 (Ar), 132.7 (Ar), 131.8 (Ar), 129.7 (2 × ArH), 129.6 (2 × ArH), 129.5 (2 × ArH), 129.4 (2 × ArH), 128.3 (2 × ArH), 127.1 (Ar), 126.6 (2 × ArH), 125.9 (=C), 124.9 (2 × ArH), 121.8 (=CH), 112.2 (2 × ArH), 56.5 (CH_2_), 21.8 (CH_3_), 21.4 (CH_3_), 21.1 (CH_3_) and 20.3 (CH_3_); HRMS (ESI-TOF) *m*/*z* [M + H]^+^ calcd for C_32_H_33_N_2_O_3_S^+^ 525.2206; found 525.2204.

#### 1-[(4-Chlorophenyl)(2-oxo-2-(4-tolyl)ethyl)amino]-1-(4-tolyl)-2-(tosylamino)ethene (**17b**)

Triazole **4a** (94 mg, 0.30 mmol, 1.0 equiv) and α-aminoketone **16b** (86 mg, 0.33 mmol, 1.1 equiv) were treated with Rh_2_(OAc)_4_ (7 mg, 0.02 mmol, 5 mol %) in PhMe (10 mL) according to General Procedure 3 to give the title compound **17b** (147 mg, 90%) as a yellow solid: mp 72 °C dec; ν_max_ (film) 3264, 3063, 2924, 1686, 1605, 1493, 1339, 1289, 1161, and 1092 cm^–1^; ^1^H NMR (400 MHz, 25.0 °C, CDCl_3_) δ 9.40 (1 H, d, *J* = 10.6 Hz, NH), 7.89 (2 H, d, *J* = 8.2 Hz, COTol), 7.64 (2 H, d, *J* = 8.3 Hz, SO_2_Tol), 7.32 (2 H, d, *J* = 7.9 Hz, Tol), 7.18–7.12 (6 H, m, Ar), 6.94 (1 H, d, *J* = 10.6 Hz, =CH), 6.84 (2 H, d, *J* = 9.1 Hz, NTol), 6.30 (2 H, d, *J* = 9.1 Hz, NTol), 4.68 (2 H, br s, CH_2_), 2.47 (3 H, s, CH_3_), 2.40 (3 H, s, CH_3_) and 2.36 (3 H, s, CH_3_); ^13^C{^1^H} NMR (101 MHz, 25.0 °C, CDCl_3_) δ 196.8 (C=O), 145.7 (Ar), 144.5 (Ar), 143.4 (Ar), 138.1 (Ar), 137.8 (Ar), 132.2 (Ar), 131.6 (Ar), 129.7 (2 × ArH), 129.6 (2 × ArH), 129.5 (2 × ArH), 128.9 (2 × ArH), 128.4 (2 × ArH), 126.5 (2 × ArH), 125.5 (=C), 124.8 (2 × ArH), 123.0 (Ar), 122.1 (=CH), 113.5 (2 × ArH), 56.4 (OCH_3_), 21.8 (CH_3_), 21.5 (CH_3_) and 21.1 (CH_3_); HRMS (ESI-TOF) *m*/*z* [M + H]^+^ calcd for C_31_H_30_ClN_2_O_3_S^+^ 545.1660; found 545.1655.

#### 1-[(2-Oxo-2-(4-tolyl)ethyl)(3-trifluoromethylphenyl)amino]-1-(4-tolyl)-2-(tosylamino)ethene (**17c**)

Triazole **4a** (94 mg, 0.30 mmol, 1.0 equiv) and α-aminoketone **16c** (97 mg, 0.33 mmol, 1.1 equiv) were treated with Rh_2_(OAc)_4_ (7 mg, 0.02 mmol, 5 mol %) in PhMe (10 mL) according to General Procedure 3 to give the title compound **17c** (142 mg, 82%) as a white solid: mp 110 °C dec; ν_max_ (film) 3136, 3032, 2928, 1678, 1609, 1335, and 1165 cm^–1^; ^1^H NMR (400 MHz, 25.0 °C, CDCl_3_) δ 9.40 (1 H, d, *J* = 10.6 Hz, NH), 7.86 (2 H, d, *J* = 8.3 Hz, Ar), 7.65 (2 H, d, *J* = 8.3 Hz, Ar), 7.31 (2 H, d, *J* = 7.9 Hz, Ar), 7.15–7.10 (6 H, m, Ar), 7.06 (1 H, dd, *J* = 8.1, 7.9 Hz, Ar), 6.97 (1 H, d, *J* = 10.6 Hz, =CH), 6.94 (1 H, d, *J* = 7.9 Hz, Ar), 6.64–6.60 (1 H, m, Ar), 6.57 (1 H, dd, *J* = 8.1, 2.6 Hz, Ar), 4.71 (2 H, br s, CH_2_), 2.45 (3 H, s, CH_3_), 2.34 (3 H, s, CH_3_) and 2.32 (3 H, s, CH_3_); ^13^C{^1^H} NMR (101 MHz, 25.0 °C, CDCl_3_) δ 196.6 (C=O), 146.2 (Ar), 145.8 (Ar), 143.4 (Ar), 137.8 (Ar), 137.7 (Ar), 131.8 (Ar), 131.8 (q, *J* = 33.3 Hz, Ar), 131.5 (Ar), 129.8 (ArH), 129.8 (2 × ArH), 129.7 (2 × ArH), 129.6 (2 × ArH), 128.4 (2 × ArH), 126.6 (2 × ArH), 124.7 (2 × ArH), 124.5 (=C), 122.5 (=CH), 115.5 (ArH), 114.9 (q, *J* = 4.1 Hz, ArH), 108.5 (q, *J* = 2.4 Hz, ArH), 56.5 (CH_2_), 21.9 (CH_3_), 21.4 (CH_3_) and 21.1 (CH_3_); HRMS (ESI-TOF) *m*/*z* [M + H]^+^ calcd for C_32_H_30_F_3_N_2_O_3_S^+^ 579.1924; found 579.1928.

#### 1-[(4-Methoxyphenyl)(2-oxo-2-(4-tolyl)ethyl)amino]-1-(4-tolyl)-2-(tosylamino)ethene (**17d**)

Triazole **4a** (94 mg, 0.30 mmol, 1.0 equiv) and α-aminoketone **16d** (84 mg, 0.33 mmol, 1.1 equiv) were treated with Rh_2_(OAc)_4_ (7 mg, 0.02 mmol, 5 mol %) in PhMe (10 mL) according to General Procedure 3 to give the title compound **17d** (144 mg, 89%) as a yellow solid: mp 45–50 °C; ν_max_ (film) 3252, 2924, 2855, 1690, 1609, 1512, 1250, 1161, and 1034 cm^–1^; ^1^H NMR (500 MHz, 25.1 °C, CDCl_3_) δ 9.32 (1 H, d, *J* = 10.6 Hz, NH), 7.86 (2 H, d, *J* = 8.3 Hz, COTol), 7.63 (2 H, d, *J* = 8.3 Hz, SO_2_Tol), 7.28 (2 H, d, *J* = 8.0 Hz, COTol), 7.16 (2 H, d, *J* = 8.3 Hz, Tol), 7.11 (2 H, d, *J* = 8.3 Hz, SO_2_Tol), 7.10 (2 H, d, *J* = 8.0 Hz, Tol), 6.88 (1 H, d, *J* = 10.6 Hz, =CH), 6.53 (2 H, d, *J* = 9.1 Hz, NC_6_H_4_OMe), 6.32 (2 H, d, *J* = 9.1 Hz, NC_6_H_4_OMe), 4.63 (2 H, br s, CH_2_), 3.70 (3 H, s, OCH_3_), 2.44 (3 H, s, COTol), 2.33 (3 H, s, CH_3_) and 2.33 (3 H, s, CH_3_); ^13^C{^1^H} NMR (101 MHz, 25.0 °C, CDCl_3_) δ 197.4 (C=O), 152.4 (Ar), 145.4 (Ar), 143.1 (Ar), 139.9 (Ar), 138.0 (Ar), 137.5 (Ar), 132.8 (Ar), 131.9 (Ar), 129.6 (2 × ArH), 129.6 (2 × ArH), 129.5 (2 × ArH), 128.3 (2 × ArH), 126.7 (2 × ArH), 126.2 (=C), 124.9 (2 × ArH), 121.8 (=CH), 114.7 (2 × ArH), 113.3 (2 × ArH), 56.8 (CH_2_), 55.6 (OCH_3_), 21.8 (CH_3_), 21.5 (CH_3_) and 21.1 (CH_3_); HRMS (ESI-TOF) *m*/*z* [M – H]^−^ calcd for C_32_H_31_N_2_O_4_S^–^ 539.2010; found 539.2017.

#### 1-[(1-Oxo-1-(4-tolyl)prop-2-yl)(4-tolyl)amino]-1-(4-tolyl)-2-(tosylamino)ethene (**17g**)

Triazole **4a** (94 mg, 0.30 mmol, 1.0 equiv) and α-aminoketone **16g** (84 mg, 0.33 mmol, 1.1 equiv) were treated with Rh_2_(OAc)_4_ (7 mg, 0.02 mmol, 5 mol %) in PhMe (10 mL) according to General Procedure 3 to give the title compound **17g** (142 mg, 88%) as a yellow solid: mp 65 °C dec; ν_max_ (film) 3094, 3028, 2924, 1674, 1605, 1512, 1343, 1289, 1235, and 1157 cm^–1^; ^1^H NMR (500 MHz, 25.0 °C, CDCl_3_) δ 10.51 (1 H, d, *J* = 10.8 Hz, NH), 8.09 (2 H, d, *J* = 8.2 Hz, COTol), 7.72 (2 H, d, *J* = 8.3 Hz, SO_2_Tol), 7.37 (2 H, d, *J* = 8.2 Hz, COTol), 7.31 (1 H, d, *J* = 10.8 Hz, =CH), 7.15 (2 H, d, *J* = 8.3 Hz, SO_2_Tol), 7.11 (2 H, d, *J* = 8.1 Hz, Tol), 7.04 (2 H, d, *J* = 8.1 Hz, Tol), 6.70 (2 H, d, *J* = 8.7 Hz, NTol), 6.25 (2 H, d, *J* = 8.7 Hz, NTol), 5.48 (1 H, q, *J* = 7.4 Hz, CH), 2.49 (3 H, s, COTol), 2.35 (3 H, s, SO_2_Tol), 2.28 (3 H, s, Tol), 2.13 (3 H, s, NTol) and 1.11 (3 H, d, *J* = 7.4 Hz, CH_3_); ^13^C{^1^H} NMR (101 MHz, 25.0 °C, CDCl_3_) δ 203.3 (C=O), 145.5 (Ar), 143.2 (Ar), 143.0 (Ar), 138.1 (Ar), 136.6 (Ar), 134.6 (Ar), 131.4 (Ar), 129.9 (2 × ArH), 129.7 (2 × ArH), 129.5 (2 × ArH), 129.5 (2 × ArH), 128.7 (2 × ArH), 126.9 (Ar), 126.7 (2 × ArH), 125.5 (=CH), 123.9 (2 × ArH), 121.2 (=C), 112.3 (2 × ArH), 57.7 (CH), 21.8 (CH_3_), 21.5 (CH_3_), 21.0 (CH_3_), 20.2 (CH_3_) and 16.0 (CH_3_); HRMS (ESI-TOF) *m*/*z* [M + H]^+^ calcd for C_33_H_35_N_2_O_3_S^+^ 539.2363; found 539.2368.

#### 1-[(2-(Naphth-2-yl)-2-oxoethyl)(4-tolyl)amino]-1-(4-tolyl)-2-(tosylamino)ethene (**17h**)

Triazole **4a** (94 mg, 0.30 mmol, 1.0 equiv) and α-aminoketone **16h** (91 mg, 0.33 mmol, 1.1 equiv) were treated with Rh_2_(OAc)_4_ (7 mg, 0.02 mmol, 5 mol %) in PhMe (10 mL) according to General Procedure 3 to give the title compound **17h** (159 mg, 95%) as a yellow solid: mp 58 °C dec; ν_max_ (film) 3240, 3032, 2920, 2862, 1678, 1512, 1161, and 1092 cm^–1^; ^1^H NMR (400 MHz, 25.4 °C, CDCl_3_) δ 9.31 (1 H, d, *J* = 10.7 Hz, NH), 8.46 (1 H, s, naphthyl CH), 8.02 (1 H, dd, *J* = 8.6, 1.7 Hz, naphthyl CH), 7.95 (1 H, d, *J* = 7.5 Hz, naphthyl CH), 7.94 (1 H, d, *J* = 8.7 Hz, naphthyl CH), 7.91 (1 H, d, *J* = 8.0 Hz, naphthyl CH), 7.66 (2 H, d, *J* = 8.3 Hz, Ar), 7.65–7.63 (1 H, m, naphthyl CH), 7.59 (1 H, ddd, *J* = 8.1, 6.9, 1.3 Hz, naphthyl CH), 7.21 (2 H, d, *J* = 8.3 Hz, Ar), 7.13 (2 H, d, *J* = 8.1 Hz, Ar), 7.08 (2 H, d, *J* = 8.1 Hz, Ar), 6.94 (1 H, d, *J* = 10.7 Hz, =CH), 6.77 (2 H, d, *J* = 7.9 Hz, Ar), 6.34 (2 H, d, *J* = 8.7 Hz, Ar), 4.81 (2 H, br s, CH_2_), 2.34 (3 H, s, CH_3_), 2.26 (3 H, s, CH_3_) and 2.20 (3 H, s, CH_3_); ^13^C{^1^H} NMR (101 MHz, 24.8 °C, CDCl_3_) δ 197.6 (C=O), 143.3 (Ar), 143.1 (naphthyl C), 138.0 (Ar), 137.5 (naphthyl C), 136.1 (naphthyl C), 132.8 (Ar), 132.3 (Ar), 131.7 (Ar), 130.0 (naphthyl CH), 129.7 (2 × ArH), 129.6 (2 × ArH), 129.6 (naphthyl CH), 129.4 (2 × ArH), 129.1 (naphthyl CH), 128.8 (naphthyl CH), 127.9 (naphthyl CH), 127.3 (Ar), 127.2 (naphthyl CH), 126.7 (2 × ArH), 125.9 (=C), 125.0 (2 × ArH), 123.6 (naphthyl CH), 122.0 (=CH), 112.3 (2 × ArH), 56.8 (CH_2_), 21.4 (CH_3_), 21.1 (CH_3_) and 20.3 (CH_3_); HRMS (ESI-TOF) *m*/*z* [M + H]^+^ calcd for C_35_H_33_N_2_O_3_S^+^ 561.2206; found 561.2208.

#### 1-[(2-(4-Methoxyphenyl)-2-oxoethyl)(4-tolyl)amino]-1-(4-tolyl)-2-(tosylamino)ethene (**17i**)

Triazole **4a** (94 mg, 0.30 mmol, 1.0 equiv) and α-aminoketone **16i** (84 mg, 0.33 mmol, 1.1 equiv) were treated with Rh_2_(OAc)_4_ (7 mg, 0.02 mmol, 5 mol %) in PhMe (10 mL) according to General Procedure 3 to give the title compound **17i** (123 mg, 76%) as a brown solid: mp 69–74 °C; ν_max_ (film) 3225, 3028, 2920, 2859, 1674, 1597, 1512, 1339, 1265, 1234, 1161, and 1092 cm^–1^; ^1^H NMR (400 MHz, 25.4 °C, CDCl_3_) δ 9.45 (1 H, d, *J* = 10.6 Hz, NH), 7.95 (2 H, d, *J* = 8.9 Hz, Ar), 7.63 (2 H, d, *J* = 8.3 Hz, Ar), 7.16 (2 H, d, *J* = 8.3 Hz, Ar), 7.12 (2 H, d, *J* = 8.0 Hz, Ar), 7.10 (2 H, d, *J* = 8.6 Hz, Ar), 6.95 (2 H, d, *J* = 8.9 Hz, Ar), 6.90 (1 H, d, *J* = 10.6 Hz, =CH), 6.74 (2 H, d, *J* = 8.0 Hz, Ar), 6.29 (2 H, d, *J* = 8.6 Hz, Ar), 4.62 (2 H, br s, CH_2_), 3.89 (3 H, s, OCH_3_), 2.34 (3 H, s, CH_3_), 2.32 (3 H, s, CH_3_) and 2.19 (3 H, s, CH_3_); ^13^C{^1^H} NMR (101 MHz, 25.1 °C, CDCl_3_) δ 196.0 (C=O), 164.4 (Ar), 143.4 (Ar), 143.0 (Ar), 138.1 (Ar), 137.4 (Ar), 132.8 (Ar), 130.6 (2 × ArH), 129.7 (2 × ArH), 129.6 (2 × ArH), 129.4 (2 × ArH), 127.4 (Ar), 127.1 (Ar), 126.7 (2 × ArH), 125.9 (=C), 124.9 (2 × ArH), 121.9 (=CH), 114.0 (2 × ArH), 112.2 (2 × ArH), 56.3 (CH_2_), 55.6 (OCH_3_), 21.5 (CH_3_), 21.1 (CH_3_) and 20.3 (CH_3_); HRMS (ESI-TOF) *m*/*z* [M + H]^+^ calcd for C_32_H_33_N_2_O_4_S^+^ 541.2156; found 541.2158.

#### 1-[(2-(4-Nitrophenyl)-2-oxoethyl)(4-tolyl)amino]-1-(4-tolyl)-2-(tosylamino)ethene (**17j**)

Triazole **4a** (94 mg, 0.30 mmol, 1.0 equiv) and α-aminoketone **16j** (89 mg, 0.33 mmol, 1.1 equiv) were treated with Rh_2_(OAc)_4_ (7 mg, 0.02 mmol, 5 mol %) in PhMe (10 mL) according to General Procedure 3 to give the title compound **17j** (70 mg, 42%) as a pink solid. Under the reaction conditions, there was also some cyclodehydration to the corresponding pyrrole **18j** (80 mg, 50%): mp 115 °C dec; ν_max_ (film) 3183, 2924, 2856, 1694, 1516, 1343, 1215, 1161, and 1092 cm^–1^; ^1^H NMR (400 MHz, 24.9 °C, CDCl_3_) δ 8.78 (1 H, d, *J* = 10.8 Hz, NH), 8.34 (2 H, d, *J* = 8.8 Hz, Ar), 8.11 (2 H, d, *J* = 8.8 Hz, Ar), 7.64 (2 H, d, *J* = 8.4 Hz, Ar), 7.17 (2 H, d, *J* = 8.4 Hz, Ar), 7.15 (2 H, d, *J* = 8.0 Hz, Ar), 7.11 (2 H, d, *J* = 8.6 Hz, Ar), 6.88 (1 H, d, *J* = 10.8 Hz, =CH), 6.76 (2 H, d, *J* = 8.0 Hz, Ar), 6.28 (2 H, d, *J* = 8.6 Hz, Ar), 4.71 (2 H, s, CH_2_), 2.37 (3 H, s, CH_3_), 2.33 (3 H, s, CH_3_) and 2.21 (3 H, s, CH_3_); ^13^C{^1^H} NMR (101 MHz, 25.0 °C, CDCl_3_) δ 196.5 (C=O), 150.9 (Ar), 143.3 (Ar), 143.0 (Ar), 138.7 (Ar), 137.9 (Ar), 137.8 (Ar), 132.4 (Ar), 129.8 (2 × ArH), 129.7 (2 × ArH), 129.5 (2 × ArH), 129.3 (2 × ArH), 127.8 (Ar), 126.7 (2 × ArH), 125.4 (=C), 125.0 (2 × ArH), 124.1 (2 × ArH), 121.6 (=CH), 112.3 (2 × ArH), 57.0 (CH_2_), 21.5 (CH_3_), 21.1 (CH_3_) and 20.3 (CH_3_); HRMS (ESI-TOF) *m*/*z* [M + H]^+^ calcd for C_31_H_30_N_3_O_5_S^+^ 556.1901; found 556.1883.

#### 1-[(2-(4-Cyanophenyl)-2-oxoethyl)(4-tolyl)amino]-1-(4-tolyl)-2-(tosylamino)ethene (**17k**)

Triazole **4a** (94 mg, 0.30 mmol, 1.0 equiv) and α-aminoketone **16k** (83 mg, 0.33 mmol, 1.1 equiv) were treated with Rh_2_(OAc)_4_ (7 mg, 0.02 mmol, 5 mol %) in PhMe (10 mL) according to General Procedure 3 to give the title compound **17k** (65 mg, 40%) as a yellow solid. Under the reaction conditions, there was also some cyclodehydration to the corresponding pyrrole **18k** (78 mg, 50%): mp 113 °C dec; ν_max_ (film) 3245, 3028, 2924, 2856, 2230, 1690, 1512, 1161, and 1092 cm^–1^; ^1^H NMR (500 MHz, 25.0 °C, CDCl_3_) δ 8.81 (1 H, d, *J* = 10.8 Hz, NH), 8.04 (2 H, d, *J* = 8.8 Hz, Ar), 7.80 (2 H, d, *J* = 8.8 Hz, Ar), 7.64 (2 H, d, *J* = 8.3 Hz, Ar), 7.16 (2 H, d, *J* = 7.9 Hz, Ar), 7.14 (2 H, d, *J* = 8.7 Hz, Ar), 7.11 (2 H, d, *J* = 8.3 Hz, Ar), 6.88 (1 H, d, *J* = 10.8 Hz, =CH), 6.75 (2 H, d, *J* = 7.9 Hz, Ar), 6.27 (2 H, d, *J* = 8.7 Hz, Ar), 4.68 (2 H, s, CH_2_), 2.37 (3 H, s, CH_3_), 2.33 (3 H, s, CH_3_) and 2.20 (3 H, s, CH_3_); ^13^C{^1^H} NMR (101 MHz, 26.0 °C, CDCl_3_) δ 196.7 (C=O), 143.2 (Ar), 143.0 (Ar), 137.9 (Ar), 137.7 (Ar), 137.2 (Ar), 132.7 (2 × ArH), 132.4 (Ar), 129.8 (2 × ArH), 129.7 (2 × ArH), 129.5 (2 × ArH), 128.6 (2 × ArH), 127.8 (Ar), 126.7 (2 × ArH), 125.4 (=C), 125.0 (2 × ArH), 121.6 (=CH), 117.6 (Ar), 117.5 (C≡N), 112.3 (2 × ArH), 56.9 (CH_2_), 21.5 (CH_3_), 21.1 (CH_3_) and 20.3 (CH_3_); HRMS (ESI-TOF) *m*/*z* [M + H]^+^ calcd for C_32_H_30_N_3_O_3_S^+^ 536.2002; found 536.2005.

#### 1-[(2-(4-Chlorophenyl)-2-oxoethyl)(4-tolyl)amino]-1-(4-tolyl)-2-(tosylamino)ethene (**17l**)

Triazole **4a** (94 mg, 0.30 mmol, 1.0 equiv) and α-aminoketone **16l** (86 mg, 0.33 mmol, 1.1 equiv) were treated with Rh_2_(OAc)_4_ (7 mg, 0.02 mmol, 5 mol %) in PhMe (10 mL) according to General Procedure 3 to give the title compound **17l** (109 mg, 67%) as a yellow solid. Under the reaction conditions, there was also some cyclodehydration to the corresponding pyrrole **18l** (50 mg, 32%): mp 63 °C dec; ν_max_ (film) 3175, 3028, 2920, 1686, 1589, 1512, 1339, 1223, 1161, and 1092 cm^–1^; ^1^H NMR (400 MHz, 26.0 °C, CDCl_3_) δ 9.09 (1 H, d, *J* = 10.7 Hz, NH), 7.89 (2 H, d, *J* = 8.6 Hz, Ar), 7.63 (2 H, d, *J* = 8.3 Hz, Ar), 7.47 (2 H, d, *J* = 8.6 Hz, Ar), 7.15 (2 H, d, *J* = 8.3 Hz, Ar), 7.12 (2 H, d, *J* = 8.6 Hz, Ar), 7.10 (2 H, d, *J* = 8.6 Hz, Ar), 6.90 (1 H, d, *J* = 10.7 Hz, =CH), 6.75 (2 H, d, *J* = 8.7 Hz, Ar), 6.28 (2 H, d, *J* = 8.7 Hz, Ar), 4.64 (2 H, br s, CH_2_), 2.35 (3 H, s, CH_3_), 2.33 (3 H, s, CH_3_) and 2.20 (3 H, s, CH_3_); ^13^C{^1^H} NMR (101 MHz, 26.0 °C, CDCl_3_) δ 196.6 (C=O), 143.2 (Ar), 143.1 (Ar), 140.9 (Ar), 137.9 (Ar), 137.6 (Ar), 132.6 (Ar), 132.6 (Ar), 129.7 (2 × ArH), 129.6 (2 × ArH), 129.6 (2 × ArH), 129.4 (2 × ArH), 129.2 (2 × ArH), 127.5 (Ar), 126.7 (2 × ArH), 125.7 (=C), 124.9 (2 × ArH), 121.8 (=C), 112.2 (2 × ArH), 56.6 (CH_2_), 21.5 (CH_3_), 21.1 (CH_3_) and 20.3 (CH_3_); HRMS (ESI-TOF) *m*/*z* [M + H]^+^ calcd for C_31_H_30_ClN_2_O_3_S^+^ 545.1660; found 545.1659.

#### 1-[(2-Oxo-2-(4-tolyl)ethyl)(4-tolyl)amino]-1-(thiophen-2-yl)-2-(tosylamino)ethene (**17m**)

Triazole **4m** (92 mg, 0.30 mmol, 1.0 equiv) and α-aminoketone **16a** (79 mg, 0.33 mmol, 1.1 equiv) were treated with Rh_2_(OAc)_4_ (7 mg, 0.02 mmol, 5 mol %) in PhMe (10 mL) according to General Procedure 3 to give the title compound **17m** (124 mg, 80%) as an orange solid: mp 126 °C dec; ν_max_ (film) 3148, 3032, 2920, 1678, 1609, 1516, 1339, 1231, 1161, and 1092 cm^–1^; ^1^H NMR (400 MHz, 25.0 °C, CDCl_3_) δ 9.39 (1 H, d, *J* = 10.7 Hz, NH), 7.88 (2 H, d, *J* = 8.3 Hz, Ar), 7.64 (2 H, d, *J* = 8.3 Hz, Ar), 7.31 (2 H, d, *J* = 8.3 Hz, Ar), 7.12 (1 H, dd, *J* = 5.1, 1.2 Hz, thiophene CH), 7.10 (2 H, d, *J* = 8.3 Hz, Ar), 6.92 (1 H, dd, *J* = 5.1, 3.6 Hz, thiophene CH), 6.90 (1 H, d, *J* = 10.7 Hz, =CH), 6.84 (1 H, dd, *J* = 3.6, 1.2 Hz, thiophene CH), 6.77 (2 H, d, *J* = 8.3 Hz, Ar), 6.31 (2 H, d, *J* = 8.3 Hz, Ar), 4.71 (2 H, br s, CH_2_), 2.45 (3 H, s, CH_3_), 2.33 (3 H, s, CH_3_) and 2.19 (3 H, s, CH_3_); ^13^C{^1^H} NMR (101 MHz, 25.0 °C, CDCl_3_) δ 197.2 (C=O), 145.5 (Ar), 143.1 (Ar), 142.6 (thiophene C2), 141.0 (Ar), 137.8 (Ar), 131.8 (Ar), 129.7 (2 × ArH), 129.6 (2 × ArH), 129.4 (2 × ArH), 128.3 (2 × ArH), 127.7 (thiophene CH), 127.5 (Ar), 126.7 (2 × ArH), 123.8 (thiophene CH), 122.9 (thiophene CH), 122.0 (=CH), 121.7 (=C), 112.4 (2 × ArH), 56.5 (CH_2_), 21.8 (CH_3_), 21.5 (CH_3_) and 20.3 (CH_3_); HRMS (ESI-TOF) *m*/*z* [M + H]^+^ calcd for C_29_H_29_N_2_O_3_S_2_^+^ 517.1614; found 517.1617.

#### 1-(4-Methoxyphenyl)-1-[(2-oxo-2-(4-tolyl)ethyl)(4-tolyl)amino]-2-(tosylamino)ethene (**17n**)

Triazole **4n** (99 mg, 0.30 mmol, 1.0 equiv) and α-aminoketone **16a** (79 mg, 0.33 mmol, 1.1 equiv) were treated with Rh_2_(OAc)_4_ (7 mg, 0.02 mmol, 5 mol %) in PhMe (10 mL) according to General Procedure 3 to give the title compound **17n** (152 mg, 94%) as an orange solid: mp 66–70 °C; ν_max_ (film) 3275, 3032, 2924, 1682, 1601, 1512, 1339, 1250, and 1161 cm^–1^; ^1^H NMR (400 MHz, 25.0 °C, CDCl_3_) δ 9.27 (1 H, d, *J* = 10.6 Hz, NH), 7.86 (2 H, d, *J* = 8.3 Hz, Ar), 7.64 (2 H, d, *J* = 8.3 Hz, Ar), 7.28 (2 H, d, *J* = 8.3 Hz, Ar), 7.19 (2 H, d, *J* = 8.8 Hz, Ar), 7.11 (2 H, d, *J* = 8.3 Hz, Ar), 6.82 (2 H, d, *J* = 8.8 Hz, Ar), 6.81 (1 H, d, *J* = 10.6 Hz, =CH), 6.74 (2 H, d, *J* = 8.6 Hz, Ar), 6.29 (2 H, d, *J* = 8.6 Hz, Ar), 4.63 (2 H, br s, CH_2_), 3.79 (3 H, s, CH_3_), 2.44 (3 H, s, CH_3_), 2.34 (3 H, s, CH_3_) and 2.19 (3 H, s, CH_3_); ^13^C{^1^H} NMR (101 MHz, 25.0 °C, CDCl_3_) δ 197.2 (C=O), 159.3 (Ar), 145.3 (Ar), 143.3 (Ar), 143.0 (Ar), 138.0 (Ar), 131.9 (Ar), 129.7 (2 × ArH), 129.5 (2 × ArH), 129.4 (2 × ArH), 128.3 (2 × ArH), 128.1 (Ar), 127.2 (Ar), 126.7 (2 × ArH), 126.3 (2 × ArH), 125.8 (=C), 120.8 (=CH), 114.3 (2 × ArH), 112.3 (2 × ArH), 56.4 (CH_2_), 55.4 (OCH_3_), 21.8 (CH_3_), 21.5 (CH_3_) and 20.3 (CH_3_); HRMS (ESI-TOF) *m*/*z* [M + H]^+^ calcd for C_32_H_33_N_2_O_4_S^+^ 541.2156; found 541.2148.

#### 1-(Cyclohexen-1-yl)-1-[(2-oxo-2-(4-tolyl)ethyl)(4-tolyl)amino]-2-(tosylamino)ethene (**17o**)

Triazole **4o** (91 mg, 0.30 mmol, 1.0 equiv) and α-aminoketone **16a** (79 mg, 0.33 mmol, 1.1 equiv) were treated with Rh_2_(OAc)_4_ (7 mg, 0.02 mmol, 5 mol %) in PhMe (10 mL) according to General Procedure 3 to give the title compound **17o** (126 mg, 82%) as a yellow solid: mp 140 °C dec; ν_max_ (film) 3152, 3032, 2924, 1678, 1609, 1516, 1343, and 1161 cm^–1^; ^1^H NMR (400 MHz, 25.0 °C, CDCl_3_) δ 9.41 (1 H, d, *J* = 10.7 Hz, NH), 7.90 (2 H, d, *J* = 8.2 Hz, Ar), 7.64 (2 H, d, *J* = 8.3 Hz, Ar), 7.32 (2 H, d, *J* = 8.2 Hz, Ar), 7.09 (2 H, d, *J* = 8.3 Hz, Ar), 6.79 (2 H, d, *J* = 8.6 Hz, Ar), 6.51 (1 H, d, *J* = 10.7 Hz, =CH), 6.18 (2 H, d, *J* = 8.6 Hz, Ar), 5.46 (1 H, t, *J* = 4.2 Hz, =CH), 4.62 (2 H, br s, CH_2_), 2.46 (3 H, s, CH_3_), 2.31 (3 H, s, CH_3_), 2.19 (3 H, s, CH_3_), 2.15–2.08 (2 H, m, CH_2_), 2.07–2.00 (2 H, m, CH_2_), 1.72–1.64 (2 H, m, CH_2_) and 1.62–1.52 (2 H, m, CH_2_); ^13^C{^1^H} NMR (101 MHz, 25.0 °C, CDCl_3_) δ 197.6 (C=O), 145.3 (Ar), 143.3 (Ar), 142.9 (Ar), 137.8 (Ar), 132.0 (Ar), 129.9 (=C), 129.6 (2 × ArH), 129.5 (2 × ArH), 129.3 (2 × ArH), 128.4 (=C), 128.3 (2 × ArH), 126.6 (2 × ArH), 126.5 (Ar), 123.5 (=CH), 121.3 (=CH), 111.7 (2 × ArH), 57.2 (CH_2_), 25.5 (CH_2_), 24.9 (CH_2_), 22.5 (CH_2_), 22.2 (CH_2_), 21.8 (CH_3_), 21.4 (CH_3_) and 20.3 (CH_3_); HRMS (ESI-TOF) *m*/*z* [M + H]^+^ calcd for C_31_H_35_N_2_O_3_S^+^ 515.2363; found 515.2355.

#### 2-(4-Methoxybenzenesulfonylamino)-1-[(2-oxo-2-(4-tolyl)ethyl)(4-tolyl)amino]-1-(4-tolyl)ethene (**17p**)

Triazole **4r** (99 mg, 0.30 mmol, 1.0 equiv) and α-aminoketone **16a** (79 mg, 0.33 mmol, 1.1 equiv) were treated with Rh_2_(OAc)_4_ (7 mg, 0.02 mmol, 5 mol %) in PhMe (10 mL) according to General Procedure 3 to give the title compound **17p** (142 mg, 87%) as a yellow solid: mp 60–66 °C; ν_max_ (film) 3148, 3028, 2920, 1678, 1597, 1516, 1339, 1304, 1258, 1157, and 1092 cm^–1^; ^1^H NMR (400 MHz, 25.0 °C, CDCl_3_) δ 9.31 (1 H, d, *J* = 10.7 Hz, NH), 7.86 (2 H, d, *J* = 8.3 Hz, Ar), 7.69 (2 H, d, *J* = 9.0 Hz, Ar), 7.28 (2 H, d, *J* = 7.8 Hz, Ar), 7.16 (2 H, d, *J* = 8.3 Hz, Ar), 7.10 (2 H, d, *J* = 7.8 Hz, Ar), 6.90 (1 H, d, *J* = 10.7 Hz, =CH), 6.77 (2 H, d, *J* = 9.0 Hz, Ar), 6.76 (2 H, d, *J* = 8.4 Hz, Ar), 6.30 (2 H, d, *J* = 8.4 Hz, Ar), 4.65 (2 H, br s, CH_2_), 3.79 (3 H, s, OCH_3_), 2.44 (3 H, s, CH_3_), 2.32 (3 H, s, CH_3_) and 2.19 (3 H, s, CH_3_); ^13^C{^1^H} NMR (101 MHz, 25.0 °C, CDCl_3_) δ 197.2 (C=O), 162.6 (Ar), 145.3 (Ar), 143.4 (Ar), 137.4 (Ar), 132.8 (Ar), 132.7 (Ar), 131.9 (Ar), 129.7 (2 × ArH), 129.6 (2 × ArH), 129.5 (2 × ArH), 128.8 (2 × ArH), 128.3 (2 × ArH), 127.2 (Ar), 125.8 (=C), 124.9 (2 × ArH), 122.0 (=CH), 113.9 (2 × ArH), 112.2 (2 × ArH), 56.5 (OCH_3_), 55.4 (CH_3_), 21.8 (CH_3_), 21.1 (CH_3_) and 20.3 (CH_3_); HRMS (ESI-TOF) *m*/*z* [M + H]^+^ calcd for C_32_H_33_N_2_O_4_S^+^ 541.2156; found 541.2158.

#### 2-(4-Nitrobenzenesulfonylamino)-1-[(2-oxo-2-(4-tolyl)ethyl)(4-tolyl)amino]-1-(4-tolyl)ethene (**17q**)

Triazole **4q** (103 mg, 0.30 mmol, 1.0 equiv) and α-aminoketone **16a** (79 mg, 0.33 mmol, 1.1 equiv) were treated with Rh_2_(OAc)_4_ (7 mg, 0.02 mmol, 5 mol %) in PhMe (10 mL) according to General Procedure 3 to give the title compound **17q** (66 mg, 40%) as a yellow solid: mp 143 °C dec; ν_max_ (film) 3102, 3032, 2920, 1678, 1605, 1516, 1346, 1231, and 1165 cm^–1^; ^1^H NMR (400 MHz, 25.0 °C, CDCl_3_) δ 9.77 (1 H, d, *J* = 10.2 Hz, NH), 8.12 (2 H, d, *J* = 9.0 Hz, Ar), 7.88 (2 H, d, *J* = 9.0 Hz, Ar), 7.85 (2 H, d, *J* = 8.3 Hz, Ar), 7.29 (2 H, d, *J* = 8.6 Hz, Ar), 7.18 (2 H, d, *J* = 8.3 Hz, Ar), 7.12 (2 H, d, *J* = 7.9 Hz, Ar), 6.88 (1 H, d, *J* = 10.2 Hz, =CH), 6.68 (2 H, d, *J* = 7.9 Hz, Ar), 6.26 (2 H, d, *J* = 8.6 Hz, Ar), 4.65 (2 H, br s, CH_2_), 2.44 (3 H, s, CH_3_), 2.34 (3 H, s, CH_3_) and 2.16 (3 H, s, CH_3_); ^13^C{^1^H} NMR (101 MHz, 25.0 °C, CDCl_3_) δ 197.9 (C=O), 149.7 (Ar), 146.6 (Ar), 145.9 (Ar), 143.7 (Ar), 138.2 (Ar), 132.1 (Ar), 131.5 (Ar), 129.7 (2 × ArH), 129.7 (2 × ArH), 129.6 (2 × ArH), 128.3 (2 × ArH), 128.0 (Ar), 127.9 (=C), 127.7 (2 × ArH), 125.2 (2 × ArH), 124.0 (2 × ArH), 120.6 (=CH), 112.5 (2 × ArH), 56.6 (CH_2_), 21.8 (CH_3_), 21.1 (CH_3_) and 20.1 (CH_3_); HRMS (ESI-TOF) *m*/*z* [M + H]^+^ calcd for C_31_H_30_N_3_O_5_S^+^ 556.1901; found 556.1903.

#### 2-(Mesylamino)-1-[(2-oxo-2-(4-tolyl)ethyl)(4-tolyl)amino]-1-(4-tolyl)ethene (**17r**)

Triazole **4p** (71 mg, 0.30 mmol, 1.0 equiv) and α-aminoketone **16a** (79 mg, 0.33 mmol, 1.1 equiv) were treated with Rh_2_(OAc)_4_ (7 mg, 0.02 mmol, 5 mol %) in PhMe (10 mL) according to General Procedure 3 to give the title compound **17r** (65 mg, 48%) as a yellow solid: mp 111–116 °C; ν_max_ (film) 2924, 1682, 1609, 1516, 1331, 1231, and 1150 cm^–1^; ^1^H NMR (400 MHz, 25.0 °C, CDCl_3_) δ 9.18 (1 H, d, *J* = 10.4 Hz, NH), 7.91 (2 H, d, *J* = 8.2 Hz, Ar), 7.29 (2 H, d, *J* = 7.9 Hz, Ar), 7.24 (2 H, d, *J* = 8.2 Hz, Ar), 7.14 (2 H, d, *J* = 7.9 Hz, Ar), 6.98 (2 H, d, *J* = 8.5 Hz, Ar), 6.85 (1 H, d, *J* = 10.4 Hz, =CH), 6.54 (2 H, d, *J* = 8.5 Hz, Ar), 4.76 (2 H, br s, CH_2_), 2.90 (3 H, s, CH_3_), 2.43 (3 H, s, CH_3_), 2.35 (3 H, s, CH_3_) and 2.23 (3 H, s, CH_3_); ^13^C{^1^H} NMR (101 MHz, 25.0 °C, CDCl_3_) δ 197.5 (C=O), 145.5 (Ar), 143.7 (Ar), 137.7 (Ar), 132.6 (Ar), 131.8 (Ar), 130.0 (2 × ArH), 129.7 (2 × ArH), 129.6 (2 × ArH), 128.3 (2 × ArH), 127.8 (Ar), 125.7 (=C), 125.0 (2 × ArH), 121.4 (=CH), 112.6 (2 × ArH), 56.4 (CH_2_), 42.0 (CH_3_), 21.8 (CH_3_), 21.1 (CH_3_) and 20.3 (CH_3_); HRMS (ESI-TOF) *m*/*z* [M + H]^+^ calcd for C_26_H_29_N_2_O_3_S^+^ 449.1893; found 449.1901.

### General Procedure 4: Cyclodehydration

Under argon, boron trifluoride diethyl etherate (3.0 equiv) was added in one portion to a solution of 1,2-diamine **17** (1.0 equiv) in CH_2_Cl_2_ (0.03 M) in a flame-dried vial. The vial was sealed with a Teflon cap and heated at 80 °C (heating block) for 15 min. The reaction mixture was cooled to ambient temperature; saturated aqueous NaHCO_3_ was added, and the aqueous phase was extracted with CH_2_Cl_2_ (3 × 30 mL mmol^–1^). The combined organic layers were washed with brine, dried (MgSO_4_), and concentrated in vacuo. Purification by column chromatography (SiO_2_, gradient of 10–30% EtOAc in petroleum ether) gave the pyrrole **18**.

### General Procedure 5: One-Pot Pyrrole Synthesis

Under argon, copper(I) thiophene-2-carboxylate (5 mol %) was added to a solution of alkyne **21** (1.1 equiv) in CH_2_Cl_2_ (0.03 M) in a flame-dried vial with freshly dried 4 Å molecular sieves. The mixture was cooled to 0 °C (ice bath) and stirred for 10 min. Then sulfonyl azide **22** (1.1 equiv) was added, and the reaction mixture was allowed to reach ambient temperature. When the CuAAC reaction was complete (TLC, 6–16 h), amine **16** (1.0 equiv) was added, followed by Rh_2_(OAc)_4_ (1 mol %), and the vial was sealed with a Teflon cap and heated to 80 °C (heating block) for 3 h. The reaction mixture was cooled to ambient temperature and boron trifluoride diethyl etherate (3.0 equiv) was added in one portion. The vial was sealed and heated to 80 °C (heating block) for 15 min. After being cooled to ambient temperature, the reaction was quenched by addition of saturated aqueous NaHCO_3_, and the aqueous phase was extracted with CH_2_Cl_2_ (3 × 30 mL mmol^–1^). The combined organic layers were washed with brine, dried (MgSO_4_), filtered through a short pad of silica (eluting with CH_2_Cl_2_), and concentrated in vacuo to deliver the pyrrole **18**.

#### 3-Tosylamino-1,2,4-tri(4-tolyl)pyrrole (**18a**)

1,2-Diaminoalkene **17a** (105 mg, 0.20 mmol, 1.0 equiv) was treated with BF_3_·OEt_2_ (74 μL, 0.60 mmol, 3.0 equiv) in CH_2_Cl_2_ (6.7 mL) according to General Procedure 4 to give the title compound **18a** (74 mg, 73%) as an orange solid. 4-Ethynyltoluene (64 μL, 0.55 mmol, 1.1 equiv) and 4-toluenesulfonyl azide (108 mg, 0.55 mmol, 1.1 equiv) were treated with CuTC (5 mg, 26 μmol, 5 mol %) in CH_2_Cl_2_ (17 mL), followed by α-aminoketone **16a** (120 mg, 0.50 mmol, 1.0 equiv) and Rh_2_(OAc)_4_ (2 mg, 4.5 μmol, 1 mol %), and finally BF_3_·OEt_2_ (185 μL, 1.5 mmol, 3.0 equiv) according to General Procedure 5 to give the title compound **18a** (228 mg, 90%): mp 170 °C dec; ν_max_ (film) 3268, 3028, 1516, 1389, 1327, and 1157 cm^–1^; ^1^H NMR (500 MHz, 25.0 °C, CDCl_3_) δ 7.35 (2 H, d, *J* = 8.1 Hz, Ar), 7.25 (2 H, d, *J* = 8.0 Hz, Ar), 7.09 (2 H, d, *J* = 8.1 Hz, Ar), 7.07 (2 H, d, *J* = 8.4 Hz, Ar), 6.97 (2 H, d, *J* = 8.4 Hz, Ar), 6.94 (2 H, d, *J* = 8.1 Hz, Ar), 6.88 (1 H, s, pyrrole CH), 6.87 (2 H, d, *J* = 8.1 Hz, Ar), 6.82 (2 H, d, *J* = 8.0 Hz, Ar), 6.24 (1 H, s, NH), 2.37 (3 H, s, CH_3_), 2.33 (3 H, s, CH_3_), 2.32 (3 H, s, CH_3_) and 2.30 (3 H, s, CH_3_); ^13^C{^1^H} NMR (126 MHz, 25.0 °C, CDCl_3_) δ 142.3 (Ar), 137.4 (Ar), 137.0 (Ar), 136.7 (Ar), 136.4 (Ar), 135.5 (Ar), 131.9 (Ar), 131.0 (Ar), 129.8 (2 × ArH), 129.5 (2 × ArH), 129.0 (2 × ArH), 128.7 (2 × ArH), 128.6 (2 × ArH), 127.4 (2 × ArH), 127.2 (pyrrole), 127.0 (2 × ArH), 125.2 (2 × ArH), 123.6 (pyrrole), 119.3 (pyrrole CH), 115.3 (pyrrole), 21.3 (CH_3_), 21.3 (CH_3_), 21.2 (CH_3_) and 21.2 (CH_3_); HRMS (ESI-TOF) *m*/*z* [M + H]^+^ calcd for C_32_H_31_N_2_O_2_S^+^ 507.2101; found 507.2100.

#### 1-(4-Chlorophenyl)-2,4-di(4-tolyl)-3-tosylaminopyrrole (**18b**)

1,2-Diaminoalkene **17b** (109 mg, 0.20 mmol, 1.0 equiv) was treated with BF_3_·OEt_2_ (74 μL, 0.60 mmol, 3.0 equiv) in CH_2_Cl_2_ (6.7 mL) according to General Procedure 4 to give the title compound **18b** (86 mg, 82%) as a yellow solid. 4-Ethynyltoluene (38 μL, 0.33 mmol, 1.1 equiv) and 4-toluenesulfonyl azide (66 mg, 0.33 mmol, 1.1 equiv) were treated with CuTC (3 mg, 16 μmol, 5 mol %) in CH_2_Cl_2_ (10 mL), followed by α-aminoketone **16b** (78 mg, 0.30 mmol, 1.0 equiv) and Rh_2_(OAc)_4_ (1 mg, 2.3 μmol, 1 mol %), and finally BF_3_·OEt_2_ (111 μL, 0.90 mmol, 3.0 equiv) according to General Procedure 5 to give the title compound **18b** (101 mg, 64%): mp 170 °C dec; ν_max_ (film) 3268, 3020, 2920, 1497, 1385, 1327, 1157, and 1092 cm^–1^; ^1^H NMR (400 MHz, 25.0 °C, CDCl_3_) δ 7.31 (2 H, d, *J* = 8.0 Hz, Ar), 7.22 (2 H, d, *J* = 8.0 Hz, Ar), 7.21 (2 H, d, *J* = 8.7 Hz, Ar), 7.07 (2 H, d, *J* = 8.0 Hz, Ar), 6.99 (2 H, d, *J* = 8.7 Hz, Ar), 6.94 (2 H, d, *J* = 7.9 Hz, Ar), 6.85 (1 H, s, pyrrole CH), 6.84 (2 H, d, *J* = 7.9 Hz, Ar), 6.81 (2 H, d, *J* = 8.0 Hz, Ar), 6.19 (1 H, s, NH), 2.35 (3 H, s, CH_3_), 2.31 (3 H, s, CH_3_) and 2.28 (3 H, s, CH_3_); ^13^C{^1^H} NMR (101 MHz, 23.0 °C, CDCl_3_) δ 142.4 (Ar), 138.4 (Ar), 137.1 (Ar), 137.0 (Ar), 135.8 (Ar), 132.3 (Ar), 130.7 (Ar), 129.9 (2 × ArH), 129.3 (Ar), 129.1 (2 × ArH), 129.0 (2 × ArH), 128.9 (2 × ArH), 128.7 (2 × ArH), 127.4 (2 × ArH), 127.0 (2 × ArH), 126.8 (pyrrole), 126.5 (2 × ArH), 124.2 (pyrrole), 119.1 (pyrrole CH), 115.9 (pyrrole), 21.4 (CH_3_), 21.3 (CH_3_) and 21.2 (CH_3_); HRMS (ESI-TOF) *m*/*z* [M + H]^+^ calcd for C_31_H_28_ClN_2_O_2_S^+^ 527.1555; found 527.1562.

#### 2,4-Di(4-tolyl)-3-tosyl-1-(3-trifluoromethylphenyl)aminopyrrole (**18c**)

1,2-Diaminoalkene **17c** (58 mg, 0.10 mmol, 1.0 equiv) was treated with BF_3_·OEt_2_ (37 μL, 0.30 mmol, 3.0 equiv) in CH_2_Cl_2_ (3.3 mL) according to General Procedure 4 to give the title compound **18c** (36 mg, 64%) as a yellow solid. 4-Ethynyltoluene (38 μL, 0.33 mmol, 1.1 equiv) and 4-toluenesulfonyl azide (66 mg, 0.33 mmol, 1.1 equiv) were treated with CuTC (3 mg, 16 μmol, 5 mol %) in CH_2_Cl_2_ (10 mL), followed by α-aminoketone **16c** (88 mg, 0.30 mmol, 1.0 equiv) and Rh_2_(OAc)_4_ (1 mg, 2.3 μmol, 1 mol %), and finally BF_3_·OEt_2_ (111 μL, 0.90 mmol, 3.0 equiv) according to General Procedure 5 to give the title compound **18c** (131 mg, 78%): mp 145 °C dec; ν_max_ (film) 3256, 2924, 1497, 1458, 1385, 1327, 1161, 1130, 1096, and 1072 cm^–1^; ^1^H NMR (400 MHz, 25.0 °C, CDCl_3_) δ 7.48–7.43 (1 H, m, Ar), 7.40–7.37 (1 H, m, Ar), 7.35 (1 H, d, *J* = 7.8 Hz, Ar), 7.31 (2 H, d, *J* = 7.9 Hz, Ar), 7.23 (2 H, d, *J* = 8.2 Hz, Ar), 7.17–7.13 (1 H, m, Ar), 7.07 (2 H, d, *J* = 7.9 Hz, Ar), 6.95 (2 H, d, *J* = 8.0 Hz, Ar), 6.91 (1 H, s, pyrrole CH), 6.84 (2 H, d, *J* = 8.0 Hz, Ar), 6.82 (2 H, d, *J* = 8.2 Hz, Ar), 6.20 (1 H, s, NH), 2.35 (3 H, s, CH_3_), 2.30 (3 H, s, CH_3_) and 2.28 (3 H, s, CH_3_); ^13^C{^1^H} NMR (101 MHz, 25.0 °C, CDCl_3_) δ 142.5 (Ar), 140.3 (Ar), 137.4 (Ar), 136.9 (Ar), 135.9 (Ar), 132.1 (Ar), 131.5 (q, *J* = 32.9 Hz, Ar), 130.5 (Ar), 129.9 (2 × ArH), 129.4 (ArH), 129.0 (2 × ArH), 129.0 (2 × ArH), 128.7 (2 × ArH), 128.6 (ArH), 127.5 (2 × ArH), 127.0 (2 × ArH), 126.6 (pyrrole), 124.6 (pyrrole), 123.5 (q, *J* = 272.4 Hz, CF_3_), 123.2 (q, *J* = 3.8 Hz, ArH), 121.9 (q, *J* = 3.5 Hz, ArH), 118.9 (pyrrole CH), 116.4 (pyrrole), 21.4 (CH_3_), 21.3 (CH_3_) and 21.2 (CH_3_); HRMS (ESI-TOF) *m*/*z* [M + H]^+^ calcd for C_32_H_28_F_3_N_2_O_2_S^+^ 561.1818; found 561.1826.

#### 2,4-Di(4-tolyl)-1-(4-methoxyphenyl)-3-tosylaminopyrrole (**18d**)

1,2-Diaminoalkene **17d** (108 mg, 0.20 mmol, 1.0 equiv) was treated with BF_3_·OEt_2_ (74 μL, 0.60 mmol, 3.0 equiv) in CH_2_Cl_2_ (6.7 mL) according to General Procedure 4 to give the title compound **18d** (69 mg, 66%) as a yellow solid: mp 158 °C dec; ν_max_ (film) 3271, 2920, 2859, 1512, 1323, 1250, 1157, 1092, and 1034 cm^–1^; ^1^H NMR (400 MHz, 22.1 °C, CDCl_3_) δ 7.33 (2 H, d, *J* = 8.0 Hz, Ar), 7.23 (2 H, d, *J* = 8.2 Hz, Ar), 7.06 (2 H, d, *J* = 8.0 Hz, Ar), 6.99 (2 H, d, *J* = 9.0 Hz, Ar), 6.91 (2 H, d, *J* = 7.8 Hz, Ar), 6.84 (2 H, d, *J* = 7.8 Hz, Ar), 6.83 (1 H, s, pyrrole CH), 6.80 (2 H, d, *J* = 8.2 Hz, Ar), 6.77 (2 H, d, *J* = 9.0 Hz, Ar), 6.23 (1 H, s, NH), 3.77 (3 H, s, OCH_3_), 2.34 (3 H, s, CH_3_), 2.29 (3 H, s, CH_3_) and 2.27 (3 H, s, CH_3_); ^13^C{^1^H} NMR (101 MHz, 22.9 °C, CDCl_3_) δ 158.1 (Ar), 142.3 (Ar), 137.0 (Ar), 136.7 (Ar), 135.5 (Ar), 133.0 (Ar), 132.1 (Ar), 131.1 (Ar), 129.9 (2 × ArH), 128.9 (2 × ArH), 128.7 (2 × ArH), 128.6 (2 × ArH), 127.4 (2 × ArH), 127.2 (pyrrole), 127.0 (2 × ArH), 126.7 (2 × ArH), 123.4 (pyrrole), 119.5 (pyrrole CH), 115.0 (pyrrole), 114.0 (2 × ArH), 55.4 (OCH_3_), 21.4 (CH_3_), 21.3 (CH_3_) and 21.2 (CH_3_); HRMS (ESI-TOF) *m*/*z* [M + Na]^+^ calcd for C_32_H_30_N_2_NaO_3_S^+^ 545.1869; found 545.1858.

#### 2,4-Di(4-tolyl)-3-tosyl-1-(2,4,6-trimethylphenyl)aminopyrrole (**18e**)

Triazole **4a** (94 mg, 0.30 mmol, 1.0 equiv) and α-aminoketone **16e** (88 mg, 0.33 mmol, 1.1 equiv) were treated with Rh_2_(OAc)_4_ (7 mg, 0.02 mmol, 5 mol %) in PhMe (10 mL) according to General Procedure 3. During the N–H insertion, spontaneous cyclodehydration occurred to give the title compound **18e** (111 mg, 69%) as a yellow wax: ν_max_ (film) 3264, 3024, 2920, 2866, 1493, 1381, 1327, and 1161 cm^–1^; ^1^H NMR (400 MHz, 25.0 °C, CDCl_3_) δ 7.35 (2 H, d, *J* = 8.0 Hz, Ar), 7.19 (2 H, d, *J* = 8.3 Hz, Ar), 7.09 (2 H, d, *J* = 8.0 Hz, Ar), 6.84 (2 H, d, *J* = 8.0 Hz, Ar), 6.81 (2 H, s, Ar), 6.80 (2 H, d, *J* = 8.3 Hz, Ar), 6.69 (2 H, d, *J* = 8.0 Hz, Ar), 6.57 (1 H, s, pyrrole CH), 6.27 (1 H, s, NH), 2.35 (3 H, s, CH_3_), 2.28 (3 H, s, CH_3_), 2.25 (3 H, s, CH_3_), 2.24 (3 H, s, CH_3_) and 1.92 (6 H, s, 2 × CH_3_); ^13^C{^1^H} NMR (101 MHz, 25.0 °C, CDCl_3_) δ 142.5 (Ar), 137.9 (Ar), 136.5 (Ar), 136.0 (Ar), 135.7 (2 × Ar), 135.6 (Ar), 135.4 (Ar), 132.0 (Ar), 131.3 (Ar), 128.9 (2 × ArH), 128.7 (2 × ArH), 128.6 (2 × ArH), 128.5 (4 × ArH), 127.3 (pyrrole), 127.3 (2 × ArH), 127.2 (2 × ArH), 123.4 (pyrrole), 118.6 (pyrrole CH), 113.7 (pyrrole), 21.5 (CH_3_), 21.2 (CH_3_), 21.2 (CH_3_), 21.0 (CH_3_) and 17.7 (2 × CH_3_); HRMS (ESI-TOF) *m*/*z* [M + H]^+^ calcd for C_34_H_35_N_2_O_2_S^+^ 535.2414; found 535.2423.

#### 2,4-Di(4-tolyl)-1-naphth-2-yl-3-tosylaminopyrrole (**18f**)

Triazole **4a** (94 mg, 0.30 mmol, 1.0 equiv) and α-aminoketone **16f** (91 mg, 0.33 mmol, 1.1 equiv) were treated with Rh_2_(OAc)_4_ (7 mg, 0.02 mmol, 5 mol %) in PhMe (10 mL) according to General Procedure 3. During the N–H insertion, spontaneous cyclodehydration occurred to give the title compound **18f** (117 mg, 72%) as a yellow solid: mp 150 °C dec; ν_max_ (film) 3268, 3048, 2920, 2866, 1412, 1323, 1157, and 1092 cm^–1^; ^1^H NMR (400 MHz, 25.0 °C, CDCl_3_) δ 7.88–7.83 (1 H, m, naphthyl CH), 7.79 (1 H, d, *J* = 8.4 Hz, naphthyl CH), 7.62–7.58 (1 H, m, naphthyl CH), 7.55–7.45 (2 H, m, naphthyl CH), 7.40 (2 H, d, *J* = 8.1 Hz, Ar), 7.34 (1 H, dd, *J* = 8.3, 7.3 Hz, naphthyl CH), 7.28 (2 H, d, *J* = 8.4 Hz, Ar), 7.21 (1 H, dd, *J* = 7.3, 1.2 Hz, naphthyl CH), 7.10 (2 H, d, *J* = 7.6 Hz, Ar), 6.89 (1 H, s, pyrrole CH), 6.83 (2 H, d, *J* = 7.6 Hz, Ar), 6.73 (4 H, app s, Ar), 6.29 (1 H, s, NH), 2.36 (3 H, s, CH_3_), 2.27 (3 H, s, CH_3_) and 2.16 (3 H, s, CH_3_); ^13^C{^1^H} NMR (101 MHz, 25.0 °C, CDCl_3_) δ 142.5 (naphthyl C), 136.7 (Ar), 136.6 (Ar), 136.4 (Ar), 135.6 (Ar), 134.0 (naphthyl C), 133.8 (pyrrole), 131.0 (Ar), 130.8 (naphthyl C), 129.2 (2 × ArH), 129.0 (2 × ArH), 128.7 (2 × ArH), 128.5 (2 × ArH), 128.4 (naphthyl CH), 128.1 (naphthyl CH), 127.4 (2 × ArH), 127.2 (2 × ArH), 127.1 (Ar), 127.0 (naphthyl CH), 126.5 (naphthyl CH), 125.7 (naphthyl CH), 125.0 (naphthyl CH), 123.3 (pyrrole), 123.1 (naphthyl CH), 121.1 (pyrrole CH), 114.6 (pyrrole), 21.4 (CH_3_), 21.2 (CH_3_) and 21.1 (CH_3_); HRMS (ESI-TOF) *m*/*z* [M + H]^+^ calcd for C_35_H_31_N_2_O_2_S^+^ 543.2101; found 543.2109.

#### 5-Methyl-3-tosylamino-1,2,4-tri(4-tolyl)pyrrole (**18g**)

1,2-Diaminoalkene **17g** (54 mg, 0.10 mmol, 1.0 equiv) was treated with BF_3_·OEt_2_ (37 μL, 0.30 mmol, 3.0 equiv) in CH_2_Cl_2_ (3.3 mL) according to General Procedure 4 to give the title compound **18g** (38 mg, 73%) as a yellow solid. 4-Ethynyltoluene (38 μL, 0.33 mmol, 1.1 equiv) and 4-toluenesulfonyl azide (66 mg, 0.33 mmol, 1.1 equiv) were treated with CuTC (3 mg, 16 μmol, 5 mol %) in CH_2_Cl_2_ (10 mL), followed by α-aminoketone **16g** (76 mg, 0.30 mmol, 1.0 equiv) and Rh_2_(OAc)_4_ (1 mg, 2.3 μmol, 1 mol %), and finally BF_3_·OEt_2_ (111 μL, 0.90 mmol, 3.0 equiv) according to General Procedure 5 to give the title compound **18g** (106 mg, 68%): mp 154 °C dec; ν_max_ (film) 3271, 3028, 2920, 2866, 1512, 1381, 1323, 1157, and 1092 cm^–1^; ^1^H NMR (400 MHz, 25.0 °C, CDCl_3_) δ 7.19 (2 H, d, *J* = 8.3 Hz, Ar), 7.14–7.06 (6 H, m, Ar), 6.98 (2 H, d, *J* = 8.3 Hz, Ar), 6.86–6.84 (4 H, m, Ar), 6.81 (2 H, d, *J* = 7.9 Hz, Ar), 6.18 (1 H, s, NH), 2.37 (3 H, s, CH_3_), 2.32 (3 H, s, CH_3_), 2.29 (3 H, s, CH_3_), 2.25 (3 H, s, CH_3_) and 2.01 (3 H, s, CH_3_); ^13^C{^1^H} NMR (101 MHz, 25.0 °C, CDCl_3_) δ 142.0 (Ar), 137.2 (Ar), 137.2 (Ar), 136.0 (Ar), 135.9 (Ar), 135.2 (Ar), 131.4 (Ar), 131.0 (Ar), 129.8 (2 × ArH), 129.6 (2 × ArH), 129.4 (2 × ArH), 128.8 (2 × ArH), 128.6 (2 × ArH), 128.4 (2 × ArH), 128.3 (2 × ArH), 127.9 (pyrrole), 126.9 (2 × ArH), 125.9 (pyrrole), 120.6 (pyrrole), 114.6 (pyrrole), 21.4 (CH_3_), 21.2 (CH_3_), 21.2 (CH_3_), 21.1 (CH_3_) and 12.0 (CH_3_); HRMS (ESI-TOF) *m*/*z* [M + H]^+^ calcd for C_33_H_33_N_2_O_2_S^+^ 521.2257; found 521.2256.

#### 1,2-Di(4-tolyl)-4-(naphth-2-yl)-3-tosylaminopyrrole (**18h**)

1,2-Diaminoalkene **17h** (56 mg, 0.10 mmol, 1.0 equiv) was treated with BF_3_·OEt_2_ (37 μL, 0.30 mmol, 3.0 equiv) in CH_2_Cl_2_ (3.3 mL) according to General Procedure 4 to give the title compound **18h** (36 mg, 66%) as an orange solid. 4-Ethynyltoluene (38 μL, 0.33 mmol, 1.3 equiv) and 4-toluenesulfonyl azide (66 mg, 0.33 mmol, 1.3 equiv) were treated with CuTC (3 mg, 16 μmol, 6 mol %) in CH_2_Cl_2_ (10 mL), followed by α-aminoketone **16h** (72 mg, 0.26 mmol, 1.0 equiv) and Rh_2_(OAc)_4_ (1 mg, 2.3 μmol, 1 mol %), and finally BF_3_·OEt_2_ (111 μL, 0.90 mmol, 3.4 equiv) according to General Procedure 5 to give the title compound **18h** (142 mg, > 98%). On a larger scale, 4-ethynyltoluene (590 μL, 5.1 mmol, 1.1 equiv) and 4-toluenesulfonyl azide (1.29 g, 6.5 mmol, 1.4 equiv) were treated with CuTC (44 mg, 0.23 mmol, 5 mol %) in CH_2_Cl_2_ (170 mL), followed by α-aminoketone **16h** (1.27 g, 4.6 mmol, 1.0 equiv) and Rh_2_(OAc)_4_ (20 mg, 45 μmol, 1 mol %), and finally BF_3_·OEt_2_ (1.71 mL, 14 mmol, 3.0 equiv) according to General Procedure 5 to give the title compound (1.89 g, 76%) as an orange solid: mp 160 °C dec; ν_max_ (film) 3264, 3036, 2924, 1516, 1377, 1327, 1157, and 1092 cm^–1^; ^1^H NMR (400 MHz, 25.0 °C, CDCl_3_) δ 7.79–7.76 (2 H, m, Ar), 7.73–7.69 (1 H, m, Ar), 7.67 (1 H, d, *J* = 8.5 Hz, Ar), 7.52 (1 H, dd, *J* = 8.4, 1.7 Hz, Ar), 7.47–7.39 (2 H, m, Ar), 7.21 (2 H, d, *J* = 8.3 Hz, Ar), 7.08 (2 H, d, *J* = 7.9 Hz, Ar), 7.02–6.98 (6 H, m, Ar), 7.00 (1 H, s, pyrrole CH), 6.57 (2 H, d, *J* = 7.8 Hz, Ar), 6.27 (1 H, s, NH), 2.33 (3 H, s, CH_3_), 2.32 (3 H, s, CH_3_) and 1.98 (3 H, s, CH_3_); ^13^C{^1^H} NMR (101 MHz, 25.0 °C, CDCl_3_) δ 142.5 (Ar), 137.4 (Ar), 137.0 (Ar), 136.9 (Ar), 136.6 (Ar), 133.6 (naphthyl C), 132.7 (Ar), 132.0 (naphthyl C), 131.5 (naphthyl C), 130.0 (2 × ArH), 129.5 (2 × ArH), 128.8 (2 × ArH), 128.6 (2 × ArH), 127.9 (naphthyl CH), 127.7 (naphthyl CH), 127.5 (naphthyl CH), 127.2 (naphthyl CH), 126.9 (2 × ArH), 126.3 (naphthyl CH), 125.7 (pyrrole), 125.6 (naphthyl CH), 125.3 (2 × ArH), 125.2 (naphthyl CH), 123.4 (pyrrole), 119.9 (pyrrole CH), 115.5 (pyrrole), 21.3 (CH_3_), 21.1 (CH_3_) and 21.0 (CH_3_); HRMS (ESI-TOF) *m*/*z* [M + H]^+^ calcd for C_35_H_31_N_2_O_2_S^+^ 543.2101; found 543.2109.

#### 1,2-Di(4-tolyl)-4-(4-methoxyphenyl)-3-tosylaminopyrrole (**18i**)

1,2-Diaminoalkene **17i** (54 mg, 0.10 mmol, 1.0 equiv) was treated with BF_3_·OEt_2_ (37 μL, 0.30 mmol, 3.0 equiv) in CH_2_Cl_2_ (3.3 mL) according to General Procedure 4 to give the title compound **18i** (32 mg, 61%) as a yellow solid: mp 160 °C dec; ν_max_ (film) 3264, 3036, 2920, 2859, 1505, 1389, 1323, 1242, 1157, and 1092 cm^–1^; ^1^H NMR (400 MHz, 26.0 °C, CDCl_3_) δ 7.36 (2 H, d, *J* = 8.8 Hz, Ar), 7.24 (2 H, d, *J* = 8.4 Hz, Ar), 7.04 (2 H, d, *J* = 7.9 Hz, Ar), 6.94 (2 H, d, *J* = 8.2 Hz, Ar), 6.91 (2 H, d, *J* = 8.2 Hz, Ar), 6.84 (2 H, d, *J* = 7.9 Hz, Ar), 6.83 (1 H, s, pyrrole CH), 6.82 (2 H, d, *J* = 8.4 Hz, Ar), 6.80 (2 H, d, *J* = 8.8 Hz, Ar), 6.17 (1 H, s, NH), 3.82 (3 H, s, OCH_3_), 2.30 (3 H, s, CH_3_), 2.29 (3 H, s, CH_3_) and 2.28 (3 H, s, CH_3_); ^13^C{^1^H} NMR (101 MHz, 26.0 °C, CDCl_3_) δ 158.1 (Ar), 142.4 (Ar), 137.4 (Ar), 137.1 (Ar), 136.8 (Ar), 136.4 (Ar), 131.8 (Ar), 129.9 (2 × ArH), 129.5 (2 × ArH), 128.7 (2 × ArH), 128.7 (2 × ArH), 128.6 (2 × ArH), 127.3 (Ar), 127.1 (2 × ArH), 126.6 (pyrrole), 125.2 (2 × ArH), 123.3 (pyrrole), 119.1 (pyrrole CH), 115.3 (pyrrole), 113.7 (2 × ArH), 55.2 (OCH_3_), 21.4 (CH_3_), 21.3 (CH_3_) and 20.9 (CH_3_); HRMS (ESI-TOF) *m*/*z* [M + H]^+^ calcd for C_32_H_31_N_2_O_3_S^+^ 523.2050; found 523.2059.

#### 1,2-Di(4-tolyl)-4-(4-nitrophenyl)-3-tosylaminopyrrole (**18j**)

1,2-Diaminoalkene **17j** (56 mg, 0.10 mmol, 1.0 equiv) was treated with BF_3_·OEt_2_ (37 μL, 0.30 mmol, 3.0 equiv) in CH_2_Cl_2_ (3.3 mL) according to General Procedure 4 to give the title compound **18j** (42 mg, 78%) as an orange solid. 4-Ethynyltoluene (38 μL, 0.33 mmol, 1.1 equiv) and 4-toluenesulfonyl azide (66 mg, 0.33 mmol, 1.1 equiv) were treated with CuTC (3 mg, 16 μmol, 5 mol %) in CH_2_Cl_2_ (10 mL), followed by α-aminoketone **16j** (81 mg, 0.30 mmol, 1.0 equiv) and Rh_2_(OAc)_4_ (1 mg, 2.3 μmol, 1 mol %), and finally BF_3_·OEt_2_ (111 μL, 0.90 mmol, 3.0 equiv) according to General Procedure 5 to give the title compound **18j** (120 mg, 74%): mp 175 °C dec; ν_max_ (film) 3264, 3036, 2924, 1597, 1393, 1335, 1161, and 1151 cm^–1^; ^1^H NMR (400 MHz, 25.0 °C, CDCl_3_) δ 8.11 (2 H, d, *J* = 8.9 Hz, Ar), 7.71 (2 H, d, *J* = 8.9 Hz, Ar), 7.23 (2 H, d, *J* = 8.3 Hz, Ar), 7.07 (2 H, d, *J* = 8.1 Hz, Ar), 7.04 (1 H, s, pyrrole CH), 6.96–6.90 (4 H, m, Ar), 6.83 (2 H, d, *J* = 8.3 Hz, Ar), 6.74 (2 H, d, *J* = 8.1 Hz, Ar), 6.32 (1 H, s, NH), 2.31 (3 H, s, CH_3_), 2.30 (3 H, s, CH_3_) and 2.25 (3 H, s, CH_3_); ^13^C{^1^H} NMR (101 MHz, 25.0 °C, CDCl_3_) δ 145.6 (Ar), 143.1 (Ar), 141.4 (Ar), 137.4 (Ar), 137.2 (Ar), 136.8 (Ar), 136.5 (Ar), 132.9 (Ar), 129.7 (2 × ArH), 129.6 (2 × ArH), 128.9 (2 × ArH), 128.9 (2 × ArH), 127.4 (2 × ArH), 127.1 (2 × ArH), 126.3 (pyrrole), 125.1 (2 × ArH), 123.7 (2 × ArH), 121.3 (pyrrole), 120.8 (pyrrole CH), 115.4 (pyrrole), 21.3 (CH_3_), 21.3 (CH_3_) and 21.0 (CH_3_); HRMS (ESI-TOF) *m*/*z* [M + H]^+^ calcd for C_31_H_28_N_3_O_4_S^+^ 538.1795; found 538.1803.

#### 4-(4-Cyanophenyl)-1,2-di(4-tolyl)-3-tosylaminopyrrole (**18k**)

1,2-Diaminoalkene **17k** (54 mg, 0.10 mmol, 1.0 equiv) was treated with BF_3_·OEt_2_ (37 μL, 0.30 mmol, 3.0 equiv) in CH_2_Cl_2_ (3.3 mL) according to General Procedure 4 to give the title compound **18k** (37 mg, 71%) as a yellow solid: mp 165 °C dec; ν_max_ (film) 3275, 3036, 2924, 2226, 1605, 1543, 1516, 1393, 1327, and 1161 cm^–1^; ^1^H NMR (400 MHz, 25.0 °C, CDCl_3_) δ 7.65 (2 H, d, *J* = 8.7 Hz, Ar), 7.52 (2 H, d, *J* = 8.7 Hz, Ar), 7.22 (2 H, d, *J* = 8.1 Hz, Ar), 7.06 (2 H, d, *J* = 7.9 Hz, Ar), 6.99 (1 H, s, pyrrole CH), 6.95–6.90 (4 H, m, Ar), 6.84 (2 H, d, *J* = 8.1 Hz, Ar), 6.74 (2 H, d, *J* = 8.1 Hz, Ar), 6.27 (1 H, s, NH), 2.31 (3 H, s, CH_3_), 2.30 (3 H, s, CH_3_) and 2.30 (3 H, s, CH_3_); ^13^C{^1^H} NMR (101 MHz, 25.0 °C, CDCl_3_) δ 143.0 (Ar), 139.2 (Ar), 137.3 (Ar), 137.1 (Ar), 136.9 (Ar), 136.5 (Ar), 132.8 (Ar), 132.0 (2 × ArH), 129.7 (2 × ArH), 129.6 (2 × ArH), 128.9 (2 × ArH), 128.9 (2 × ArH), 127.5 (2 × ArH), 127.0 (2 × ArH), 126.4 (pyrrole), 125.1 (2 × ArH), 121.7 (pyrrole), 120.5 (pyrrole CH), 119.4 (pyrrole), 115.3 (C≡N), 109.0 (Ar), 21.4 (CH_3_), 21.3 (CH_3_) and 21.0 (CH_3_); HRMS (ESI-TOF) *m*/*z* [M + H]^+^ calcd for C_32_H_28_N_3_O_2_S^+^ 518.1897; found 518.1904.

#### 4-(4-Chlorophenyl)-1,2-di(4-tolyl)-3-tosylaminopyrrole (**18l**)

1,2-Diaminoalkene **17l** (55 mg, 0.10 mmol, 1.0 equiv) was treated with BF_3_·OEt_2_ (37 μL, 0.30 mmol, 3.0 equiv) in CH_2_Cl_2_ (3.3 mL) according to General Procedure 4 to give the title compound **18l** (41 mg, 77%) as a yellow solid: mp 160 °C dec; ν_max_ (film) 3268, 2924, 1501, 1389, 1157, and 1092 cm^–1^; ^1^H NMR (400 MHz, 26.0 °C, CDCl_3_) δ 7.36 (2 H, d, *J* = 8.5 Hz, Ar), 7.24 (2 H, d, *J* = 8.3 Hz, Ar), 7.18 (2 H, d, *J* = 8.5 Hz, Ar), 7.05 (2 H, d, *J* = 8.0 Hz, Ar), 6.95 (2 H, d, *J* = 8.1 Hz, Ar), 6.93 (2 H, d, *J* = 8.3 Hz, Ar), 6.88 (1 H, s, pyrrole CH), 6.85 (2 H, d, *J* = 8.1 Hz, Ar), 6.84 (2 H, d, *J* = 8.0 Hz, Ar), 6.18 (1 H, s, NH), 2.31 (6 H, s, 2 × CH_3_) and 2.30 (3 H, s, CH_3_); ^13^C{^1^H} NMR (101 MHz, 26.0 °C, CDCl_3_) δ 142.8 (Ar), 137.2 (Ar), 137.1 (Ar), 136.9 (Ar), 136.7 (Ar), 132.6 (Ar), 132.4 (Ar), 131.8 (Ar), 129.9 (2 × ArH), 129.5 (2 × ArH), 128.8 (2 × ArH), 128.8 (2 × ArH), 128.6 (2 × ArH), 128.3 (2 × ArH), 127.0 (2 × ArH), 126.9 (pyrrole), 125.2 (2 × ArH), 122.4 (pyrrole), 119.6 (pyrrole CH), 115.2 (pyrrole), 21.5 (CH_3_), 21.3 (CH_3_) and 21.0 (CH_3_); HRMS (ESI-TOF) *m*/*z* [M + H]^+^ calcd for C_31_H_28_ClN_2_O_2_S^+^ 527.1555; found 527.1559.

#### 1,4-Di(4-tolyl)-2-(thiophen-2-yl)-3-tosylaminopyrrole (**18m**)

1,2-Diaminoalkene **17m** (52 mg, 0.10 mmol, 1.0 equiv) was treated with BF_3_·OEt_2_ (37 μL, 0.30 mmol, 3.0 equiv) in CH_2_Cl_2_ (3.3 mL) according to General Procedure 4 to give the title compound **18m** (38 mg, 76%) as a yellow solid: mp 187 °C dec; ν_max_ (film) 3268, 2920, 1516, 1397, 1327, 1161, and 1092 cm^–1^; ^1^H NMR (400 MHz, 25.0 °C, CDCl_3_) δ 7.32 (2 H, d, *J* = 8.3 Hz, Ar), 7.24 (2 H, d, *J* = 8.1 Hz, Ar), 7.18 (1 H, dd, *J* = 5.1, 1.2 Hz, thiophene CH), 7.12 (2 H, d, *J* = 8.2 Hz, Ar), 7.05 (2 H, d, *J* = 8.2 Hz, Ar), 7.03 (2 H, d, *J* = 8.1 Hz, Ar), 6.86 (2 H, d, *J* = 8.3 Hz, Ar), 6.85 (1 H, s, pyrrole CH), 6.82 (1 H, dd, *J* = 5.1, 3.6 Hz, thiophene CH), 6.66 (1 H, dd, *J* = 3.6, 1.2 Hz, thiophene CH), 6.32 (1 H, s, NH), 2.35 (3 H, s, CH_3_), 2.34 (3 H, s, CH_3_) and 2.28 (3 H, s, CH_3_); ^13^C{^1^H} NMR (101 MHz, 25.0 °C, CDCl_3_) δ 142.5 (Ar), 137.3 (Ar), 137.1 (Ar), 136.9 (Ar), 135.6 (Ar), 130.7 (Ar), 130.7 (thiophene C2), 129.5 (2 × ArH), 128.9 (2 × ArH), 128.8 (2 × ArH), 128.4 (thiophene CH), 127.3 (2 × ArH), 127.1 (2 × ArH), 126.7 (thiophene CH), 126.4 (thiophene CH), 125.7 (2 × ArH), 125.6 (pyrrole), 123.6 (pyrrole), 120.4 (pyrrole CH), 116.5 (pyrrole), 21.4 (CH_3_), 21.2 (CH_3_) and 21.0 (CH_3_); HRMS (ESI-TOF) *m*/*z* [M + H]^+^ calcd for C_29_H_27_N_2_O_2_S_2_^+^ 499.1508; found 499.1517.

#### 1,4-Di(4-tolyl)-2-(4-methoxyphenyl)-3-tosylaminopyrrole (**18n**)

1,2-Diaminoalkene **17n** (54 mg, 0.10 mmol, 1.0 equiv) was treated with BF_3_·OEt_2_ (37 μL, 0.30 mmol, 3.0 equiv) in CH_2_Cl_2_ (3.3 mL) according to General Procedure 4 to give the title compound **18n** (44 mg, 84%) as a yellow solid: mp 75 °C dec; ν_max_ (film) 3275, 2924, 1516, 1393, 1323, 1250, 1157, and 1092 cm^–1^; ^1^H NMR (400 MHz, 25.0 °C, CDCl_3_) δ 7.31 (2 H, d, *J* = 8.1 Hz, Ar), 7.24 (2 H, d, *J* = 8.3 Hz, Ar), 7.06 (2 H, d, *J* = 8.1 Hz, Ar), 7.05 (2 H, d, *J* = 8.3 Hz, Ar), 6.94 (2 H, d, *J* = 8.4 Hz, Ar), 6.90 (2 H, d, *J* = 8.8 Hz, Ar), 6.85 (1 H, s, pyrrole CH), 6.82 (2 H, d, *J* = 8.4 Hz, Ar), 6.65 (2 H, d, *J* = 8.8 Hz, Ar), 6.15 (1 H, s, NH), 3.78 (3 H, s, OCH_3_), 2.34 (3 H, s, CH_3_), 2.31 (3 H, s, CH_3_) and 2.27 (3 H, s, CH_3_); ^13^C{^1^H} NMR (101 MHz, 25.0 °C, CDCl_3_) δ 158.7 (Ar), 142.4 (Ar), 137.4 (Ar), 137.1 (Ar), 136.5 (Ar), 135.5 (Ar), 131.8 (Ar), 131.3 (2 × ArH), 131.1 (Ar), 129.5 (2 × ArH), 129.0 (2 × ArH), 128.7 (2 × ArH), 127.4 (2 × ArH), 127.1 (2 × ArH), 125.3 (2 × ArH), 123.5 (pyrrole), 122.7 (pyrrole), 119.2 (pyrrole CH), 115.2 (pyrrole), 113.5 (2 × ArH), 55.1 (OCH_3_), 21.4 (CH_3_), 21.2 (CH_3_) and 21.0 (CH_3_); HRMS (ESI-TOF) *m*/*z* [M + H]^+^ calcd for C_32_H_31_N_2_O_3_S^+^ 523.2050; found 523.2057.

#### 1,4-Di(4-tolyl)-2-(cyclohexen-1-yl)-3-tosylaminopyrrole (**18o**)

1,2-Diaminoalkene **17o** (51 mg, 0.10 mmol, 1.0 equiv) was treated with BF_3_·OEt_2_ (37 μL, 0.30 mmol, 3.0 equiv) in CH_2_Cl_2_ (3.3 mL) according to General Procedure 4 to give the title compound **18o** (28 mg, 57%) as a brown solid: mp 80 °C dec; ν_max_ (film) 3271, 3036, 2928, 1709, 1593, 1516, 1492, 1393, 1246, 1157, and 1092 cm^–1^; ^1^H NMR (400 MHz, 25.0 °C, CDCl_3_) δ 7.41 (2 H, d, *J* = 8.3 Hz, Ar), 7.22 (2 H, d, *J* = 8.5 Hz, Ar), 7.19 (2 H, d, *J* = 8.5 Hz, Ar), 7.17 (2 H, d, *J* = 8.3 Hz, Ar), 7.00 (2 H, d, *J* = 7.9 Hz, Ar), 6.97 (2 H, d, *J* = 7.9 Hz, Ar), 6.69 (1 H, s, pyrrole CH), 6.15 (1 H, s, NH), 5.80–5.76 (1 H, m, =CH), 2.37 (3 H, s, CH_3_), 2.32 (3 H, s, CH_3_), 2.30 (3 H, s, CH_3_), 2.10–2.03 (2 H, m, CH_2_), 1.57–1.52 (2 H, m, CH_2_), 1.48–1.42 (2 H, m, CH_2_) and 1.39–1.32 (2 H, m, CH_2_); ^13^C{^1^H} NMR (101 MHz, 25.0 °C, CDCl_3_) δ 142.6 (Ar), 138.1 (Ar), 137.2 (Ar), 136.5 (Ar), 135.2 (Ar), 134.5 (pyrrole), 131.5 (=CH), 131.2 (Ar), 129.6 (2 × ArH), 128.9 (2 × ArH), 128.8 (2 × ArH), 128.7 (=C), 127.4 (2 × ArH), 127.2 (2 × ArH), 124.0 (2 × ArH), 123.0 (pyrrole), 118.1 (pyrrole CH), 114.4 (pyrrole), 28.2 (CH_2_), 25.7 (CH_2_), 22.4 (CH_2_), 21.5 (CH_2_), 21.4 (CH_3_), 21.1 (CH_3_) and 21.0 (CH_3_); HRMS (ESI-TOF) *m*/*z* [M + H]^+^ calcd for C_31_H_33_N_2_O_2_S^+^ 497.2257; found 497.2265.

#### 3-(4-Methoxybenzenesulfonylamino)-1,2,4-tri(4-tolyl)pyrrole (**18p**)

1,2-Diaminoalkene **17p** (54 mg, 0.10 mmol, 1.0 equiv) was treated with BF_3_·OEt_2_ (37 μL, 0.30 mmol, 3.0 equiv) in CH_2_Cl_2_ (3.3 mL) according to General Procedure 4 to give the title compound **18p** (26 mg, 50%) as a yellow solid. 4-Ethynyltoluene (38 μL, 0.33 mmol, 1.1 equiv) and 4-methoxybenzenesulfonyl azide (70 mg, 0.33 mmol, 1.1 equiv) were treated with CuTC (3 mg, 16 μmol, 5 mol %) in CH_2_Cl_2_ (10 mL), followed by α-aminoketone **16a** (72 mg, 0.30 mmol, 1.0 equiv) and Rh_2_(OAc)_4_ (1 mg, 2.3 μmol, 1 mol %), and finally BF_3_·OEt_2_ (111 μL, 0.90 mmol, 3.0 equiv) according to General Procedure 5 to give the title compound **18p** (88 mg, 56%): mp 150 °C dec; ν_max_ (film) 3271, 3020, 2920, 1593, 1501, 1389, 1323, 1258, 1153, and 1096 cm^–1^; ^1^H NMR (400 MHz, 25.0 °C, CDCl_3_) δ 7.33 (2 H, d, *J* = 8.2 Hz, Ar), 7.27 (2 H, d, *J* = 8.9 Hz, Ar), 7.06 (2 H, d, *J* = 7.7 Hz, Ar), 7.05 (2 H, d, *J* = 7.7 Hz, Ar), 6.95 (2 H, d, *J* = 8.9 Hz, Ar), 6.93 (2 H, d, *J* = 8.2 Hz, Ar), 6.87 (2 H, d, *J* = 9.1 Hz, Ar), 6.86 (1 H, s, pyrrole CH), 6.47 (2 H, d, *J* = 9.1 Hz, Ar), 6.17 (1 H, s, NH), 3.76 (3 H, s, OCH_3_), 2.34 (3 H, s, CH_3_), 2.31 (3 H, s, CH_3_) and 2.29 (3 H, s, CH_3_); ^13^C{^1^H} NMR (101 MHz, 25.0 °C, CDCl_3_) δ 162.3 (Ar), 137.4 (Ar), 136.7 (Ar), 136.5 (Ar), 135.5 (Ar), 131.9 (Ar), 131.6 (Ar), 131.1 (Ar), 129.9 (2 × ArH), 129.5 (2 × ArH), 129.1 (2 × ArH), 129.0 (2 × ArH), 128.8 (2 × ArH), 127.4 (2 × ArH), 127.3 (pyrrole), 125.2 (2 × ArH), 123.5 (pyrrole), 119.4 (pyrrole CH), 115.4 (pyrrole), 113.2 (2 × ArH), 55.3 (OCH_3_), 21.2 (CH_3_), 21.1 (CH_3_) and 21.0 (CH_3_); HRMS (ESI-TOF) *m*/*z* [M + H]^+^ calcd for C_32_H_31_N_2_O_3_S^+^ 523.2050; found 523.2058.

#### 3-(4-Nitrobenzenesulfonylamino)-1,2,4-tri(4-tolyl)pyrrole (**18q**)

1,2-Diaminoalkene **17q** (56 mg, 0.10 mmol, 1.0 equiv) was treated with BF_3_·OEt_2_ (37 μL, 0.30 mmol, 3.0 equiv) in CH_2_Cl_2_ (3.3 mL) according to General Procedure 4 to give the title compound **18q** (45 mg, 83%) as an orange solid: mp 180 °C dec; ν_max_ (film) 3271, 3036, 2920, 2862, 1516, 1404, 1389, 1346, 1161, and 1092 cm^–1^; ^1^H NMR (400 MHz, 25.0 °C, CDCl_3_) δ 7.80 (2 H, d, *J* = 8.8 Hz, Ar), 7.53 (2 H, d, *J* = 8.8 Hz, Ar), 7.26 (2 H, d, *J* = 8.1 Hz, Ar), 7.06 (2 H, d, *J* = 7.6 Hz, Ar), 7.05 (2 H, d, *J* = 7.6 Hz, Ar), 6.94 (2 H, d, *J* = 8.3 Hz, Ar), 6.93 (2 H, d, *J* = 7.8 Hz, Ar), 6.88 (2 H, d, *J* = 8.3 Hz, Ar), 6.87 (1 H, s, pyrrole CH), 6.53 (1 H, s, NH), 2.32 (3 H, s, CH_3_), 2.31 (3 H, s, CH_3_) and 2.29 (3 H, s, CH_3_); ^13^C{^1^H} NMR (101 MHz, 25.0 °C, CDCl_3_) δ 149.3 (Ar), 145.7 (Ar), 137.4 (Ar), 137.1 (Ar), 136.8 (Ar), 136.2 (Ar), 132.6 (Ar), 130.7 (Ar), 129.9 (2 × ArH), 129.6 (2 × ArH), 129.1 (2 × ArH), 128.9 (2 × ArH), 128.2 (2 × ArH), 127.4 (2 × ArH), 127.0 (pyrrole), 125.2 (2 × ArH), 123.7 (pyrrole), 123.2 (2 × ArH), 119.7 (pyrrole CH), 114.0 (pyrrole), 21.2 (CH_3_), 21.0 (CH_3_) and 21.0 (CH_3_); HRMS (ESI-TOF) *m*/*z* [M + H]^+^ calcd for C_31_H_28_N_3_O_4_S^+^ 538.1795; found 538.1805.

#### 3-Mesylamino-1,2,4-tri(4-tolyl)pyrrole (**18r**)

1,2-Diaminoalkene **17r** (45 mg, 0.10 mmol, 1.0 equiv) was treated with BF_3_·OEt_2_ (37 μL, 0.30 mmol, 3.0 equiv) in CH_2_Cl_2_ (3.3 mL) according to General Procedure 4 to give the title compound **18r** (30 mg, 69%) as an orange solid: mp 93–94 °C; ν_max_ (film) 3268, 2924, 1516, 1393, 1319, and 1153 cm^–1^; ^1^H NMR (400 MHz, 25.0 °C, CDCl_3_) δ 7.51 (2 H, d, *J* = 8.1 Hz, Ar), 7.22 (2 H, d, *J* = 7.8 Hz, Ar), 7.15 (2 H, d, *J* = 8.4 Hz, Ar), 7.10 (2 H, d, *J* = 8.1 Hz, Ar), 7.09 (2 H, d, *J* = 8.1 Hz, Ar), 7.02 (2 H, d, *J* = 8.4 Hz, Ar), 6.96 (1 H, s, pyrrole CH), 5.92 (1 H, s, NH), 2.37 (3 H, s, CH_3_), 2.33 (3 H, s, CH_3_), 2.32 (3 H, s, CH_3_) and 2.29 (3 H, s, CH_3_); ^13^C{^1^H} NMR (101 MHz, 25.0 °C, CDCl_3_) δ 137.5 (Ar), 137.4 (Ar), 136.7 (Ar), 136.3 (Ar), 132.1 (Ar), 131.0 (Ar), 130.2 (2 × ArH), 129.6 (2 × ArH), 129.5 (2 × ArH), 129.2 (2 × ArH), 127.9 (2 × ArH), 127.5 (pyrrole), 125.3 (2 × ArH), 123.7 (pyrrole), 119.5 (pyrrole CH), 115.3 (pyrrole), 40.7 (Ms), 21.3 (CH_3_), 21.2 (CH_3_) and 21.0 (CH_3_); HRMS (ESI-TOF) *m*/*z* [M + H]^+^ calcd for C_26_H_27_N_2_O_2_S^+^ 431.1788; found 431.1797.

#### 4-(4-Cyanophenyl)-2-(4-methoxyphenyl)-1-(4-tolyl)-3-tosylaminopyrrole (**18s**)

1-Ethynyl-4-methoxybenzene (43 μL, 0.32 mmol, 1.2 equiv) and 4-toluenesulfonyl azide (66 mg, 0.33 mmol, 1.2 equiv) were treated with CuTC (3 mg, 16 μmol, 6 mol %) in CH_2_Cl_2_ (10 mL), followed by α-aminoketone **16e** (75 mg, 0.28 mmol, 1.0 equiv) and Rh_2_(OAc)_4_ (1 mg, 2.3 μmol, 1 mol %), and finally BF_3_·OEt_2_ (111 μL, 0.90 mmol, 3.2 equiv) according to General Procedure 5 to give the title compound **18s** (127 mg, 85%) as an orange solid: mp 140 °C dec; ν_max_ (film) 3268, 2924, 2226, 1609, 1566, 1516, 1327, 1292, 1250, 1161, 1092, and 1034 cm^–1^; ^1^H NMR (400 MHz, 25.0 °C, CDCl_3_) δ 7.64 (2 H, d, *J* = 8.7 Hz, Ar), 7.52 (2 H, d, *J* = 8.7 Hz, Ar), 7.25 (2 H, d, *J* = 8.2 Hz, Ar), 7.06 (2 H, d, *J* = 7.9 Hz, Ar), 6.98 (1 H, s, pyrrole CH), 6.93 (2 H, d, *J* = 8.2 Hz, Ar), 6.86 (2 H, d, *J* = 7.9 Hz, Ar), 6.80 (2 H, d, *J* = 8.8 Hz, Ar), 6.65 (2 H, d, *J* = 8.8 Hz, Ar), 6.25 (1 H, s, NH), 3.78 (3 H, s, CH_3_), 2.31 (3 H, s, CH_3_) and 2.31 (3 H, s, CH_3_); ^13^C{^1^H} NMR (101 MHz, 25.0 °C, CDCl_3_) δ 159.0 (Ar), 143.0 (Ar), 139.2 (Ar), 137.1 (Ar), 136.9 (Ar), 136.7 (Ar), 132.7 (Ar), 132.0 (2 × ArH), 131.1 (2 × ArH), 129.7 (2 × ArH), 128.9 (2 × ArH), 127.5 (2 × ArH), 127.1 (2 × ArH), 125.2 (2 × ArH), 121.7 (pyrrole), 121.6 (pyrrole), 120.2 (pyrrole CH), 119.4 (pyrrole), 115.1 (C≡N), 113.7 (2 × ArH), 109.0 (Ar), 55.1 (OCH_3_), 21.4 (CH_3_) and 21.0 (CH_3_); HRMS (ESI-TOF) *m*/*z* [M + H]^+^ calcd for C_32_H_28_N_3_O_3_S^+^ 534.1846; found 534.1851.

#### 1,4-Di(4-tolyl)-2-(4-methoxyphenyl)-5-methyl-3-tosylaminopyrrole (**18t**)

1-Ethynyl-4-methoxybenzene (43 μL, 0.32 mmol, 1.1 equiv) and 4-toluenesulfonyl azide (66 mg, 0.33 mmol, 1.1 equiv) were treated with CuTC (3 mg, 16 μmol, 5 mol %) in CH_2_Cl_2_ (10 mL), followed by α-aminoketone **16g** (76 mg, 0.30 mmol, 1.0 equiv) and Rh_2_(OAc)_4_ (1 mg, 2.3 μmol, 1 mol %), and finally BF_3_·OEt_2_ (111 μL, 0.90 mmol, 3.0 equiv) according to General Procedure 5 to give the title compound **18t** (118 mg, 73%) as an orange solid: mp 70 °C dec; ν_max_ (film) 3275, 2920, 2851, 1512, 1462, 1381, 1323, 1246, 1157, 1092, and 1030 cm^–1^; ^1^H NMR (400 MHz, 25.0 °C, CDCl_3_) δ 7.20 (2 H, d, *J* = 7.4 Hz, Ar), 7.13–7.05 (6 H, m, Ar), 6.96 (2 H, d, *J* = 8.1 Hz, Ar), 6.88 (2 H, d, *J* = 8.6 Hz, Ar), 6.82 (2 H, d, *J* = 8.1 Hz, Ar), 6.59 (2 H, d, *J* = 8.6 Hz, Ar), 6.03 (1 H, s, NH), 3.74 (3 H, s, OCH_3_), 2.36 (3 H, s, CH_3_), 2.32 (3 H, s, CH_3_), 2.29 (3 H, s, CH_3_) and 2.01 (3 H, s, CH_3_); ^13^C{^1^H} NMR (101 MHz, 25.0 °C, CDCl_3_) δ 158.2 (Ar), 142.1 (Ar), 137.3 (Ar), 137.2 (Ar), 135.9 (Ar), 135.3 (Ar), 131.4 (Ar), 131.2 (2 × ArH), 130.8 (Ar), 129.5 (2 × ArH), 129.4 (2 × ArH), 128.8 (2 × ArH), 128.6 (2 × ArH), 128.3 (2 × ArH), 127.0 (2 × ArH), 125.7 (pyrrole), 123.4 (pyrrole), 120.4 (pyrrole), 114.5 (pyrrole), 113.2 (2 × ArH), 55.0 (CH_3_), 21.4 (CH_3_), 21.2 (CH_3_), 21.1 (CH_3_) and 11.9 (CH_3_); HRMS (ESI-TOF) *m*/*z* [M + H]^+^ calcd for C_33_H_33_N_2_O_3_S^+^ 537.2206; found 537.2207.

#### 4-Cyclopropyl-1,2-di(4-tolyl)-3-tosylaminopyrrole (**18u**)

4-Ethynyltoluene (43 μL, 0.37 mmol, 1.2 equiv) and 4-toluenesulfonyl azide (66 mg, 0.33 mmol, 1.1 equiv) were treated with CuTC (3 mg, 16 μmol, 5 mol %) in CH_2_Cl_2_ (10 mL), followed by α-aminoketone **16u** (57 mg, 0.30 mmol, 1.0 equiv) and Rh_2_(OAc)_4_ (1 mg, 2.3 μmol, 1 mol %), and finally BF_3_·OEt_2_ (111 μL, 0.90 mmol, 3.0 equiv) according to General Procedure 5 to give the title compound **18u** (70 mg, 51%) as a yellow solid: mp 66 °C dec; ν_max_ (film) 3271, 3005, 2924, 2862, 1516, 1323, and 1161 cm^–1^; ^1^H NMR (400 MHz, 25.0 °C, CDCl_3_) δ 7.37 (2 H, d, *J* = 8.3 Hz, Ar), 6.98 (2 H, d, *J* = 7.8 Hz, Ar), 6.91 (2 H, d, *J* = 8.3 Hz, Ar), 6.82 (2 H, d, *J* = 8.1 Hz, Ar), 6.81 (2 H, d, *J* = 7.8 Hz, Ar), 6.59 (2 H, d, *J* = 8.1 Hz, Ar), 6.39 (1 H, d, *J* = 0.8 Hz, pyrrole CH), 6.14 (1 H, s, NH), 2.30 (3 H, s, CH_3_), 2.27 (3 H, s, CH_3_), 2.26 (3 H, s, CH_3_), 1.85 (1 H, ttd, *J* = 8.3, 5.2, 0.8 Hz, cyclopropane CH), 0.82 (2 H, ddd, *J* = 8.3, 6.1, 4.0 Hz, 2 × cyclopropane CH) and 0.51 (2 H, ddd, *J* = 6.1, 5.2, 4.0 Hz, 2 × cyclopropane CH); ^13^C{^1^H} NMR (101 MHz, 25.0 °C, CDCl_3_) δ 142.5 (Ar), 137.6 (Ar), 136.8 (Ar), 136.4 (Ar), 136.0 (Ar), 130.2 (Ar), 129.5 (2 × ArH), 129.3 (2 × ArH), 128.8 (2 × ArH), 128.5 (2 × ArH), 127.3 (pyrrole), 127.2 (2 × ArH), 126.3 (pyrrole), 125.1 (2 × ArH), 117.6 (pyrrole), 116.9 (pyrrole CH), 21.5 (CH_3_), 21.2 (CH_3_), 20.9 (CH_3_), 7.6 (2 × cyclopropane CH_2_) and 5.7 (cyclopropane); HRMS (ESI-TOF) *m*/*z* [M + H]^+^ calcd for C_28_H_29_N_2_O_2_S^+^ 457.1944; found 457.1943.

#### 1,2-Di(4-tolyl)-4-(2-tolyl)-3-tosylaminopyrrole (**18v**)

4-Ethynyltoluene (43 μL, 0.37 mmol, 1.2 equiv) and 4-toluenesulfonyl azide (66 mg, 0.33 mmol, 1.1 equiv) were treated with CuTC (3 mg, 16 μmol, 5 mol %) in CH_2_Cl_2_ (10 mL), followed by α-aminoketone **16v** (72 mg, 0.30 mmol, 1.0 equiv) and Rh_2_(OAc)_4_ (1 mg, 2.3 μmol, 1 mol %), and finally BF_3_·OEt_2_ (111 μL, 0.90 mmol, 3.0 equiv) according to General Procedure 5 to give the title compound **18v** (82 mg, 54%) as a yellow solid: mp 80 °C dec; ν_max_ (film) 3271, 3063, 2924, 1516, 1389, 1327, and 1161 cm^–1^; ^1^H NMR (400 MHz, 25.0 °C, CDCl_3_) δ 7.19 (2 H, d, *J* = 8.3 Hz, Ar), 7.15–7.04 (6 H, m, Ar), 7.01–6.98 (4 H, m, Ar), 6.97 (2 H, d, *J* = 8.3 Hz, Ar), 6.82 (2 H, d, *J* = 7.8 Hz, Ar), 6.69 (1 H, s, pyrrole CH), 6.08 (1 H, s, NH), 2.31 (6 H, s, 2 × CH_3_), 2.29 (3 H, s, CH_3_) and 2.23 (3 H, s, CH_3_); ^13^C{^1^H} NMR (101 MHz, 25.0 °C, CDCl_3_) δ 142.2 (Ar), 137.5 (2 × Ar), 136.8 (Ar), 136.4 (Ar), 136.2 (Ar), 133.3 (Ar), 131.0 (pyrrole), 130.5 (ArH), 130.1 (2 × ArH), 129.9 (ArH), 129.5 (2 × ArH), 128.8 (2 × ArH), 128.8 (2 × ArH), 127.4 (Ar), 126.8 (2 × ArH), 126.5 (ArH), 125.4 (ArH), 125.2 (2 × ArH), 122.6 (pyrrole), 120.3 (pyrrole CH), 116.3 (pyrrole), 21.4 (CH_3_), 21.3 (CH_3_), 20.9 (CH_3_) and 20.7 (CH_3_); HRMS (ESI-TOF) *m*/*z* [M + H]^+^ calcd for C_32_H_31_N_2_O_2_S^+^ 507.2101; found 507.2111.

#### 1,4-Di(4-tolyl)-2-(4-fluorophenyl)-3-tosylaminopyrrole (**18w**)

1-Ethynyl-4-fluorobenzene (40 mg, 0.33 mmol, 1.1 equiv) and 4-toluenesulfonyl azide (66 mg, 0.33 mmol, 1.1 equiv) were treated with CuTC (3 mg, 16 μmol, 5 mol %) in CH_2_Cl_2_ (10 mL), followed by α-aminoketone **16a** (72 mg, 0.30 mmol, 1.0 equiv) and Rh_2_(OAc)_4_ (1 mg, 2.3 μmol, 1 mol %), and finally BF_3_·OEt_2_ (111 μL, 0.90 mmol, 3.0 equiv) according to General Procedure 5 to give the title compound **18w** (134 mg, 87%) as a yellow solid: mp 90 °C dec; ν_max_ (film) 3275, 3024, 2920, 2866, 1510, 1505, 1412, 1389, 1323, 1223, 1157, and 1092 cm^–1^; ^1^H NMR (400 MHz, 25.0 °C, CDCl_3_) δ 7.29–7.23 (4 H, m, ArH), 7.08–7.04 (2 H, m, ArH), 7.06 (2 H, d, *J* = 7.9 Hz, ArH), 6.99 (2 H, dd, *J* = 8.8, 5.4 Hz, C_6_H_4_F), 6.93 (2 H, d, *J* = 8.3 Hz, Ar), 6.86 (1 H, s, pyrrole CH), 6.83 (2 H, d, *J* = 7.9 Hz, ArH), 6.81 (2 H, dd, *J* = 8.8, 8.8 Hz, C_6_H_4_F), 6.21 (1 H, app d, *J* = 8.5 Hz, NH), 2.35 (3 H, s, CH_3_), 2.32 (3 H, s, CH_3_) and 2.28 (3 H, s, CH_3_); ^13^C{^1^H} NMR (101 MHz, 25.0 °C, CDCl_3_) δ 161.9 (d, *J* = 247.2 Hz, C_6_H_4_F), 142.6 (Ar), 137.1 (Ar), 137.1 (Ar), 136.8 (Ar), 135.7 (Ar), 131.9 (d, *J* = 8.2 Hz, 2 × C_6_H_4_F), 131.0 (pyrrole), 130.8 (Ar), 129.6 (2 × ArH), 129.0 (2 × ArH), 128.8 (2 × ArH), 127.4 (2 × ArH), 127.0 (2 × ArH), 126.4 (d, *J* = 3.4 Hz, C_6_H_4_F), 125.3 (2 × ArH), 123.7 (pyrrole), 119.6 (pyrrole CH), 115.6 (pyrrole), 115.0 (d, *J* = 21.6 Hz, 2 × C_6_H_4_F), 21.4 (CH_3_), 21.2 (CH_3_) and 20.9 (CH_3_); HRMS (ESI-TOF) *m*/*z* [M + H]^+^ calcd for C_31_H_28_FN_2_O_2_S^+^ 511.1850; found 511.1856.

#### 3-Amino-1,2,4-tri(4-tolyl)pyrrole (**23a**)

Triflic acid (16 μL, 0.18 mmol, 3.0 equiv) was added to a solution of pyrrole **18a** (30 mg, 0.059 mmol, 1.0 equiv) in 1,2-dichloroethane (2.0 mL) at 0 °C (ice bath). The mixture was heated at 90 °C (heating block) in a sealed vial for 2.5 h. The reaction mixture was cooled to ambient temperature, and the reaction was quenched by the addition of ethylenediamine (2 drops) followed by 1 M aqueous NaOH (2.0 mL). The aqueous phase was extracted with dichloromethane (3 × 2.0 mL), and the combined organic layers were washed with brine, dried (MgSO_4_), and concentrated in vacuo. Purification by flash column chromatography (SiO_2_, 5% hexane in ethyl acetate) gave the title compound **23a** (16 mg, 77%) as a yellow oil: ν_max_ (film) 3402, 3322, 3028, 2920, 2859, 1516, and 1389 cm^–1^; ^1^H NMR (400 MHz, 25.0 °C, CDCl_3_) δ 7.52 (2 H, d, *J* = 8.1 Hz, Ar), 7.23 (2 H, d, *J* = 7.7 Hz, Ar), 7.12–7.00 (8 H, m, Ar), 6.85 (1 H, s, pyrrole CH), 3.35 (2 H, br s, NH), 2.38 (3 H, s, 2 × CH_3_) and 2.32 (6 H, s, CH_3_); ^13^C{^1^H} NMR (101 MHz, 25.0 °C, CDCl_3_) δ 138.1 (Ar), 135.4 (Ar), 135.4 (Ar), 135.3 (Ar), 131.9 (pyrrole), 129.5 (2 × ArH), 129.5 (2 × ArH), 129.2 (3 × ArH and Ar), 128.9 (2 × ArH), 128.6 (pyrrole), 127.1 (2 × ArH), 124.5 (2 × ArH), 119.2 (pyrrole CH), 117.9 (Ar), 116.9 (pyrrole), 21.2 (CH_3_), 21.1 (CH_3_) and 20.9 (CH_3_); HRMS (ESI-TOF) *m*/*z* [M + H]^+^ calcd for C_25_H_25_N_2_^+^ 353.2012; found 353.2014.

## Supporting Information Available

High-resolution ^1^H and ^13^C NMR spectra of all products and selected NOESY spectra (PDF)
