# Supplementary material for: Modular Synthesis of Highly Substituted 3-Azapyrroles by Rh(II)-Catalyzed N–H Bond Insertion and Cyclodehydration
Source: J Org Chem. 2022 May 3;87(24):16139–56. doi: 10.1021/acs.joc.2c00434 (PMC9764362; doi:10.1021/acs.joc.2c00434)

Modular Synthesis of Highly Substituted 3-Azapyrroles by Rh(II) Catalyzed N–H Bond Insertion and Cyclodehydration

Supporting Information

Matthew B. Williams, Alistair Boyer

NMR Spectra

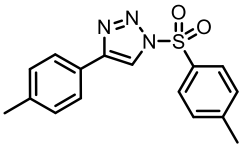

4a

^1H, $CDCl_3$, 400 MHz

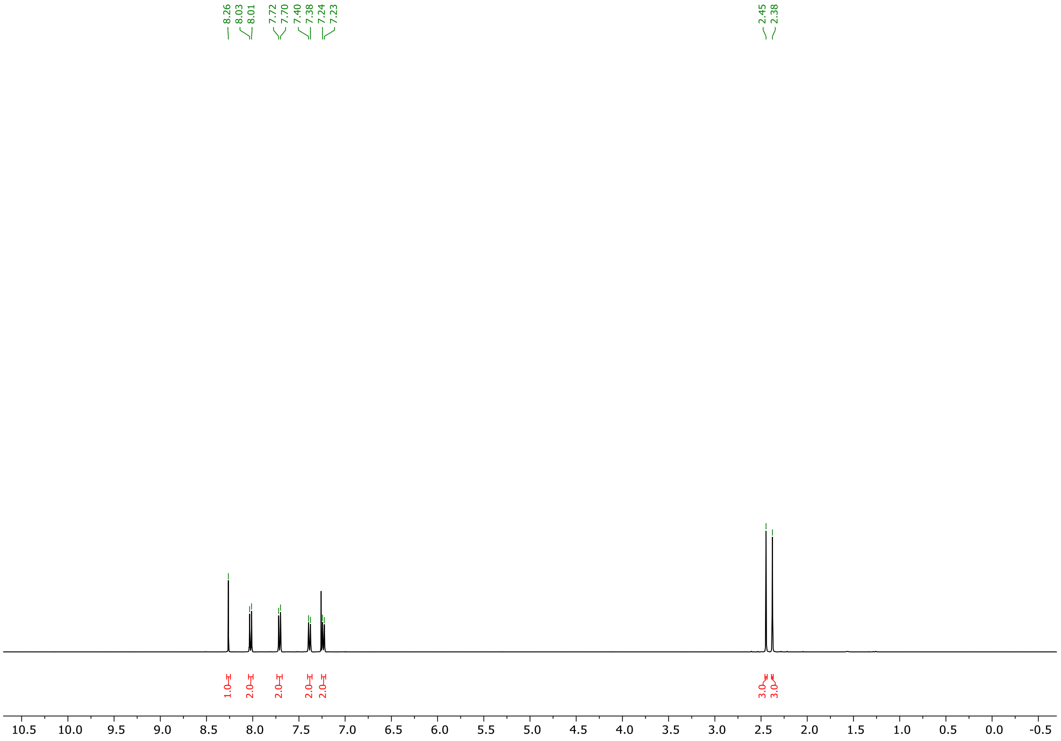

$^{13}C\{^1H\}$, $CDCl_3$, 101 MHz

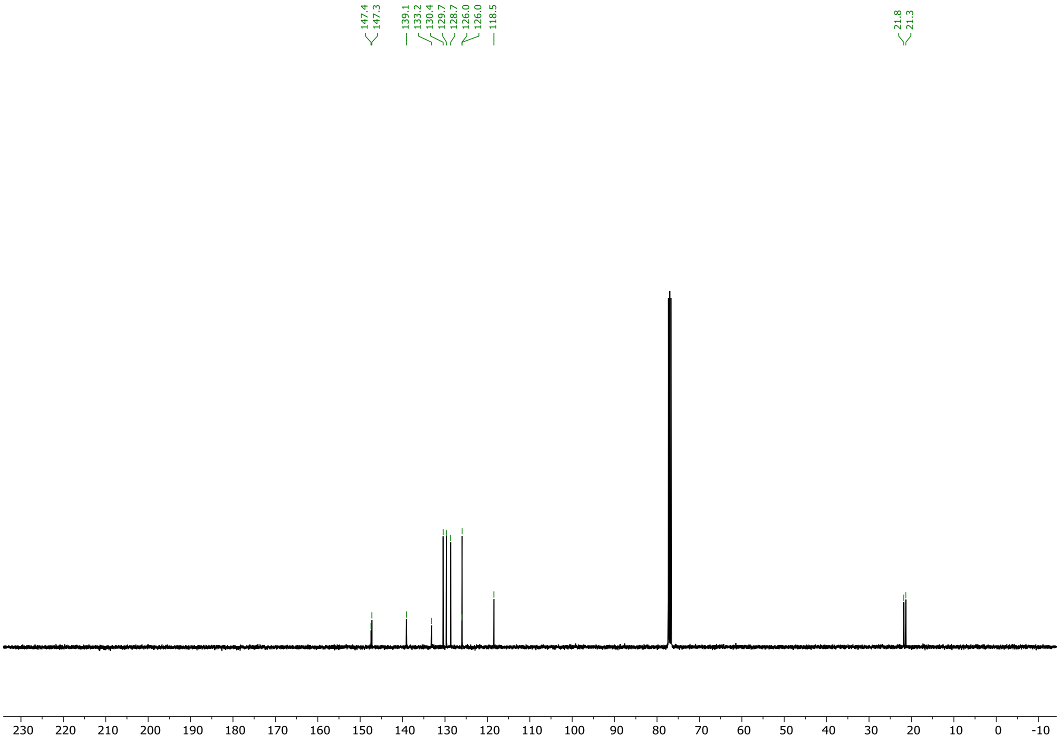

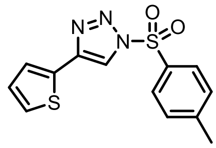

4m

^{1}H, CDCl$_3$, 400 MHz

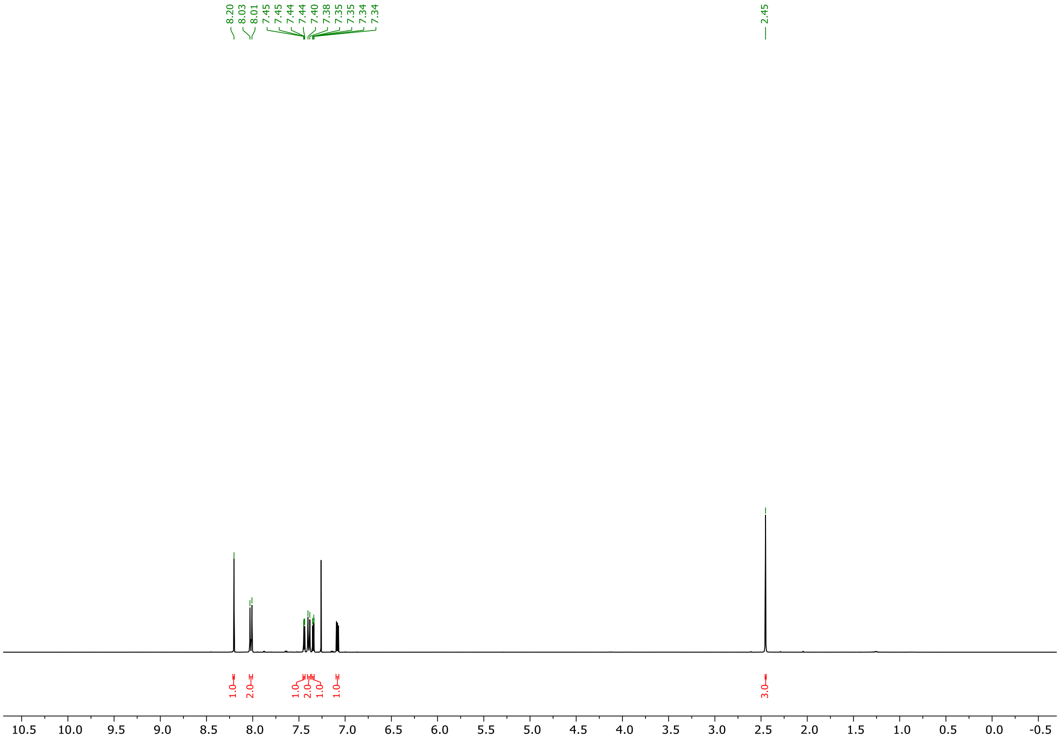

^{13}C{^{1}H}, CDCl$_3$, 101 MHz

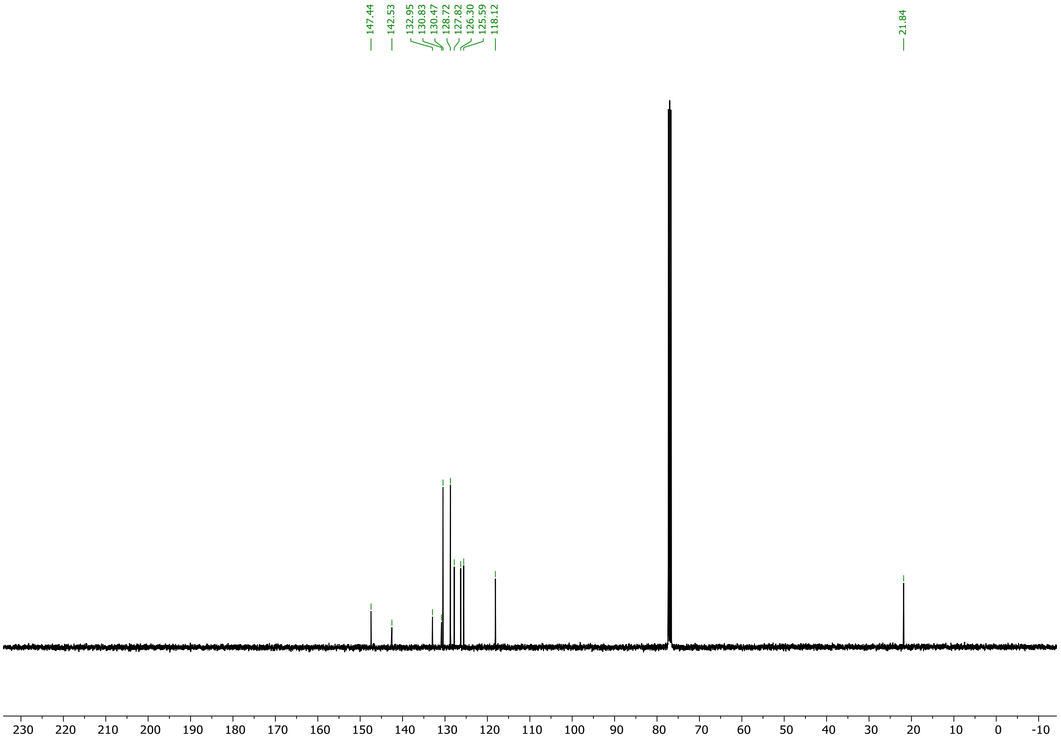

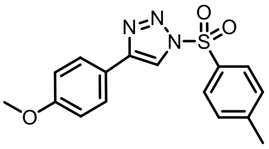

4n

^{1}H, $CDCl_3$, 400 MHz

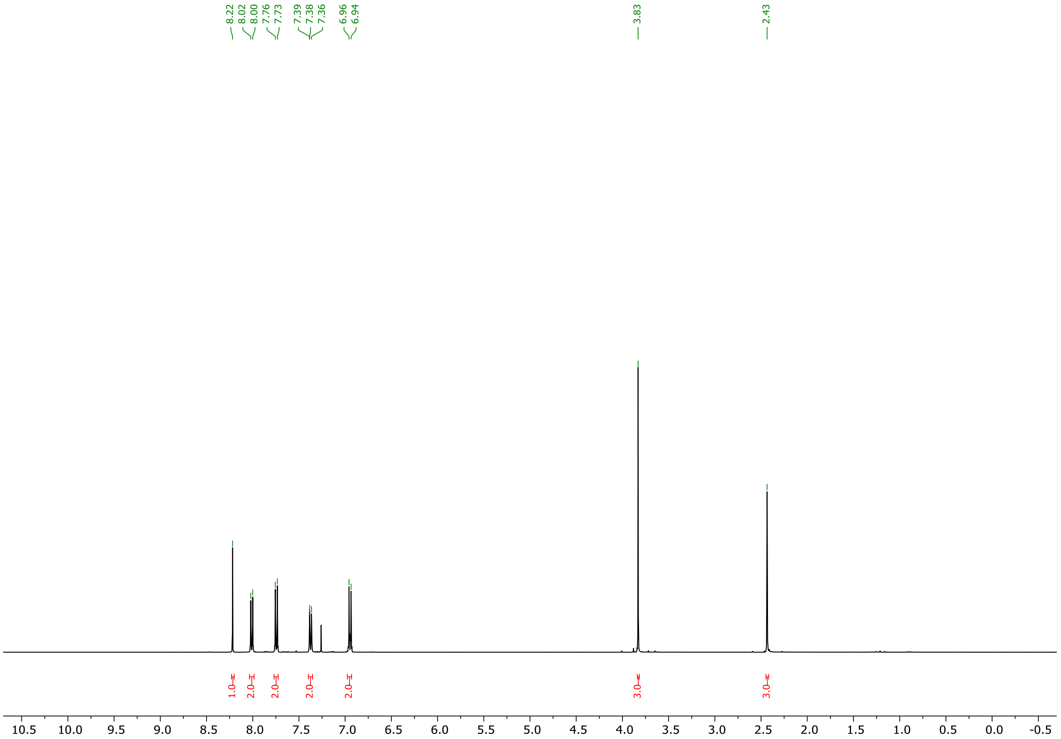

^{13}C{^{1}H}, $CDCl_3$, 101 MHz

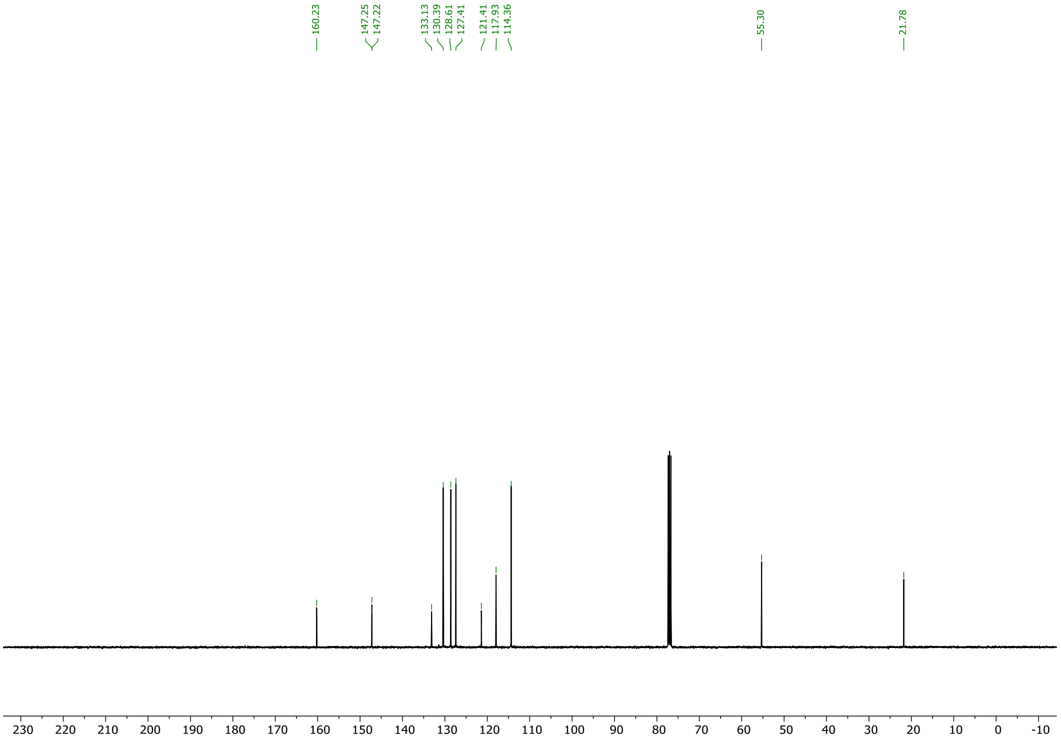

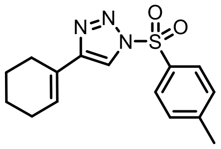

4o

^{1}H, CDCl$_3$, 400 MHz

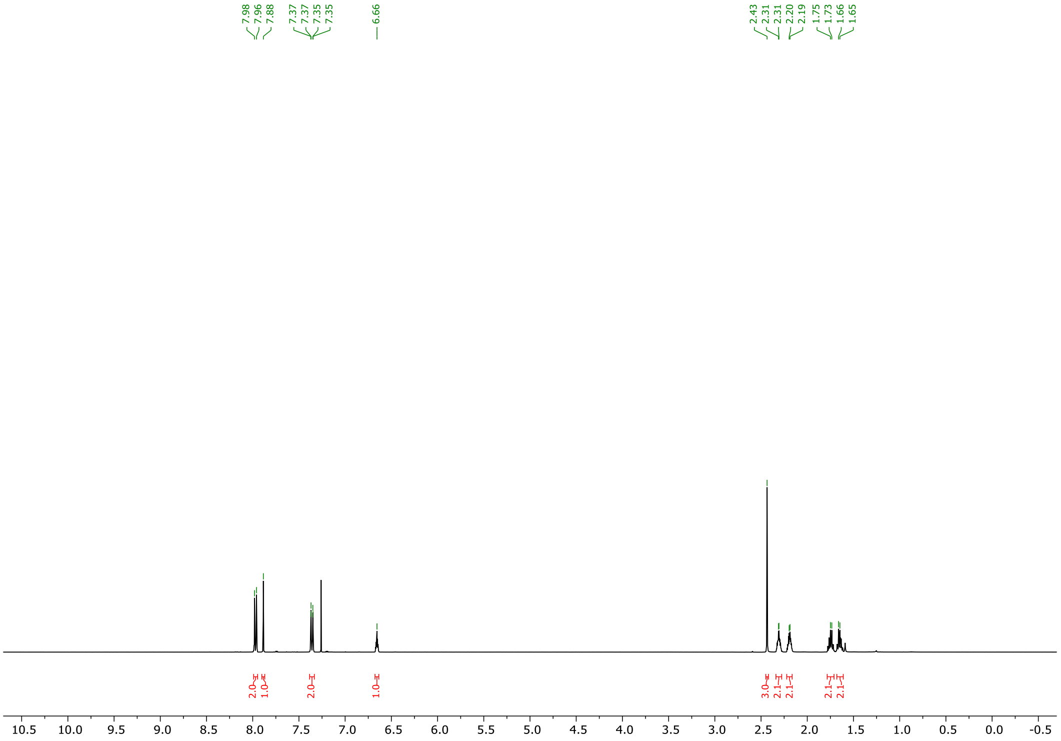

^{13}C{^{1}H}, CDCl$_3$, 101 MHz

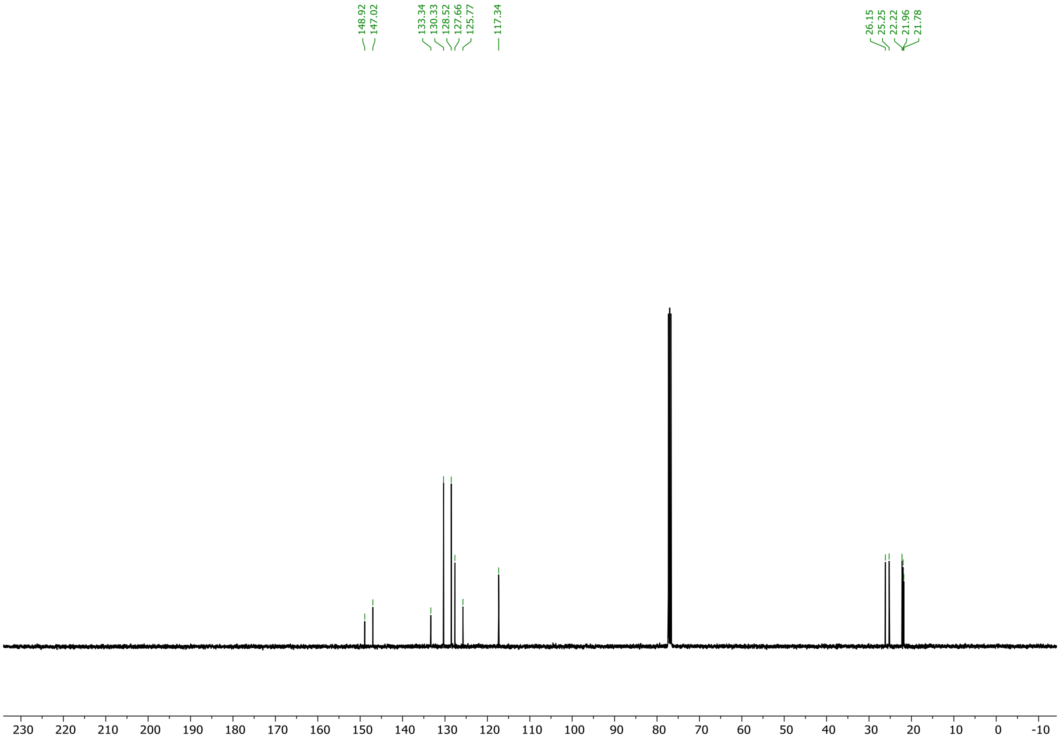

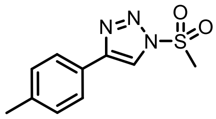

4p

^{1}H, $CDCl_3$, 500 MHz

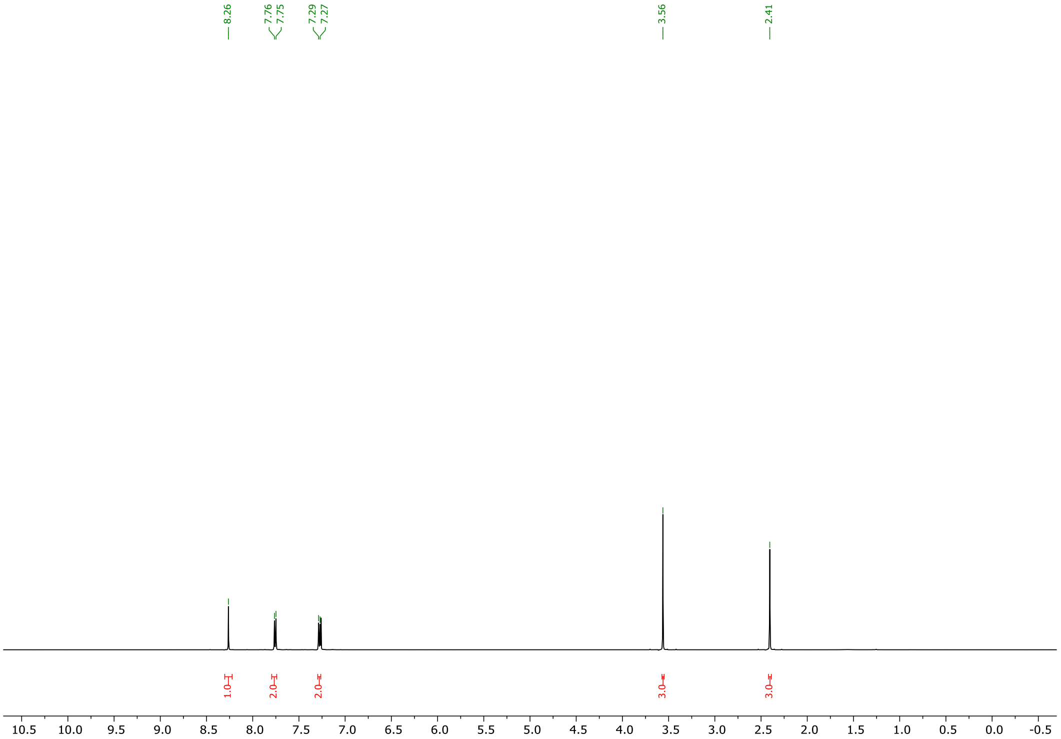

$^{13}C\{^{1}H\}$, $CDCl_3$, 126 MHz

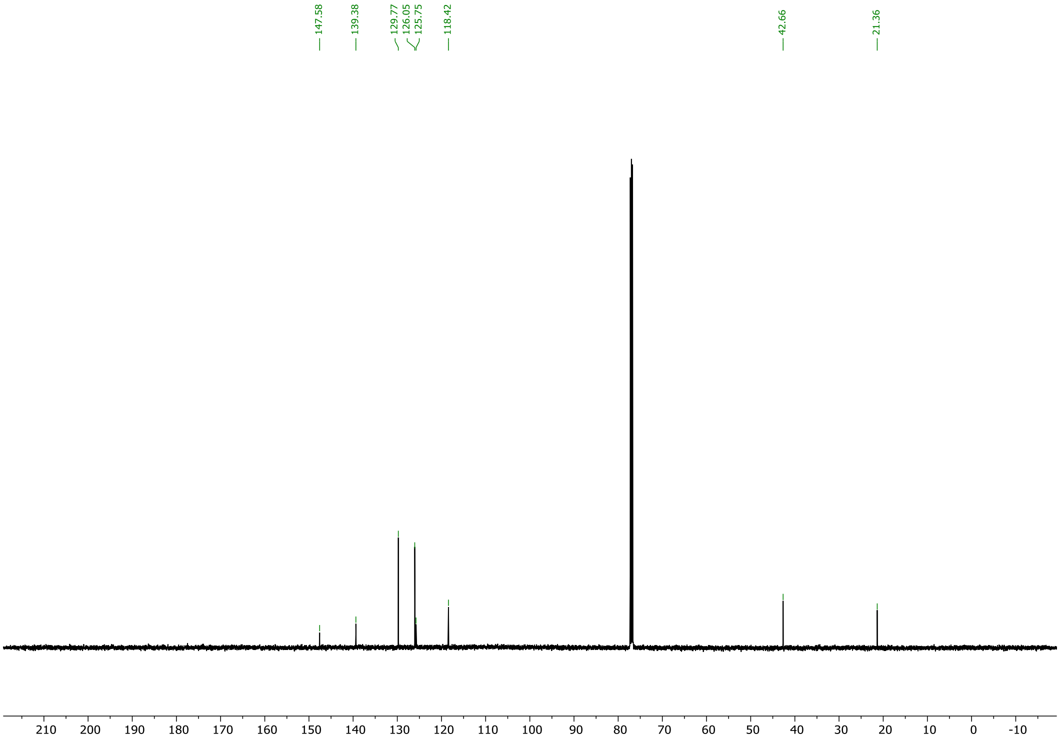

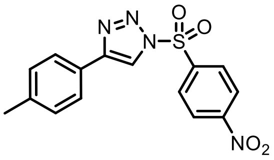

4q

^{1}H, CDCl$_3$, 500 MHz

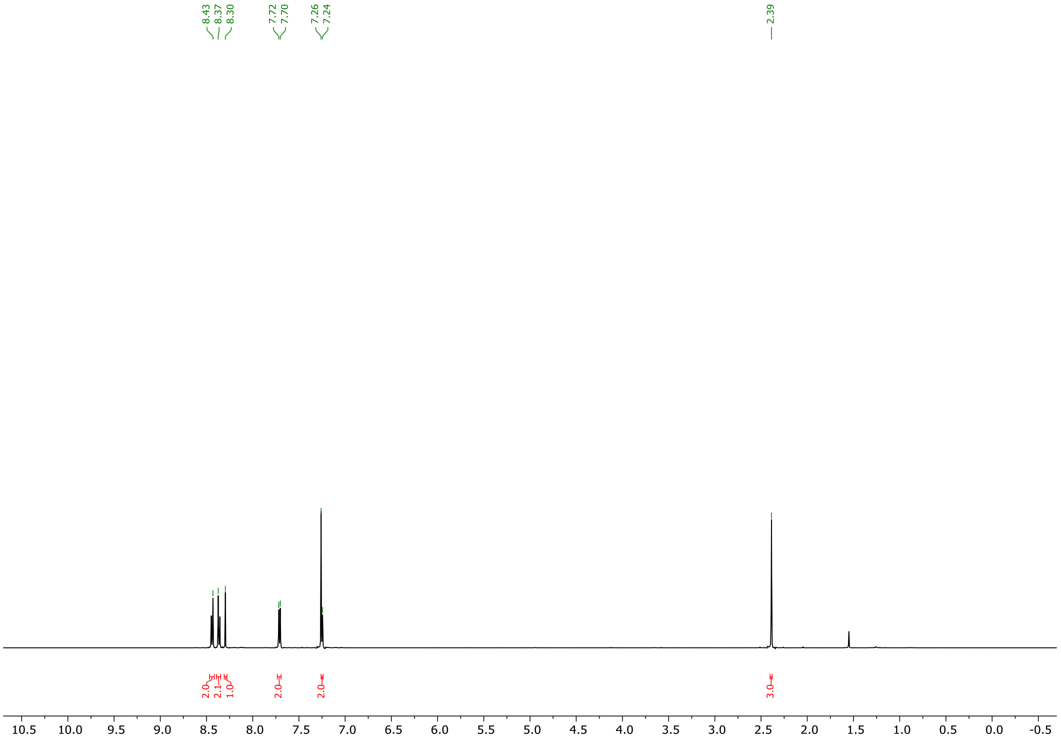

^{13}C{^{1}H}, CDCl$_3$, 126 MHz

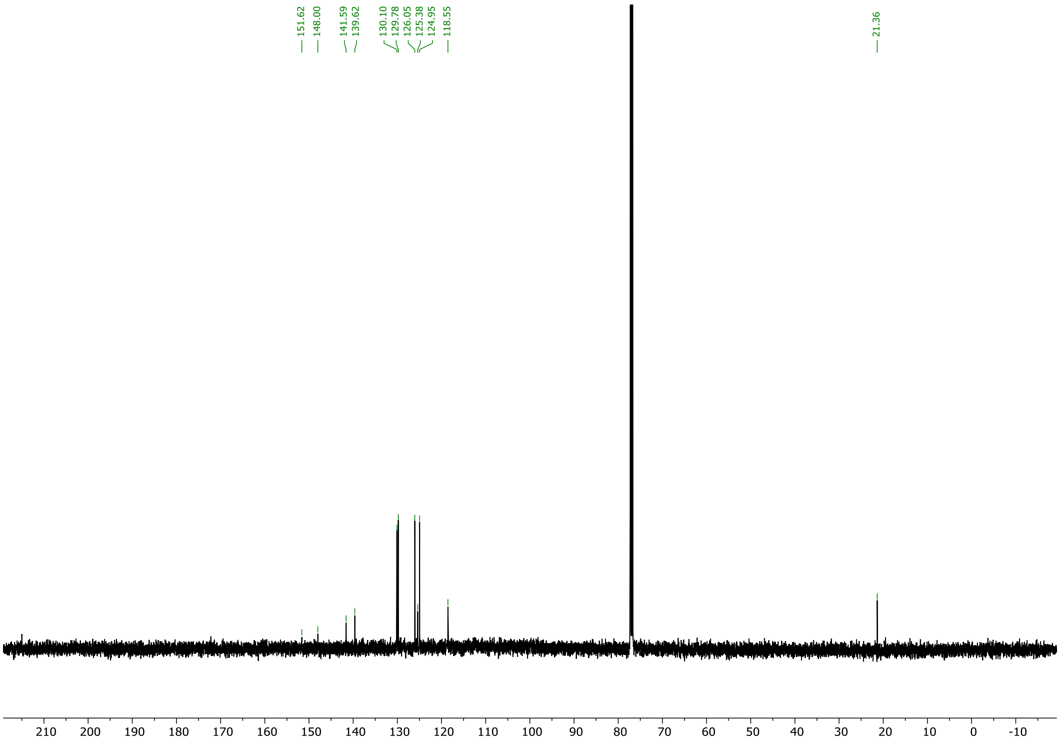

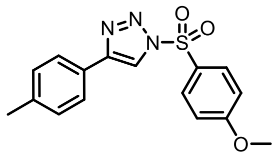

4r

^{1}H, $CDCl_3$, 500 MHz

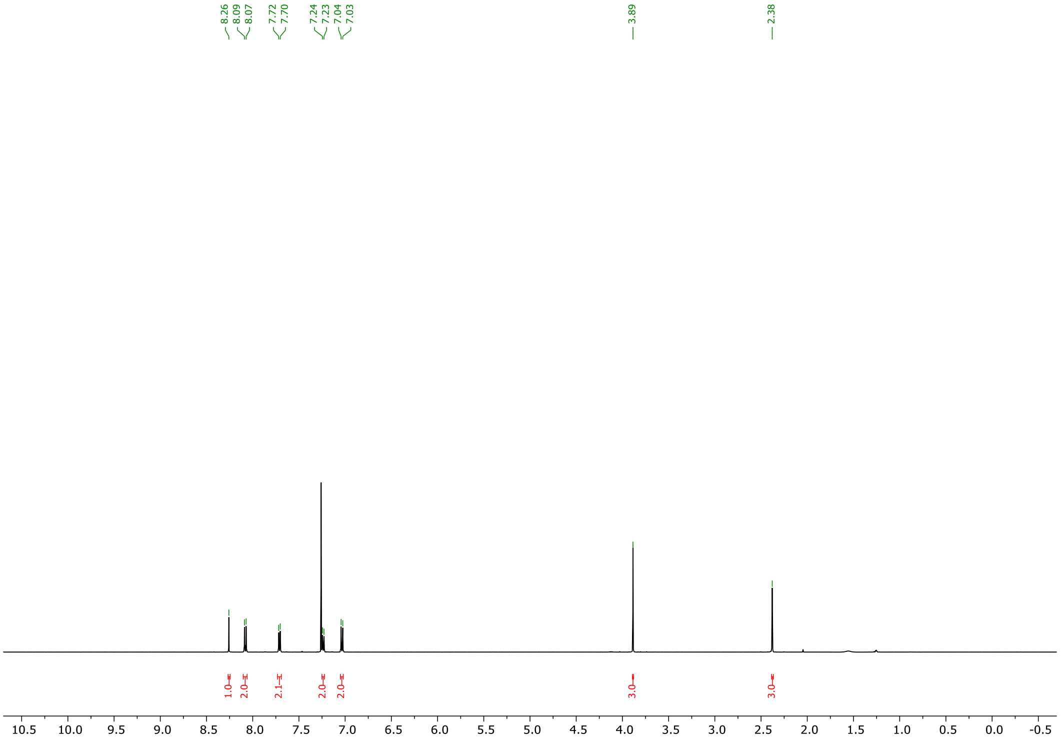

$^{13}C\{^{1}H\}$, $CDCl_3$, 101 MHz

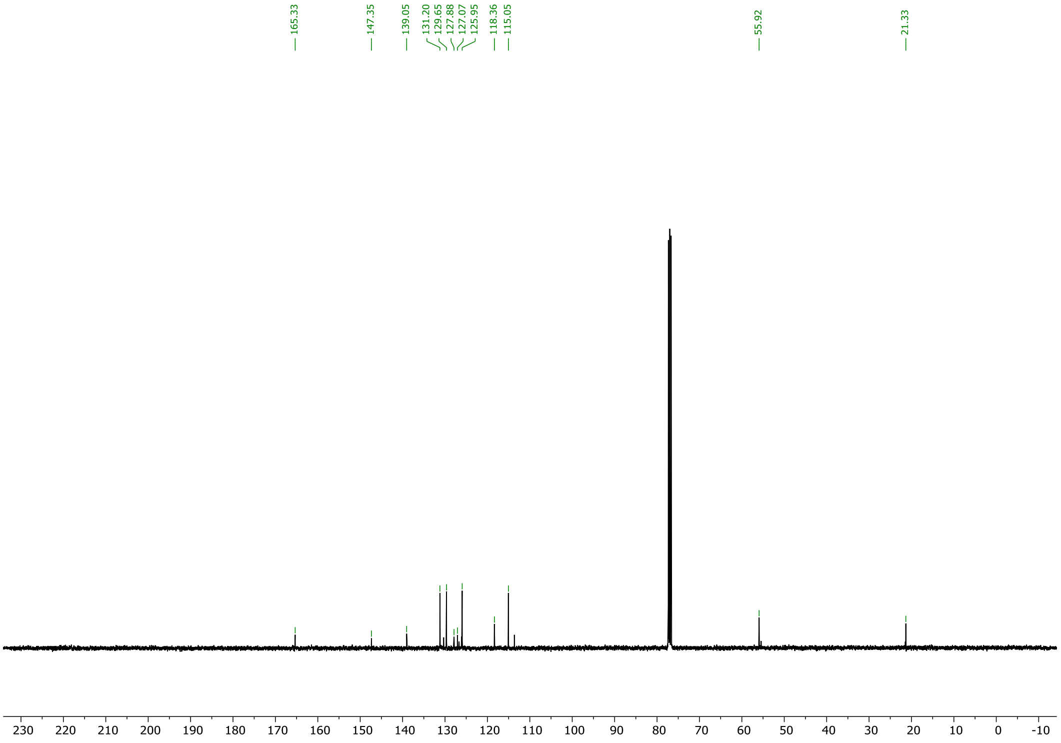

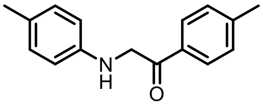

16a

^{1}H, $CDCl_3$, 500 MHz

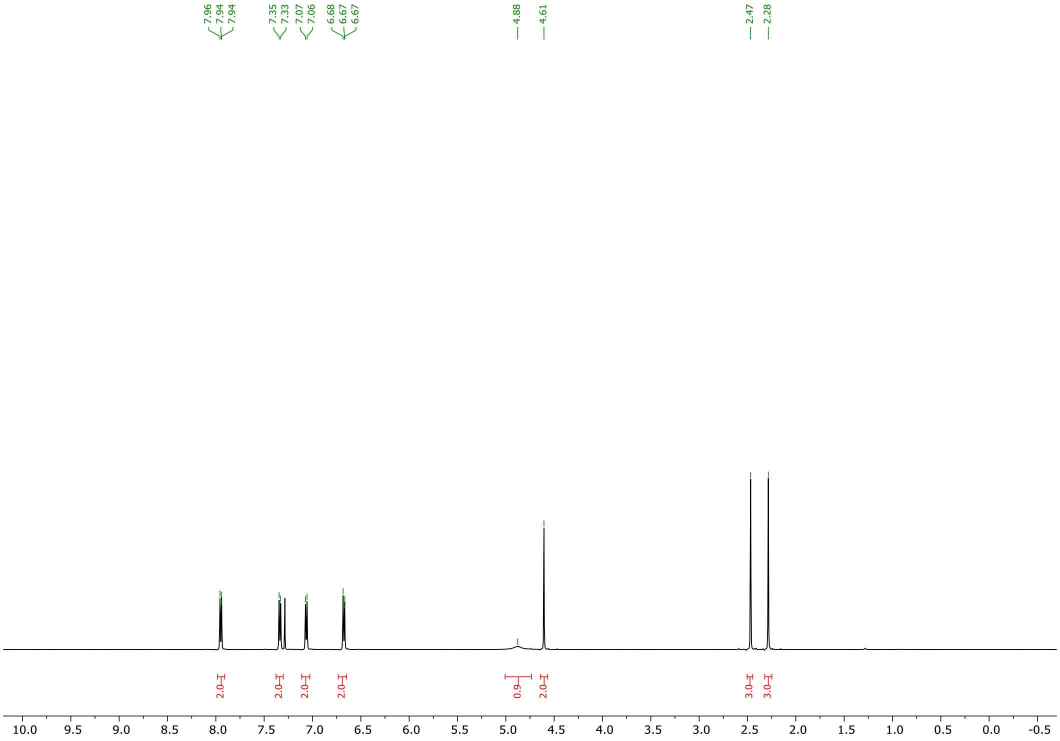

^{13}C{^{1}H}, $CDCl_3$, 126 MHz

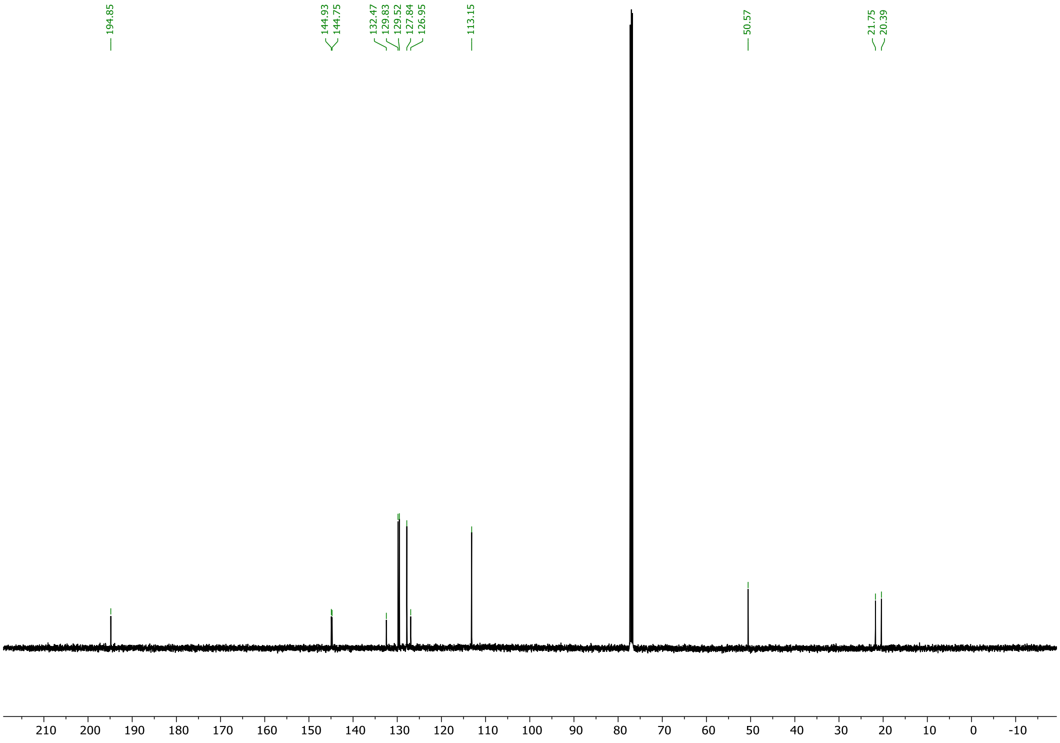

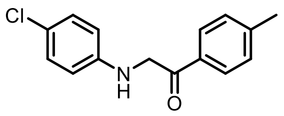

16b

^{1}H, CDCl$_3$, 400 MHz

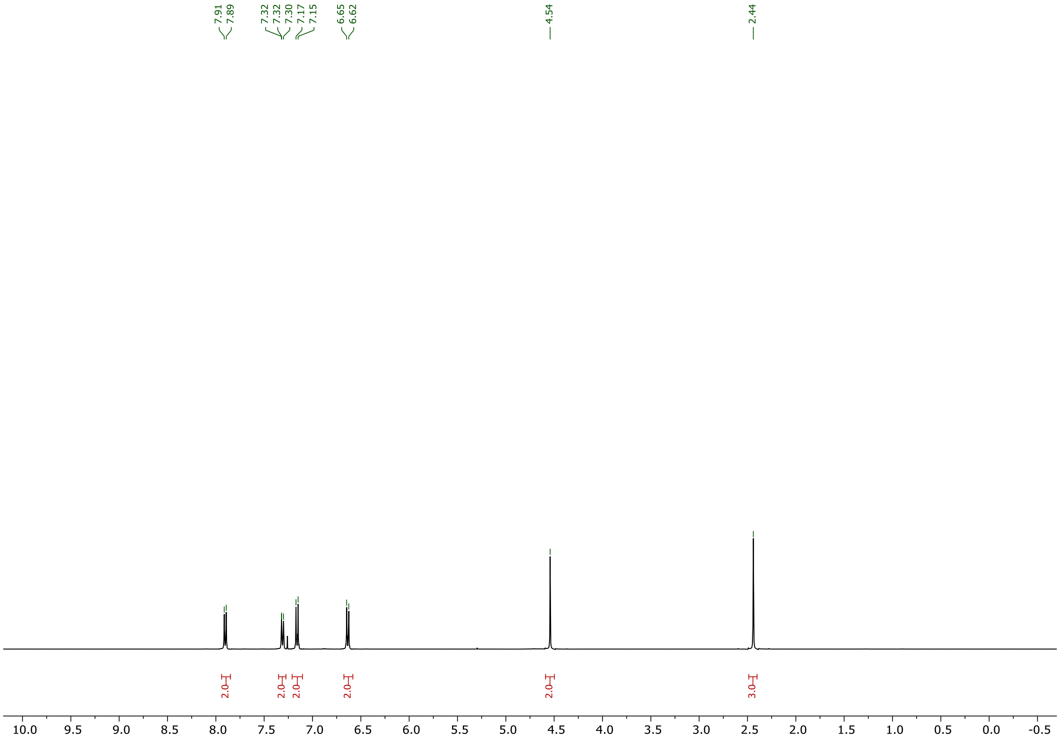

^{13}C{^{1}H}, CDCl$_3$, 101 MHz

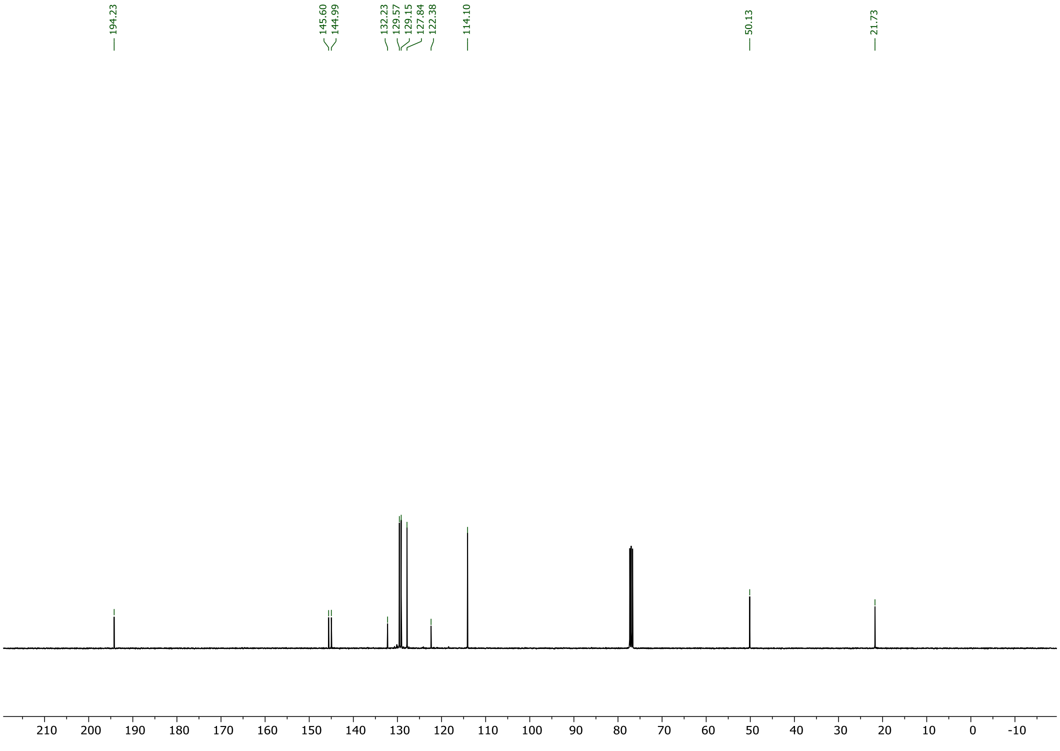

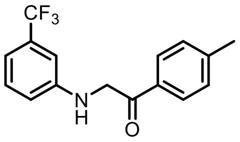

16c

^{1}H, $CDCl_3$, 400 MHz

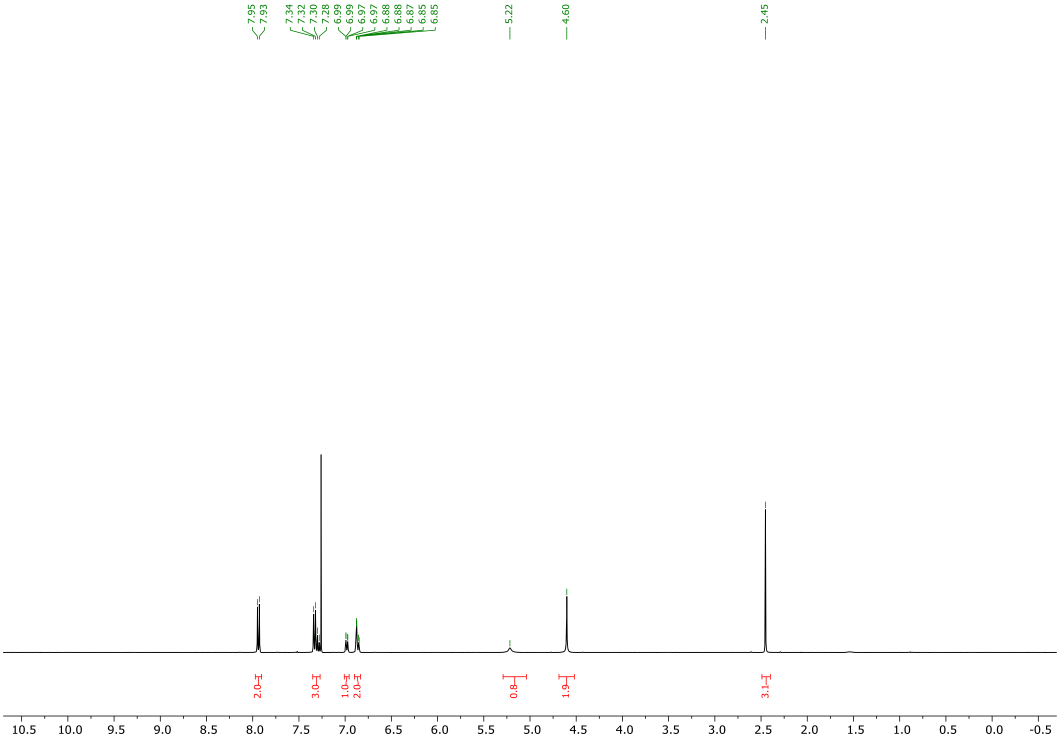

^{13}C{^{1}H}, $CDCl_3$, 101 MHz

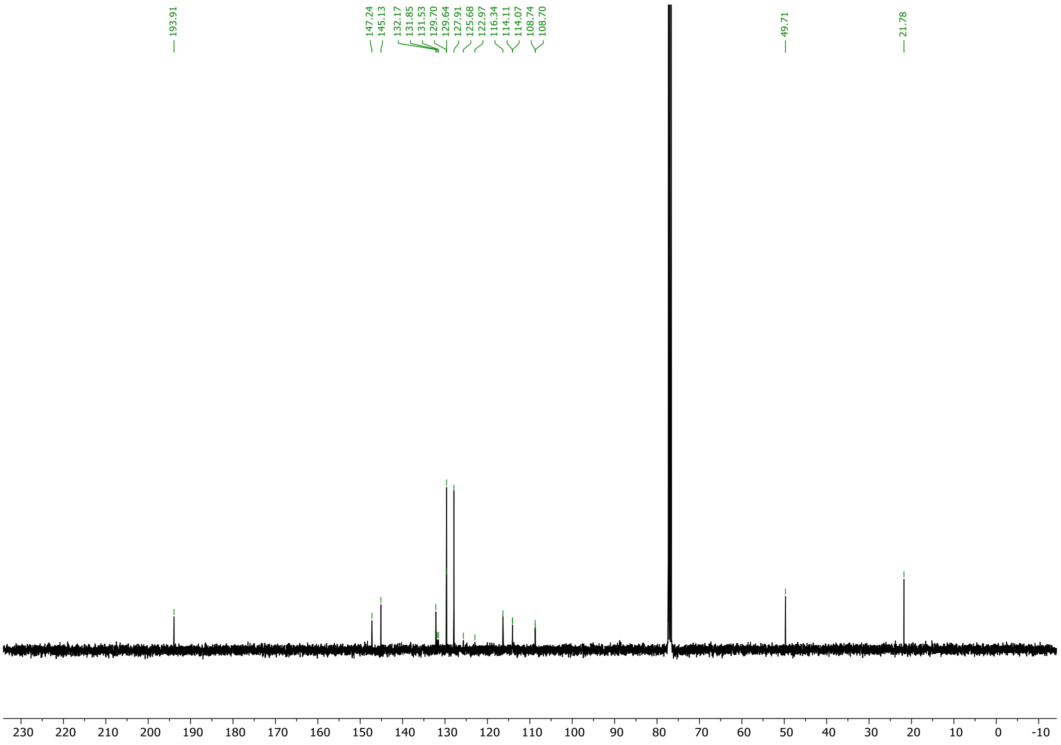

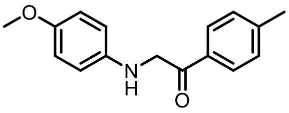

16d

^{1}H, CDCl$_3$, 400 MHz

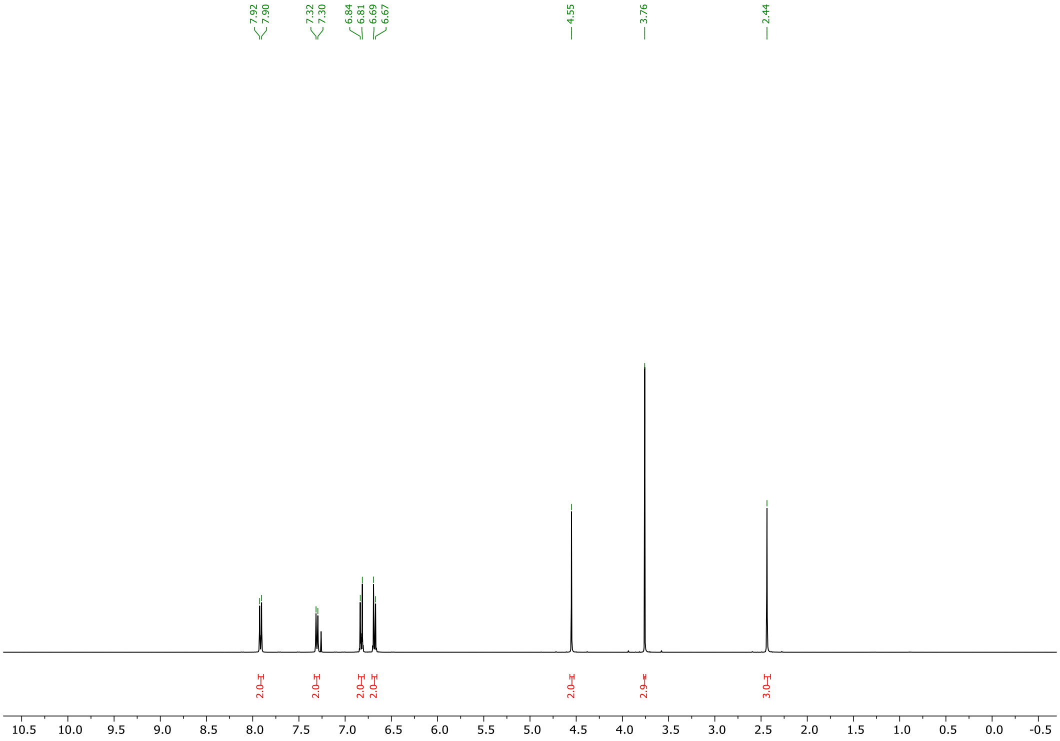

^{13}C{^{1}H}, CDCl$_3$, 101 MHz

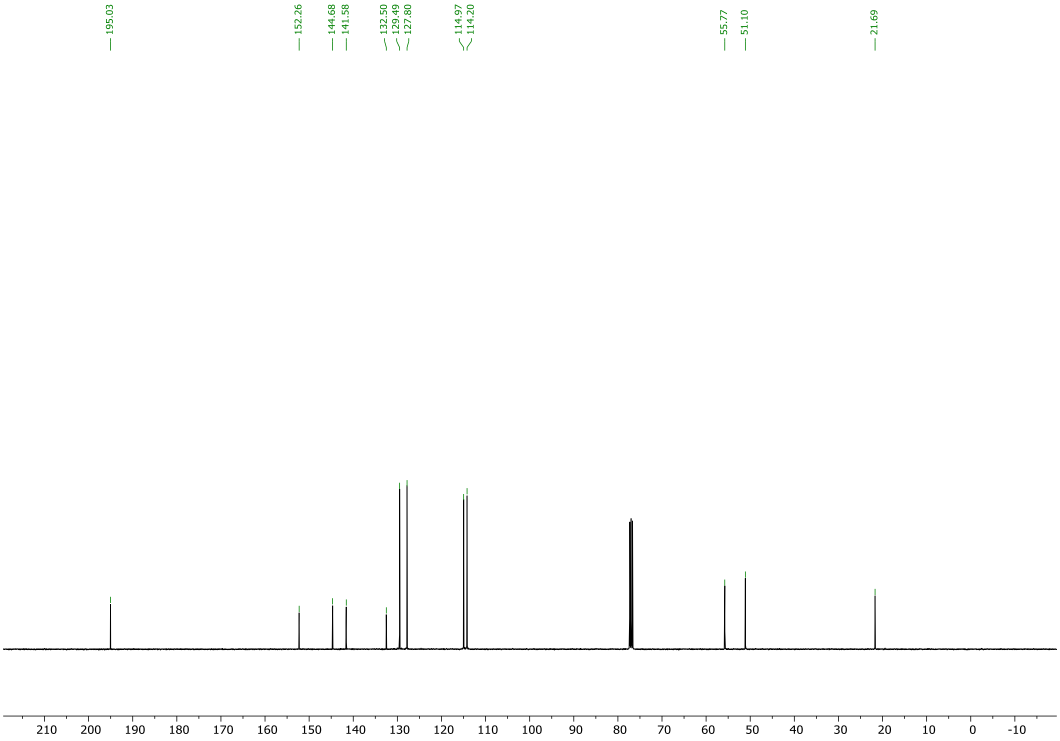

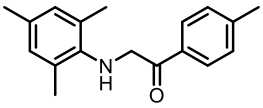

16e

^{1}H, CDCl$_3$, 400 MHz

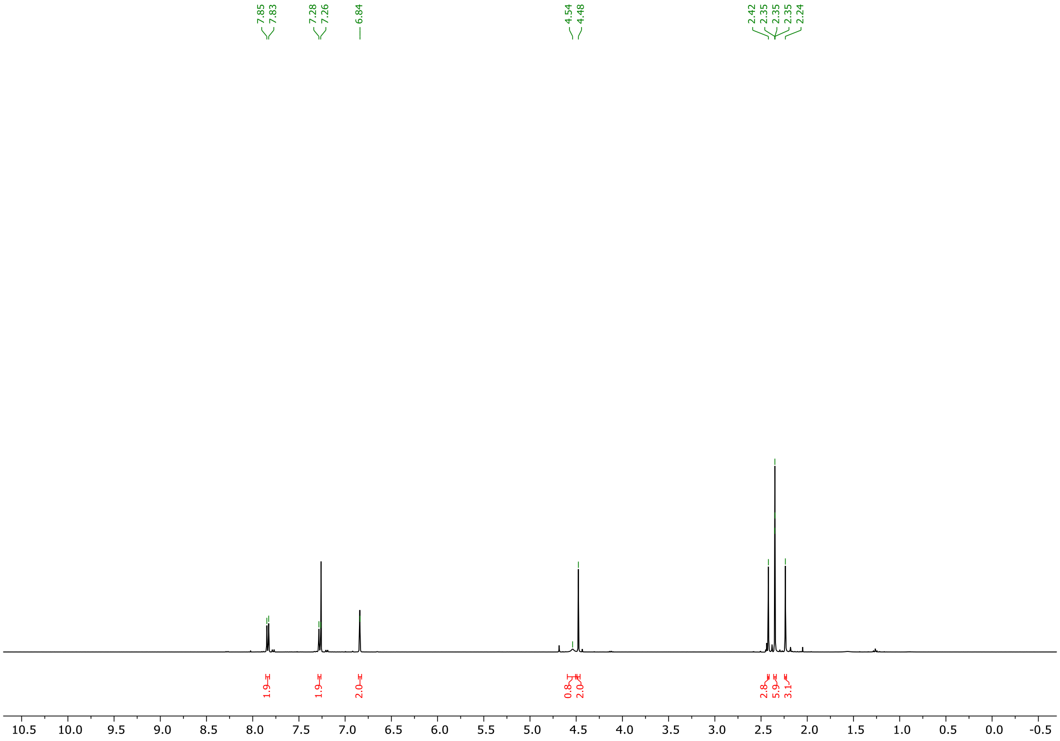

^{13}C{^{1}H}, CDCl$_3$, 101 MHz

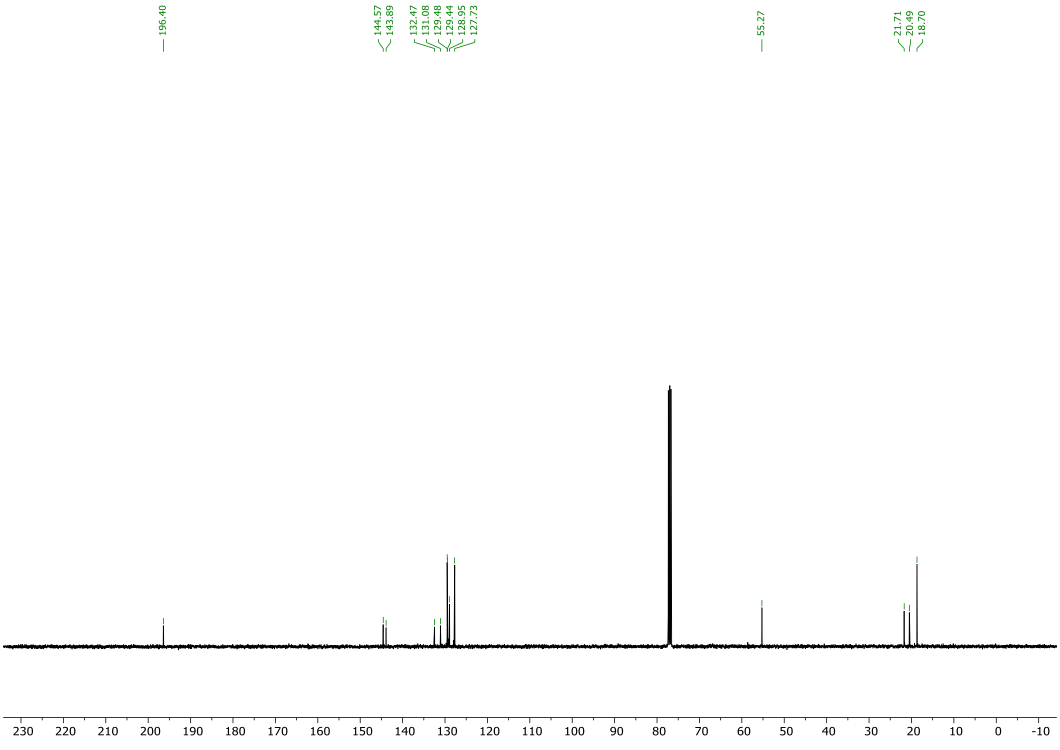

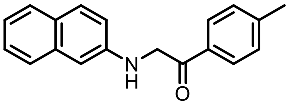

16f

^{1}H, CDCl$_3$, 400 MHz

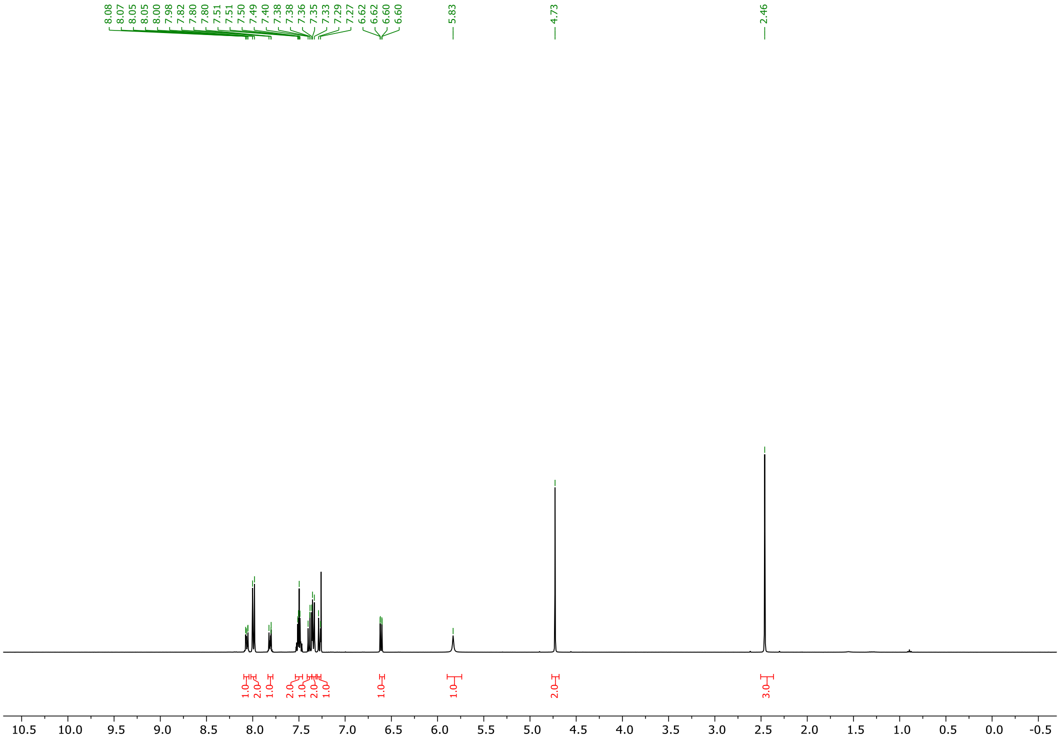

^{13}C{^{1}H}, CDCl$_3$, 101 MHz

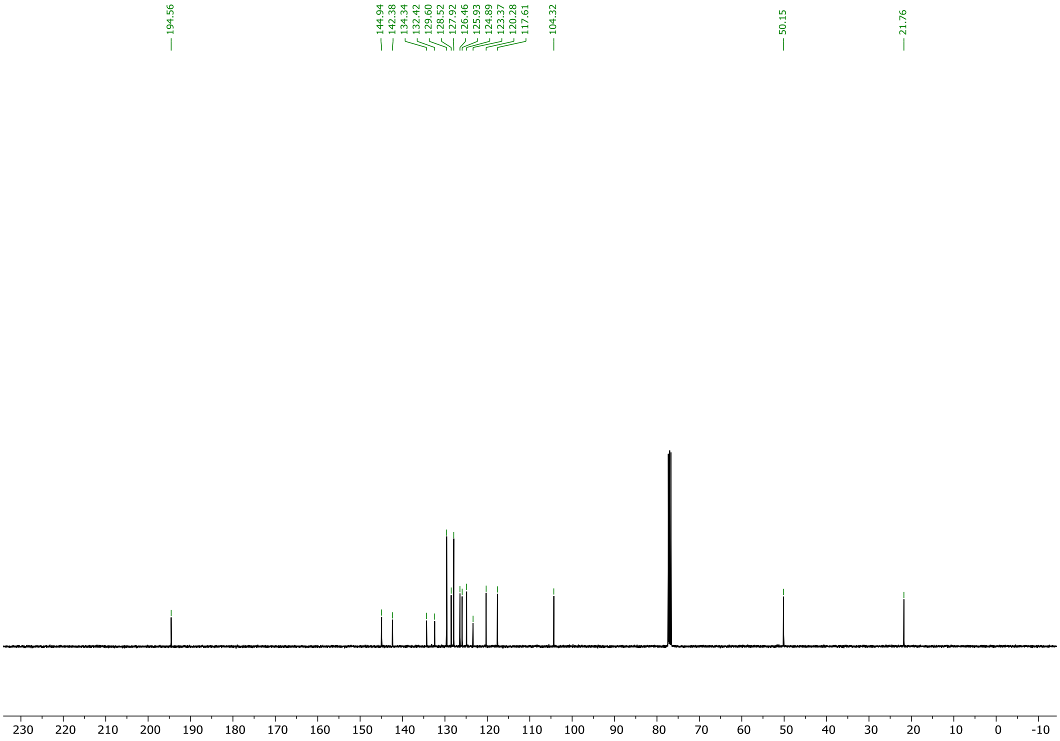

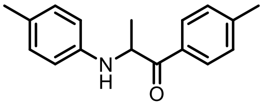

16g

^{1}H, CDCl$_3$, 400 MHz

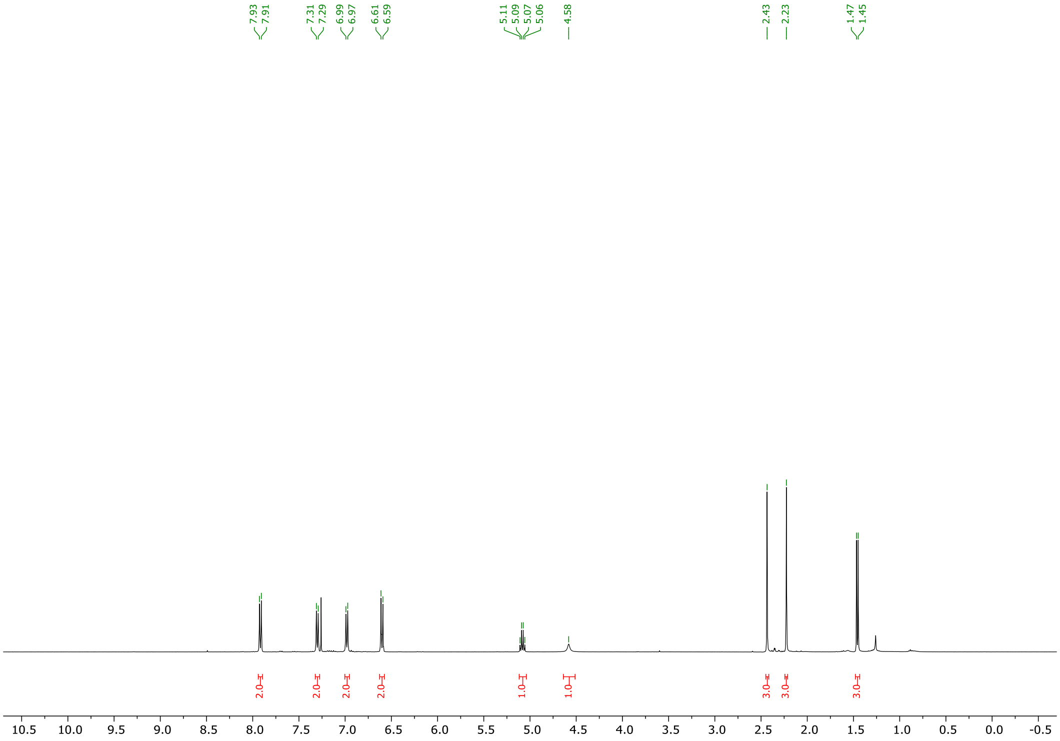

^{13}C{^{1}H}, CDCl$_3$, 101 MHz

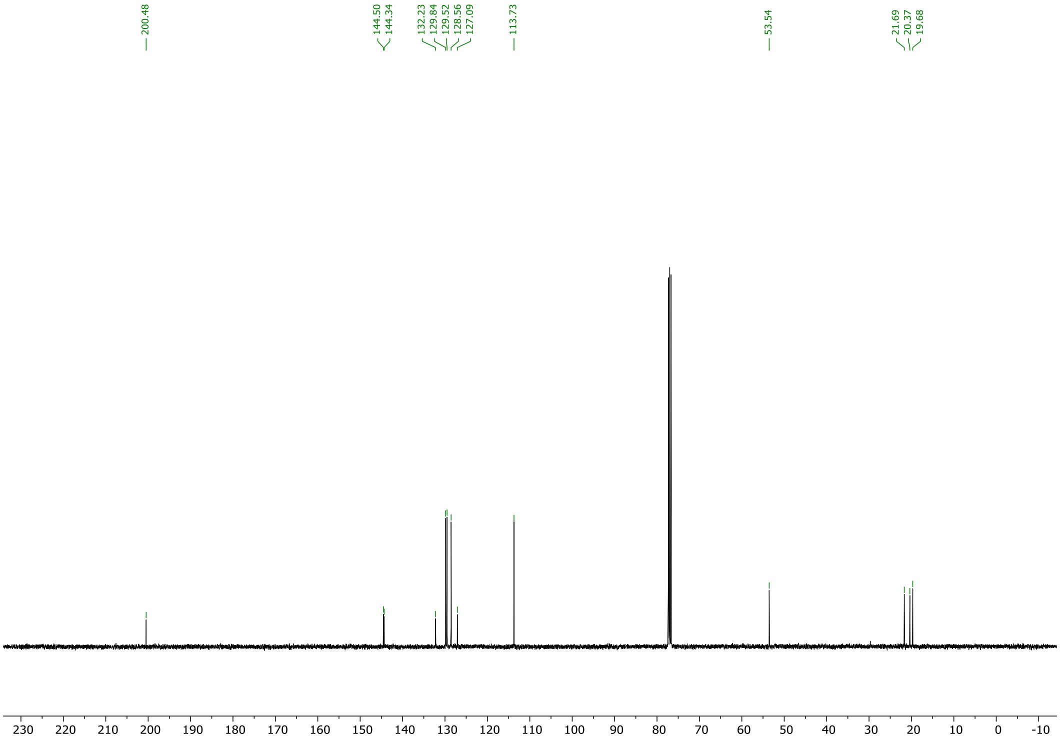

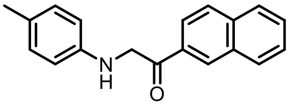

16h

^{1}H, CDCl$_3$, 400 MHz

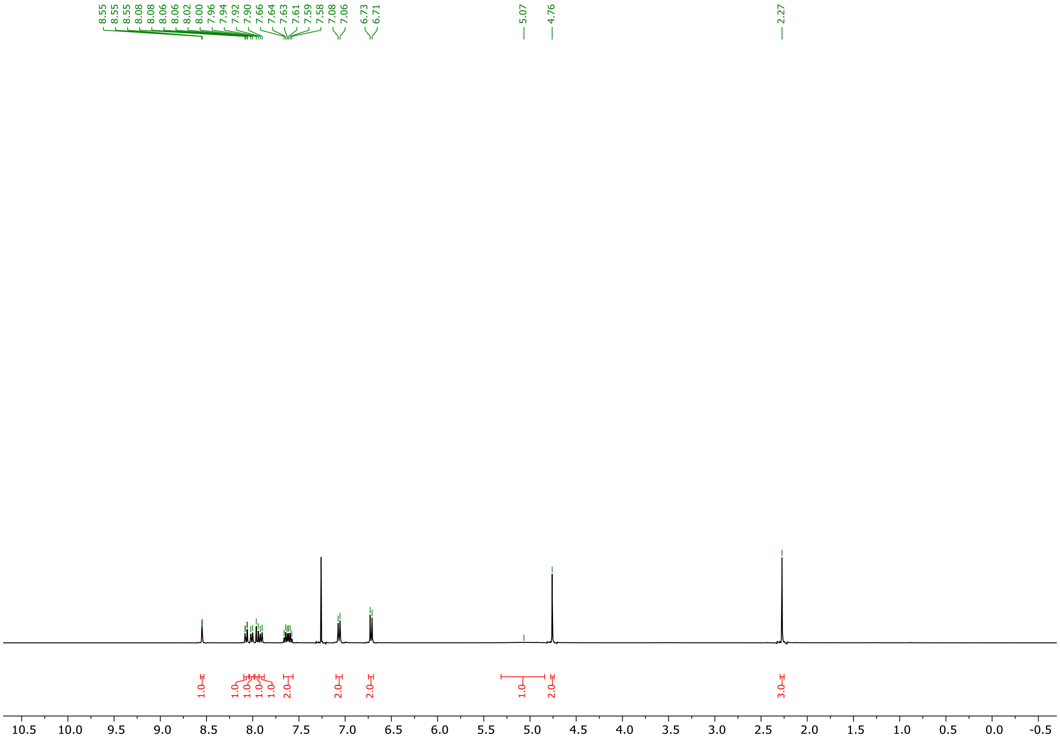

^{13}C{^{1}H}, CDCl$_3$, 101 MHz

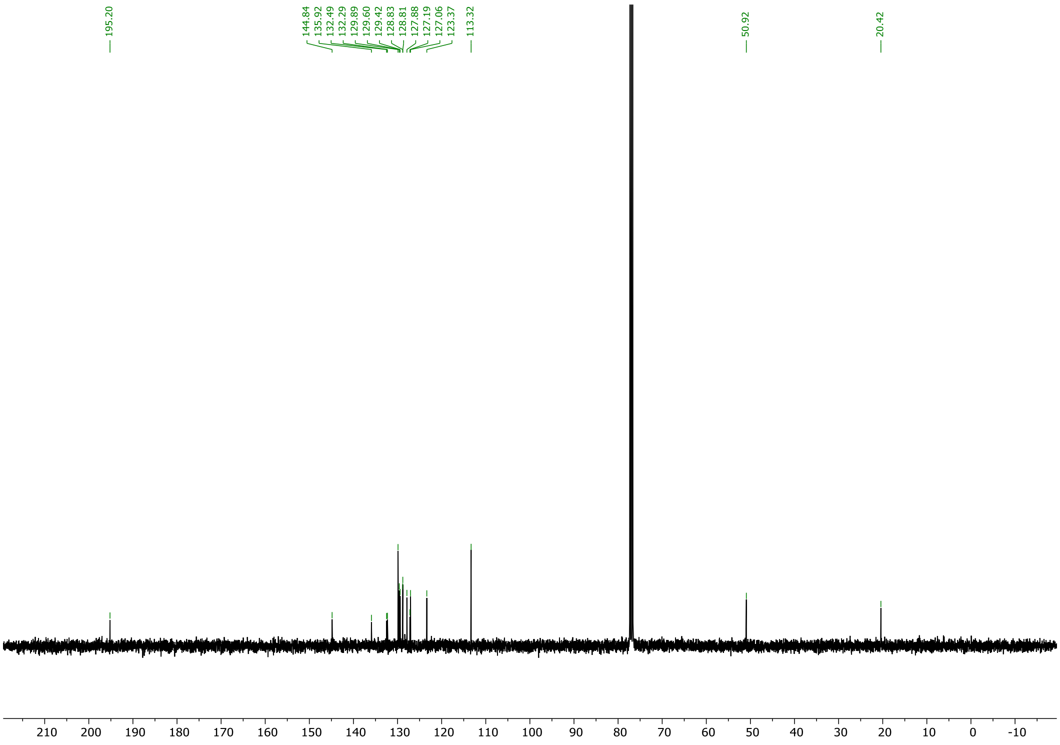

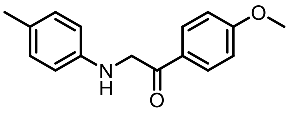

16i

^{1}H, CDCl$_3$, 400 MHz

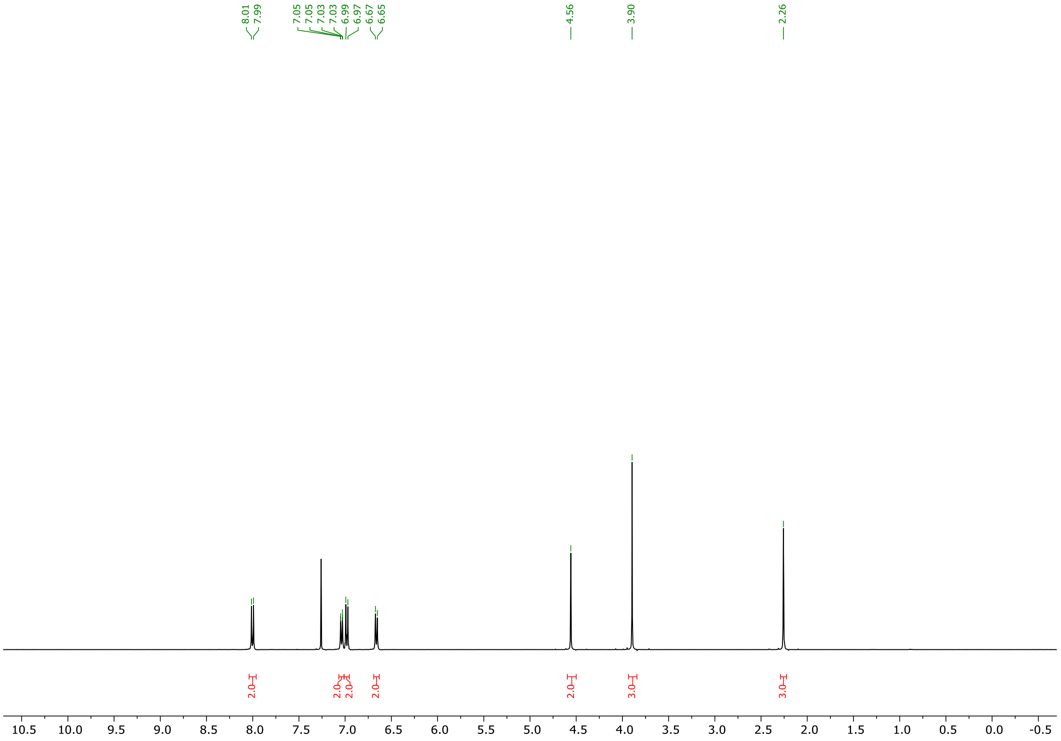

^{13}C{^{1}H}, CDCl$_3$, 101 MHz

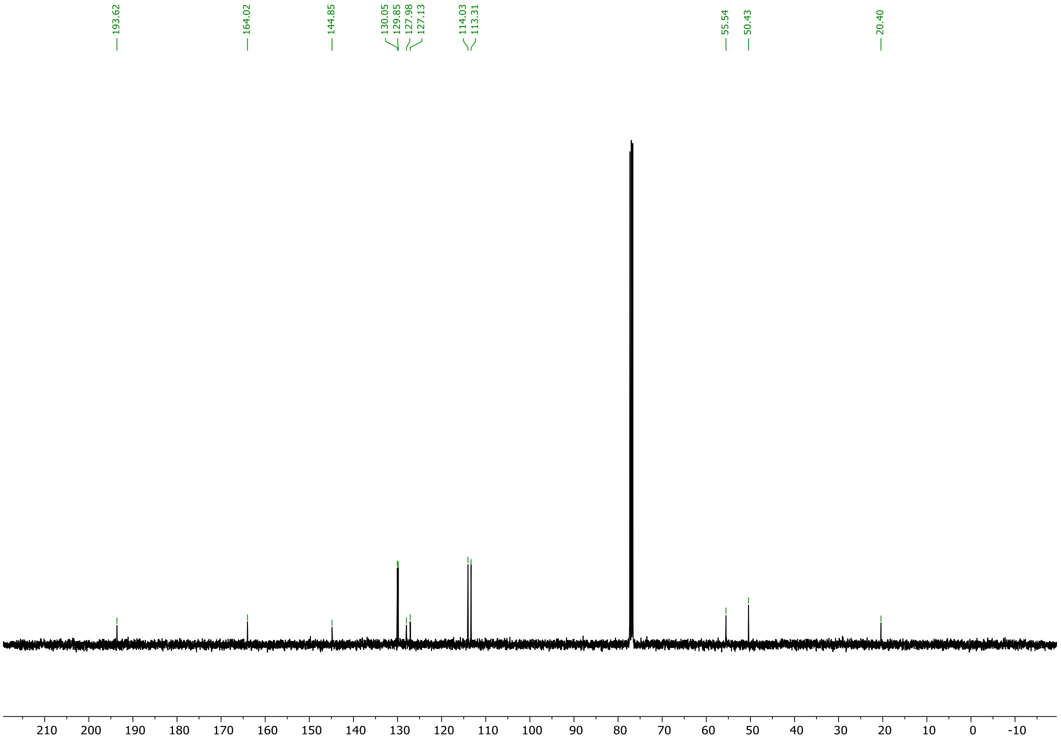

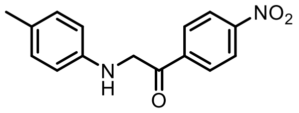

16j

^{1}H, $CDCl_3$, 400 MHz

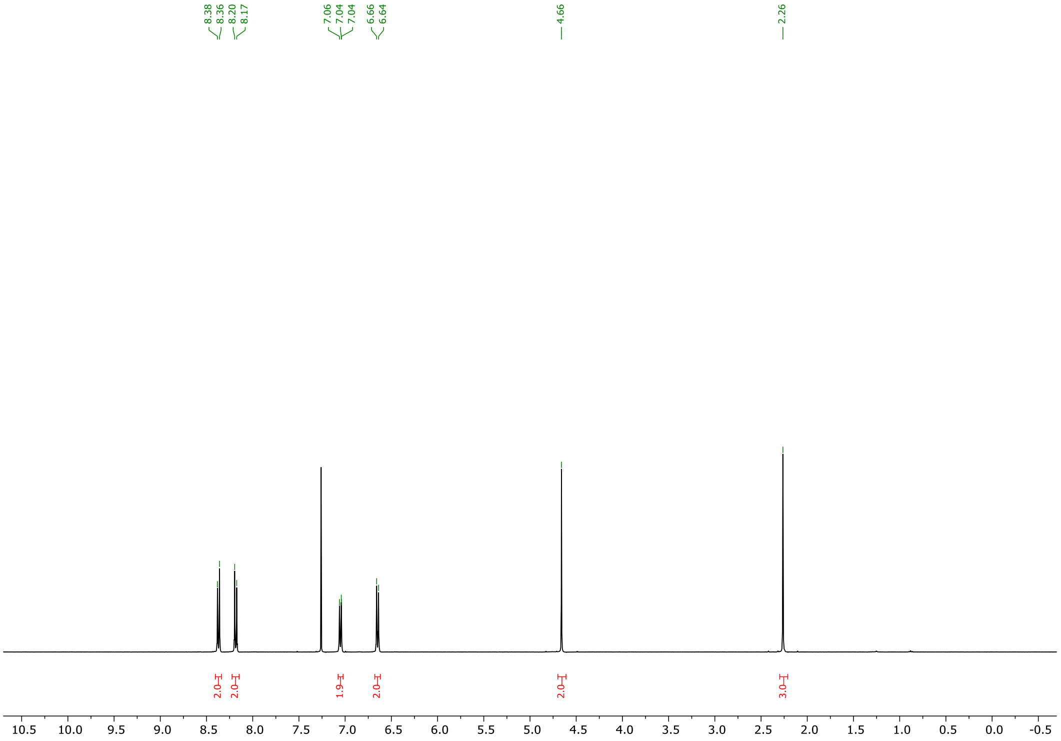

^{13}C{^{1}H}, $CDCl_3$, 101 MHz

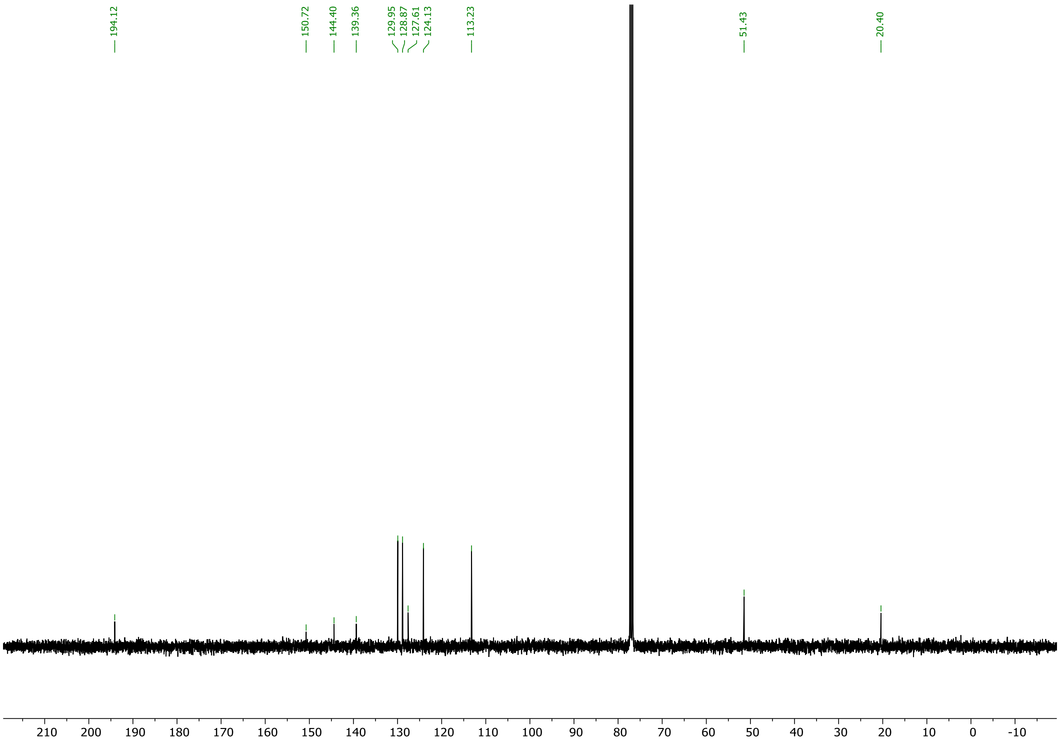

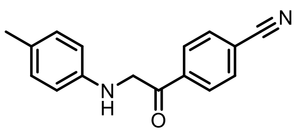

16k

^{1}H, $CDCl_3$, 400 MHz

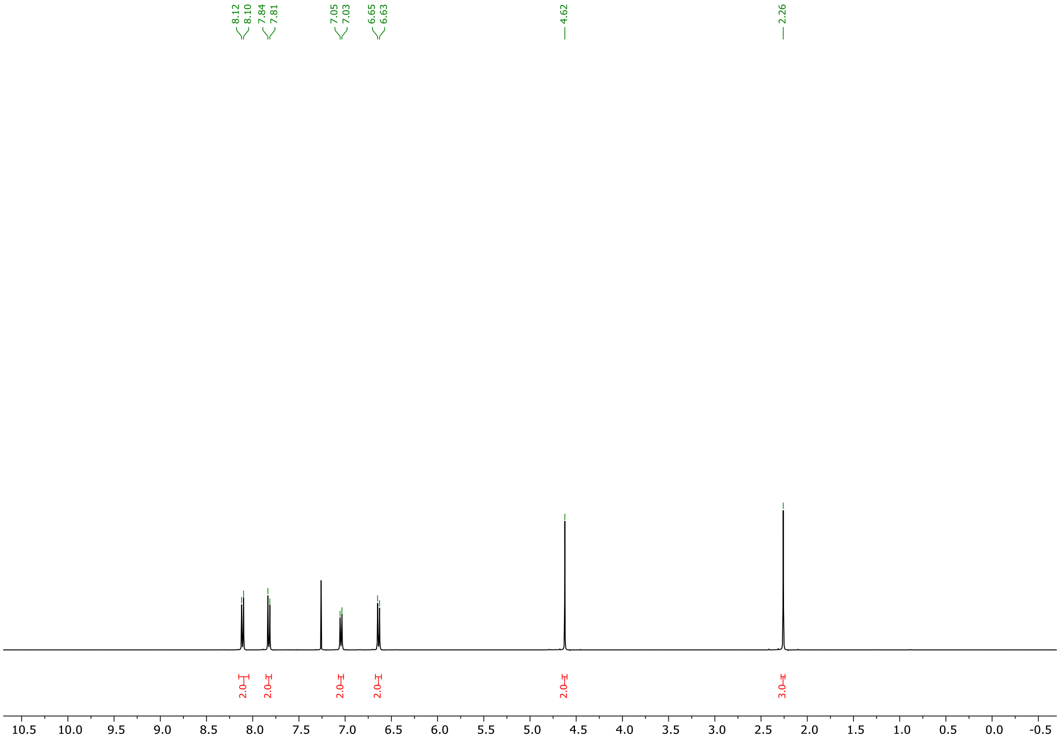

^{13}C{^{1}H}, $CDCl_3$, 101 MHz

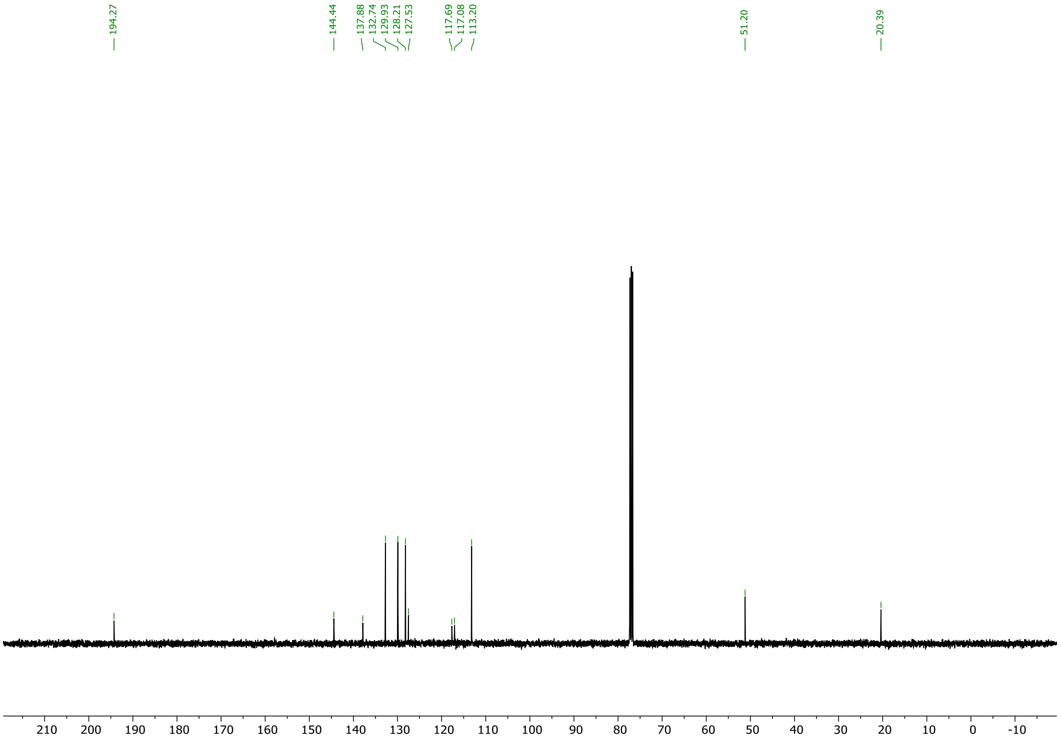

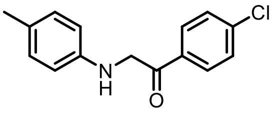

16l

^{1}H, $CDCl_3$, 400 MHz

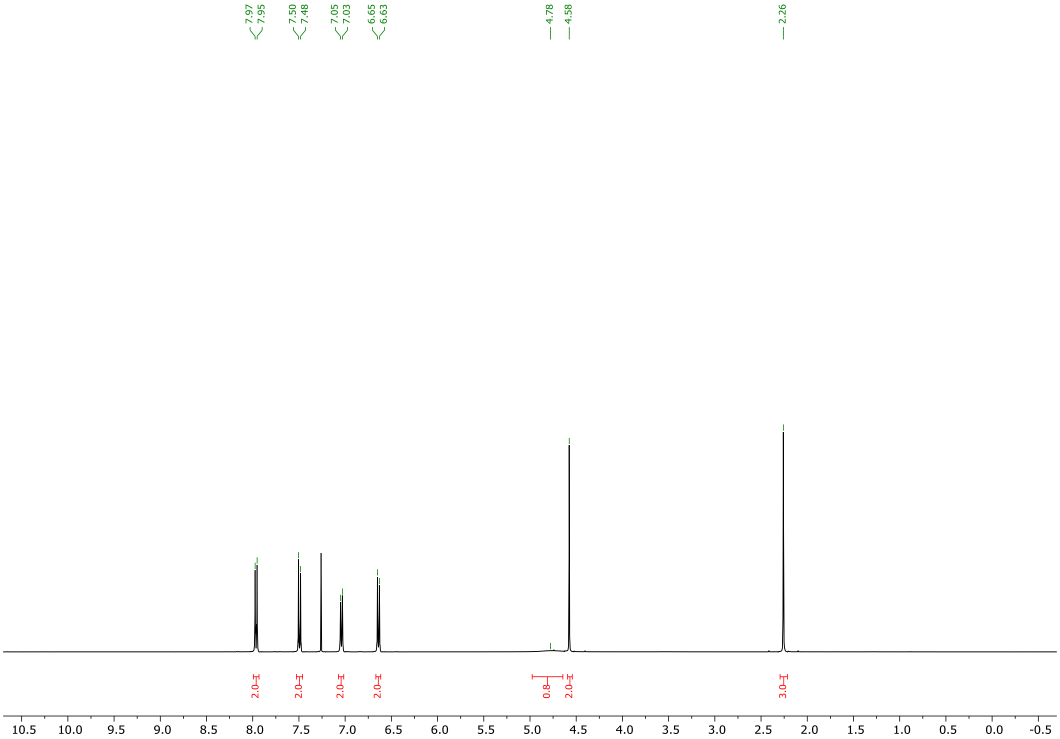

^{13}C{^{1}H}, $CDCl_3$, 101 MHz

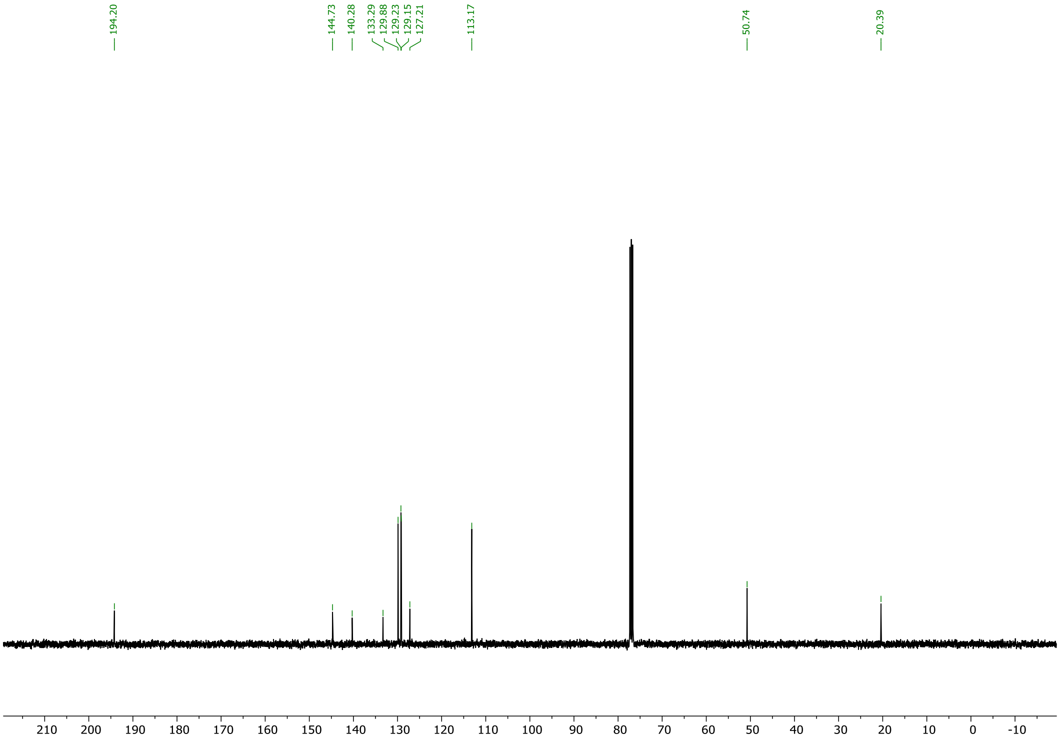

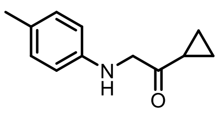

16u

^{1}H, CDCl$_3$, 400 MHz

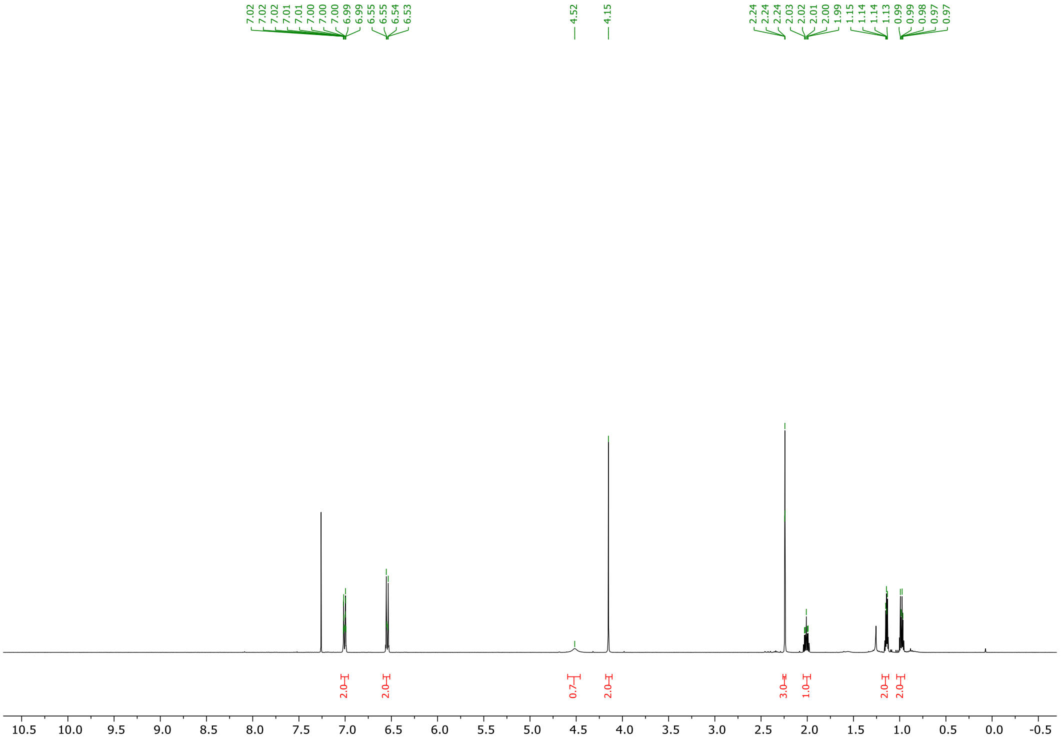

^{13}C{^{1}H}, CDCl$_3$, 101 MHz

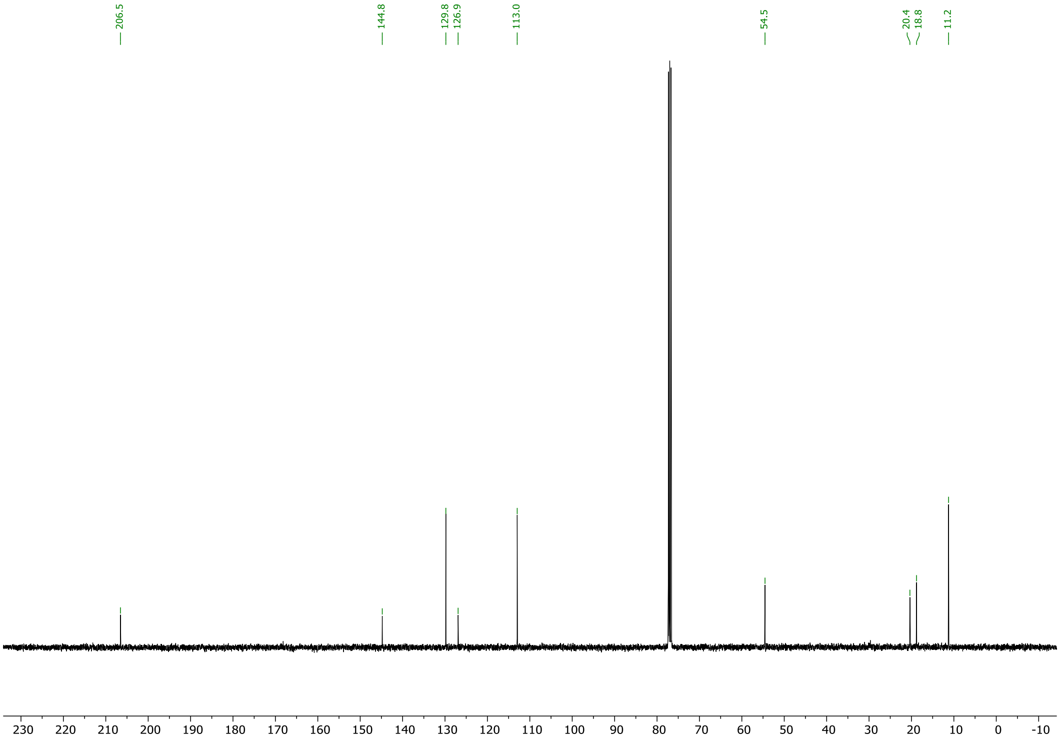

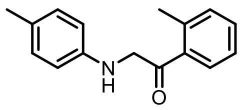

16v

^{1}H, CDCl$_3$, 400 MHz

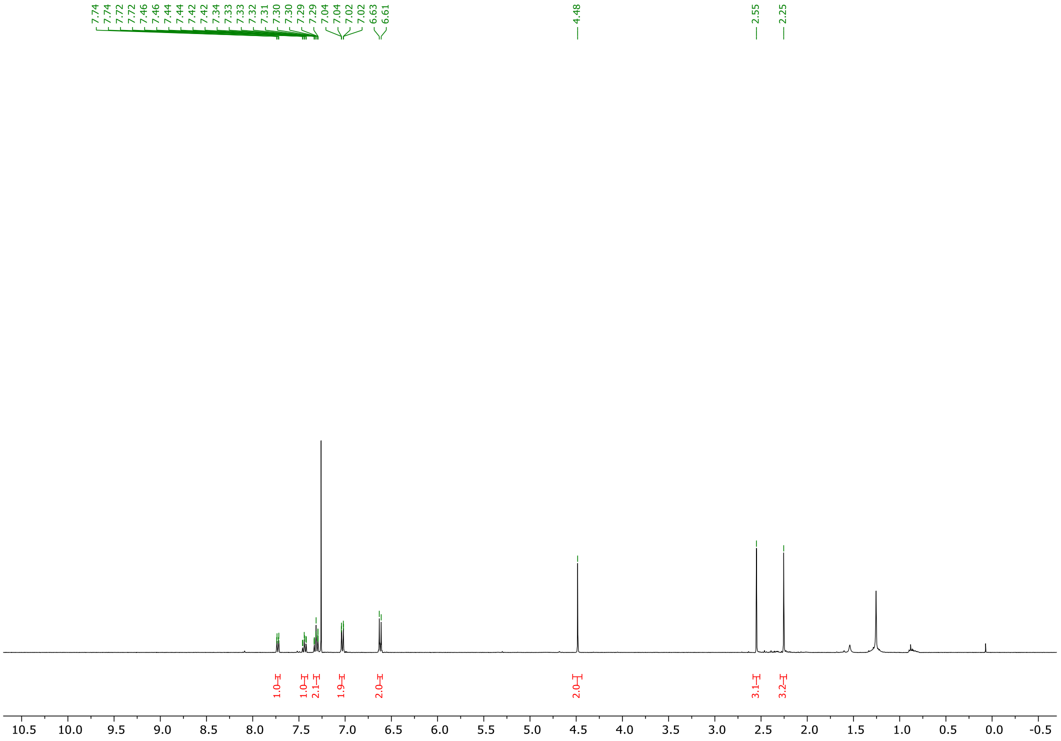

^{13}C{^{1}H}, CDCl$_3$, 101 MHz

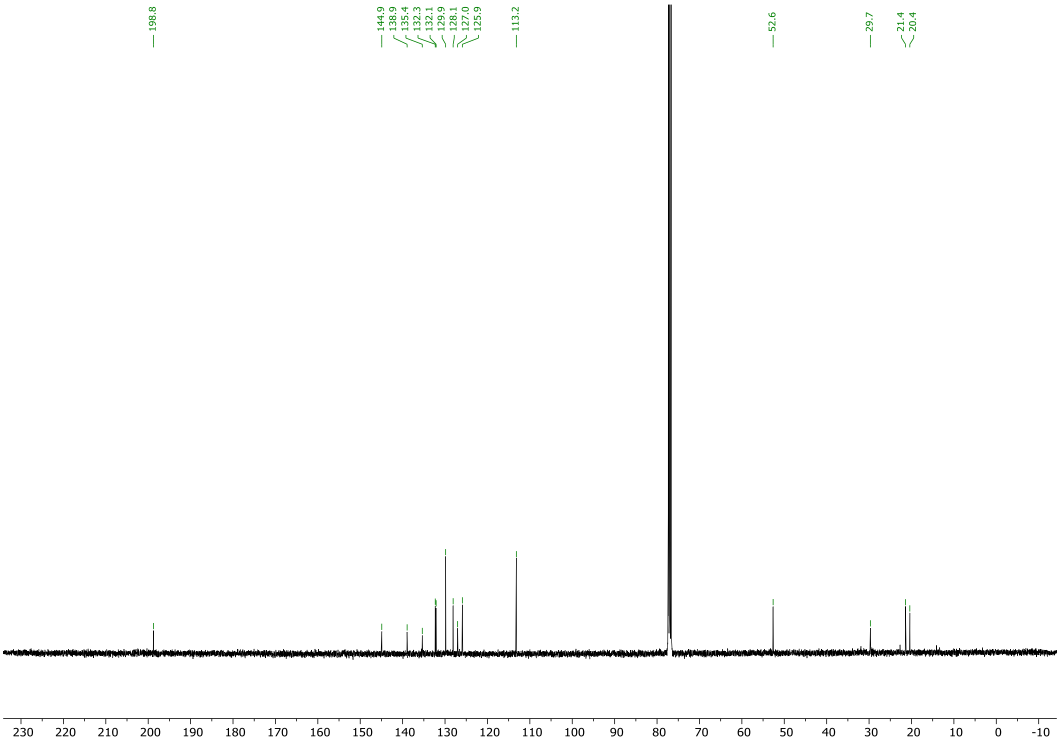

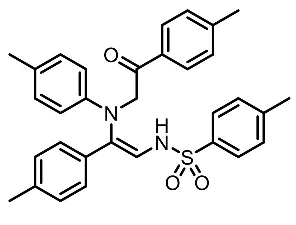

17a

^{1}H, $CDCl_3$, 400 MHz

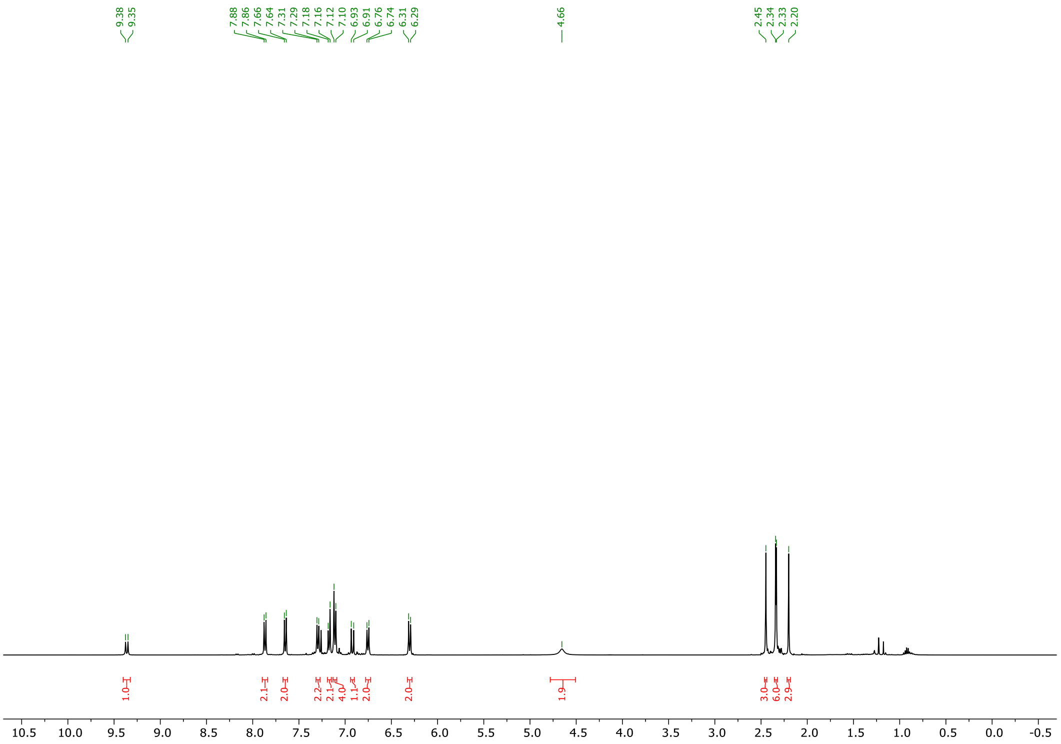

^{13}C{^{1}H}, $CDCl_3$, 101 MHz

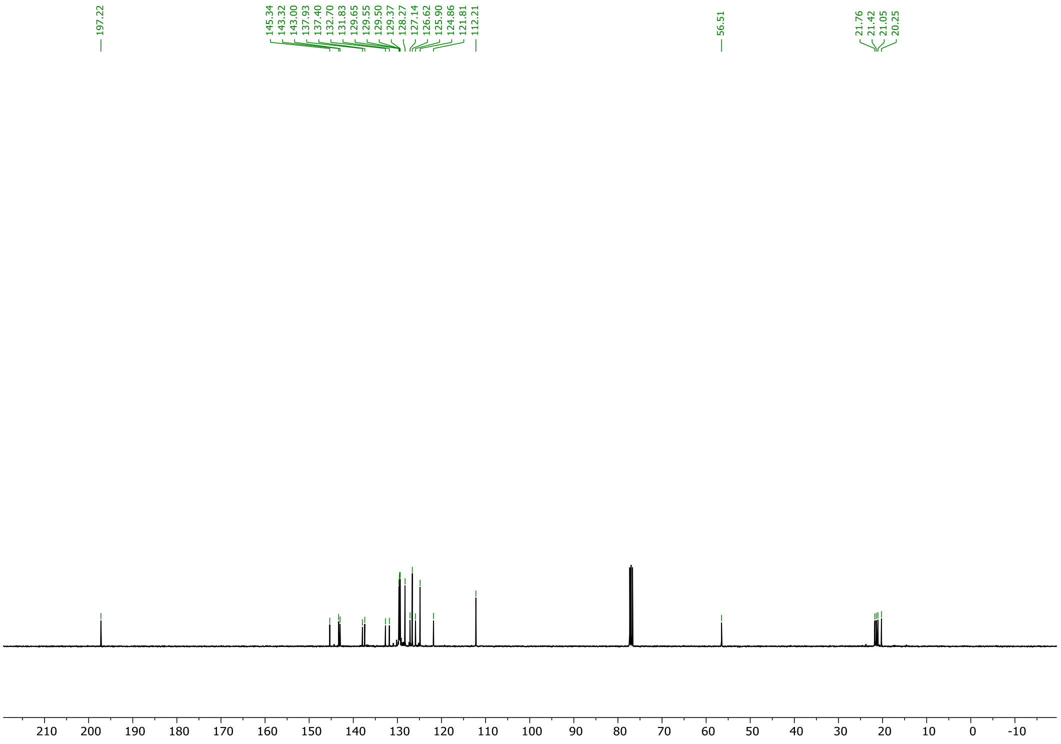

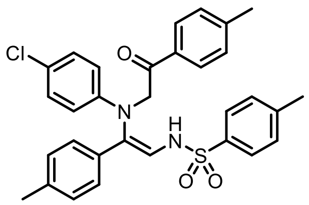

17b

^{1}H, CDCl$_3$, 400 MHz

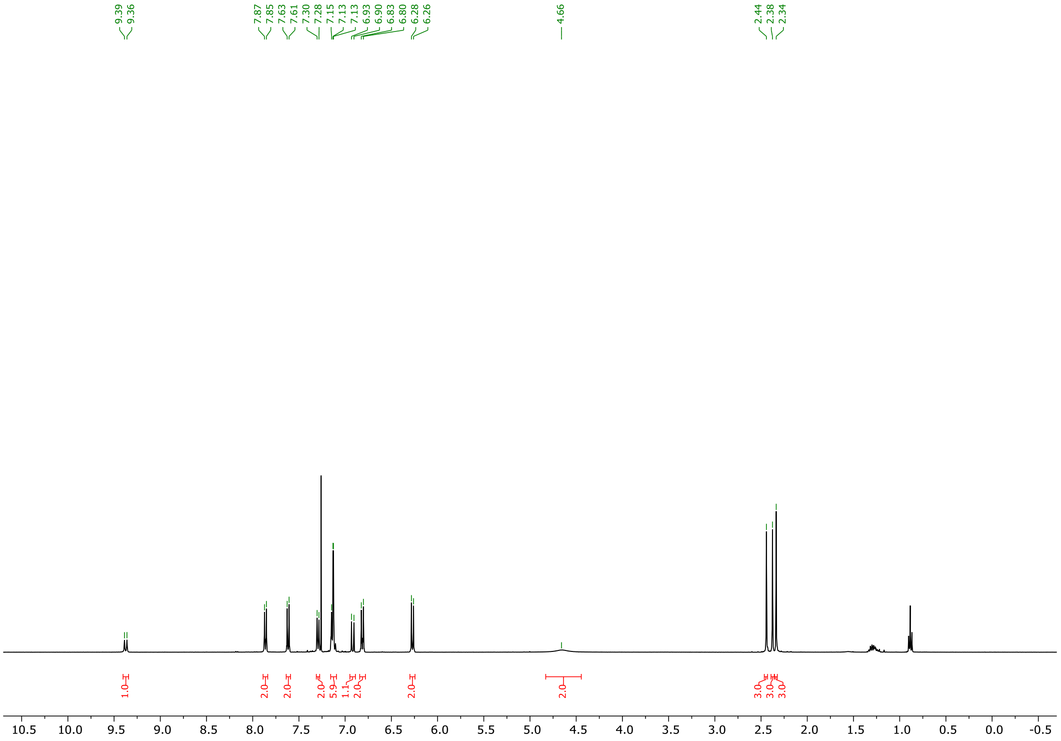

^{13}C{^{1}H}, CDCl$_3$, 101 MHz

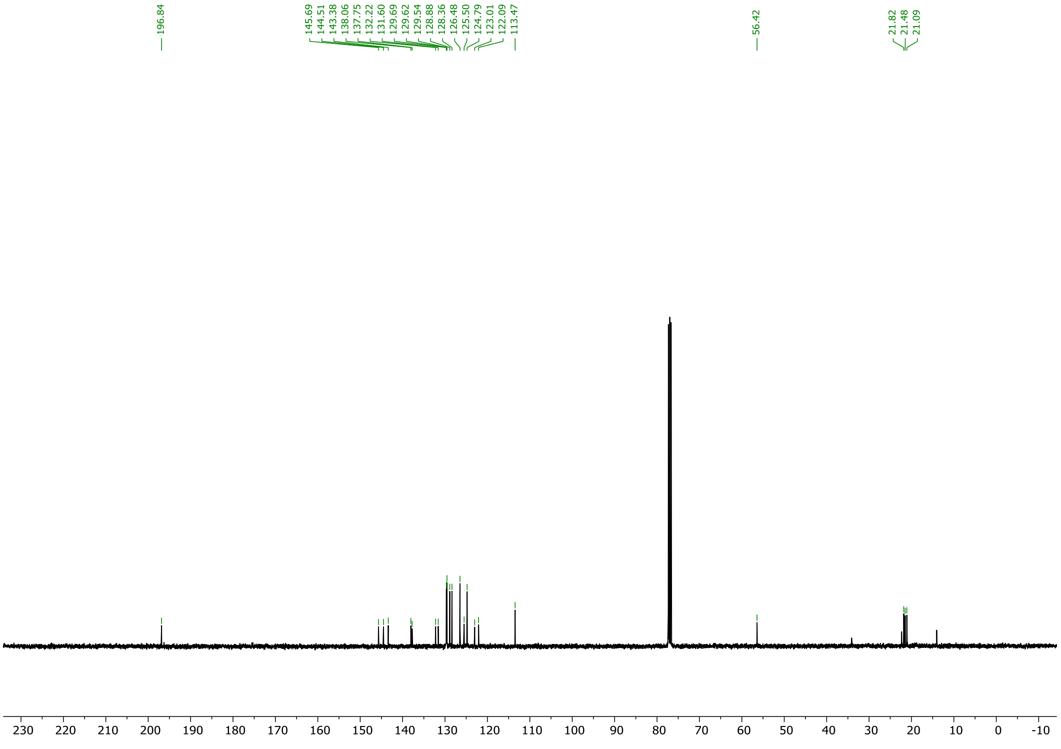

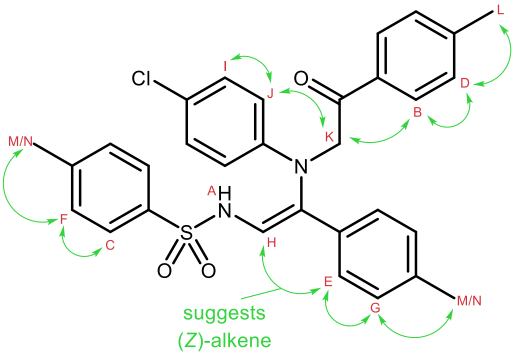

17b

$^{1}H \leftrightarrow ^{1}H$ NOESY, 500 MHz, $CDCl_3$

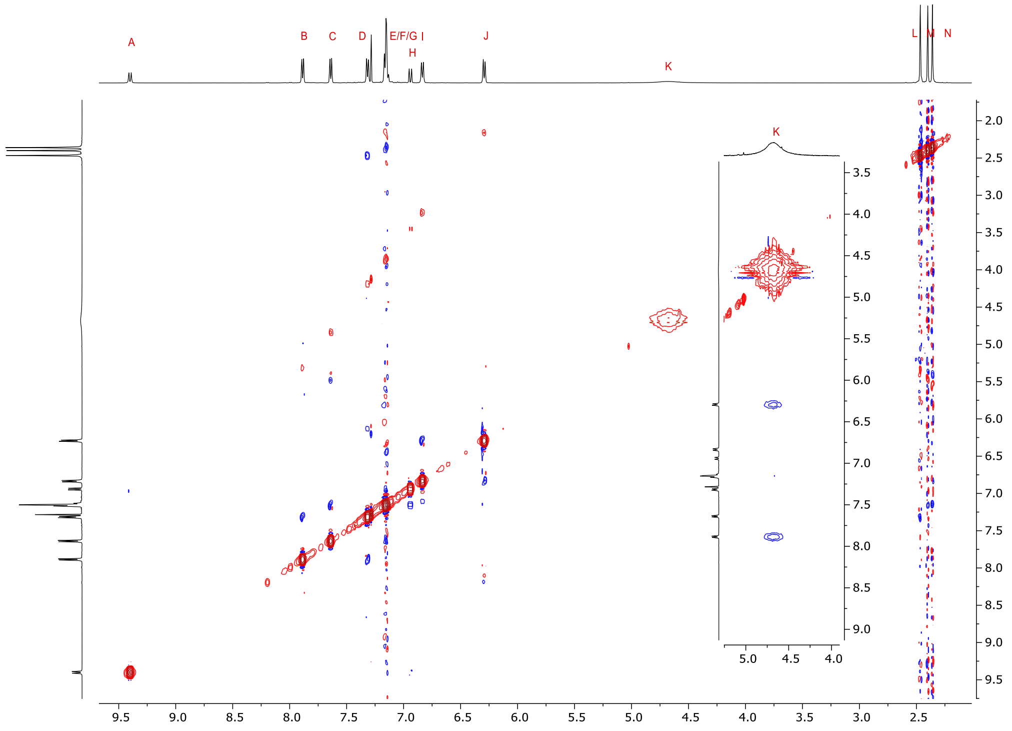

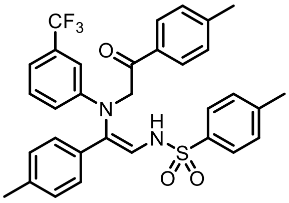

17c

^{1}H, CDCl3, 400 MHz

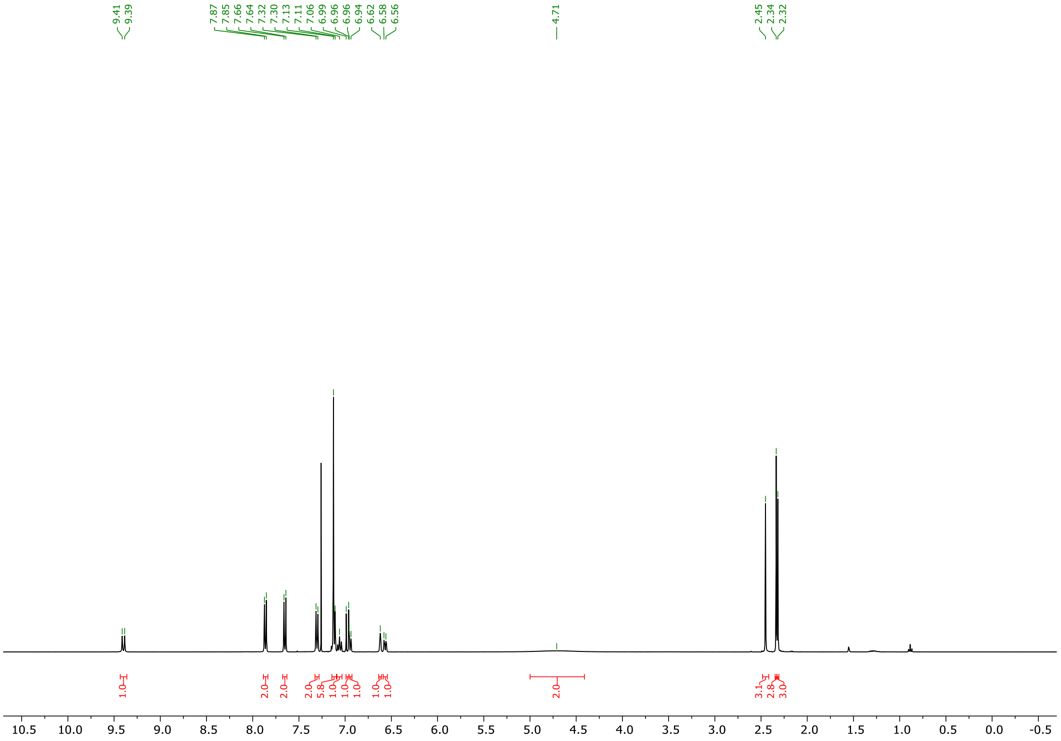

^{13}C{^{1}H}, CDCl3, 101 MHz

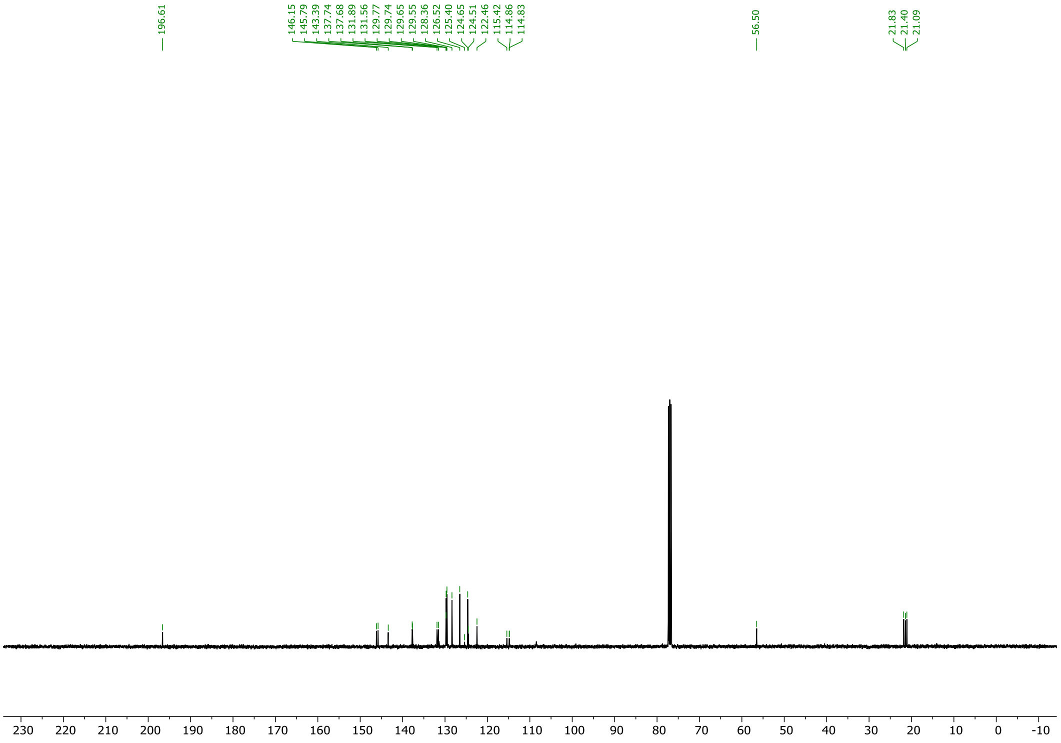

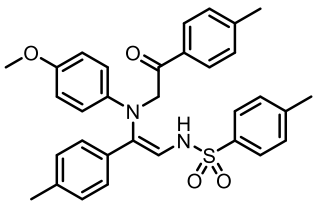

17d

^{1}H, CDCl$_3$, 500 MHz

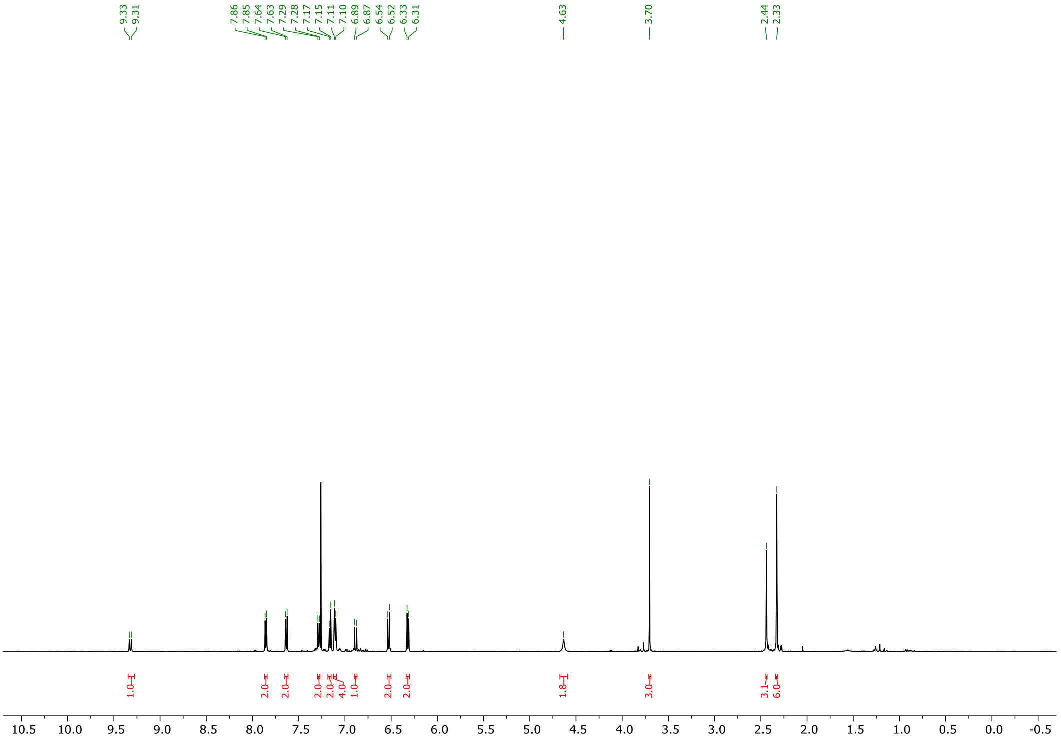

^{13}C{^{1}H}, CDCl$_3$, 101 MHz

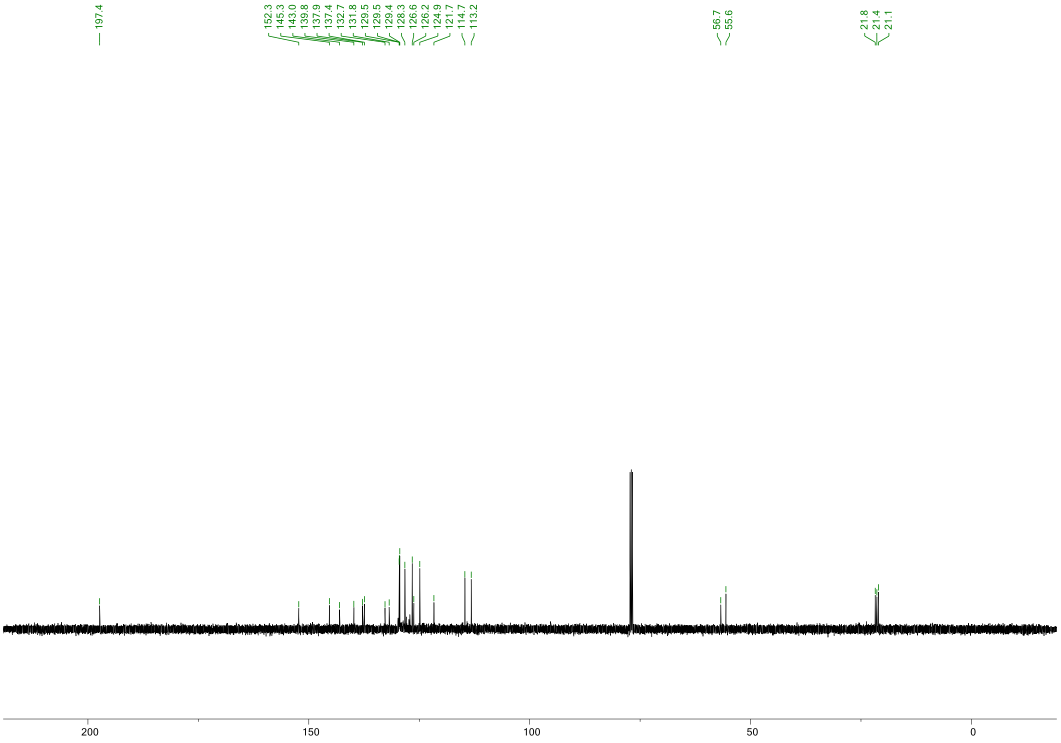

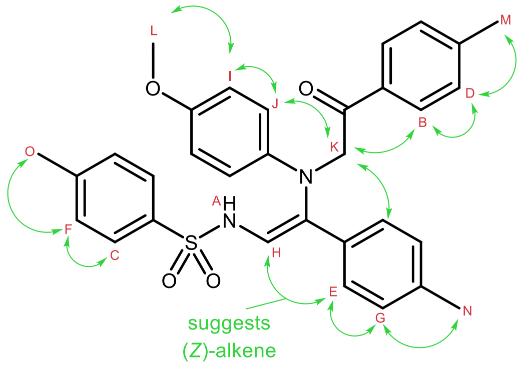

17d

^{1}H↔^{1}H NOESY, 400 MHz, CDCl$_3$

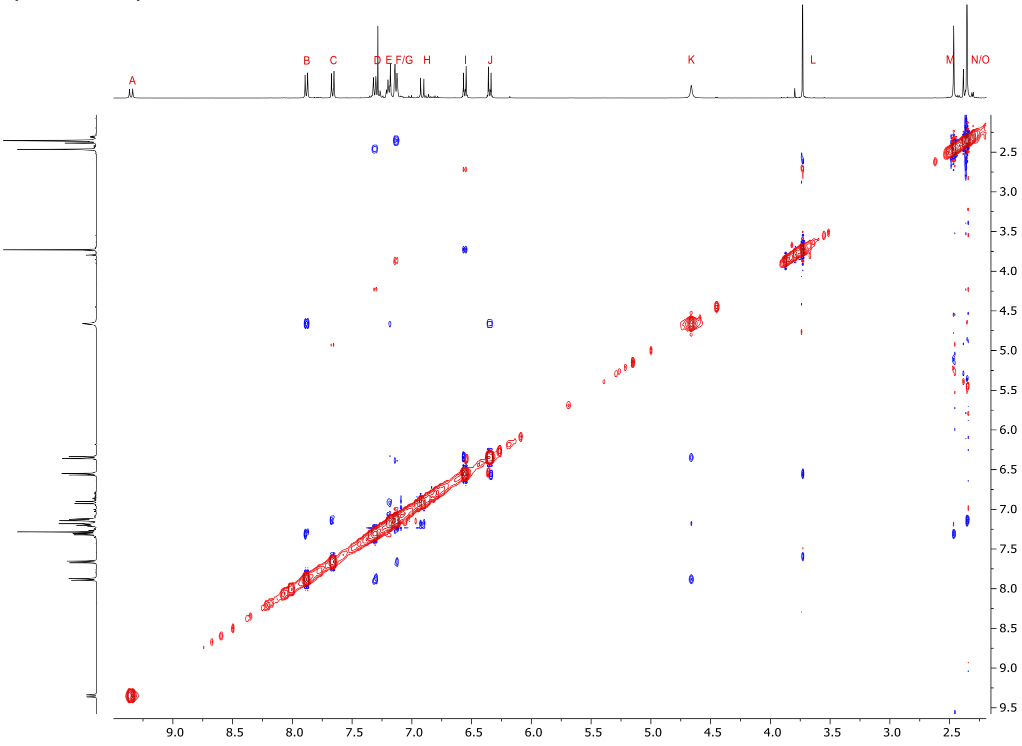

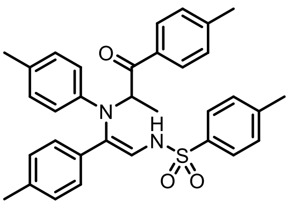

17g

^{1}H, CDCl$_3$, 500 MHz

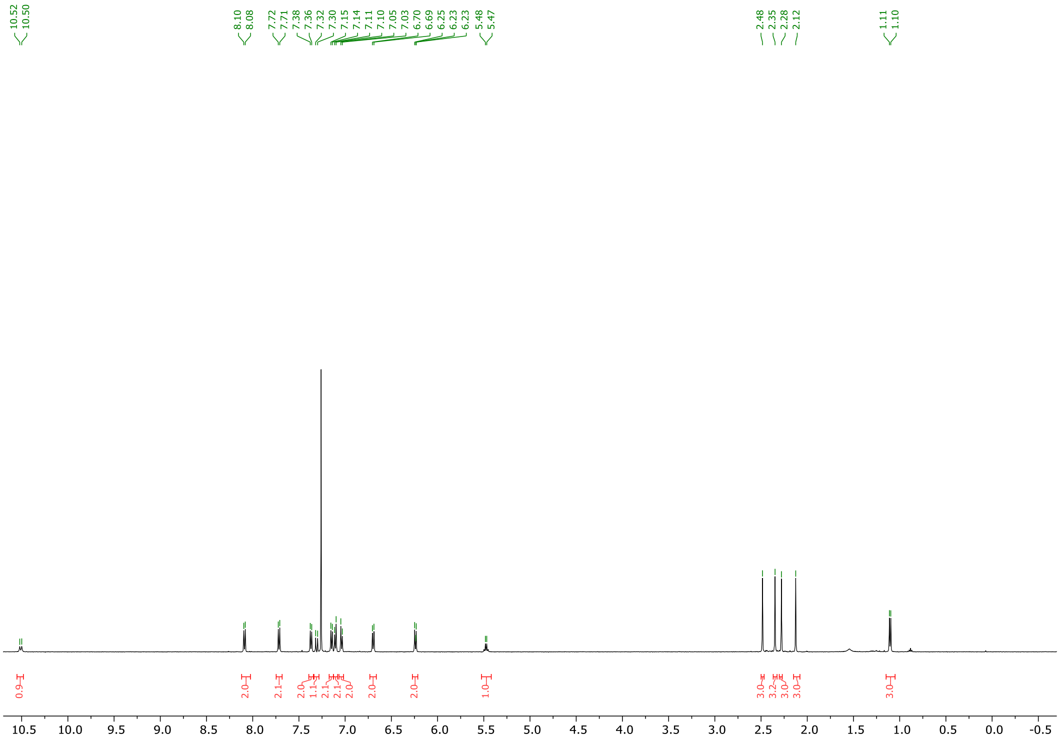

^{13}C{^{1}H}, CDCl$_3$, 101 MHz

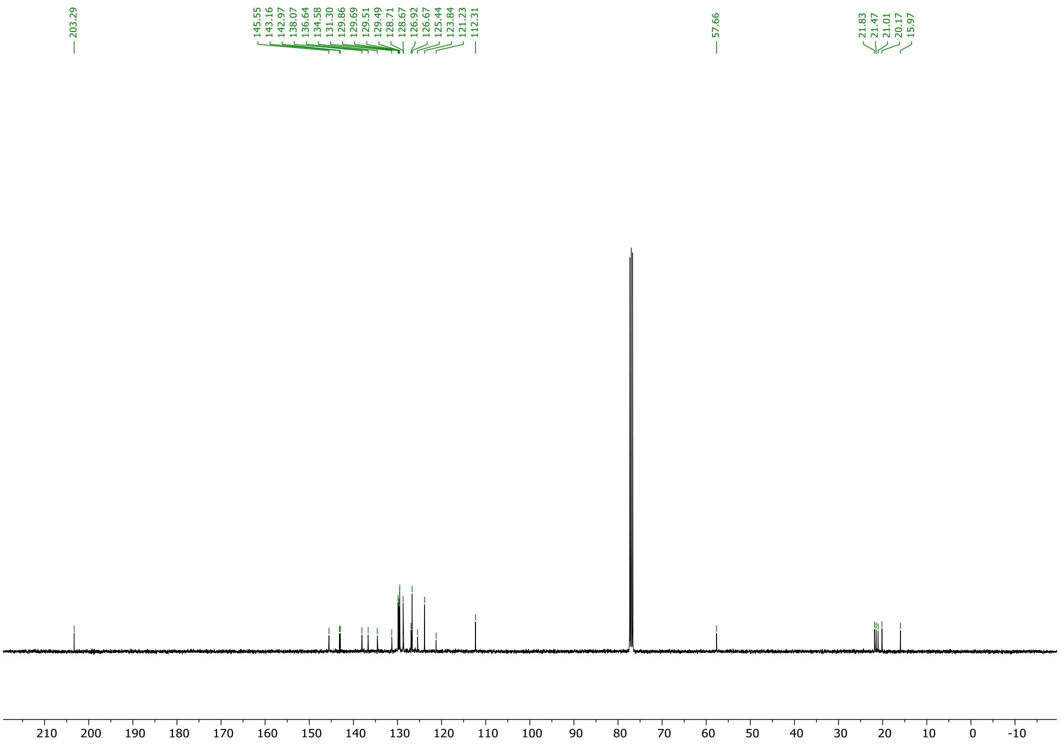

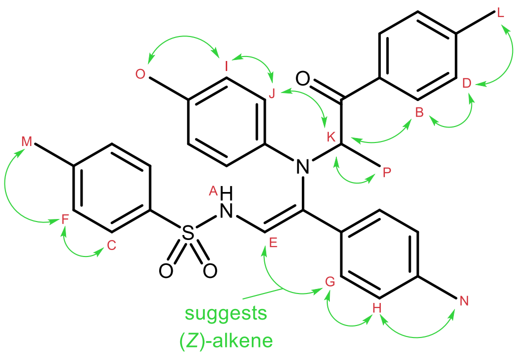

17g

^{1}H↔^{1}H NOESY, 500 MHz, $CDCl_3$

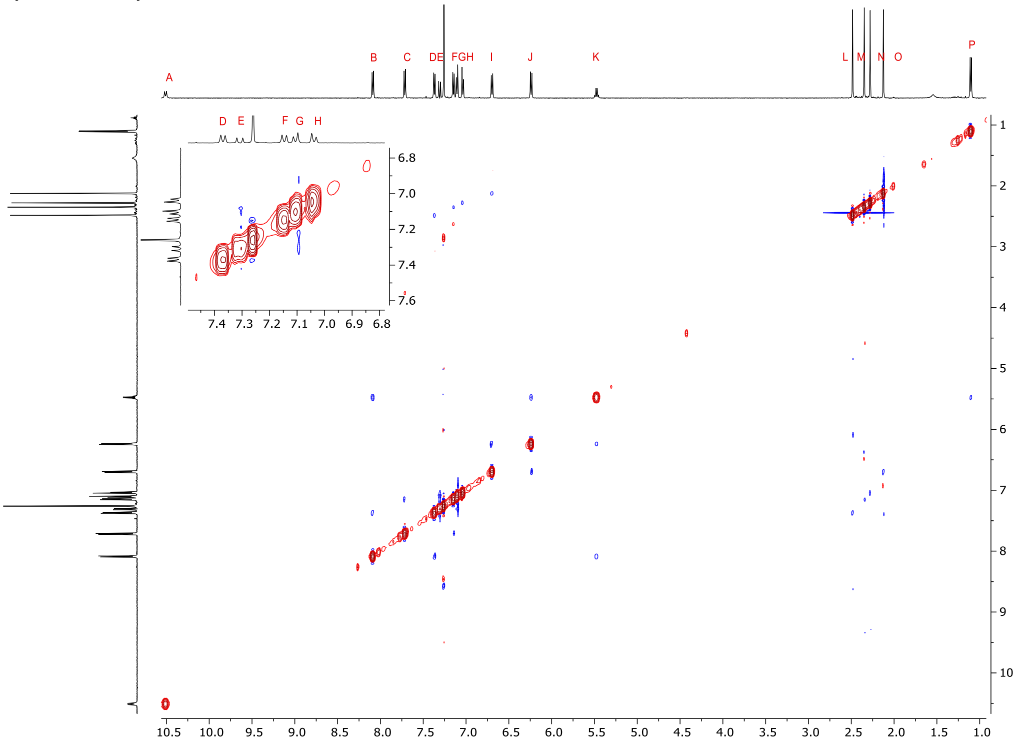

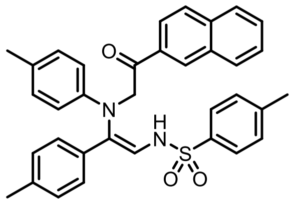

17h

^{1}H, $CDCl_3$, 400 MHz

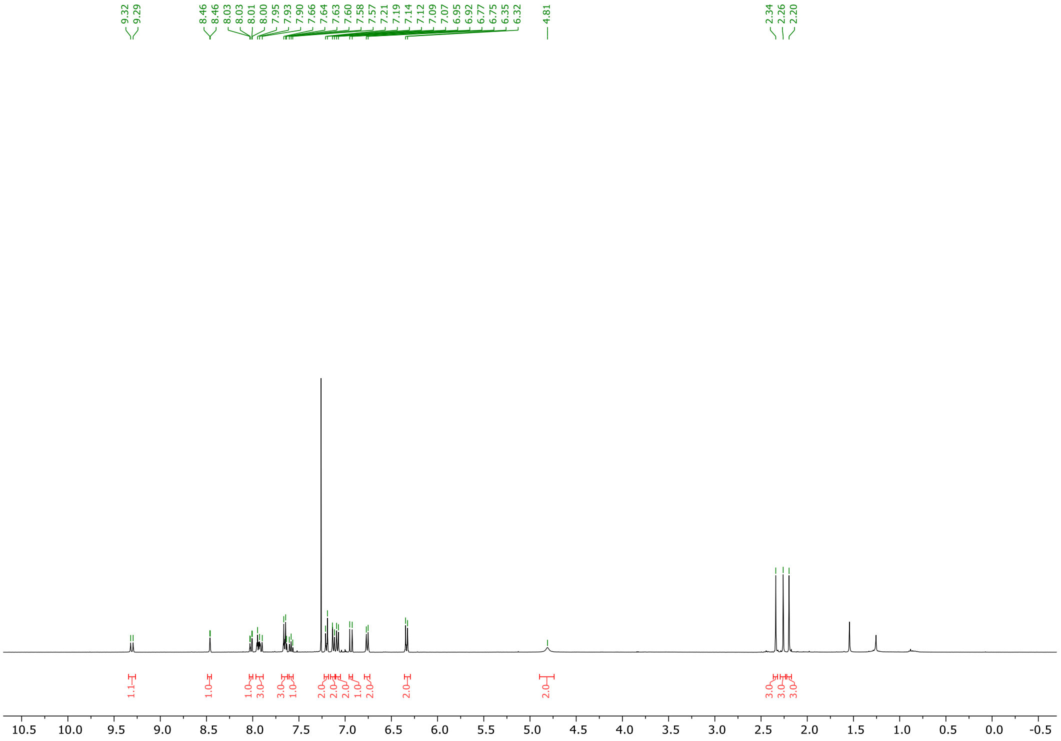

^{13}C{^{1}H}, $CDCl_3$, 101 MHz

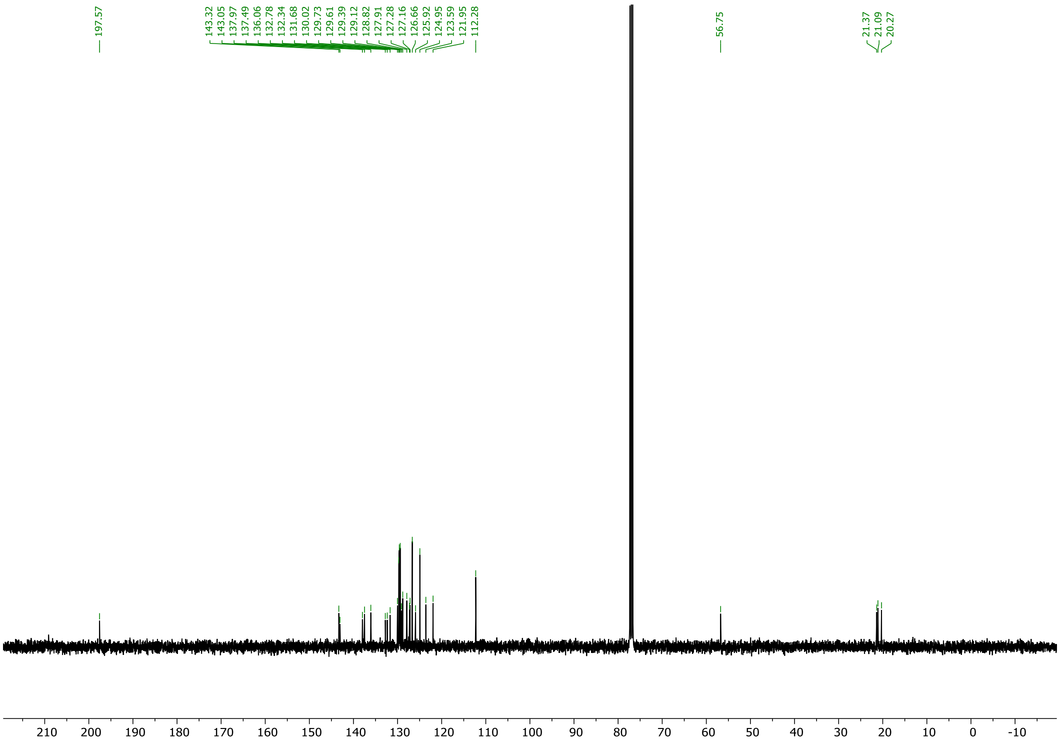

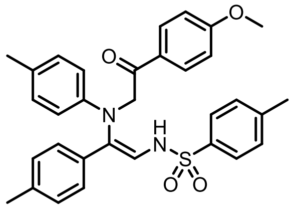

17i

^{1}H, CDCl$_3$, 400 MHz

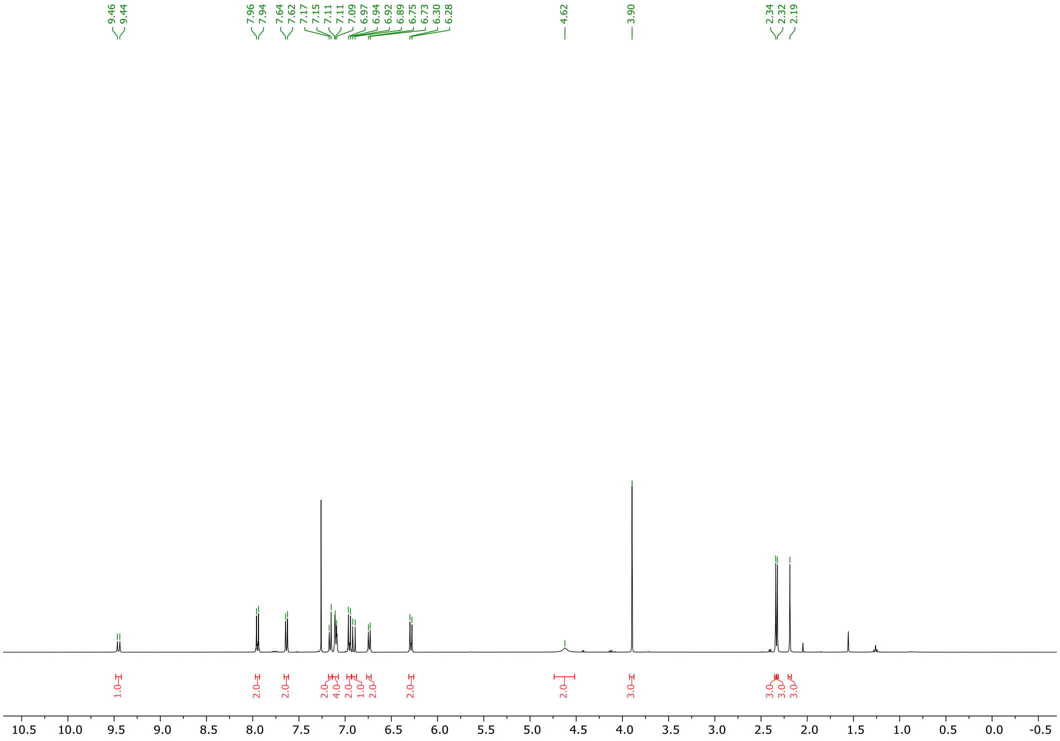

^{13}C{^{1}H}, CDCl$_3$, 101 MHz

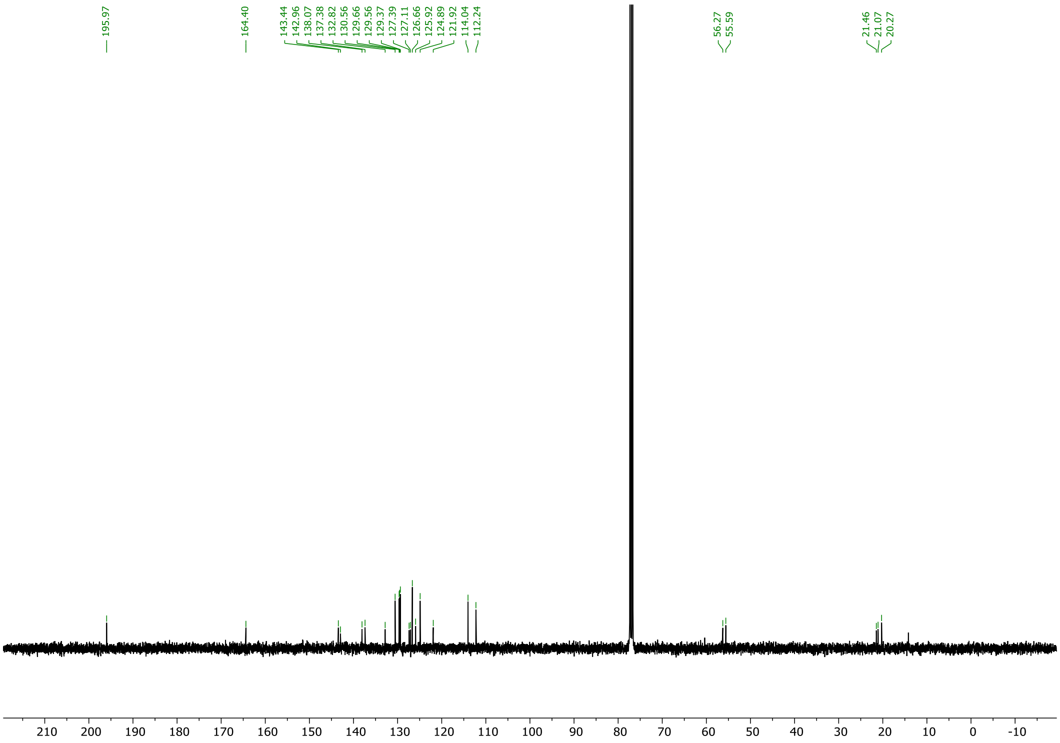

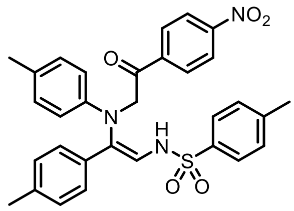

17j

^{1}H, $CDCl_3$, 400 MHz

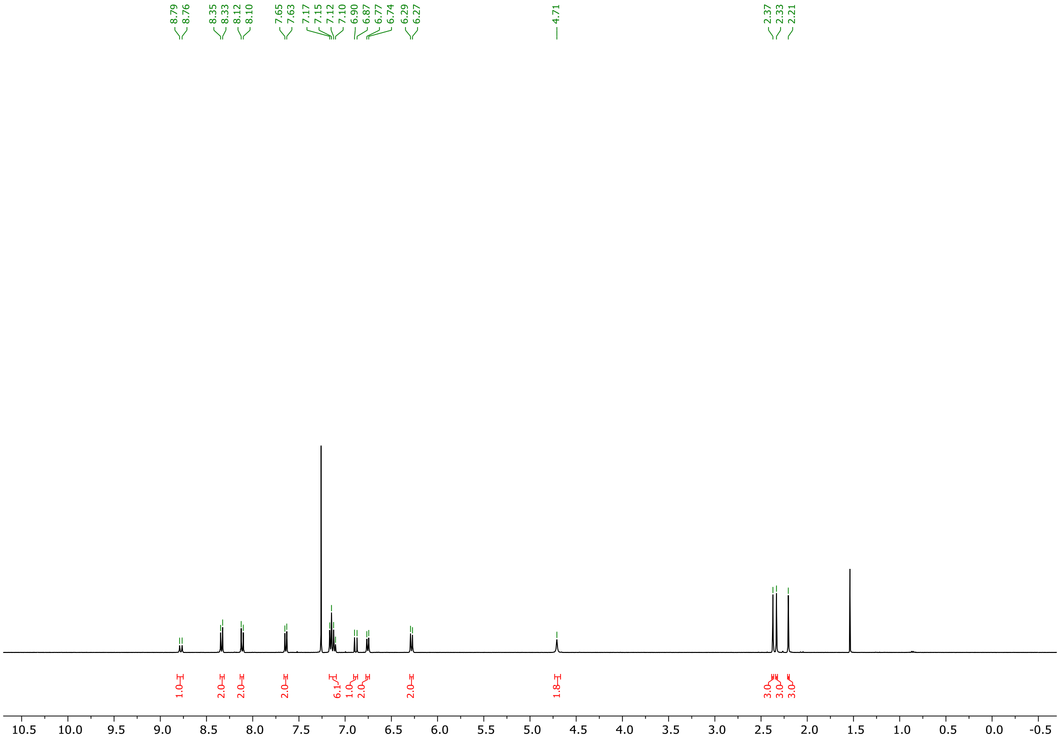

^{13}C{^{1}H}, $CDCl_3$, 101 MHz

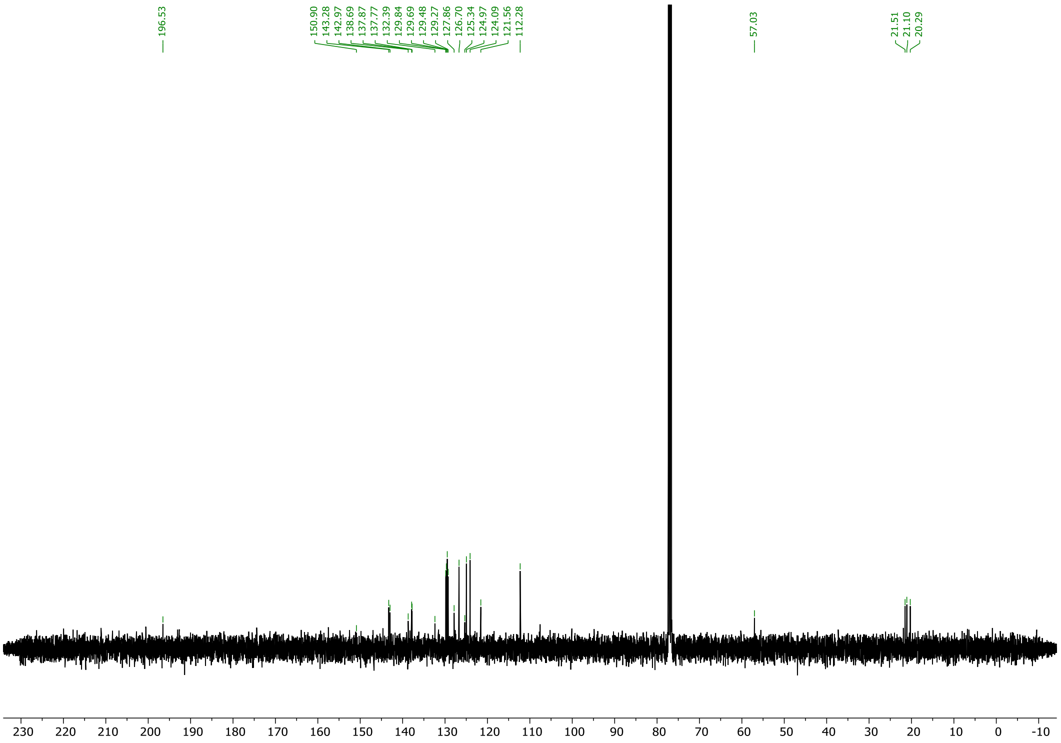

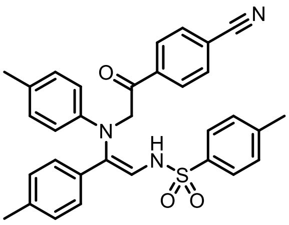

17k

^{1}H, CDCl$_3$, 500 MHz

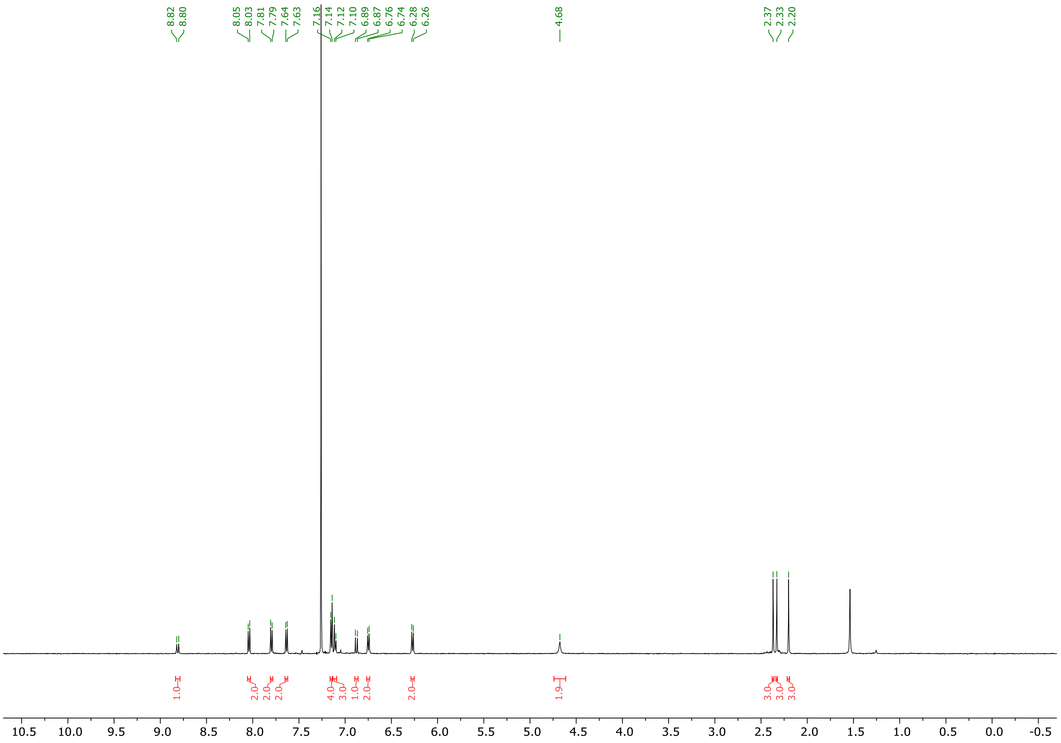

^{13}C{^{1}H}, CDCl$_3$, 101 MHz

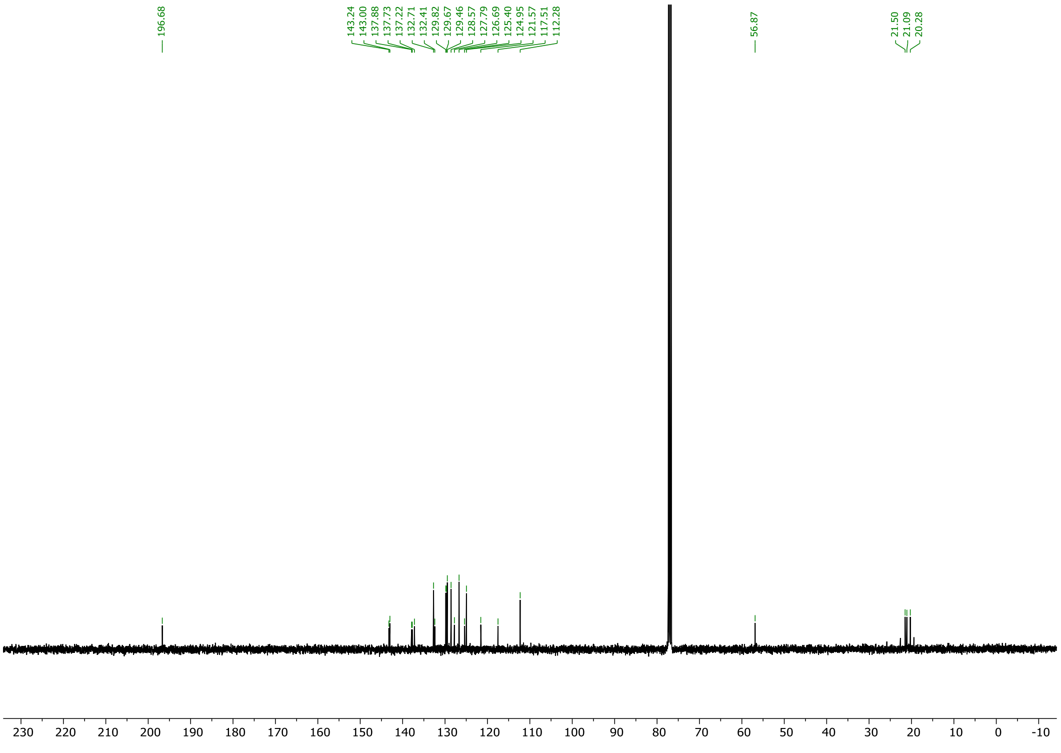

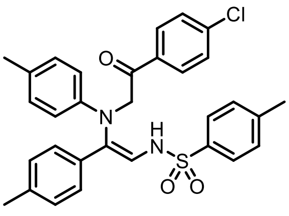

17l

^{1}H, $CDCl_3$, 400 MHz

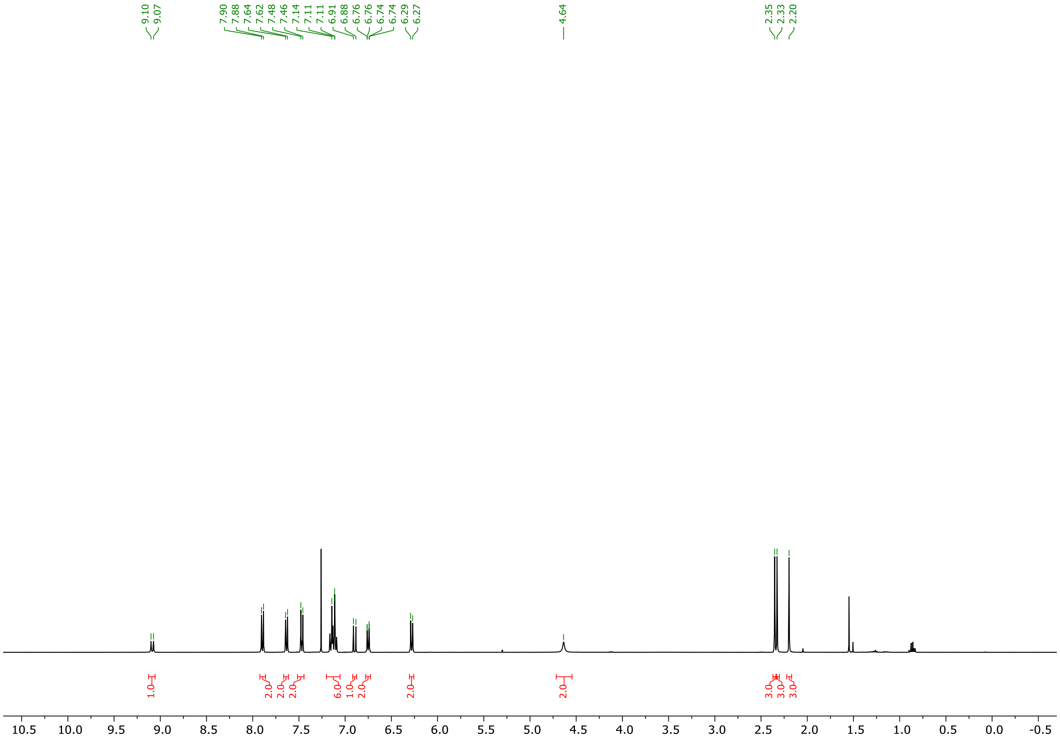

$^{13}C\{^{1}H\}$, $CDCl_3$, 101 MHz

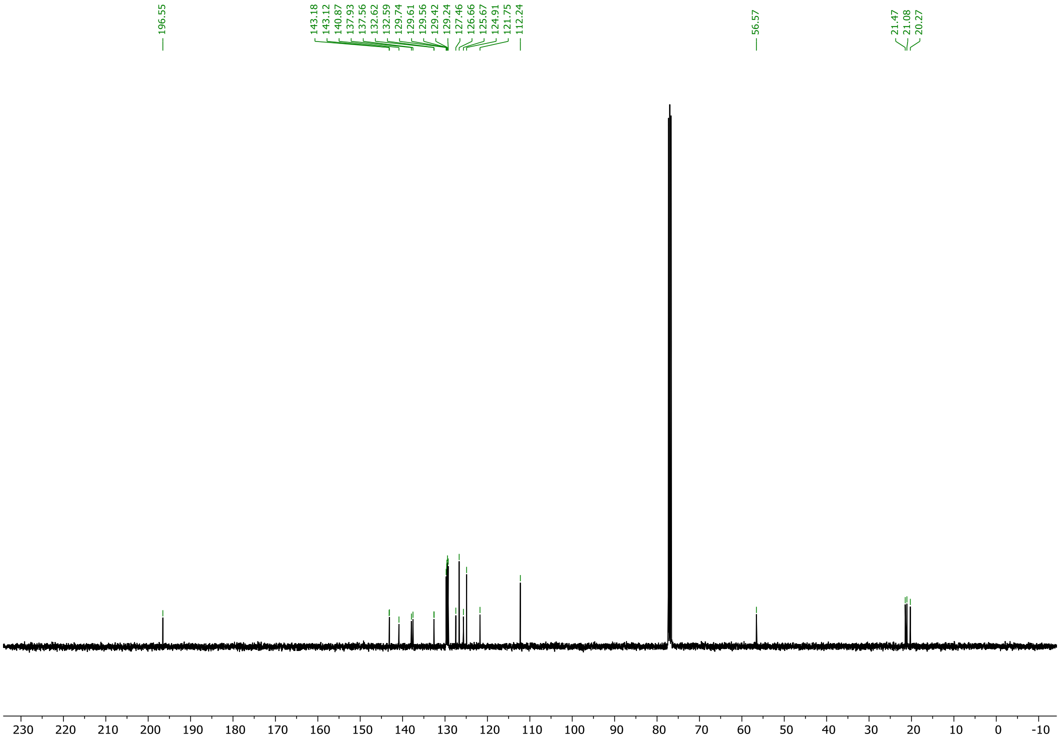

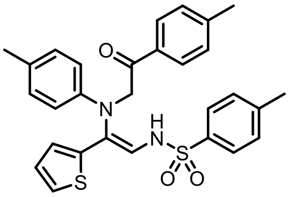

17m

^{1}H, CDCl$_3$, 400 MHz

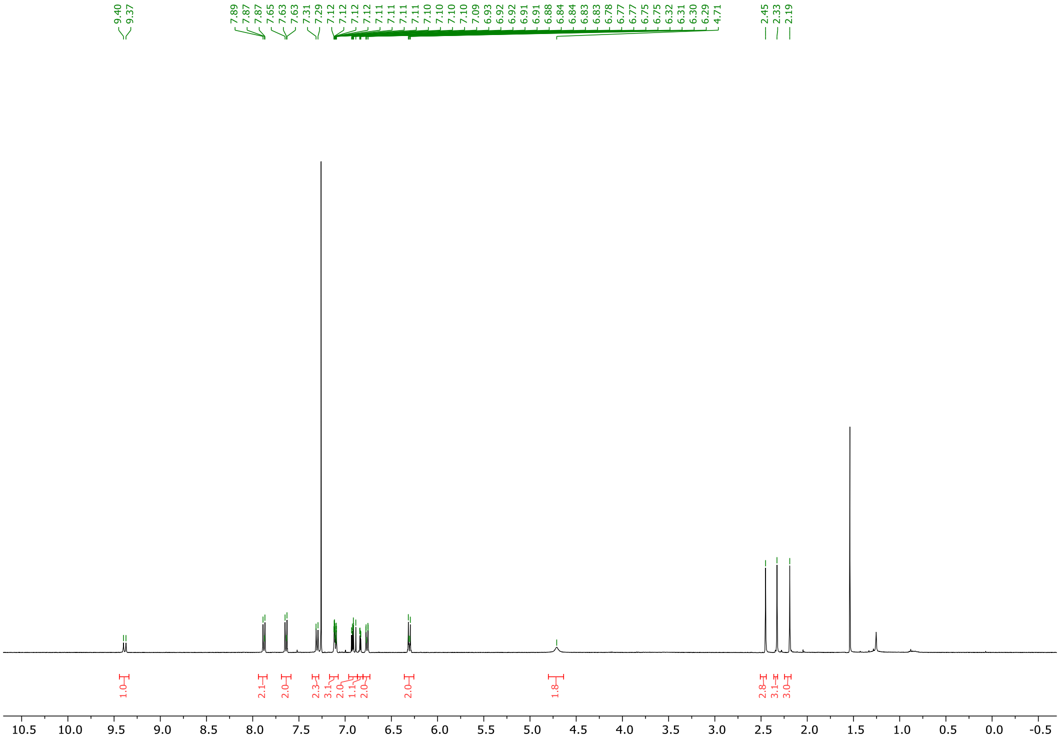

^{13}C{^{1}H}, CDCl$_3$, 101 MHz

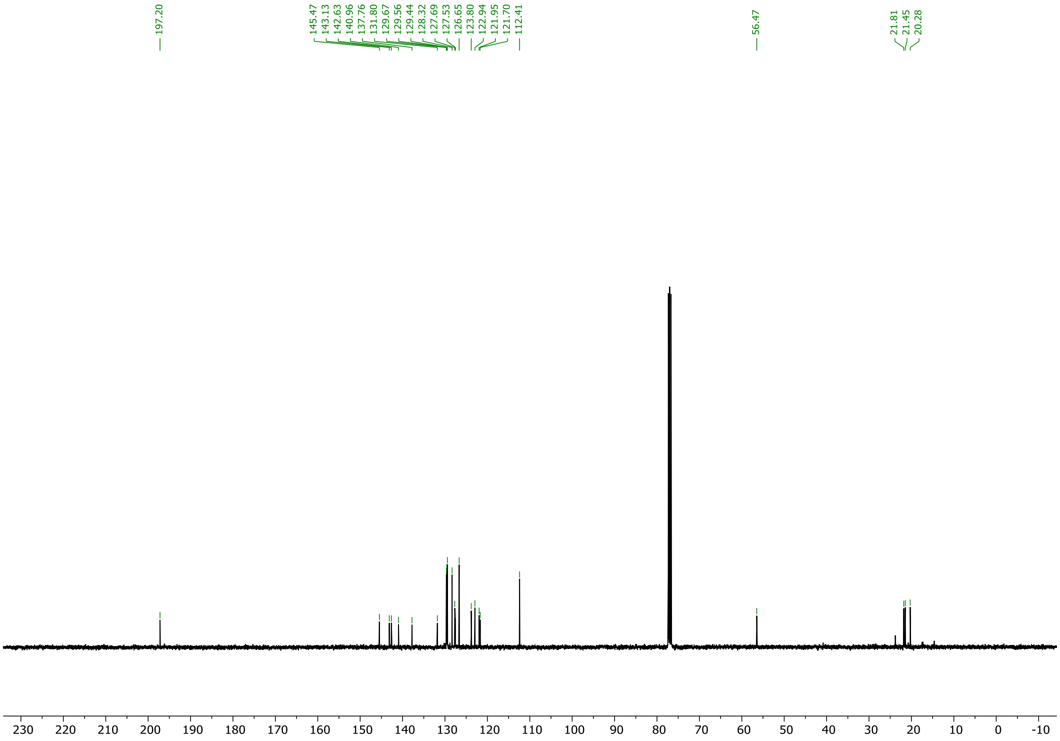

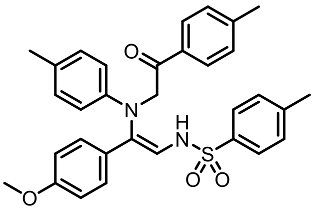

17n

^{1}H, CDCl$_3$, 400 MHz

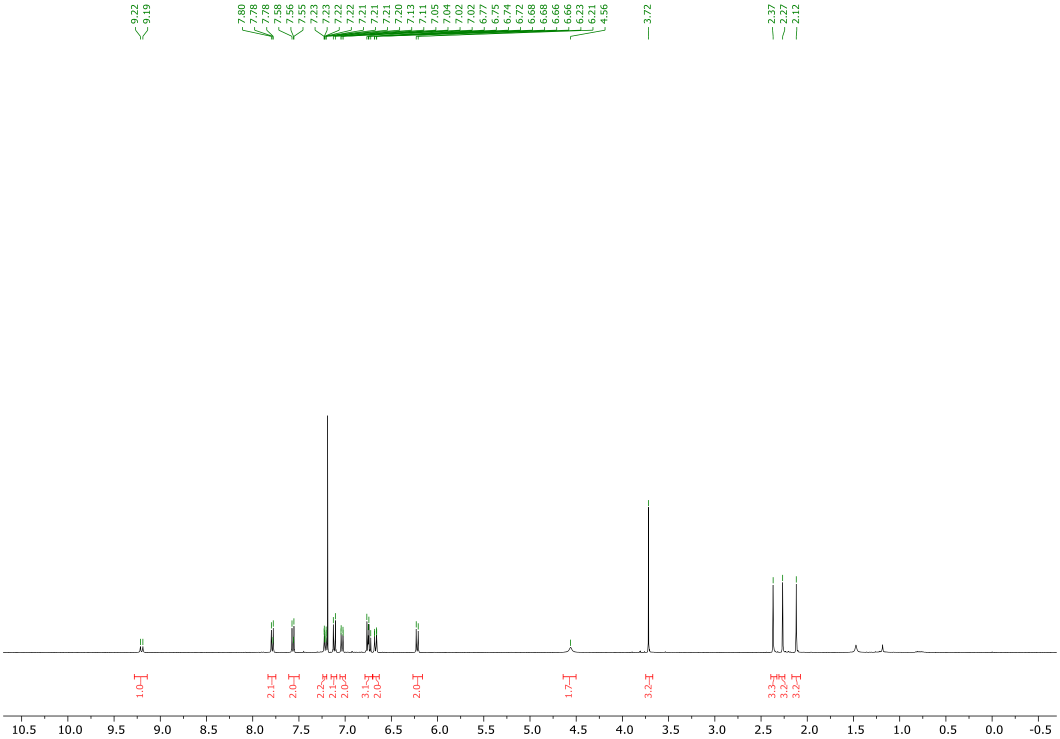

^{13}C{^{1}H}, CDCl$_3$, 101 MHz

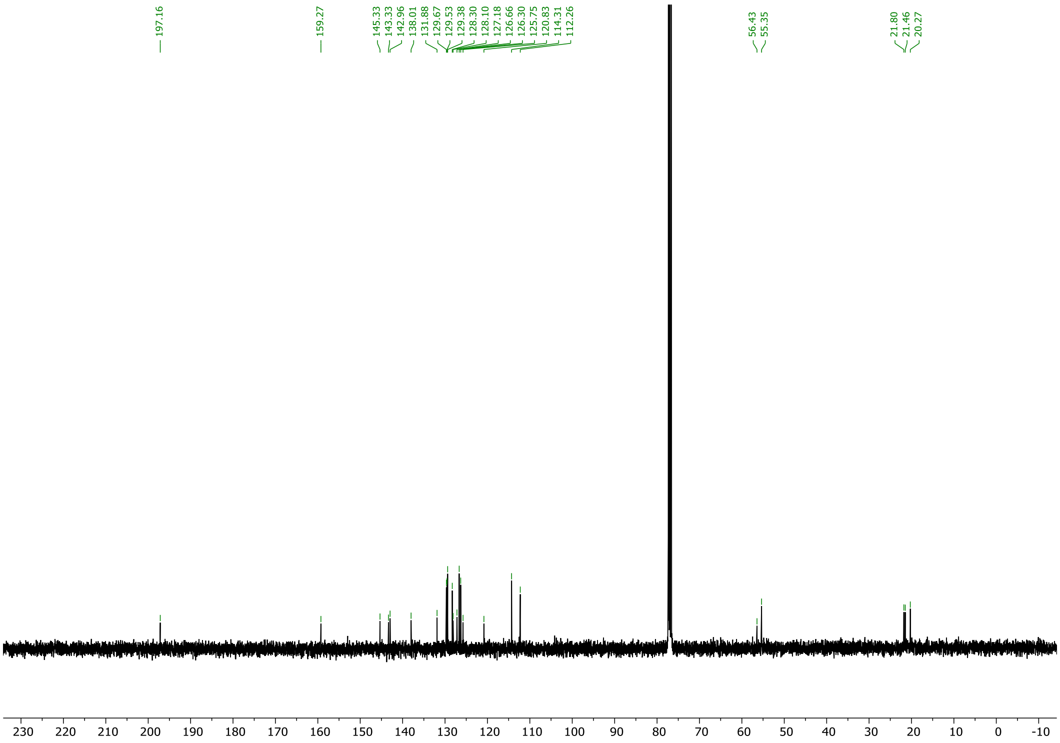

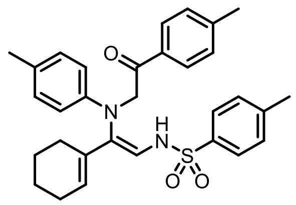

17o

^{1}H, CDCl$_3$, 400 MHz

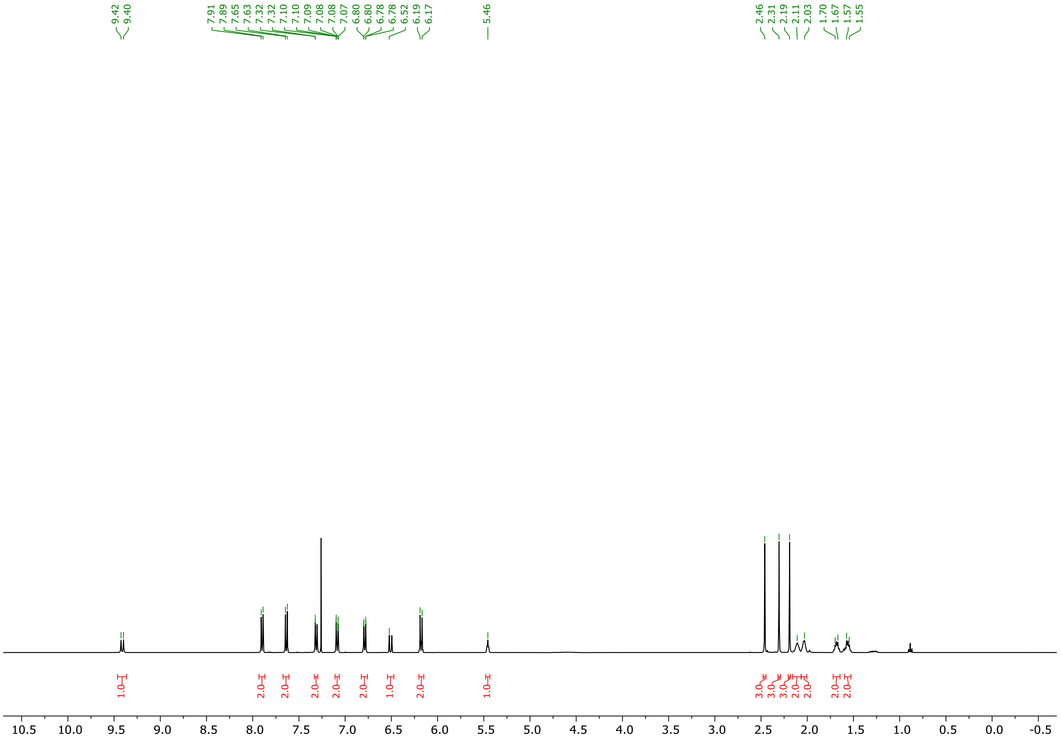

^{13}C{^{1}H}, CDCl$_3$, 101 MHz

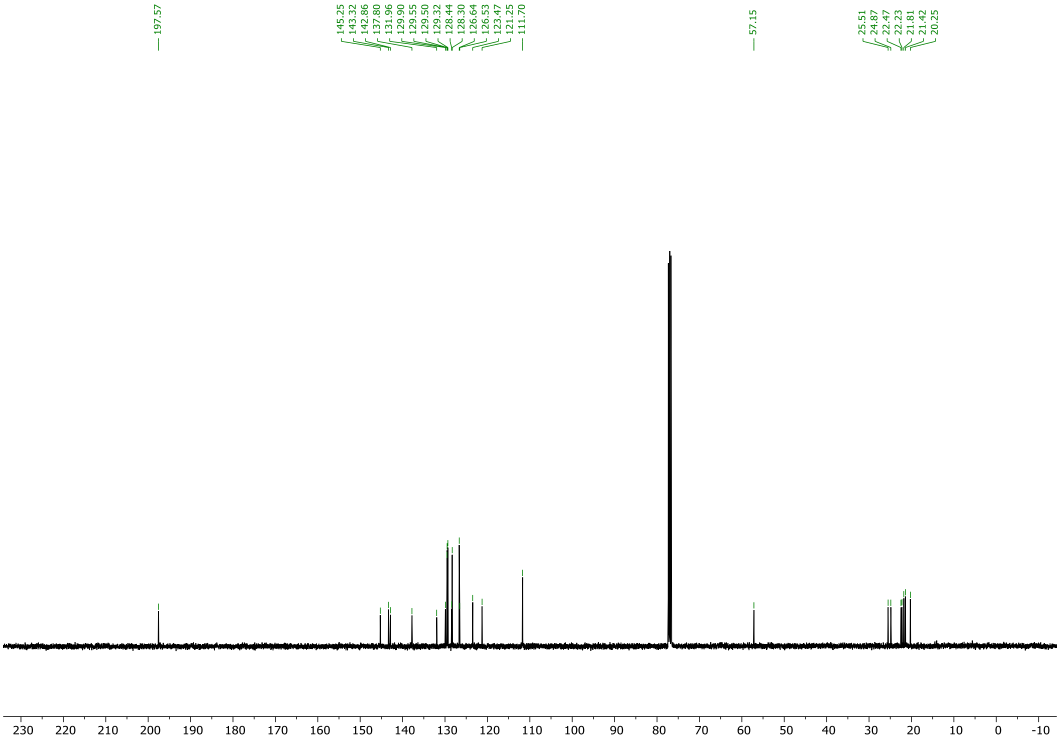

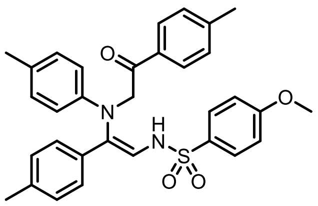

17p

^{1}H, CDCl$_3$, 400 MHz

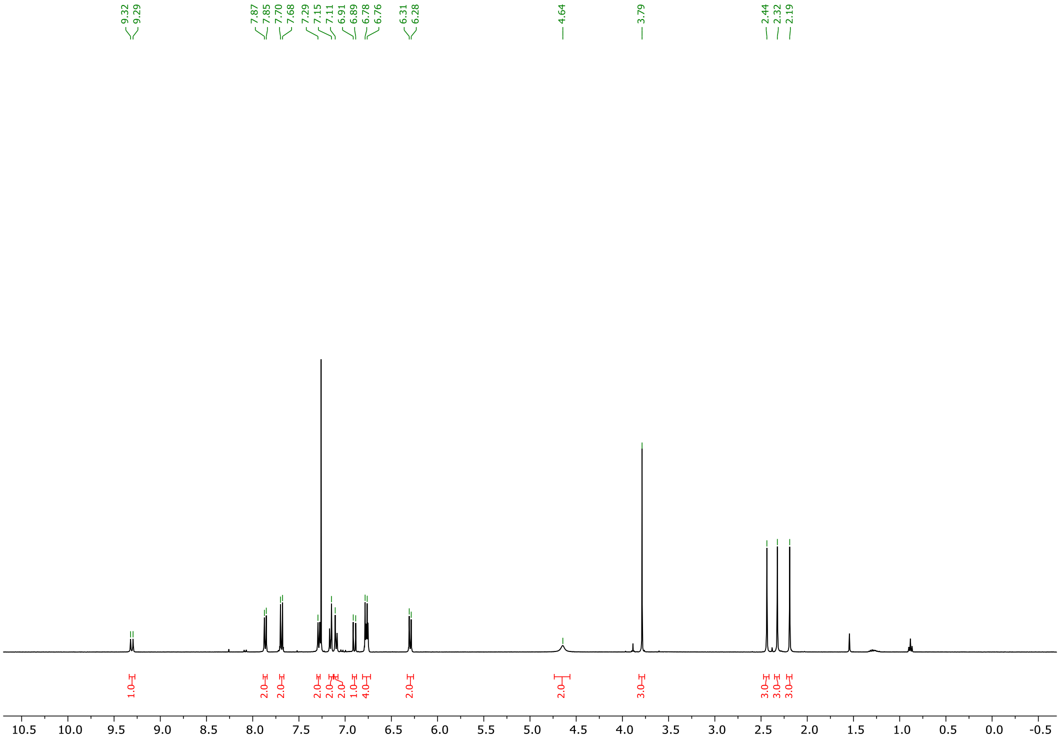

^{13}C{^{1}H}, CDCl$_3$, 101 MHz

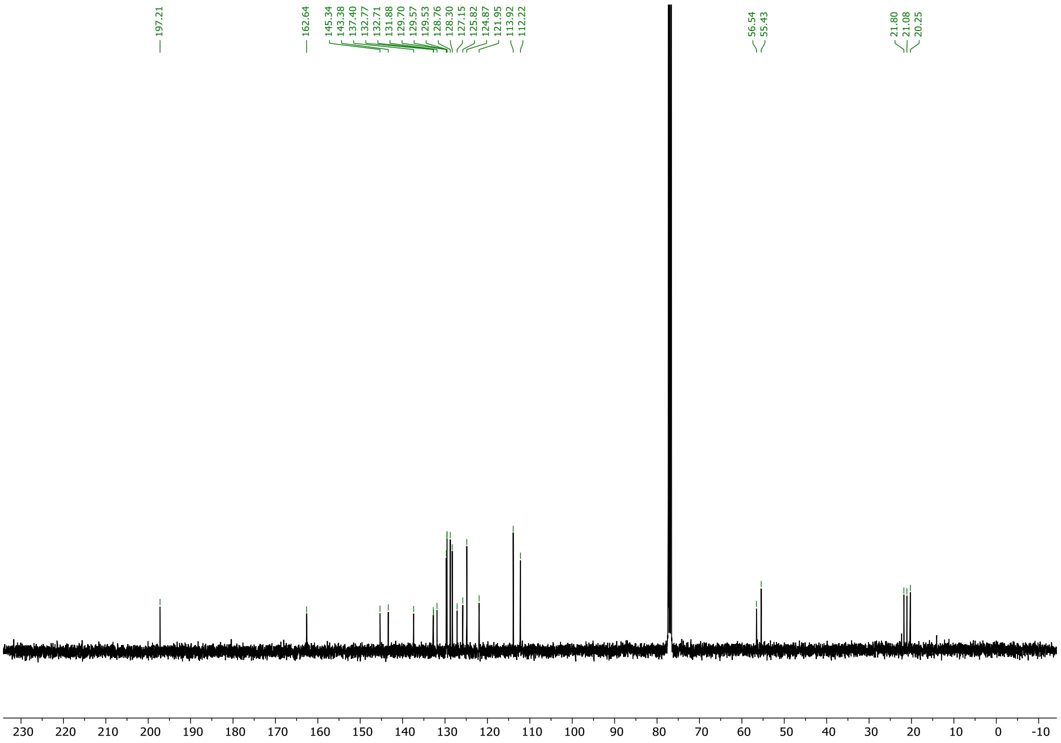

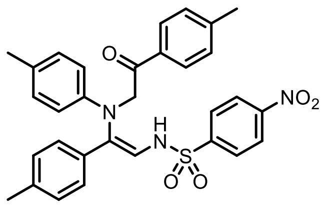

17q

^{1}H, $CDCl_3$, 400 MHz

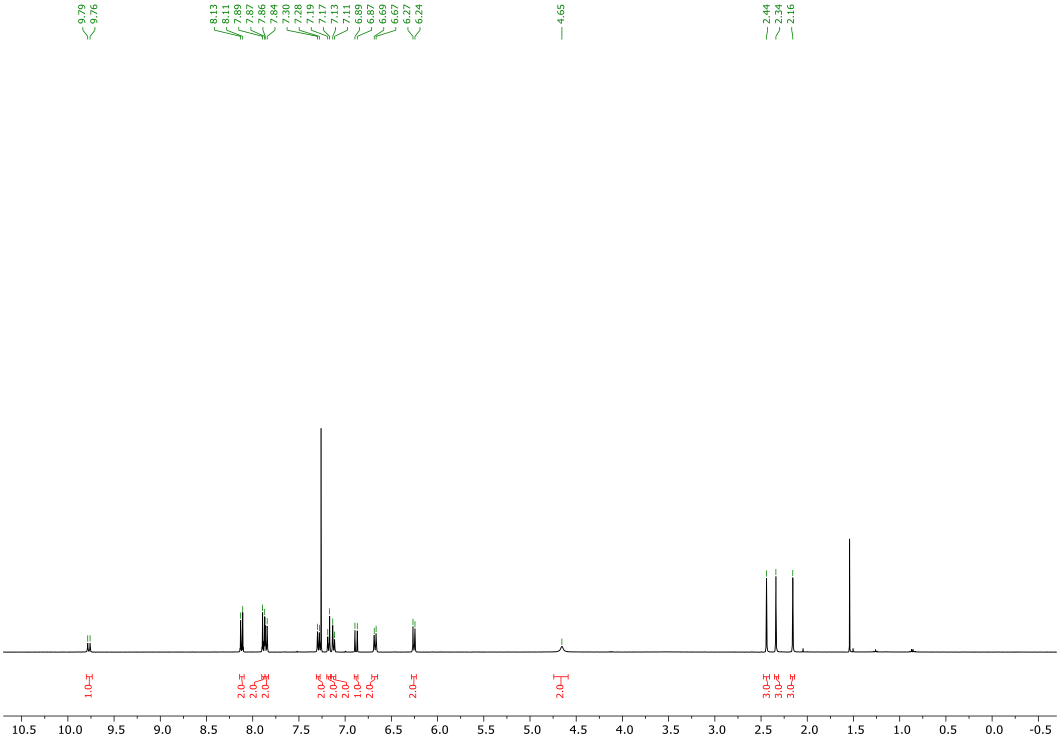

$^{13}C\{^1H\}$, $CDCl_3$, 101 MHz

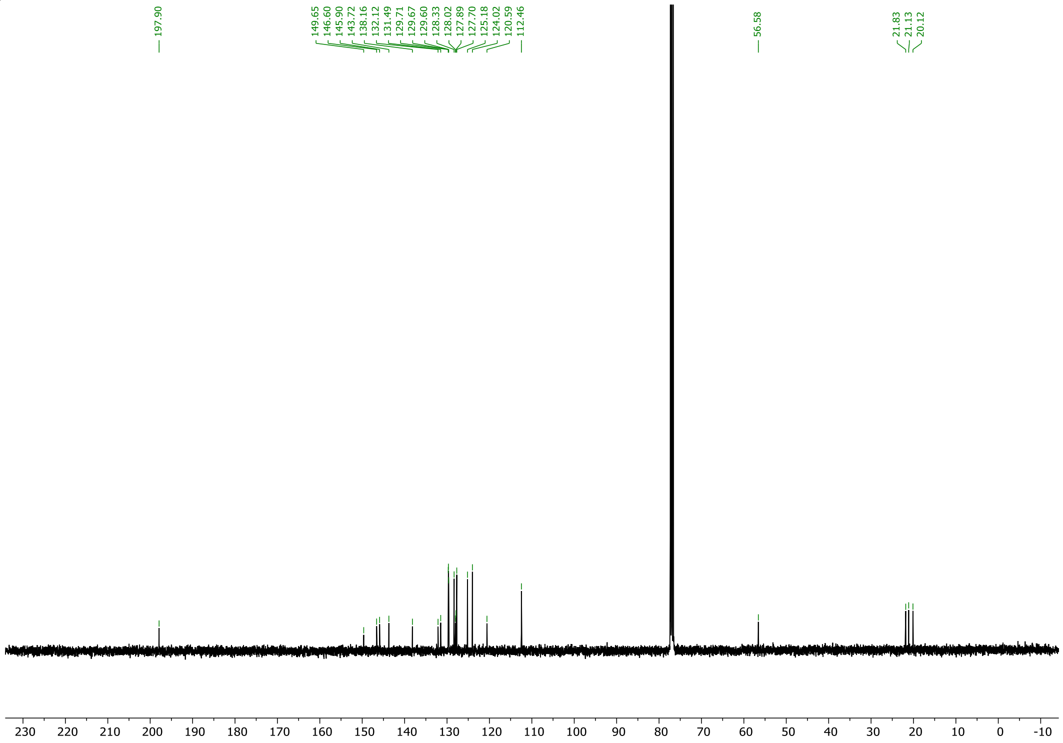

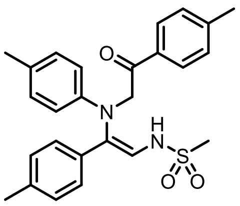

17r

¹H, CDCl₃, 400 MHz

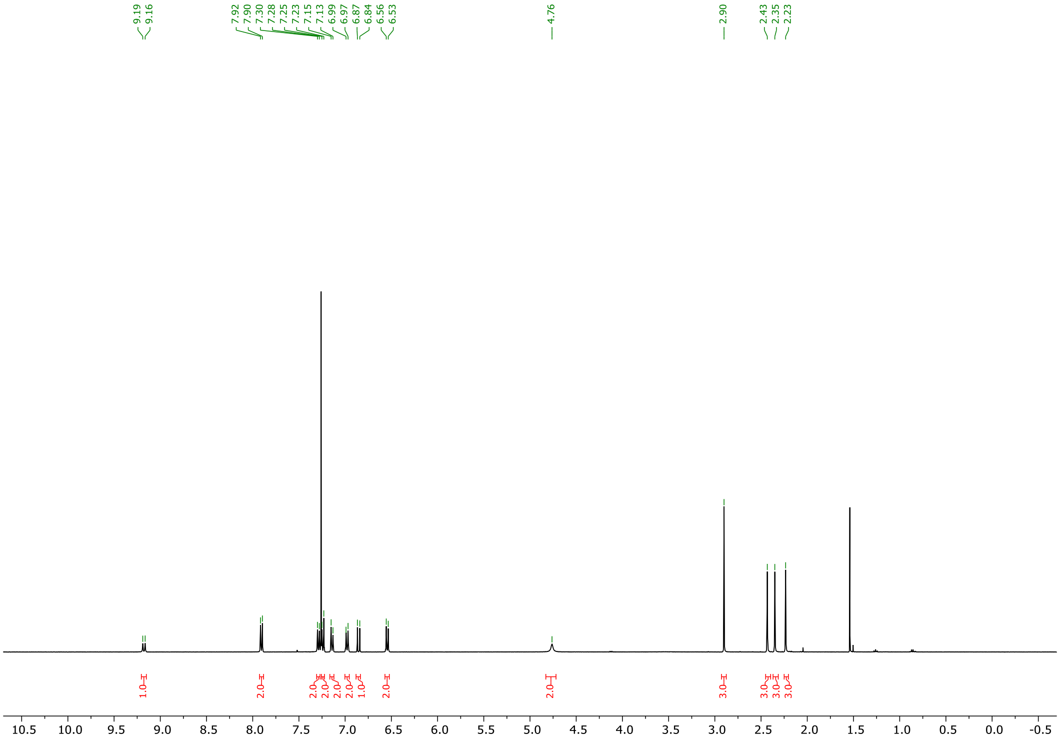

¹³C{¹H}, CDCl₃, 101 MHz

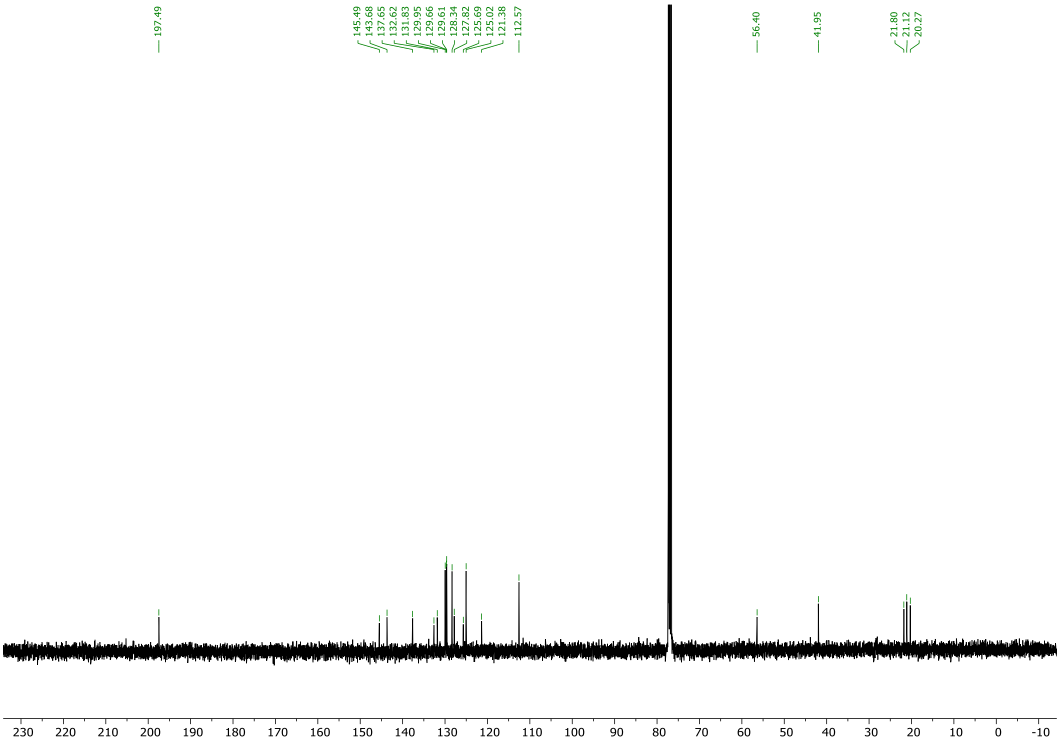

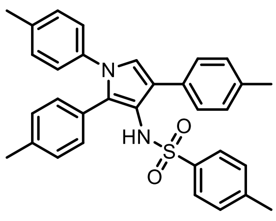

18a

^{1}H, CDCl$_3$, 500 MHz

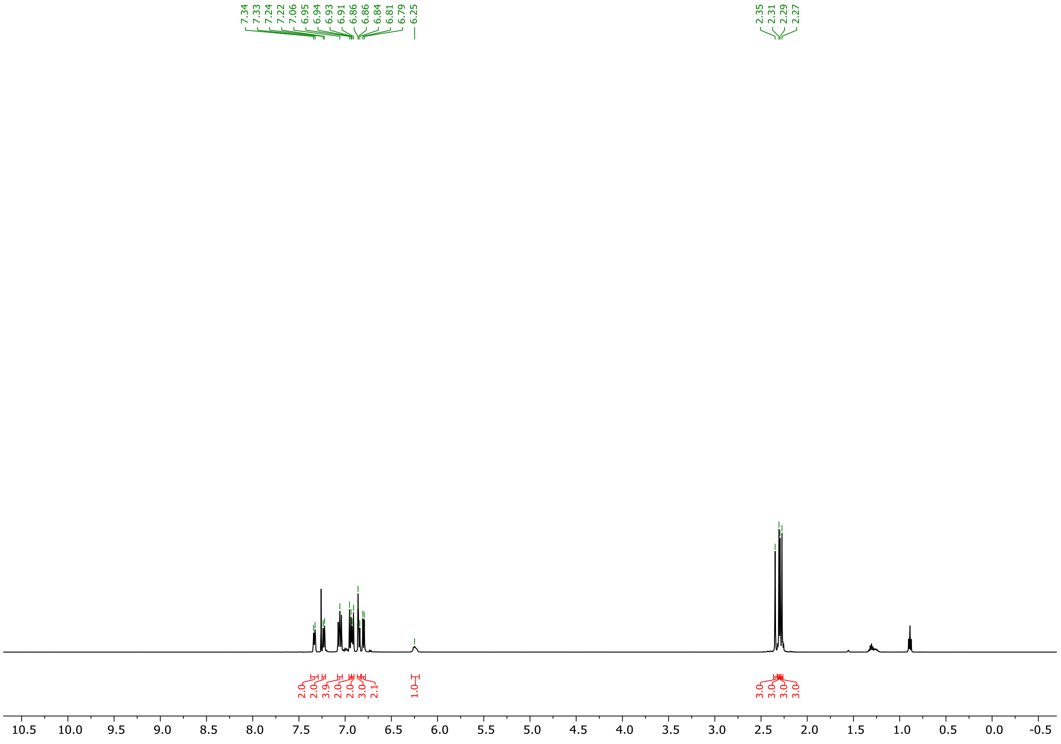

^{13}C{^{1}H}, CDCl$_3$, 126 MHz

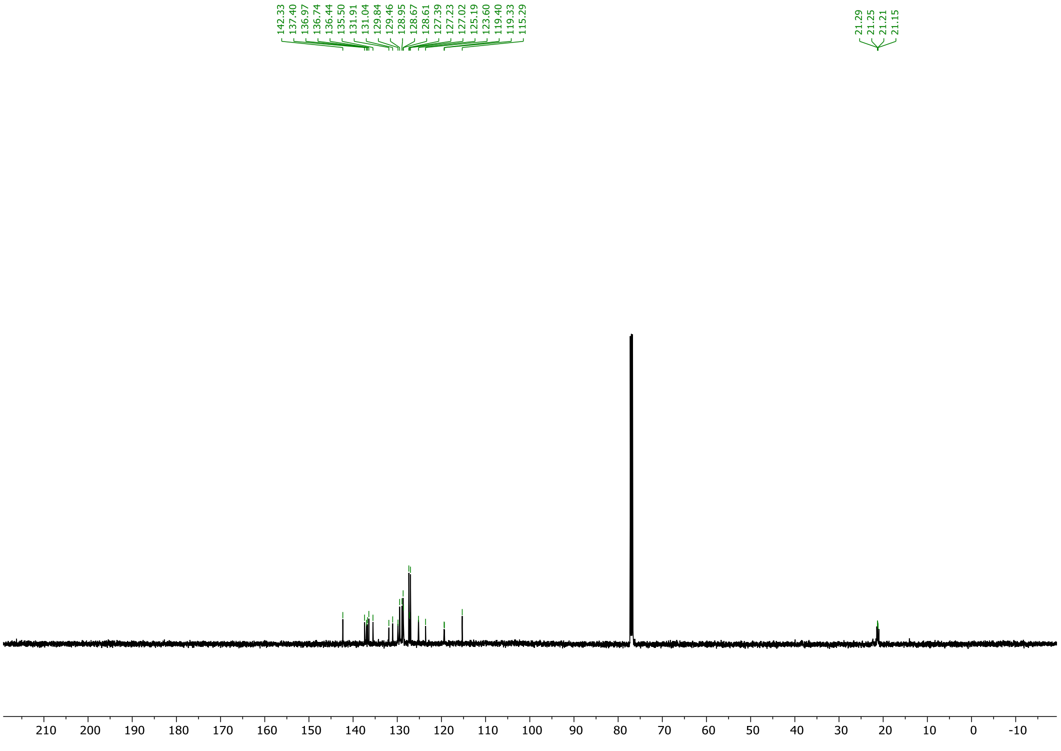

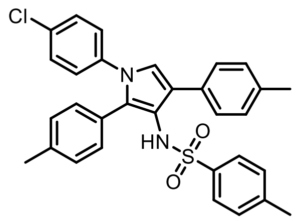

18b

^{1}H, $CDCl_3$, 400 MHz

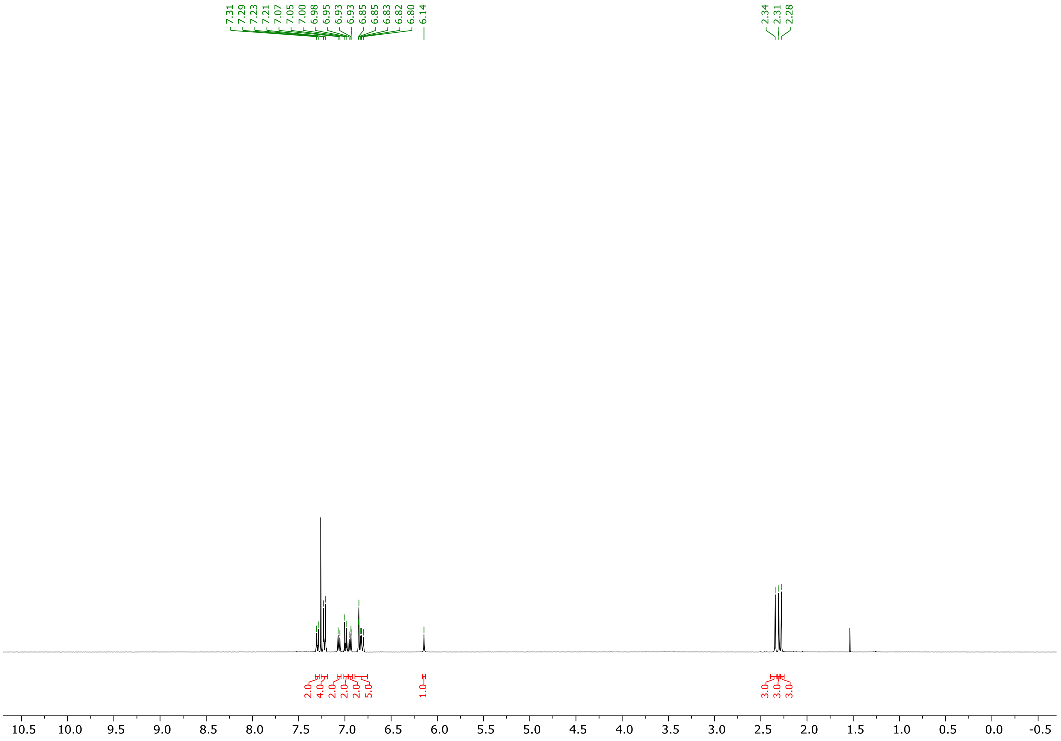

$^{13}C\{^{1}H\}$, $CDCl_3$, 101 MHz

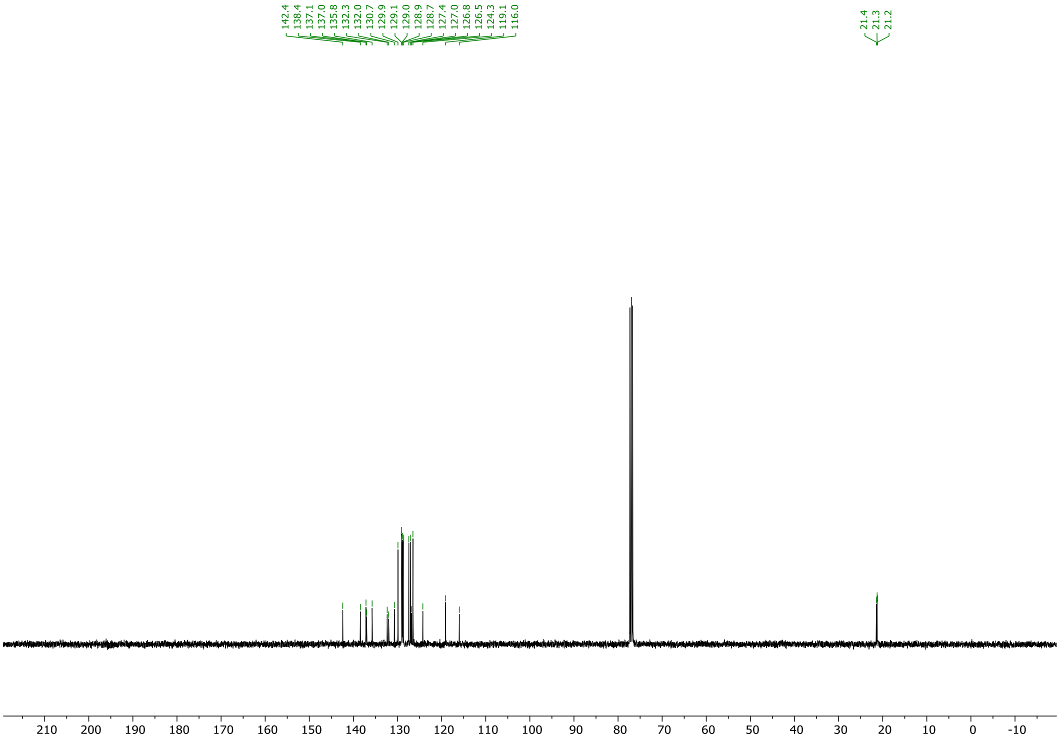

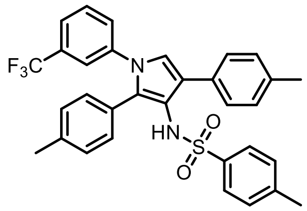

18c

^{1}H, $CDCl_3$, 400 MHz

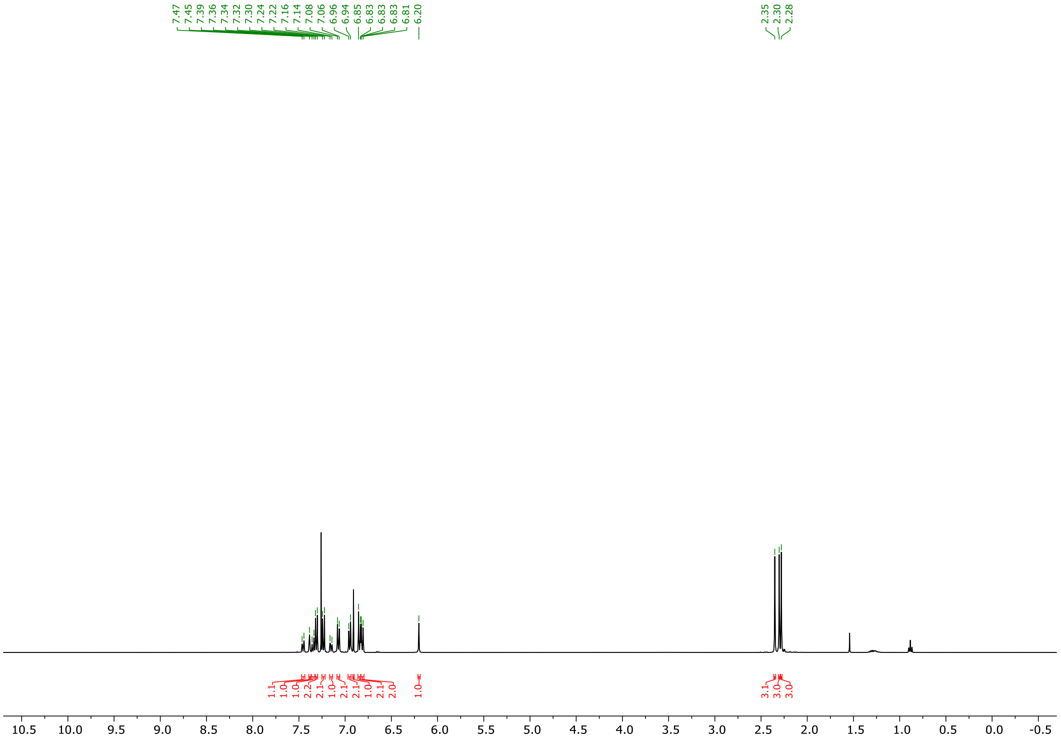

$^{13}C\{^{1}H\}$, $CDCl_3$, 101 MHz

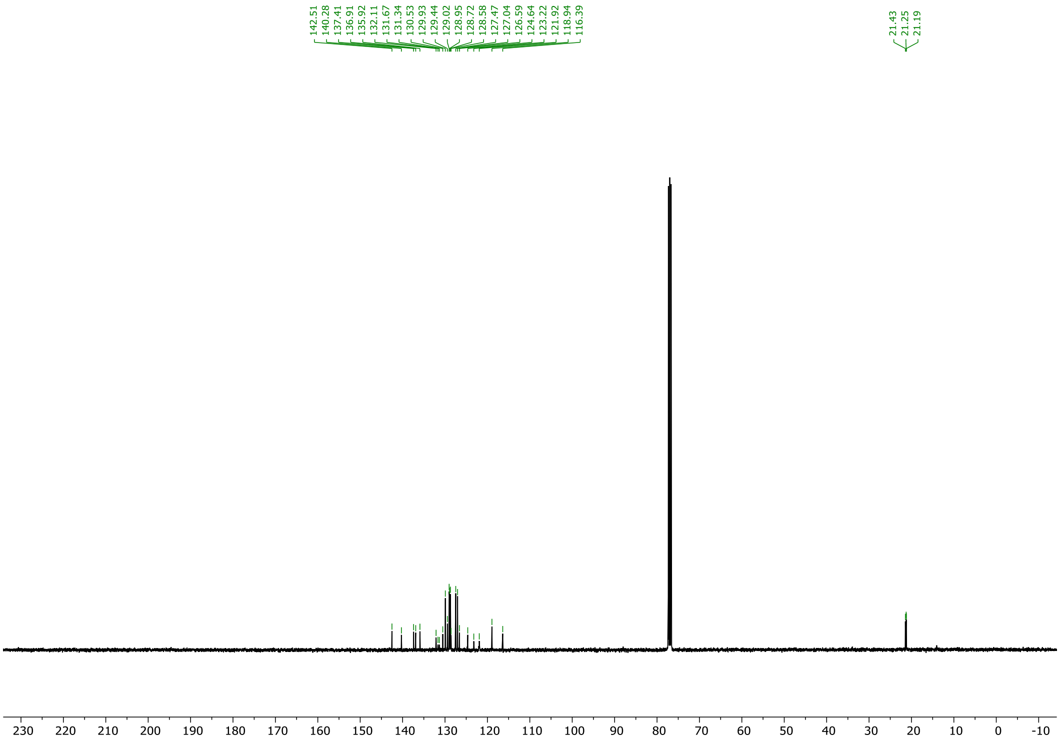

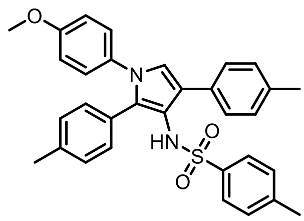

18d

^{1}H, CDCl$_3$, 400 MHz

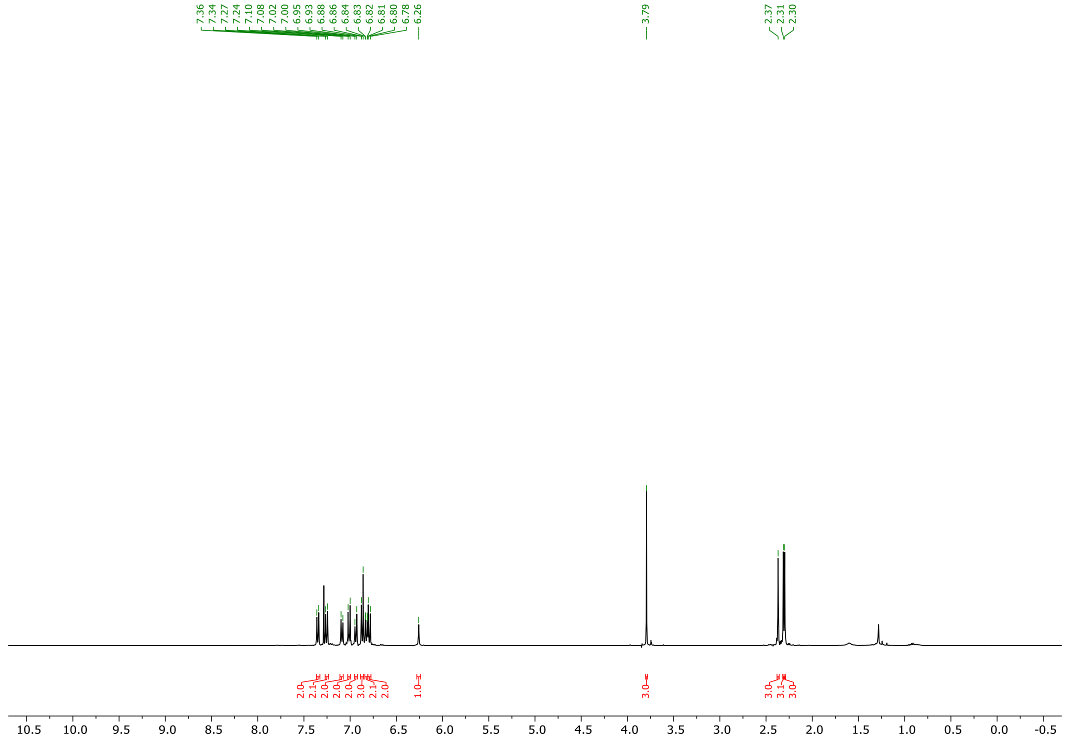

^{13}C{^{1}H}, CDCl$_3$, 101 MHz

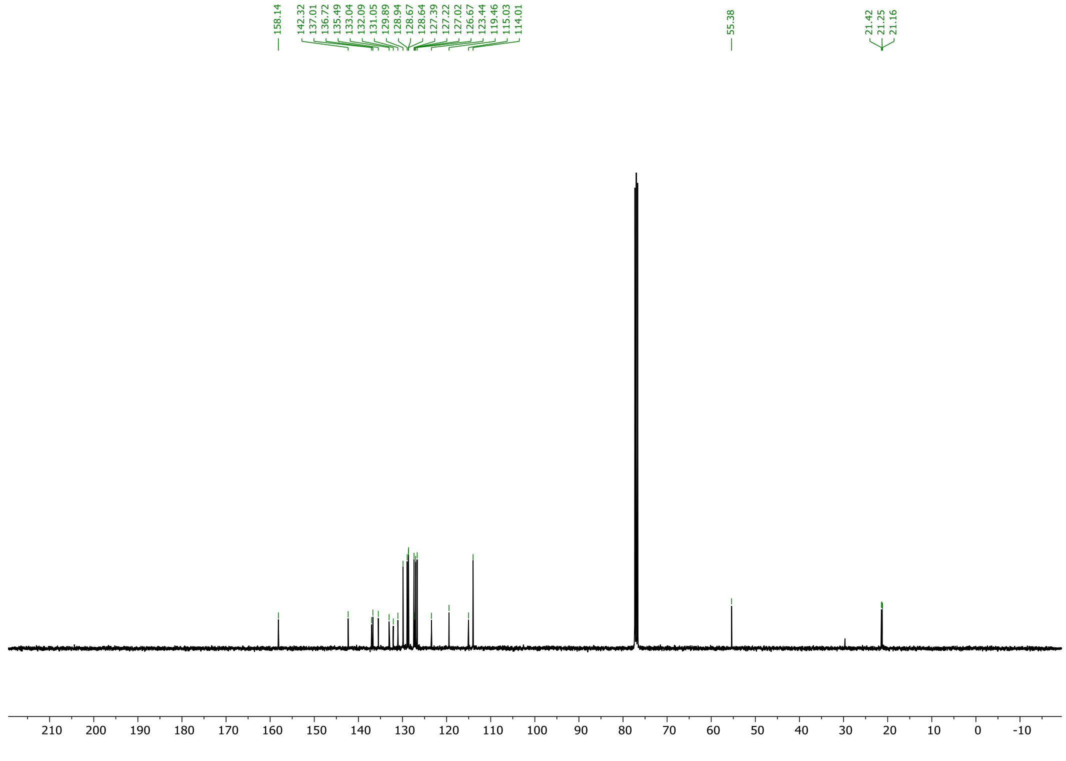

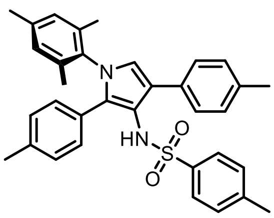

18e

^{1}H, CDCl$_3$, 400 MHz

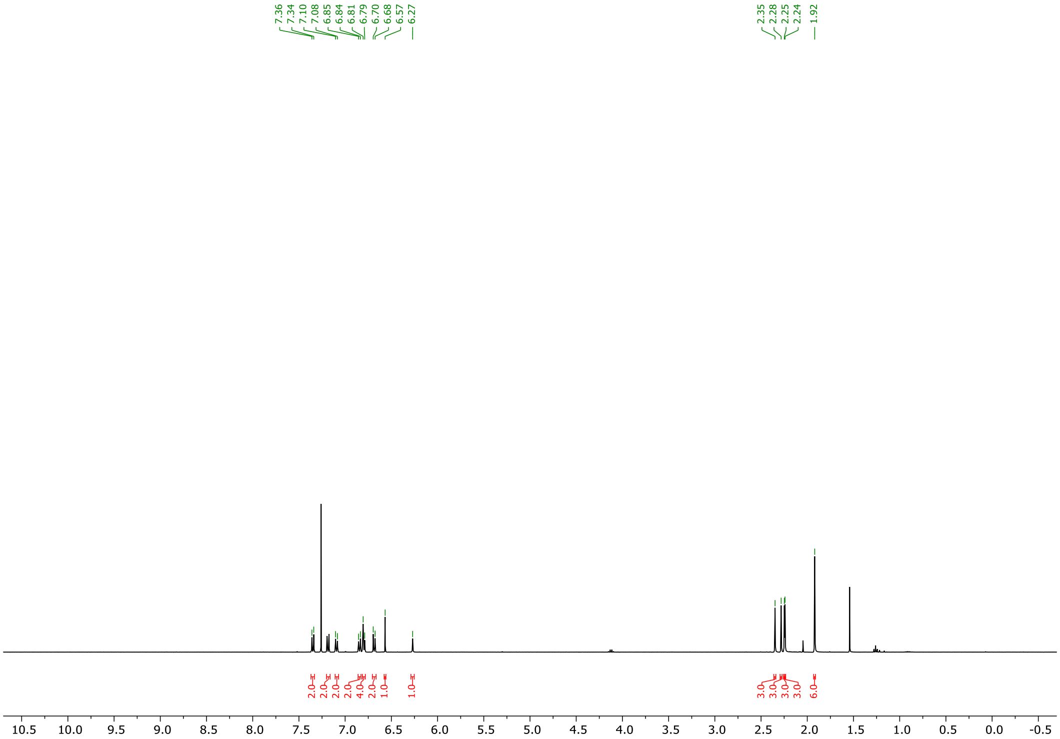

^{13}C{^{1}H}, CDCl$_3$, 101 MHz

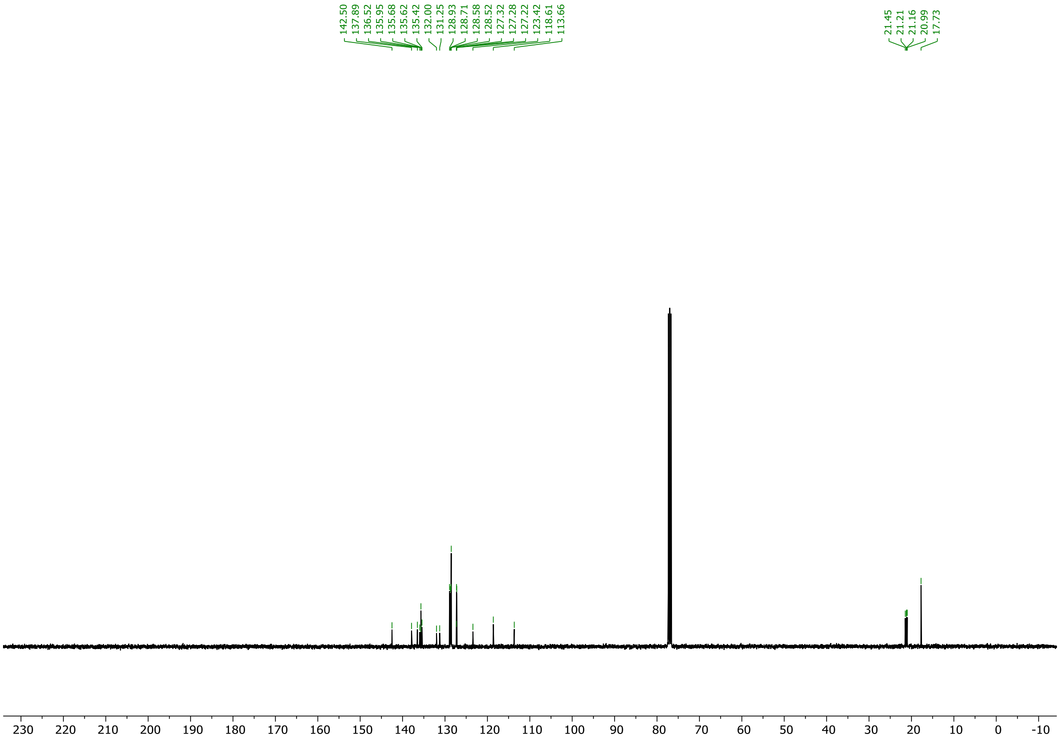

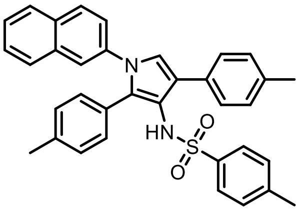

18f

¹H, CDCl₃, 400 MHz

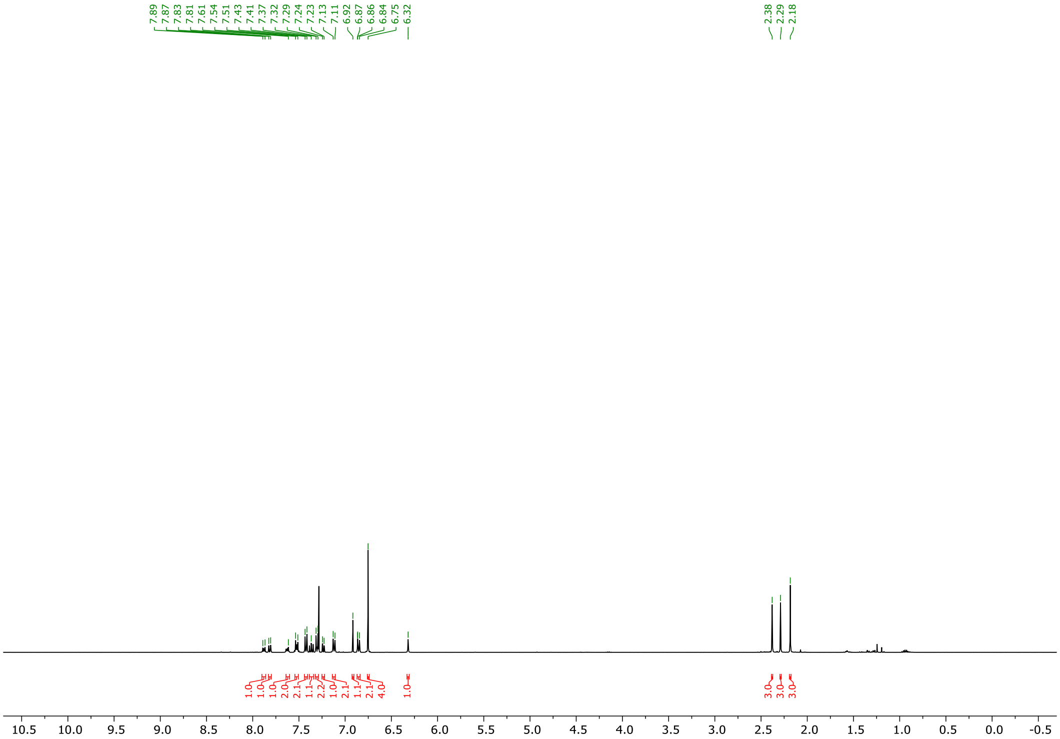

¹³C{¹H}, CDCl₃, 101 MHz

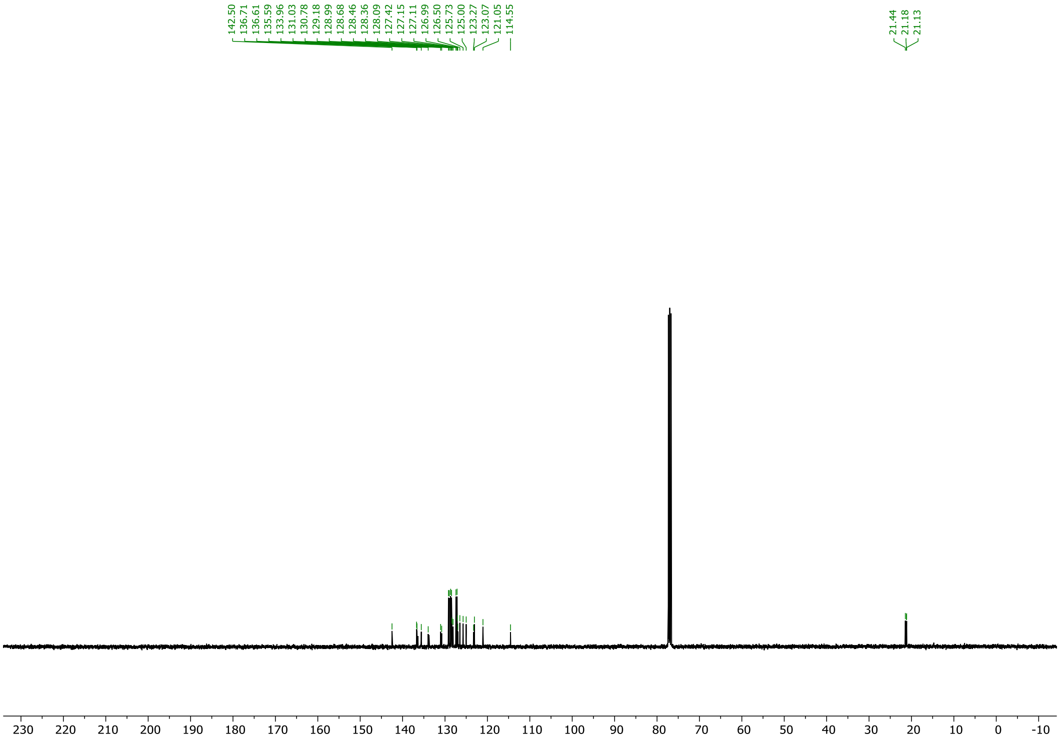

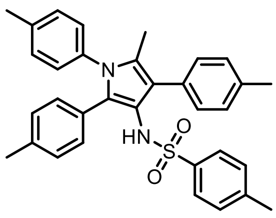

18g

^{1}H, $CDCl_3$, 400 MHz

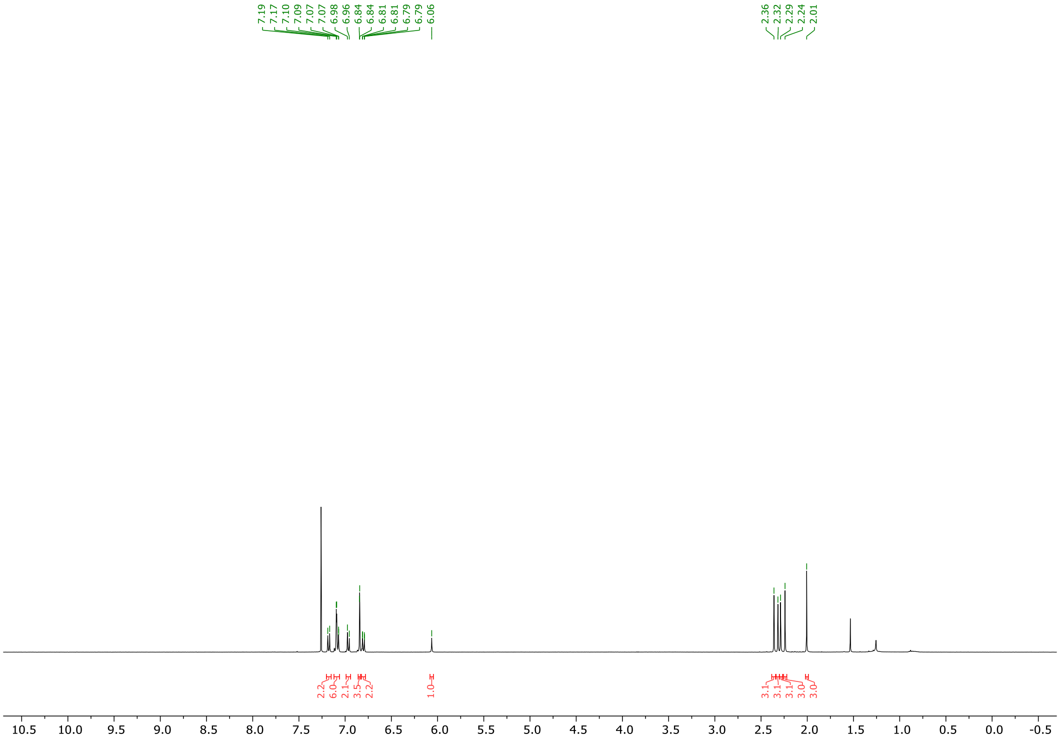

^{13}C{^{1}H}, $CDCl_3$, 101 MHz

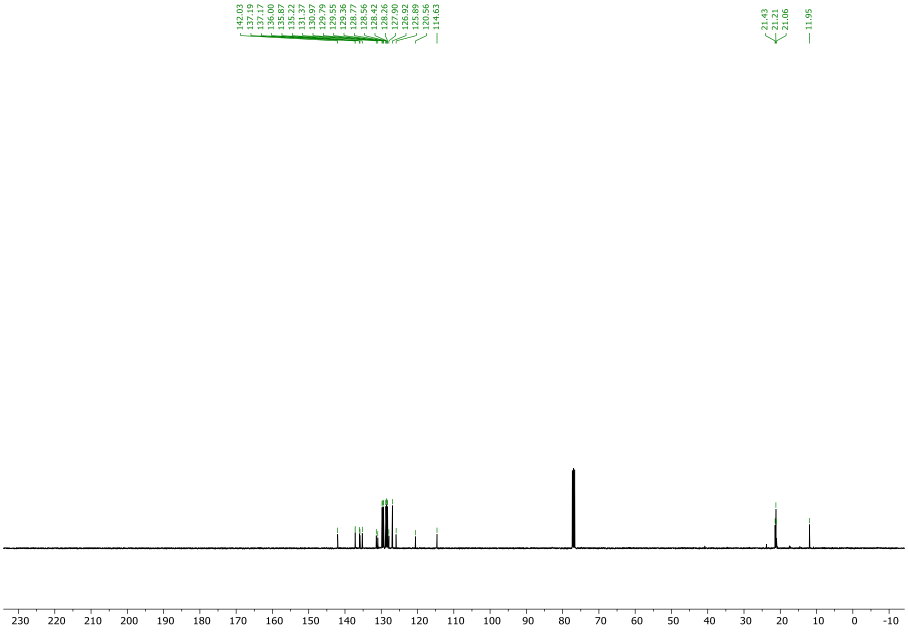

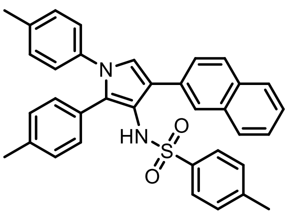

18h

^{1}H, CDCl$_3$, 400 MHz

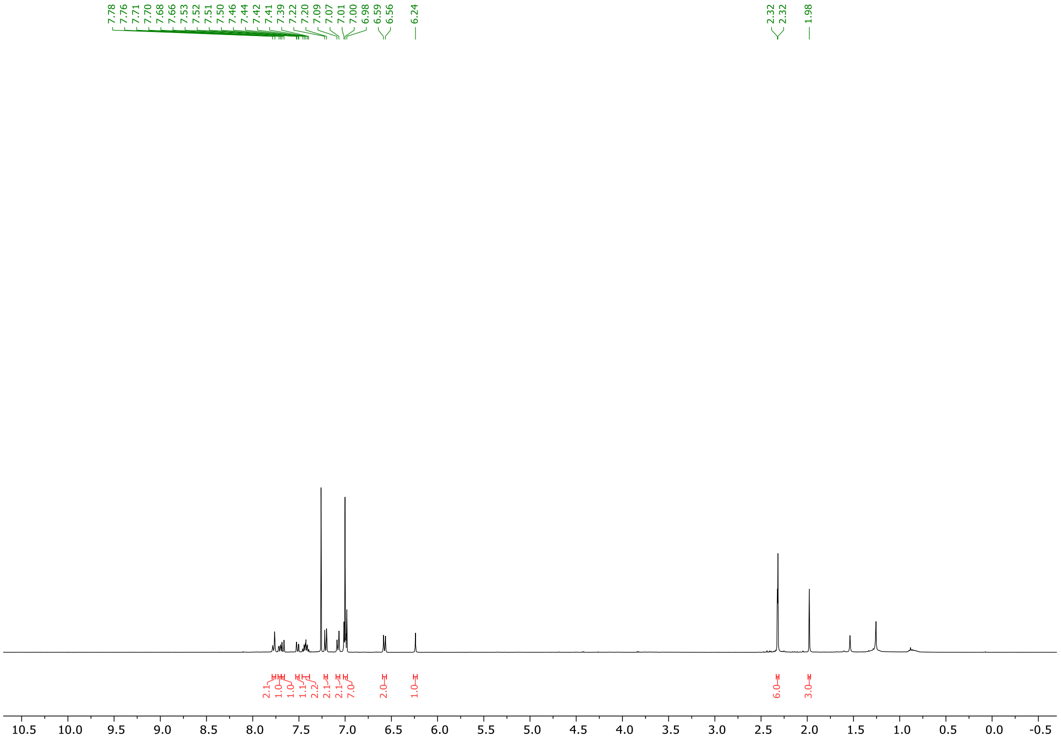

^{13}C{^{1}H}, CDCl$_3$, 101 MHz

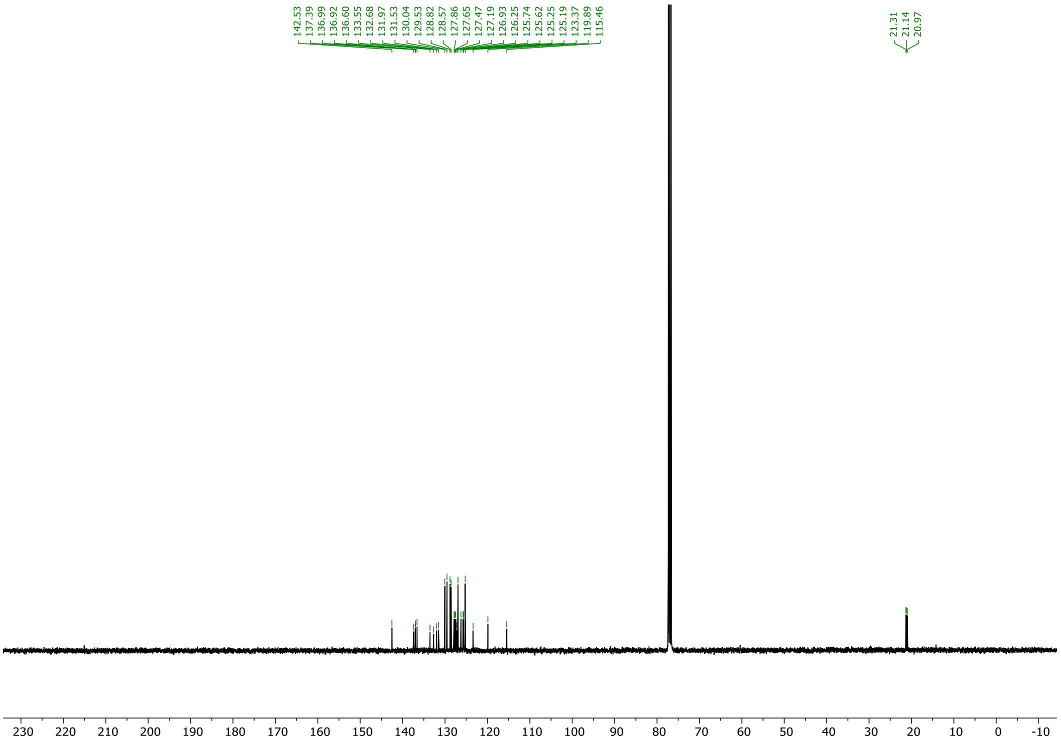

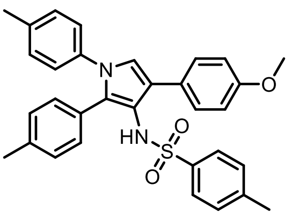

18i

^{1}H, $CDCl_3$, 400 MHz

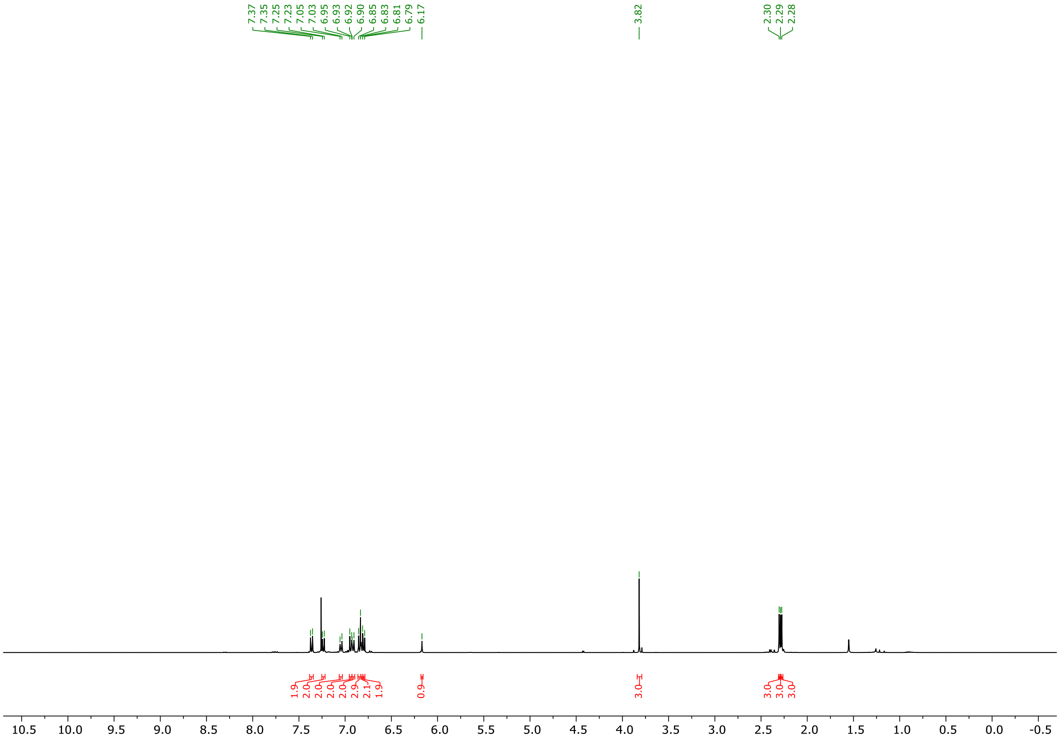

$^{13}C\{^{1}H\}$, $CDCl_3$, 101 MHz

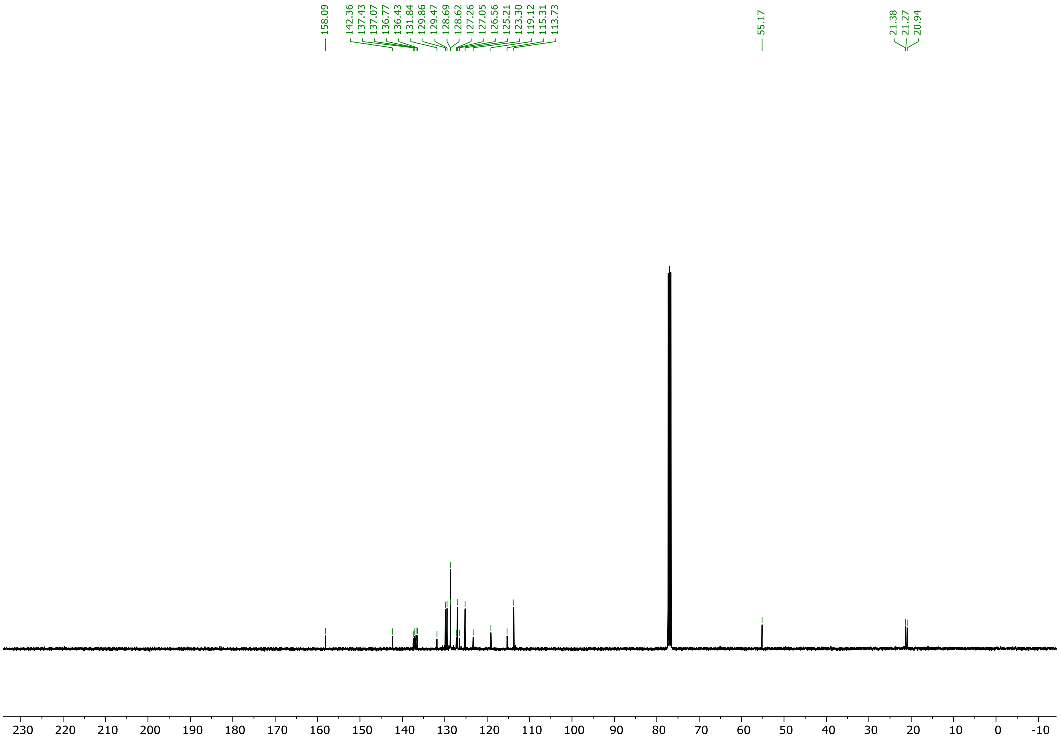

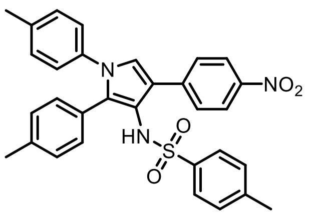

18j

^{1}H, $CDCl_3$, 400 MHz

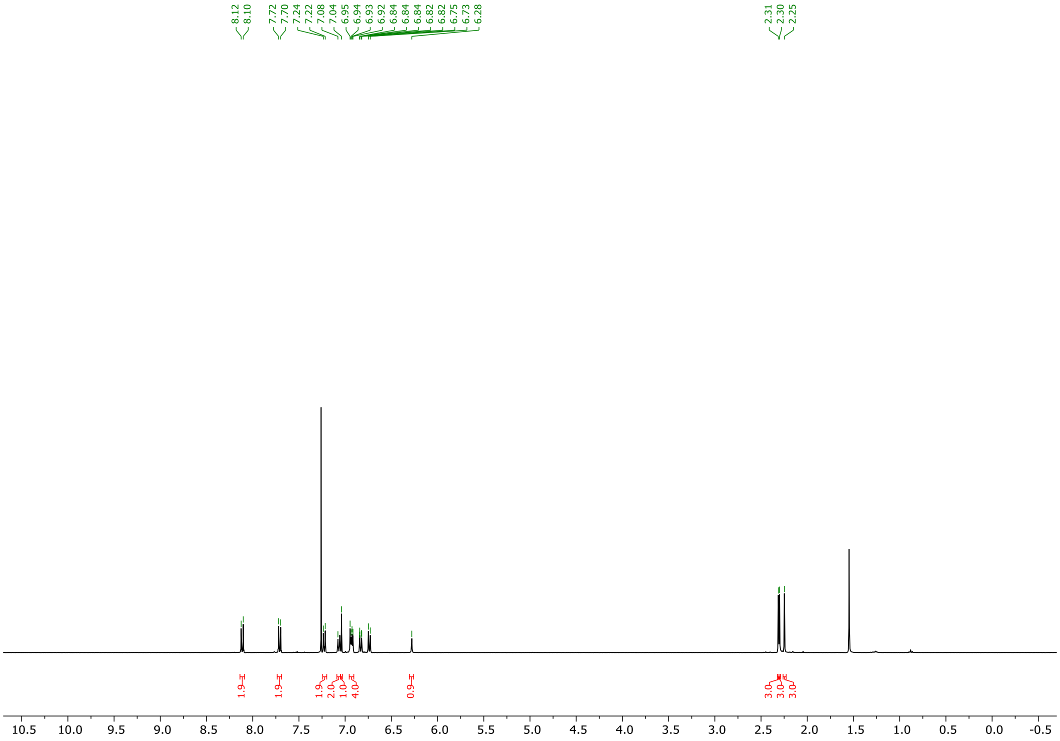

$^{13}C\{^{1}H\}$, $CDCl_3$, 101 MHz

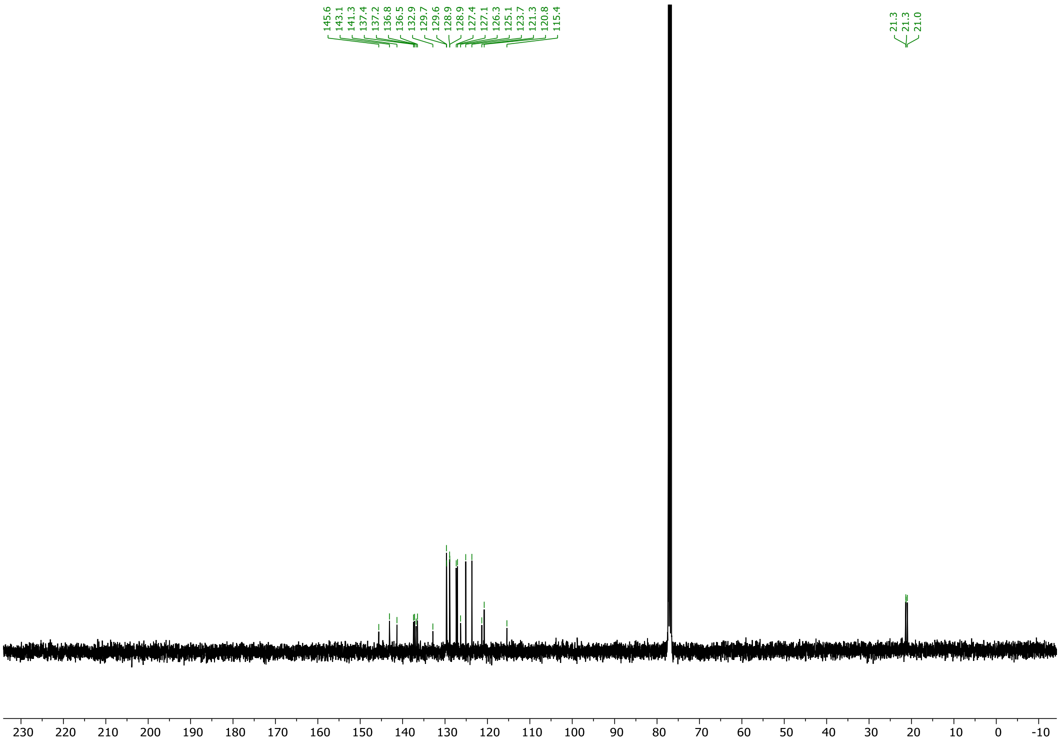

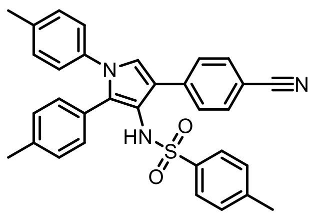

18k

^{1}H, $CDCl_3$, 400 MHz

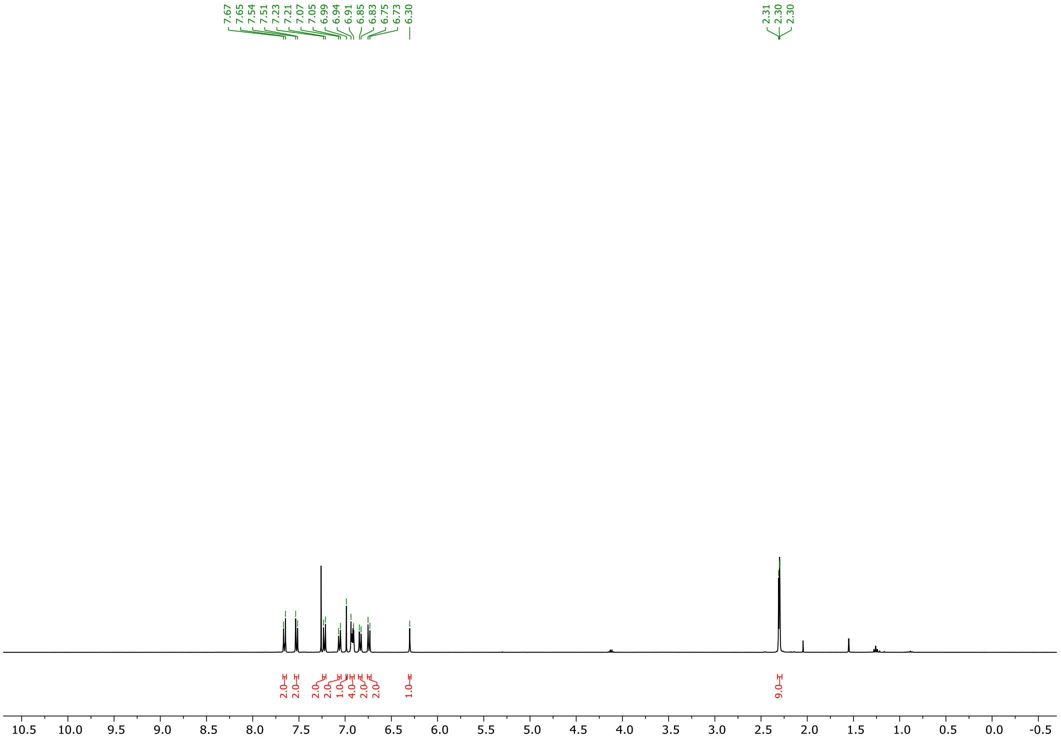

^{13}C{^{1}H}, $CDCl_3$, 101 MHz

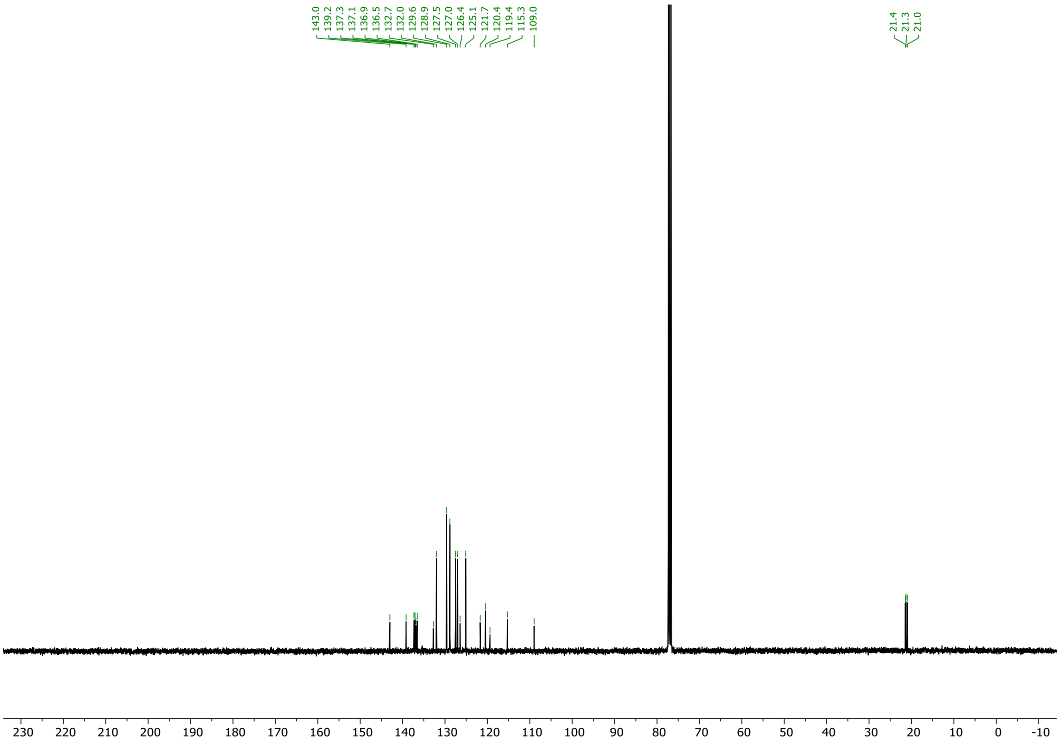

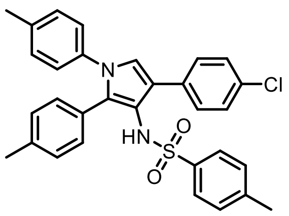

18l

^{1}H, CDCl$_3$, 400 MHz

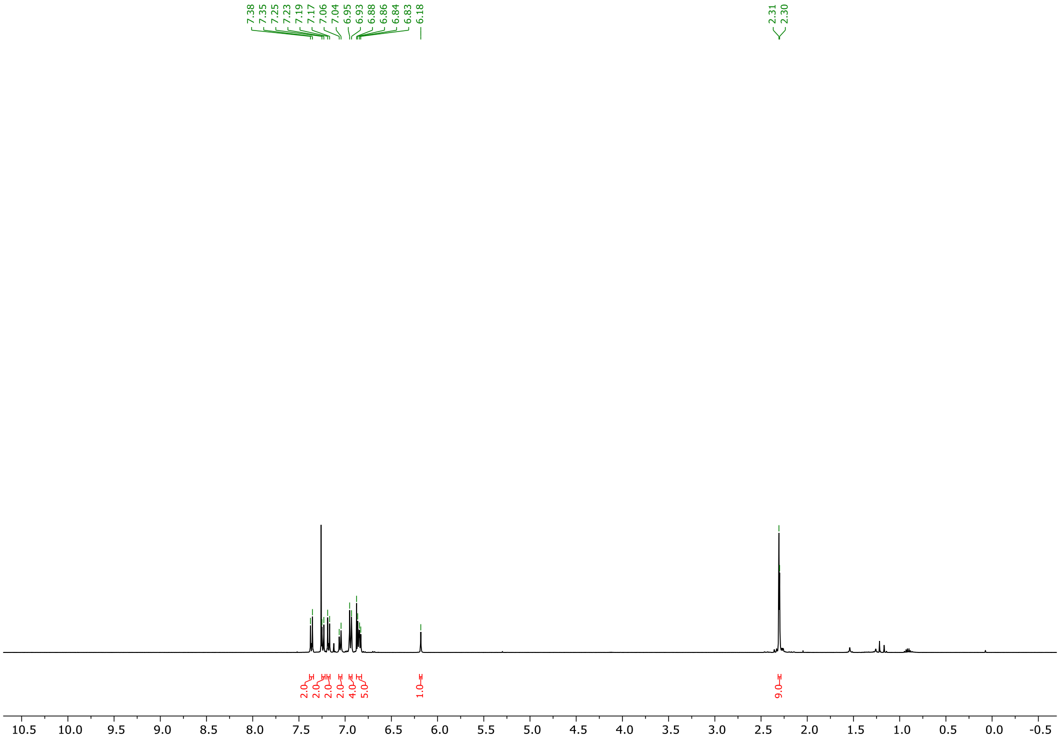

^{13}C{^{1}H}, CDCl$_3$, 101 MHz

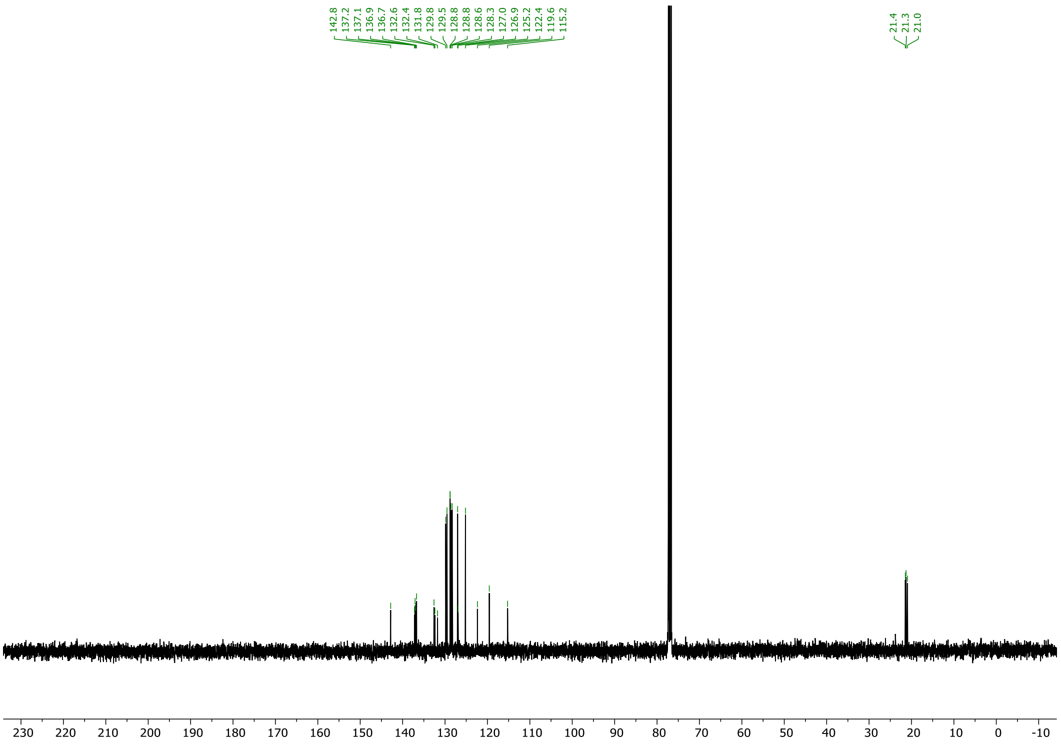

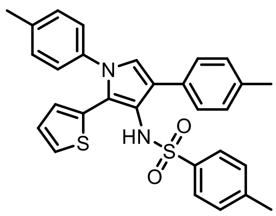

18m

^{1}H, CDCl$_3$, 400 MHz

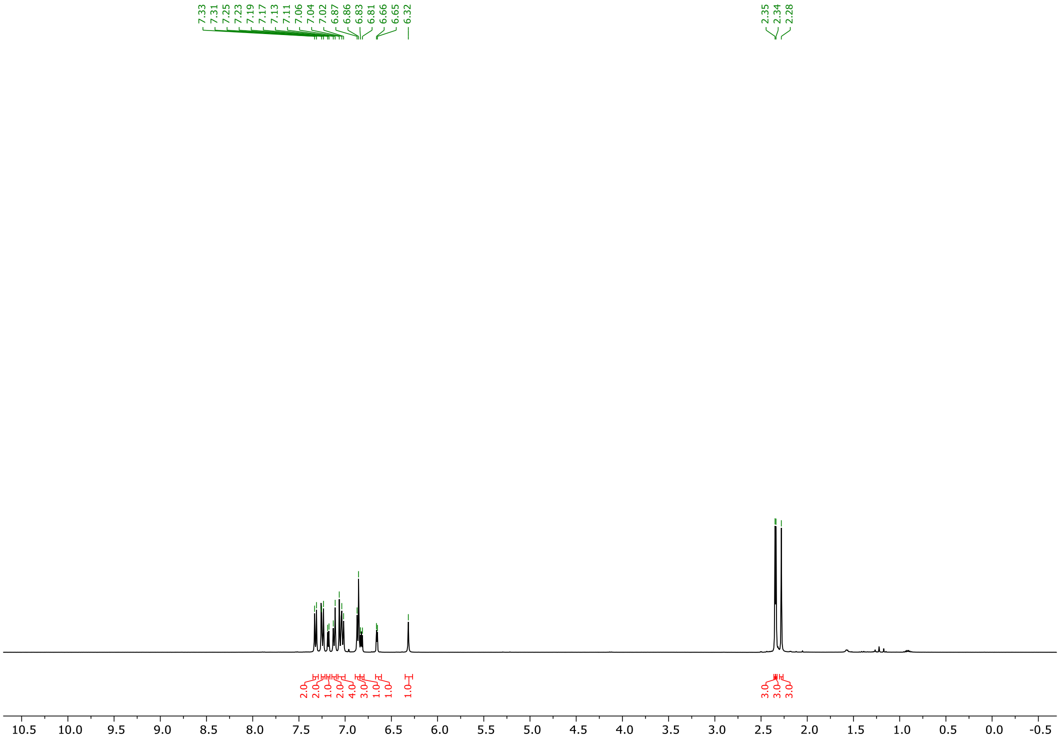

^{13}C{^{1}H}, CDCl$_3$, 101 MHz

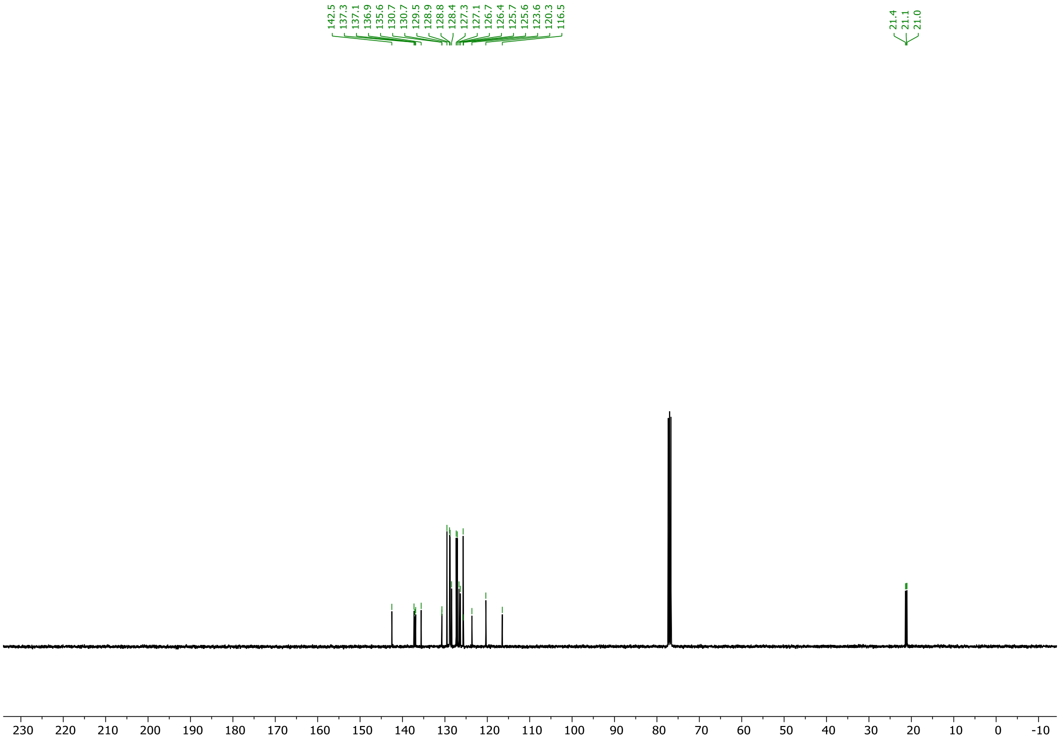

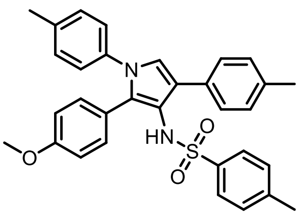

18n

^{1}H, CDCl$_3$, 400 MHz

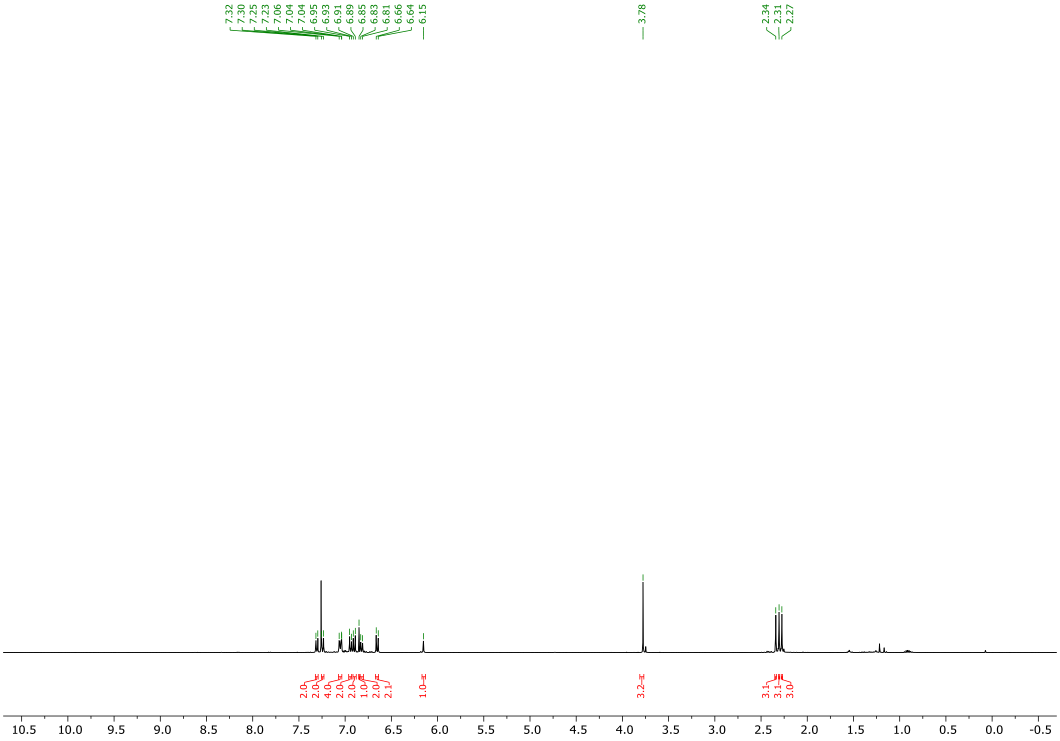

^{13}C{^{1}H}, CDCl$_3$, 101 MHz

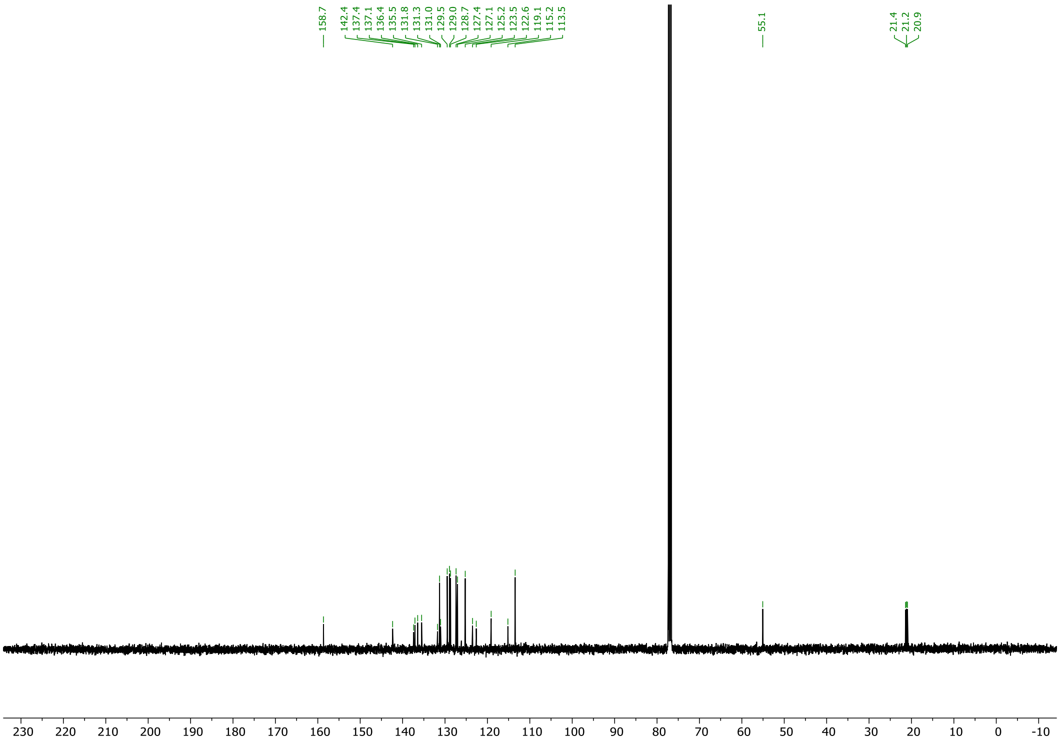

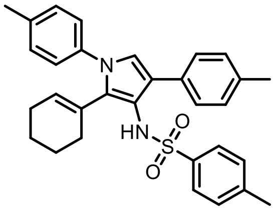

18o

^{1}H, CDCl$_3$, 400 MHz

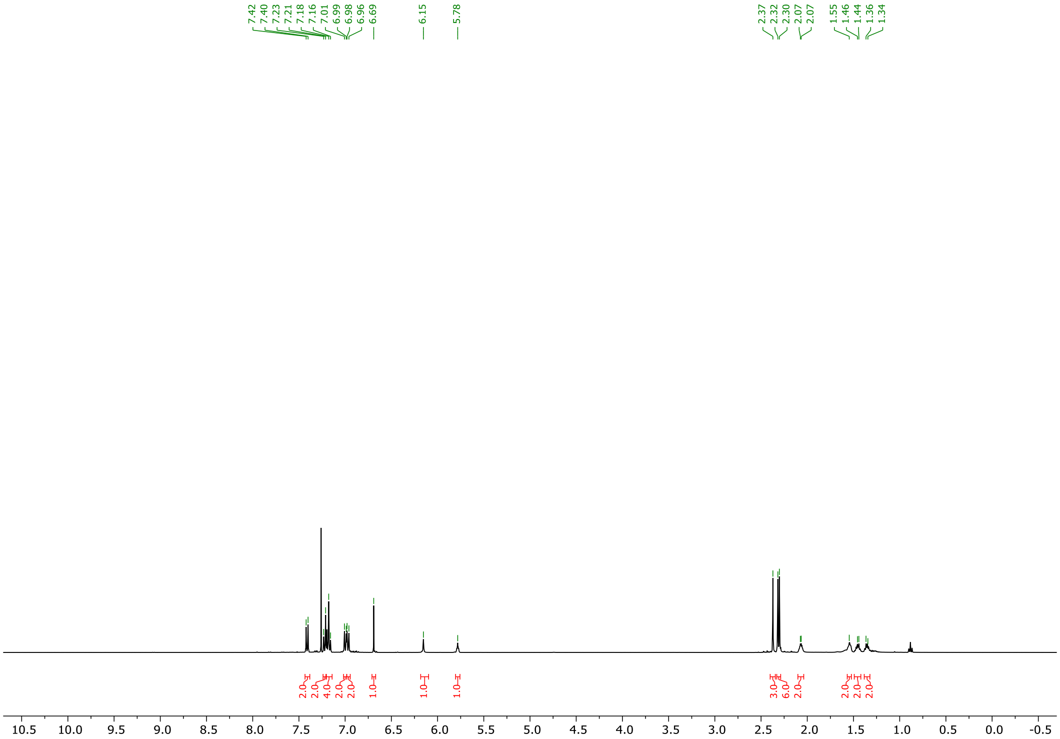

^{13}C{^{1}H}, CDCl$_3$, 101 MHz

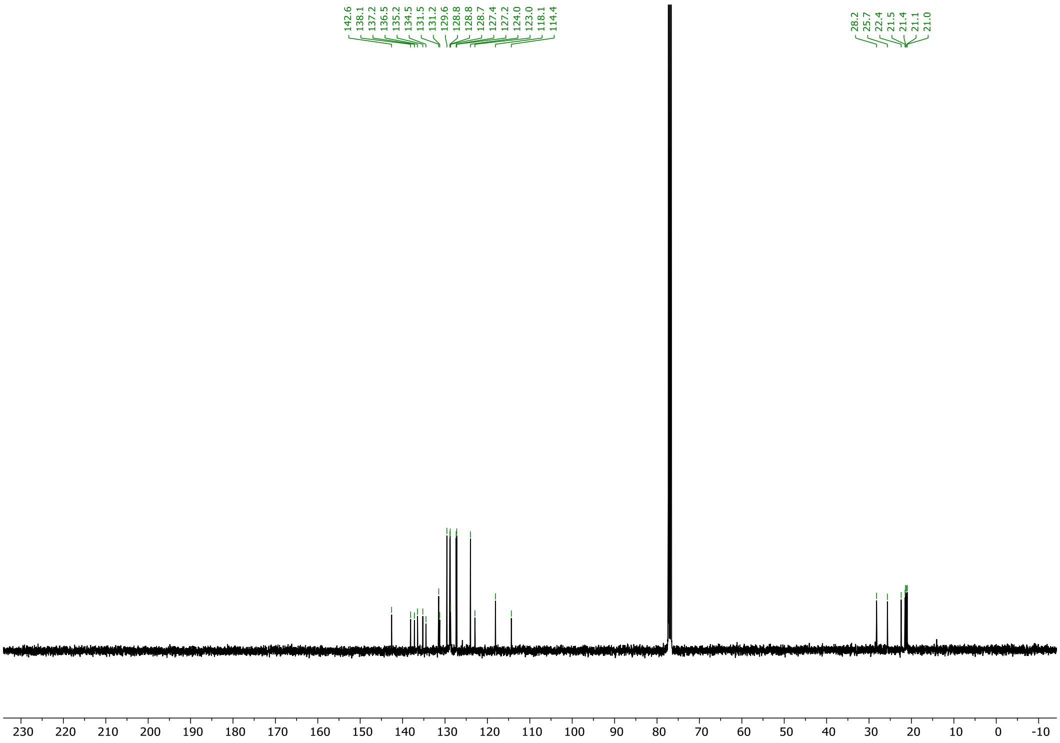

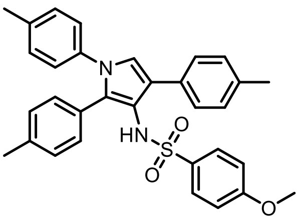

18p

^{1}H, CDCl$_3$, 400 MHz

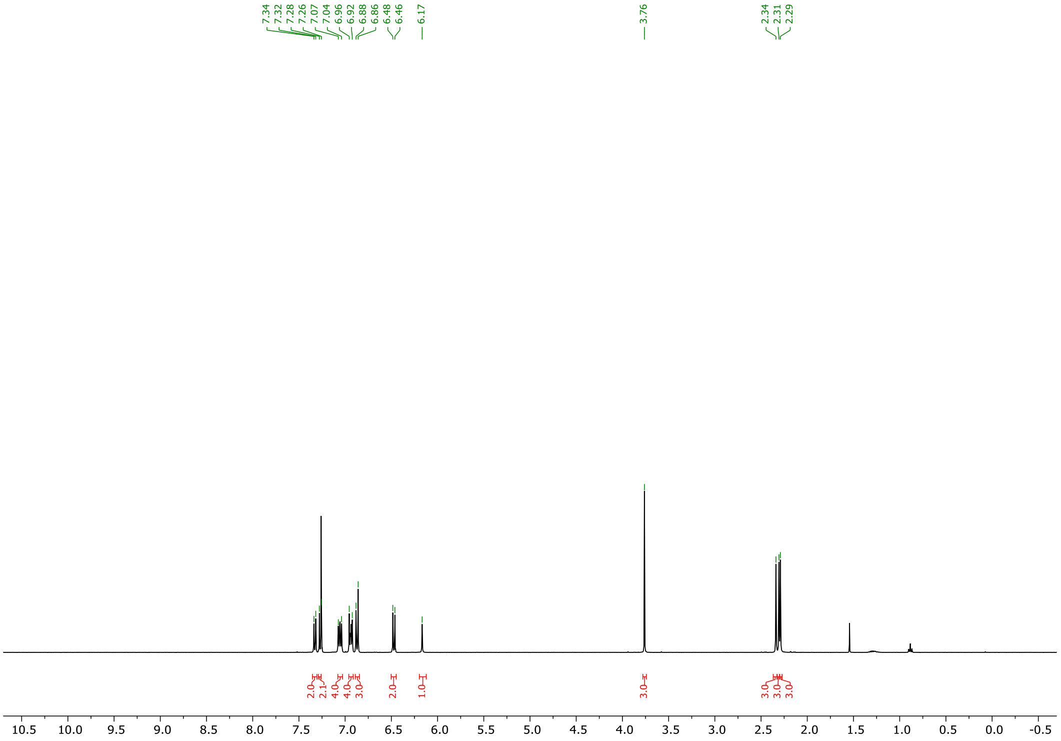

^{13}C{^{1}H}, CDCl$_3$, 101 MHz

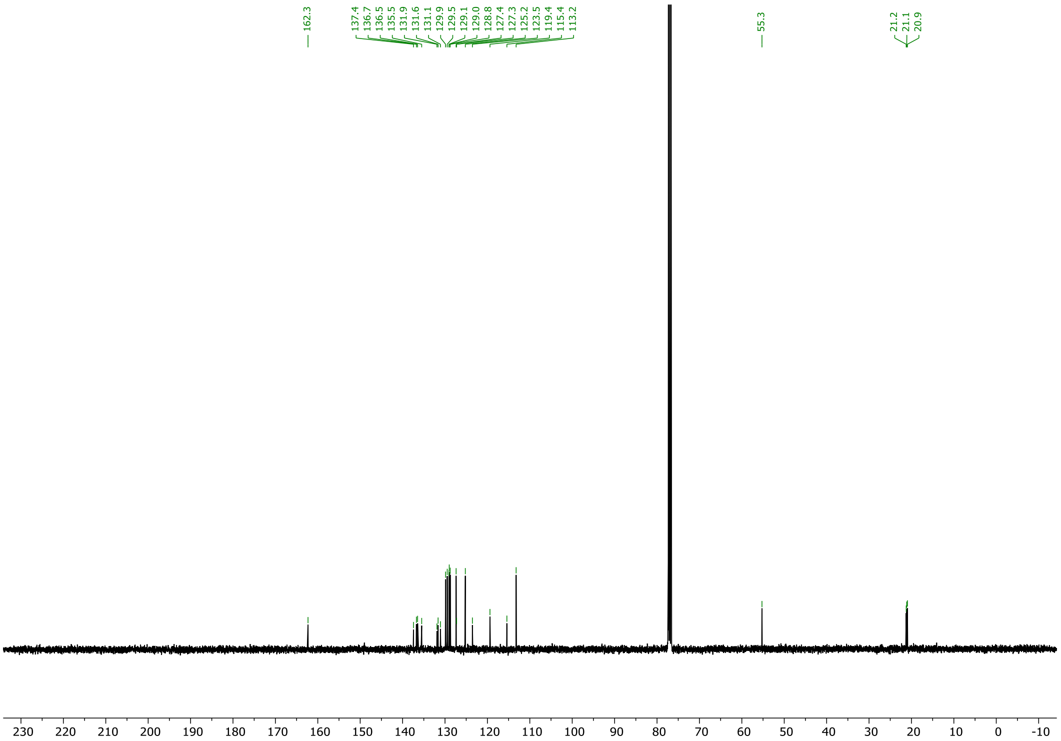

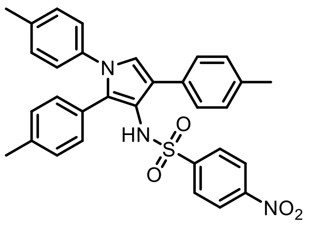

18q

1H, CDCl3, 400 MHz

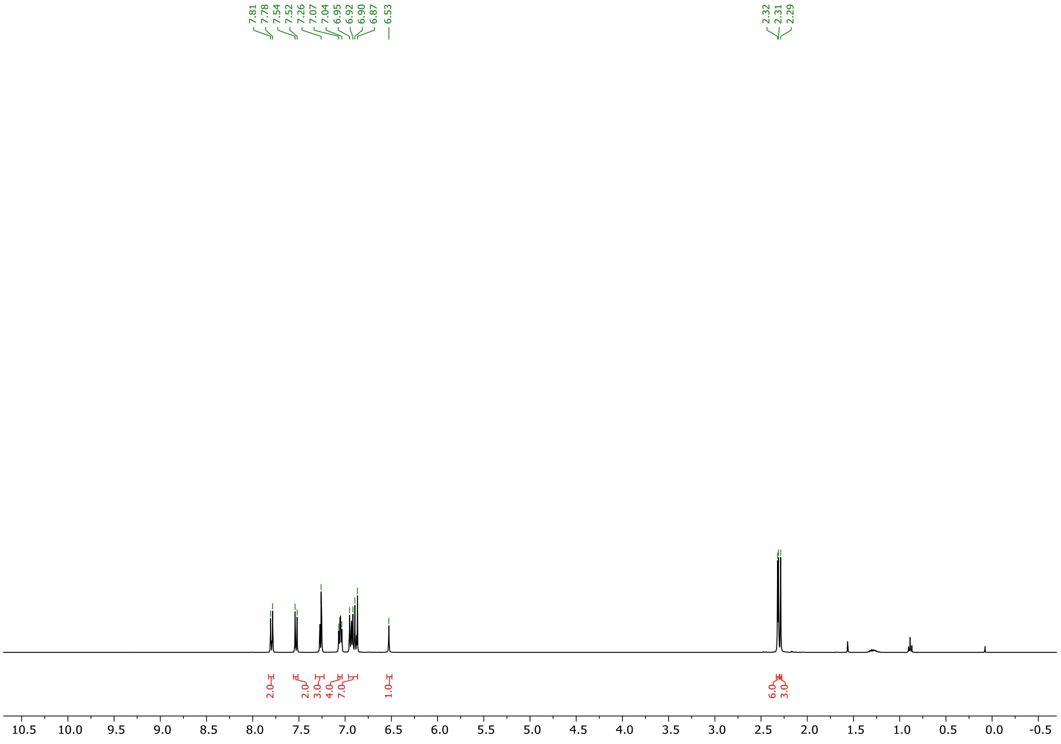

13C{1H}, CDCl3, 101 MHz

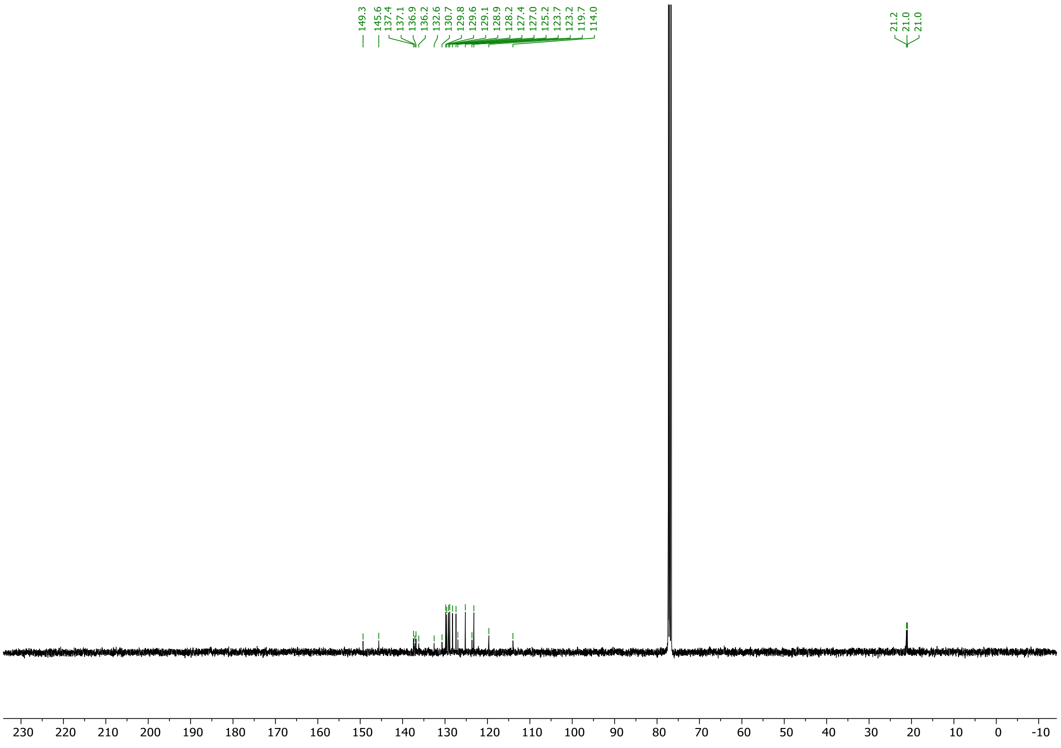

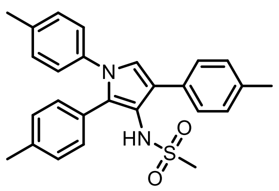

18r

^{1}H, CDCl$_3$, 400 MHz

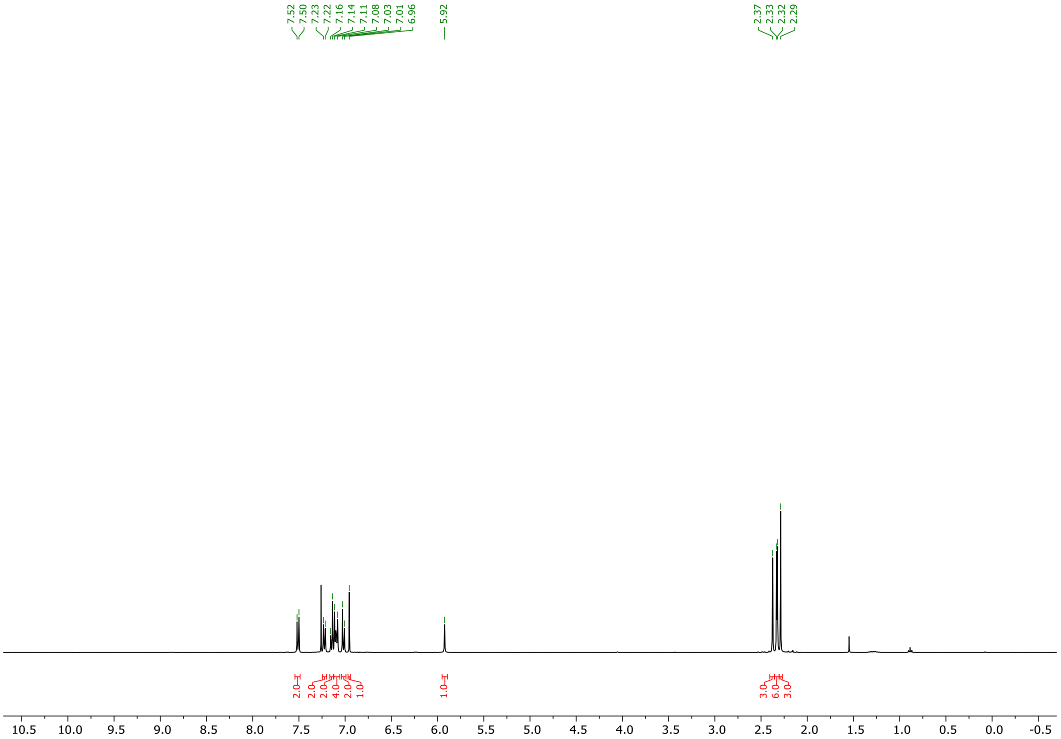

^{13}C{^{1}H}, CDCl$_3$, 101 MHz

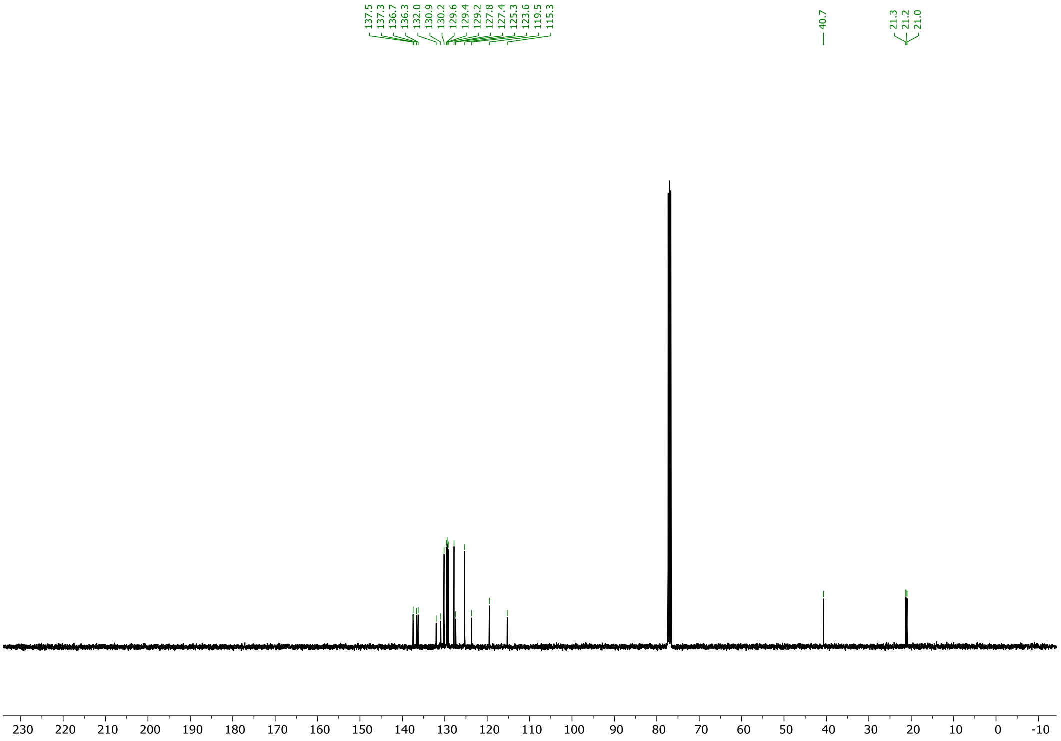

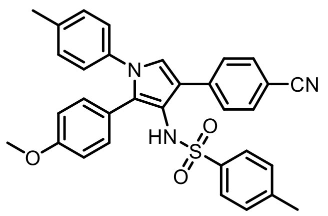

18s

^{1}H, CDCl₃, 400 MHz

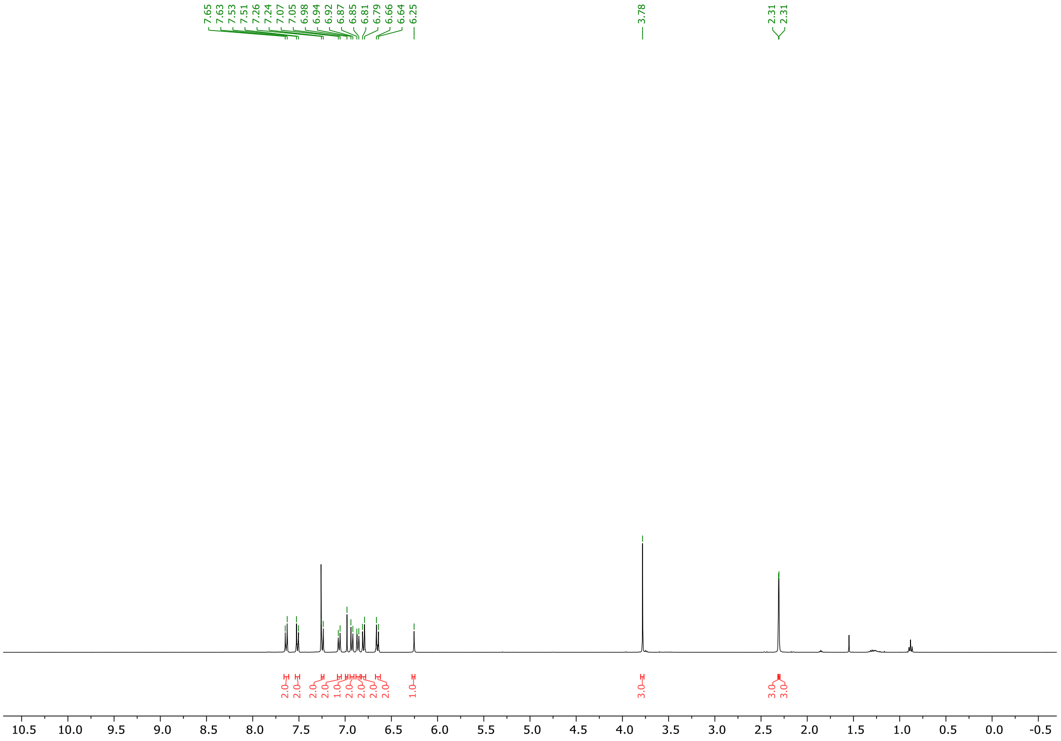

^{13}C{^{1}H}, CDCl₃, 101 MHz

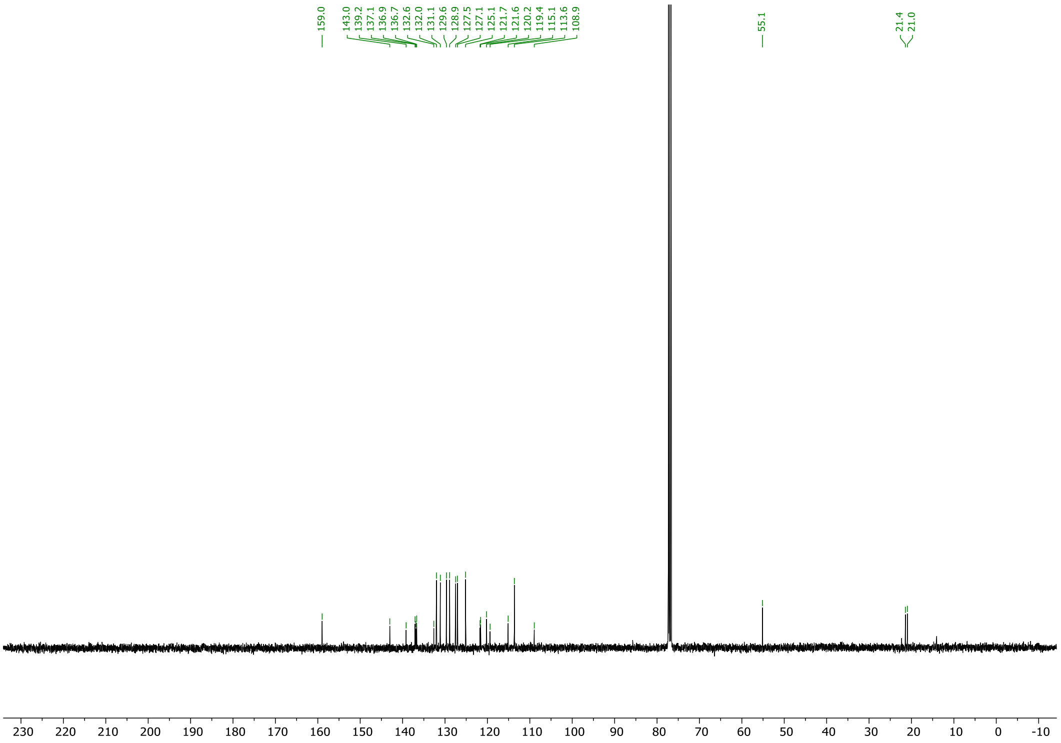

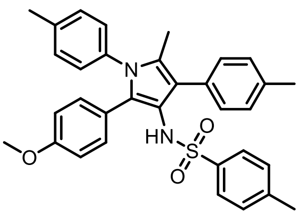

18t

^{1}H, CDCl$_3$, 400 MHz

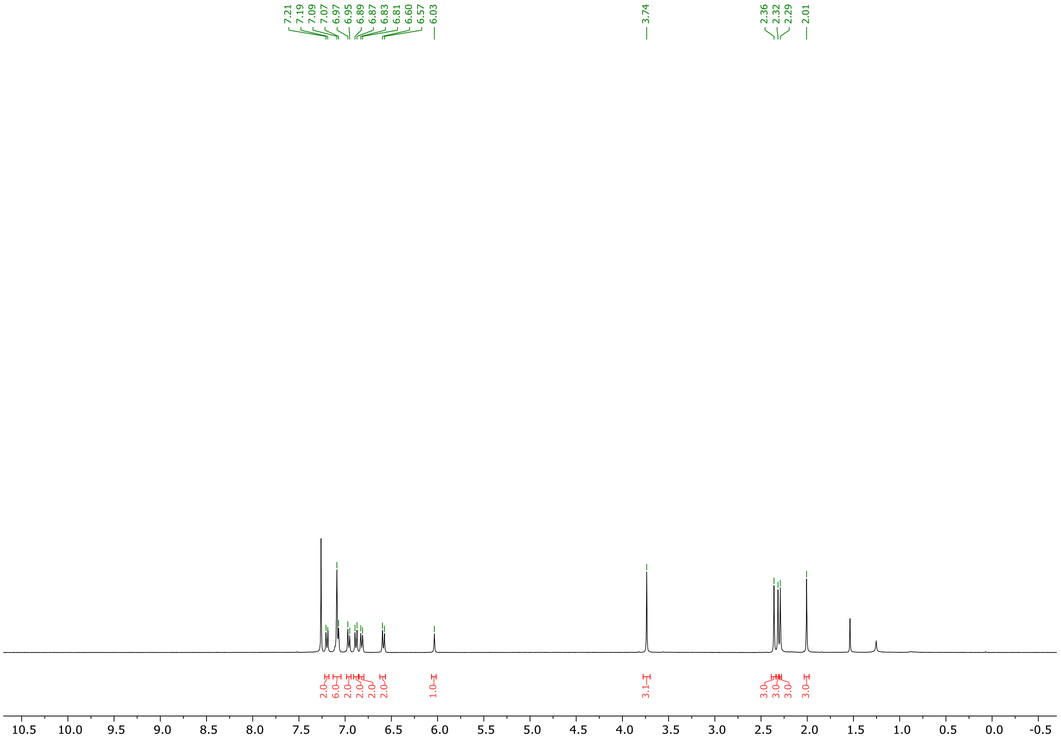

^{13}C{^{1}H}, CDCl$_3$, 101 MHz

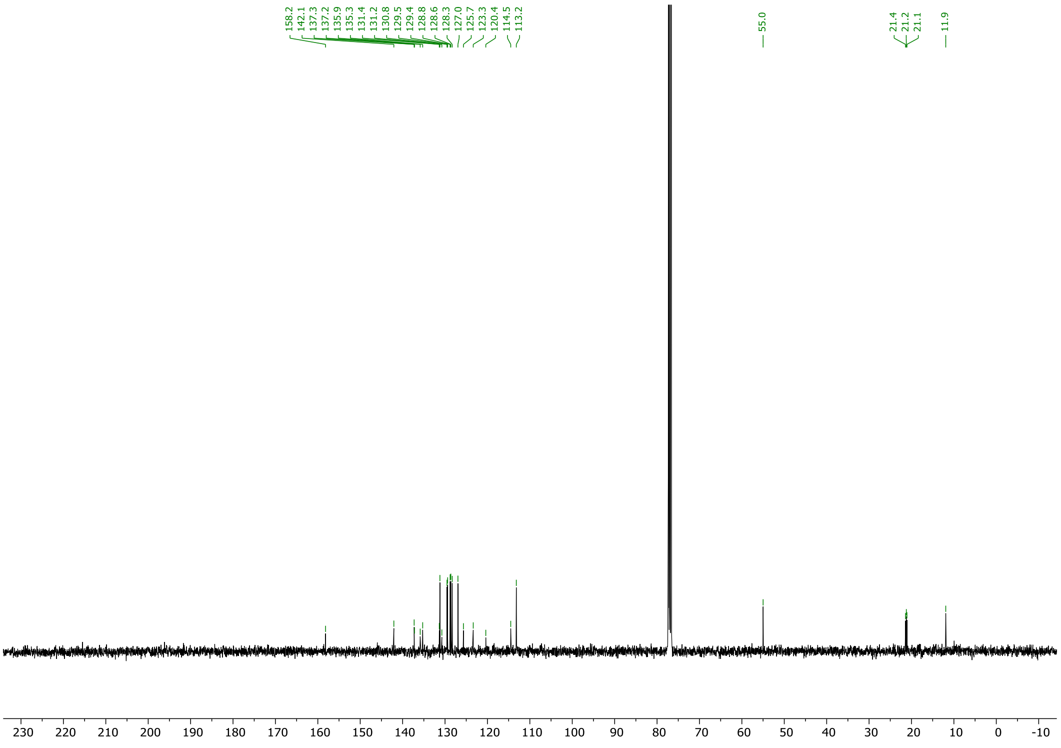

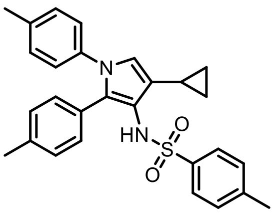

18u

^{1}H, $CDCl_3$, 400 MHz

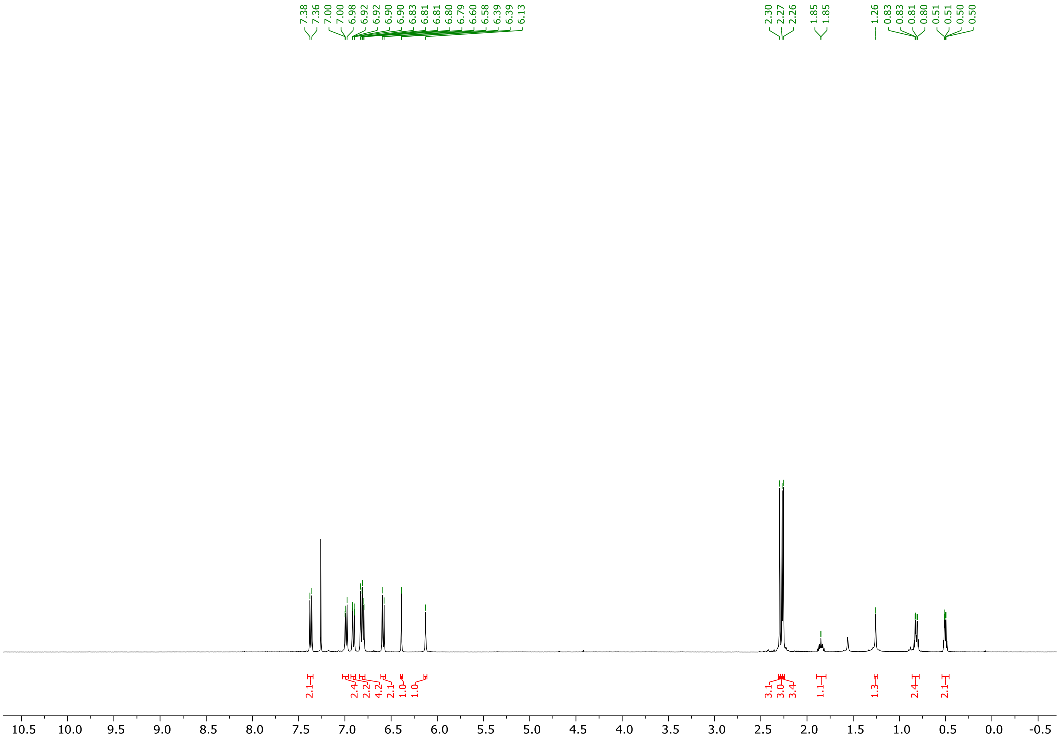

$^{13}C\{^{1}H\}$, $CDCl_3$, 101 MHz

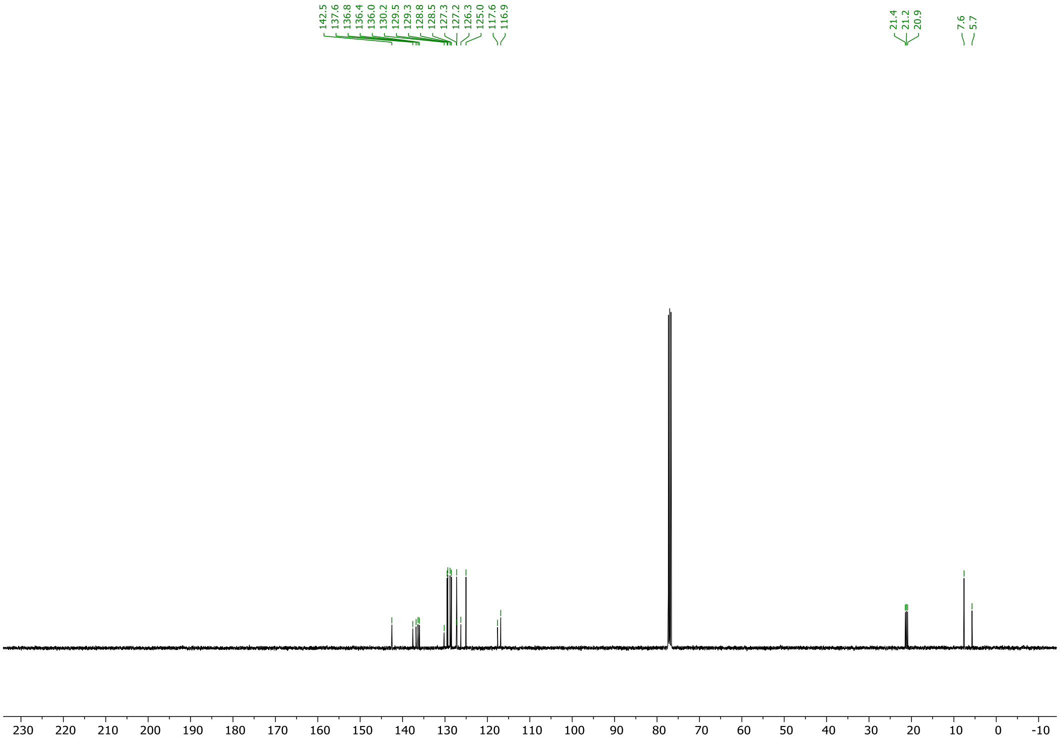

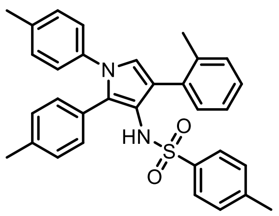

18v

^{1}H, CDCl$_3$, 400 MHz

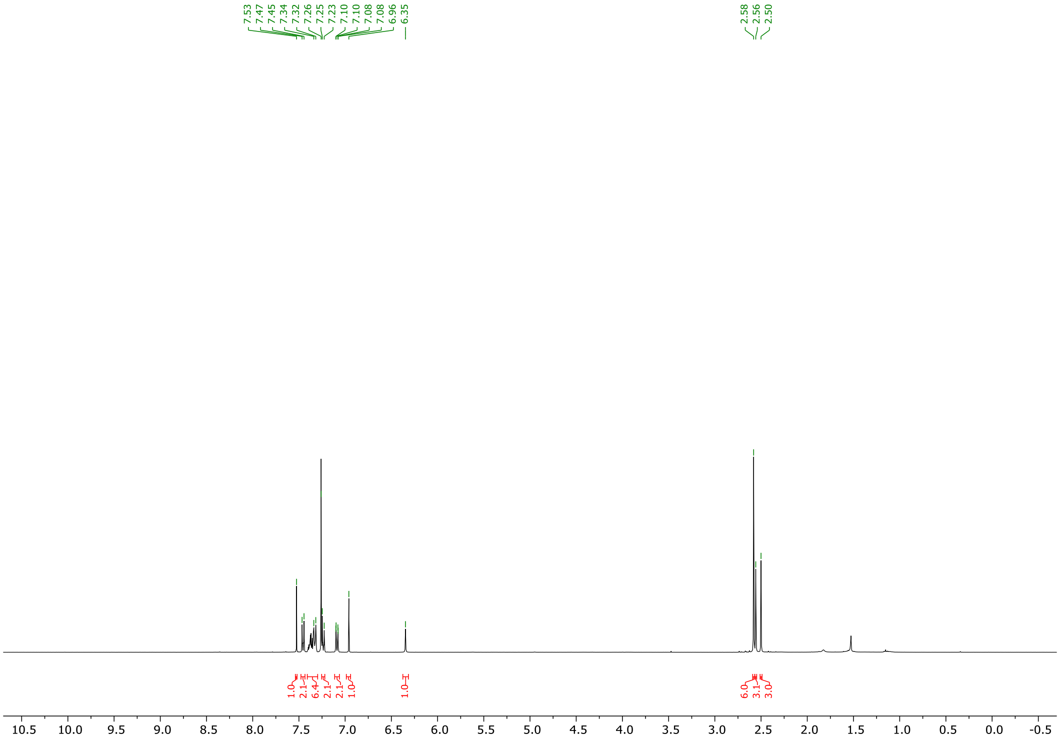

^{13}C{^{1}H}, CDCl$_3$, 101 MHz

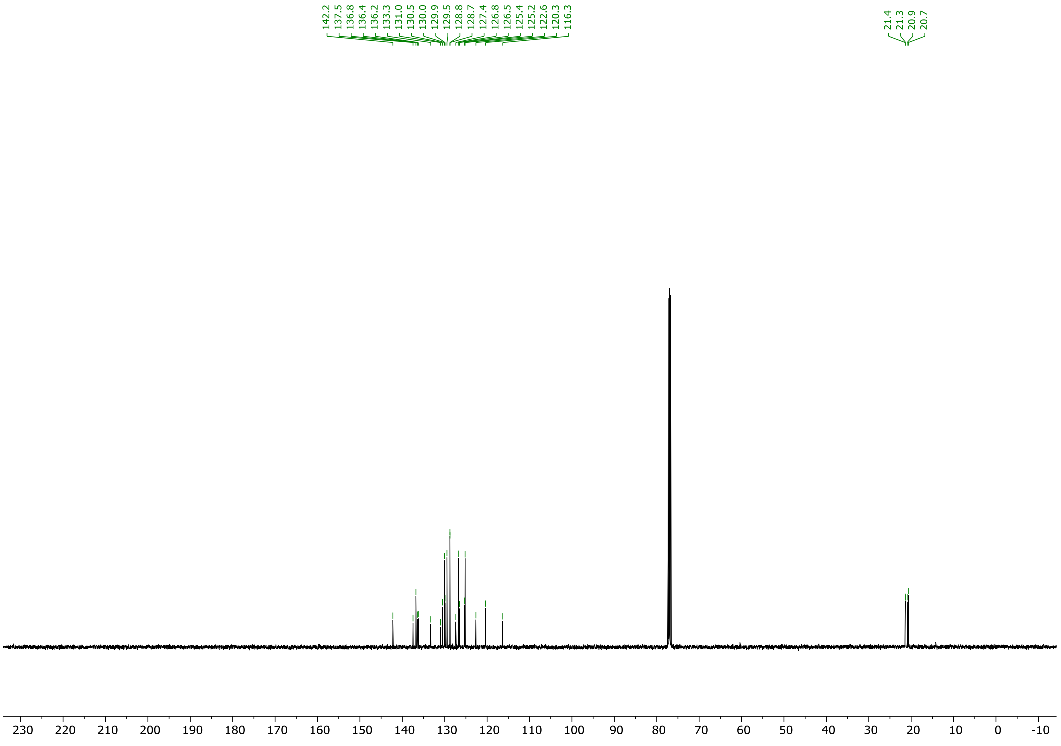

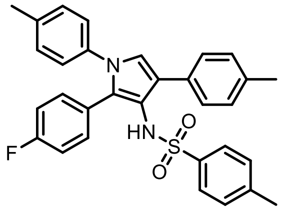

18w

^{1}H, CDCl$_3$, 400 MHz

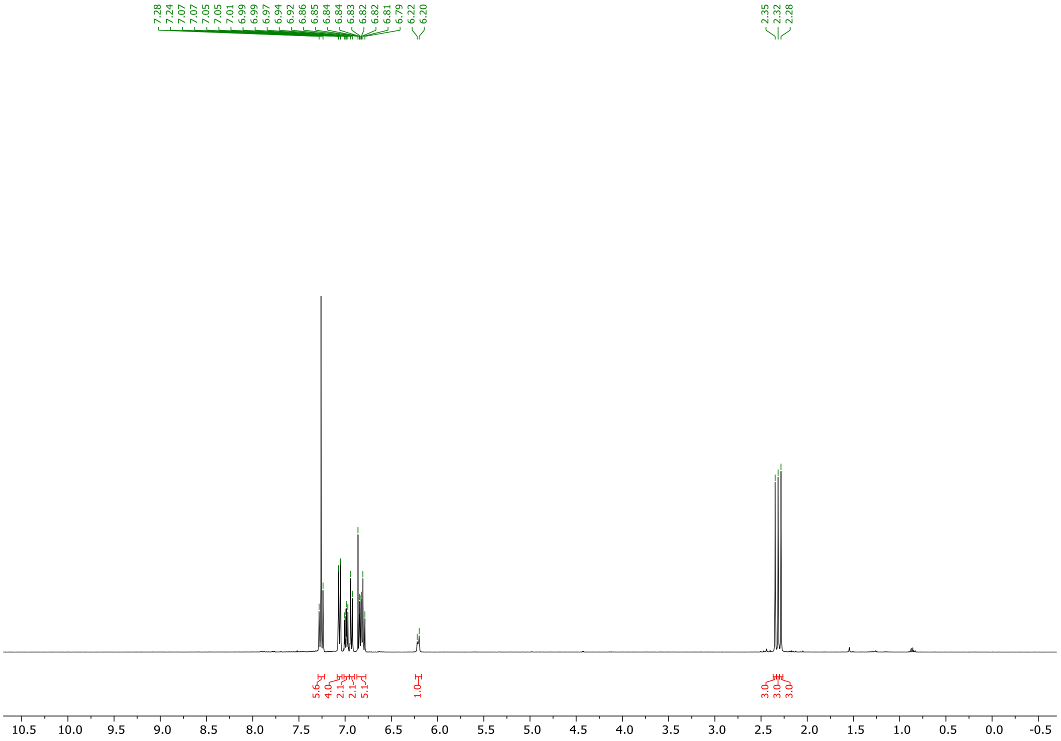

^{13}C{^{1}H}, CDCl$_3$, 101 MHz

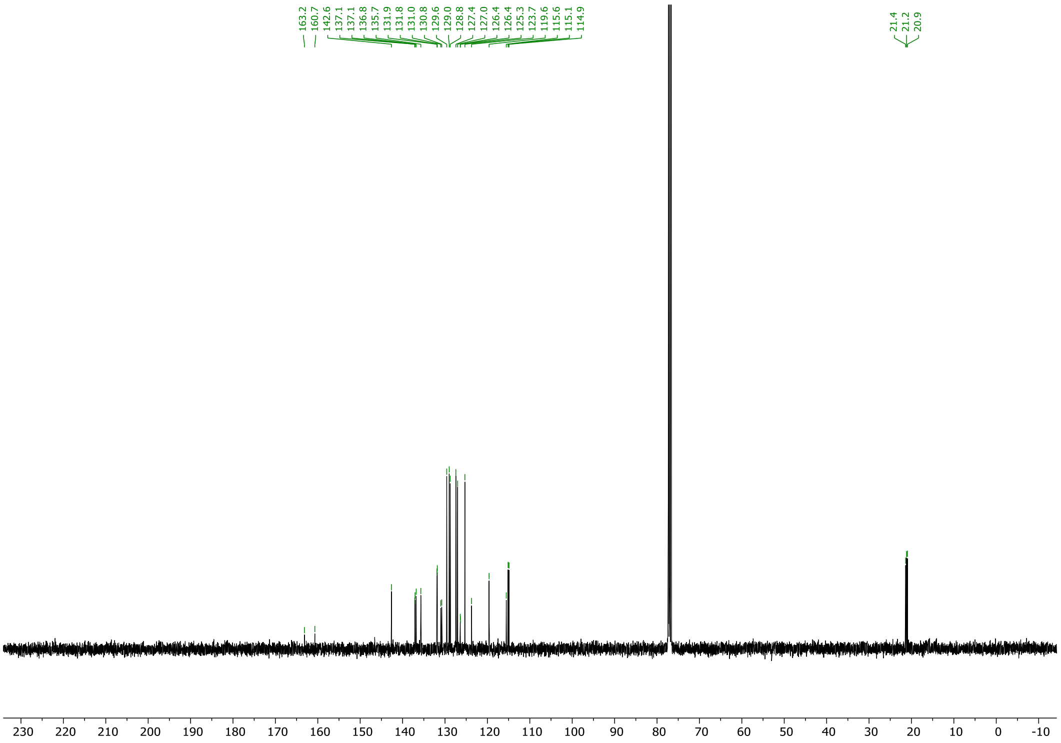

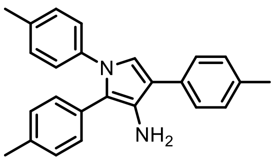

23a

^{1}H, $CDCl_3$, 400 MHz

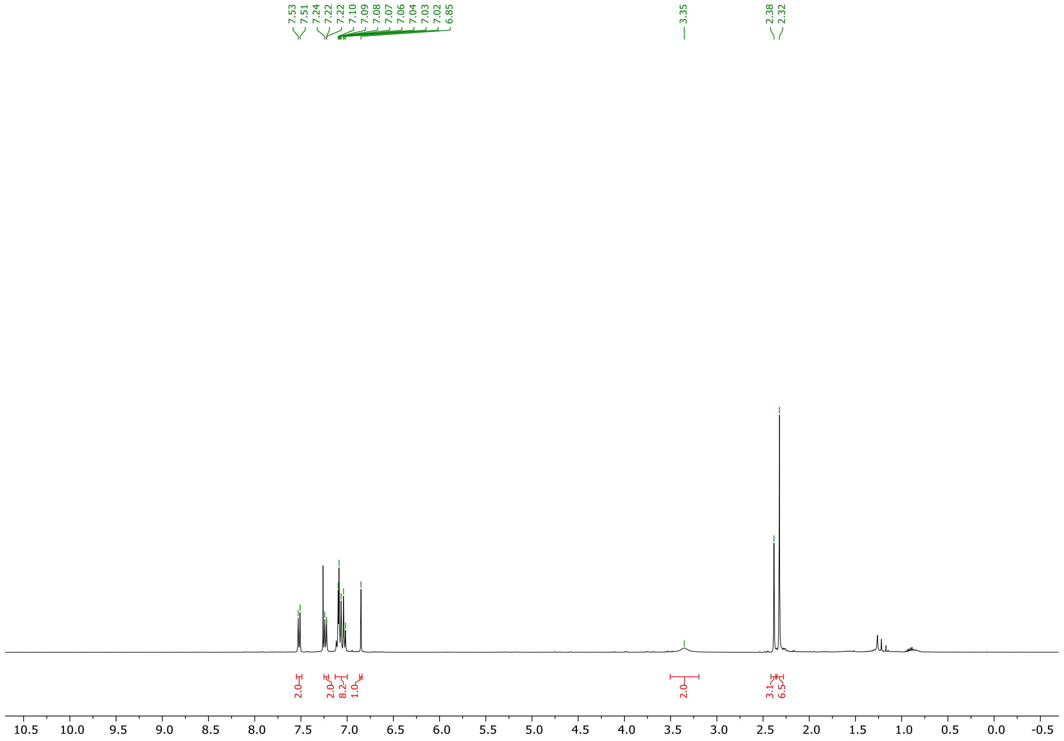

$^{13}C\{^{1}H\}$, $CDCl_3$, 101 MHz

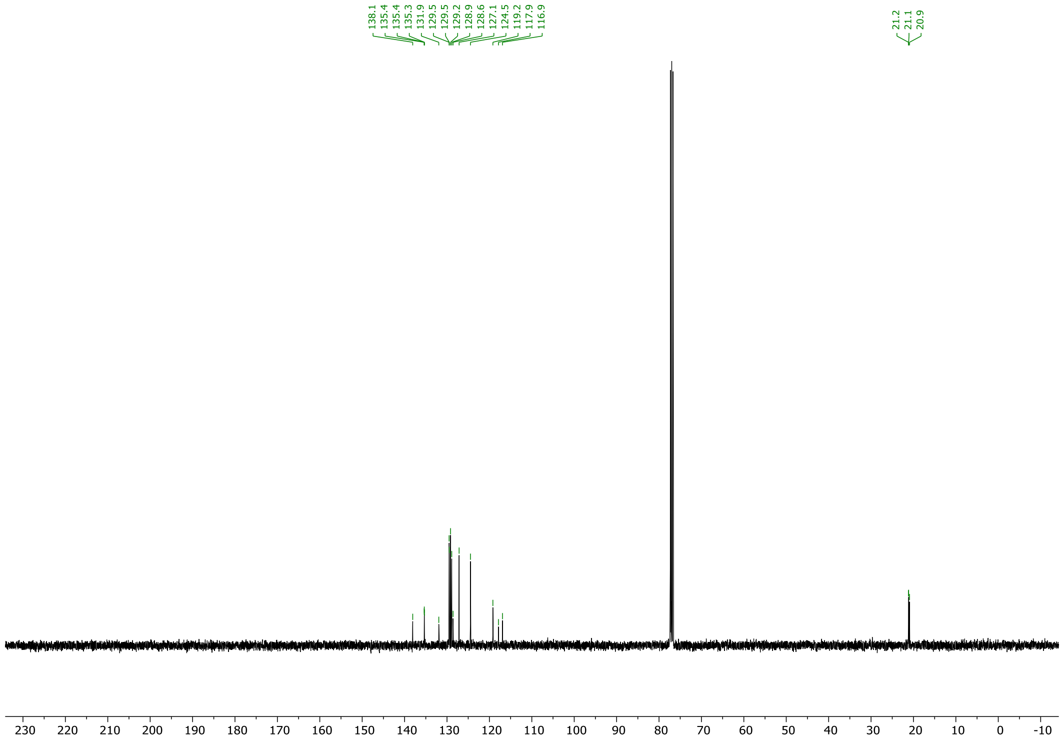

Supplement: Supplementary file 1 — jo2c00434_si_001.pdf [file jo2c00434_si_001.pdf]
